# Three-Component Stereoselective Enzymatic Synthesis of Amino-Diols and Amino-Polyols

**DOI:** 10.1021/jacsau.2c00374

**Published:** 2022-09-05

**Authors:** Grayson J. Ford, Christopher R. Swanson, Ruth T. Bradshaw Allen, James R. Marshall, Ashley P. Mattey, Nicholas J. Turner, Pere Clapés, Sabine L. Flitsch

**Affiliations:** † Manchester Institute of Biotechnology (MIB) & School of Chemistry, The University of Manchester, 131 Princess Street, Manchester M1 7DN, U.K.; ‡ Biological Chemistry Department, Institute for Advanced Chemistry of Catalonia, IQAC−CSIC, 08034 Barcelona, Spain

**Keywords:** biocatalysis, enzyme cascades, FSA, aldolase, IRED, stereoselective synthesis, amino-diol, amino-polyol

## Abstract

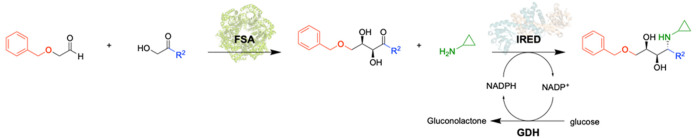

Amino-polyols represent attractive chemical building blocks but can be challenging to synthesize because of the high density of asymmetric functionalities and the need for extensive protecting-group strategies. Here we present a three-component strategy for the stereoselective enzymatic synthesis of amino-diols and amino-polyols using a diverse set of prochiral aldehydes, hydroxy ketones, and amines as starting materials. We were able to combine biocatalytic aldol reactions, using variants of d-fructose-6-phosphate aldolase (FSA), with reductive aminations catalyzed by IRED-259, identified from a metagenomic library. A two-step process, without the need for intermediate isolation, was developed to avoid cross-reactivity of the carbonyl components. Stereoselective formation of the 2*R*,3*R*,4*R* enantiomers of amino-polyols was observed and confirmed by X-ray crystallography.

Chiral polyhydroxylated amines are important pharmacophores that occupy a distinct chemical space in terms of log *P*, molecular weight, hydrogen bonding, and other physicochemical properties.^1−3^ Amino-diols and amino-polyols are present in the backbone of many bioactive compounds, including pactamycin^4^ (antitumor), myriocin^5^ (antibiotic), the proteasome inhibitor TMC-95A,^6^ and imino sugars,^7^ many of which are potent glycosidase inhibitors, including miglustat and miglitol, which are in clinical development. These imino sugars have been the targets for many elegant chemical syntheses^8−12^ but require lengthy routes due to protection strategies, all of which limit scale-up and wider industrial application.

Biocatalysis has proven to be an effective alternative to traditional synthetic techniques for the preparation of chiral amines due to the mild conditions under which enzymes typically operate, while providing excellent regio-, chemo-, and stereoselectivity.^13,14^

Transaminases (TAs),^15−22^ amine dehydrogenases (AmDHs),^23^ imine reductases (IREDs),^24−29^ and amine oxidases (MAOs)^30^ have all been utilized for the preparation of enantiopure amines, which prompted us to investigate their use for amino-polyol formation.

A retrosynthetic analysis^31^ suggested that the most direct route to amino-diols would require imine reductase (IRED) catalysis for the final step with 2,3-dihydroxy ketones as substrates, which in turn can be generated by aldolase-catalyzed biotransformations from aldehydes and hydroxy ketones^12^ (Figure 1). However, this strategy would present a number of challenges to overcome. First, any unreacted aldehyde or ketone from the initial aldolase-catalyzed reaction is also a potential substrate for the IRED, resulting in side-product formation. Second, the aldolase-catalyzed reaction is reversible, which precluded a simple overall “one-pot” process.^32^ Third, there was no precedent for using IREDs on multifunctional polar dihydroxy ketones. A further concern was that the dihydroxy ketone intermediates might be prone to epimerization. Finally, the diol intermediates and products are highly polar and hence extraction from water would be a challenge.

**Figure 1:**
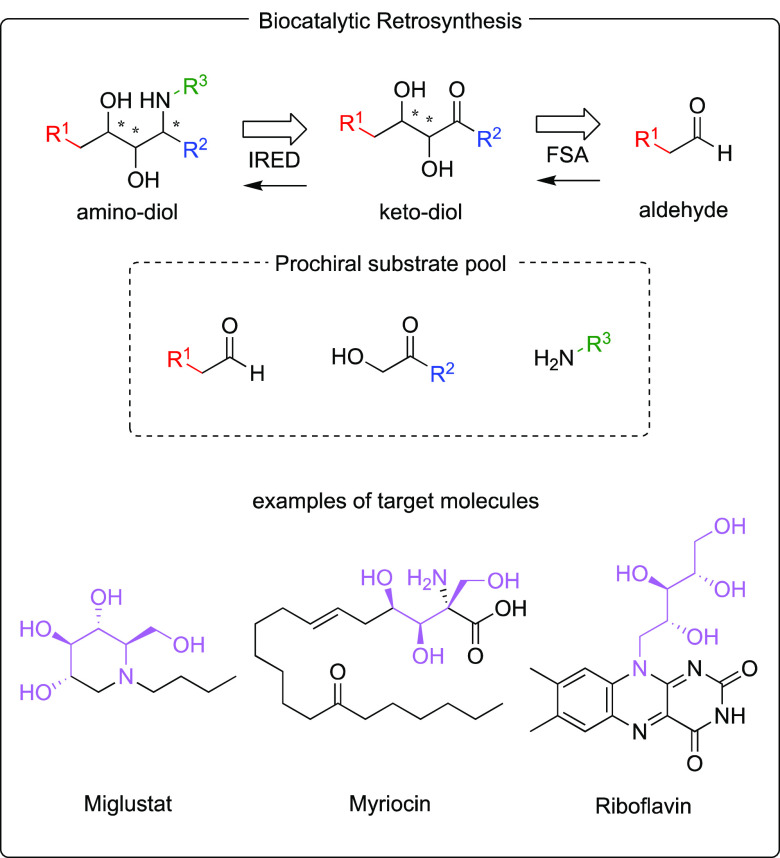
Proposed FSA-IRED cascade for the synthesis of amino-diol targets and examples of target molecules.

Despite these concerns, the simplicity of the two-step strategy in Figure 1 was very attractive and prompted us to look for suitable biocatalysts that might have the required selectivities. For the first step in the proposed cascade the d-fructose-6-phosphate-aldolase (FSA) from *Escherichia coli* was of interest, since it readily accepts dihydroxyacetone (DHA) analogues as donors while maintaining complete control over the stereoselectivity of the reaction.^12,33−38^

Wild-type FSA (WT) and variants A129S, A165G, and A129S/A165 were screened for initial aldol addition using aldehydes **1**–**6** with donor molecules hydroxyacetone (HA, **a**), dihydroxyacetone (DHA, **b**) or hydroxybutanone (HB, **c**) to form aldol adducts **1a**–**6c** (Table 1). These FSA variants have previously been shown to accept phenyl-ring-containing aldehydes, with WT shown to be a good variant with HA and HB as donors and FSA A129S for DHA.^12,34^ The A165G variant has exhibited higher activity toward α-substitued aldehydes. Thus, the double mutant A129S/A165G has shown improved performance in many FSA-mediated reactions.^35,39,40^

**Table 1:**
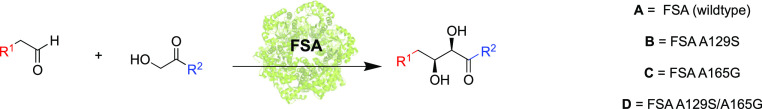

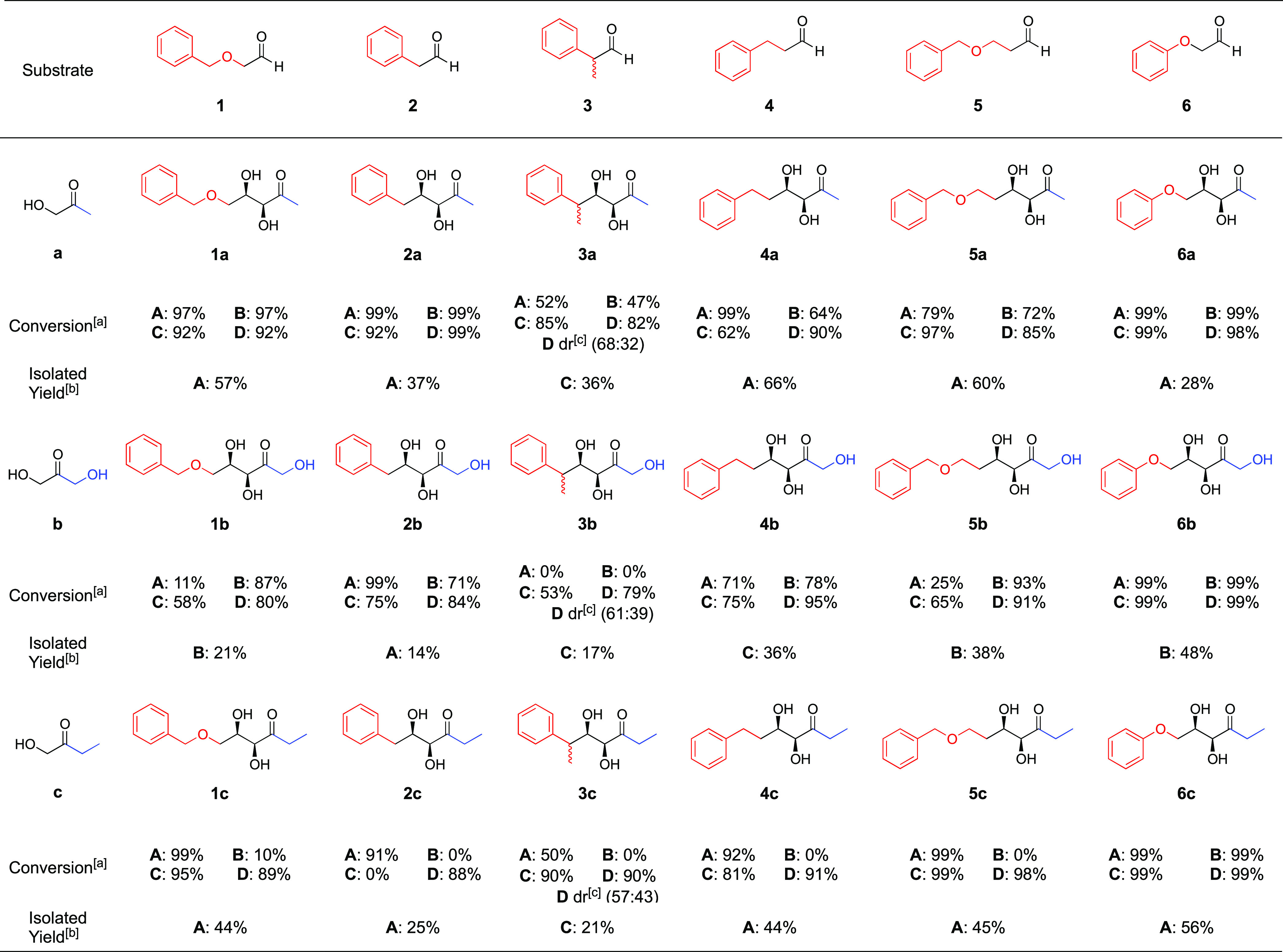
Screening of FSA-Catalyzed Aldol Additions^d^

a Conversions of aldehyde substrates were determined by UPLC-QDa.

b Purification procedures were not optimized and, in some cases, resulted in low isolated yields obtained. Purified products identified as the single diastereomer 3*S*,4*R* with **a** and **b** or 4*S*,5*R* with **c**.

c dr values of products were determined by LC-MS.

d Reaction conditions: 100 mM aldehyde, 100 mM HA/100 mM DHA/100 mM HB, 2 mg mL^–1^ FSA variant lysate, 20% vol/vol DMSO, 100 mM TEA buffer (pH 8), reaction volume 500 μL, 25 °C, 250 rpm for 24 h.

The initial screen revealed conversion for all FSA variants with all aldehyde substrates. FSA WT and A165G showed high conversions of the aldehyde substrates when they were combined with HA or HB as donors, whereas the A129S variants showed a higher affinity when DHA was used as a donor.

Variants that gave the highest substrate conversion for each aldehyde and donor combination were chosen for preparative-scale reactions (total reaction volume 60 mL) with isolated yields for compounds **1a**–**6c** ranging from 14% to 86% (Supporting Information). Isolated yields were considerably lower than the conversions reported, which was attributed to the strong hydrophilic character of the two (or three) hydroxyl groups leading to poor extractability and purification.

Analysis of the purified products by NMR spectroscopy and chromatography showed formation of a single diastereomer, assigned as the 3*S*,4*R* configuration with **a** and **b** and the 4*S*,5*R* configuration with **c**. For products **1a**–**2c** optical rotation values agreed with literature data^12^ (Supporting Information).

The next step required a reductive amination. Although there have been a number of reports on the application of imine reductases (IREDs) for this chemistry, polyols had not been reported as substrates for known IREDs before, and there was concern of cross-reactivity through any ketoreductase activity on the alcohol groups.

To find a biocatalyst with the required stringent selectivity, a metagenomic library of 384 IREDs^41^ was screened using aldol adducts **1a**–**c** and cyclopropylamine **i** as substrates. Conversion of the aldol substrates was determined by HPLC using chemically synthesized amine products **1ai**–**ci**, as mixtures of diastereomers for standards.

Active enzymes obtained from the screening of the IRED plates are shown in Table 2. Pleasingly, 12 active enzymes were obtained for substrate **1a** with up to 94% conversion, while **1b** only gave 4 active enzymes, with only IR-259 showing a high conversion (92%). There were 7 enzymes for substrate **1c**, with IR-259 yielding 65% conversion.

**Table 2:**
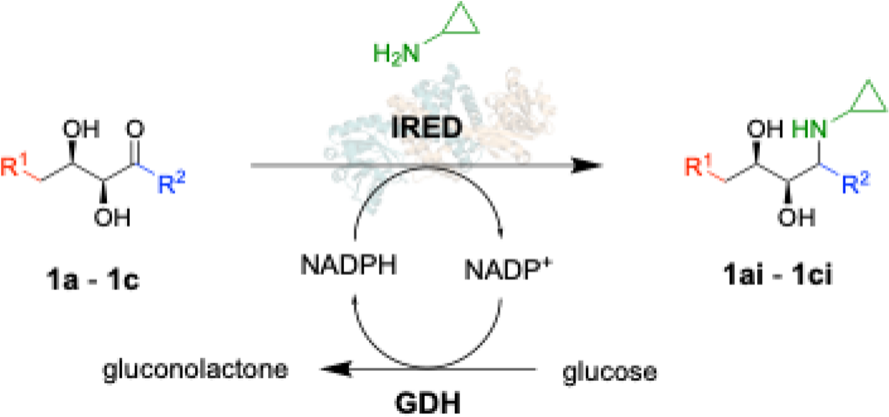

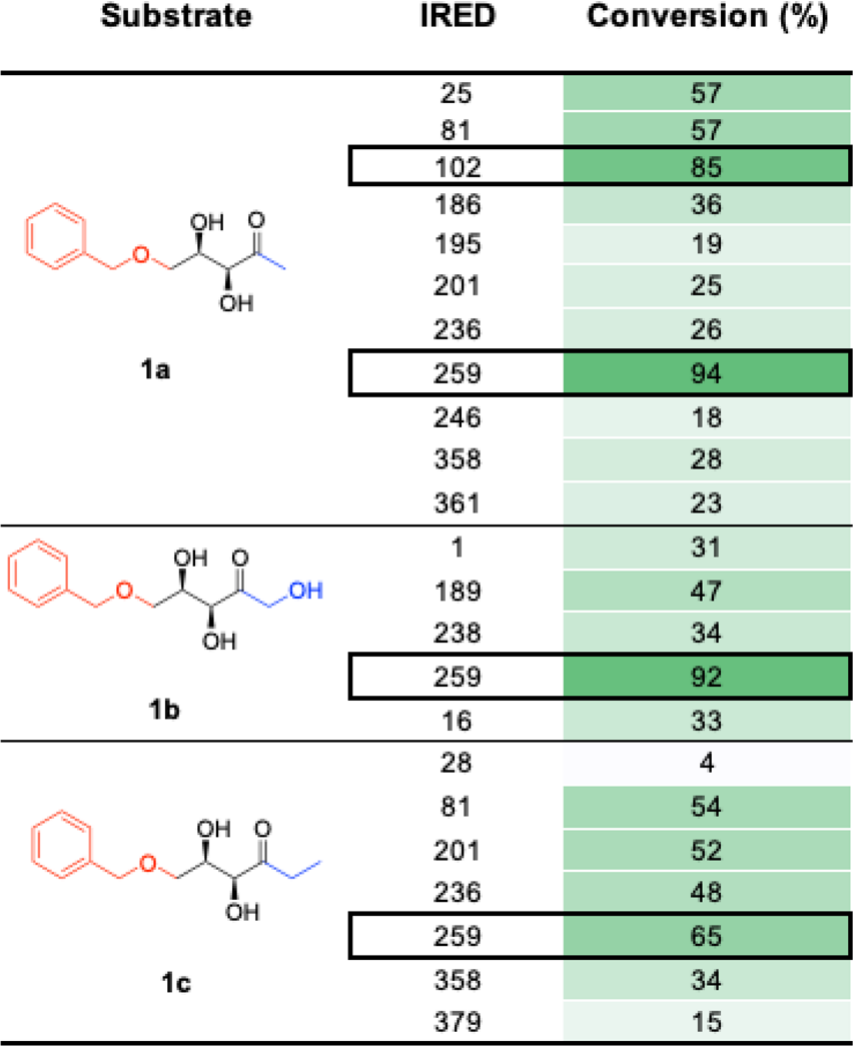
Positive Hits from Screening of a 384-IRED Panel for the Reductive Amination of Aldol Adducts with Cyclopropylamine^a^

a Reaction conditions: 10 mM aldol adduct, 200 mM cyclopropylamine, 0.25 mg mL^–1^ CDX-901 GDH, 50 mM glucose, 5 mg mL^–1^ IRED lysate, 0.5 mM NADP^+^, 10% DMSO, 100 mM TEA buffer (pH 8), 50 μL reaction volume, 30 °C, 700 rpm, 24 h. Conversion of the aldol adduct was determined by reverse-phase HPLC.

Based on a combination of these results, the amine donor scope was then investigated with IR-259 (Table 3 and the Supporting Information). Aldol adducts **1a**–**6c** were subjected to reductive amination with amine donors **i**–**vi**. Conversion of the aldol adducts was determined by UPLC analysis coupled with QDa mass spectrometry.

**Table 3:**
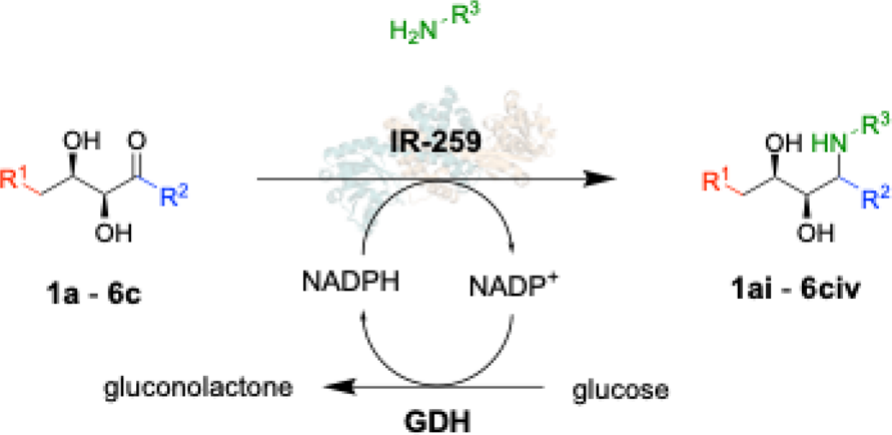

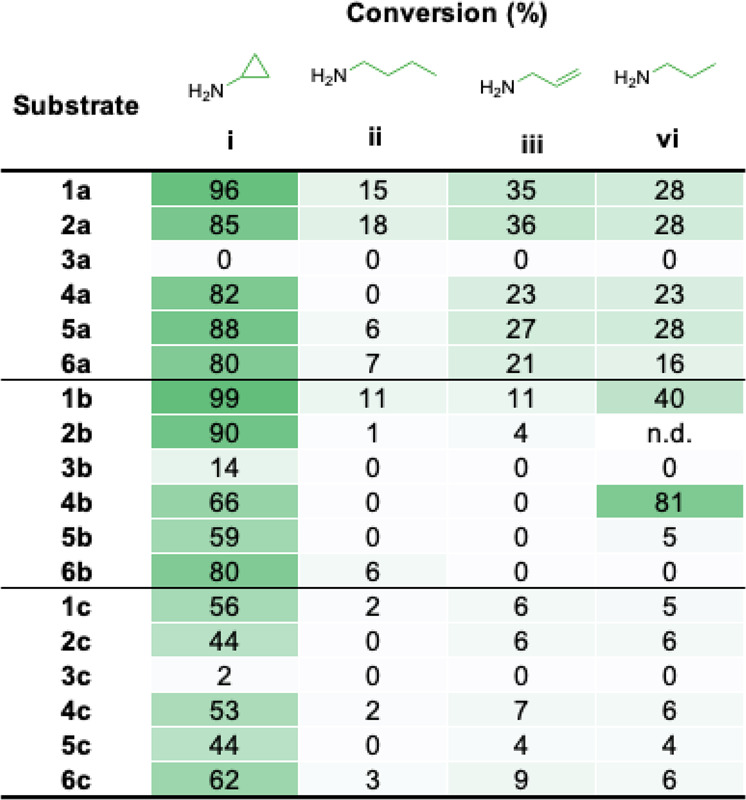
Screening of Amine Donor (i–vi) Scope for IR-259-Catalyzed Reductive Amination of Aldol Adducts **1a**–**6c**^a^

a Reaction conditions: 10 mM aldol adduct, 200 mM cyclopropylamine, 10 mg mL^–1^ IRED lysate, 0.25 mg mL^–1^ CDX-901 GDH, 50 mM glucose, 0.5 mM NADP^+^, 10% DMSO, 100 mM TEA buffer (pH 8), 50 μL reaction volume, 30 °C, 700 rpm, 24 h. Conversion of aldol adduct was determined by UPLC-QDa.

A peak with an [M + H]^+^ of the expected reductive amination product was assumed to be the product in the initial screen. The product formation was highest when hydroxyacetone (**a**) was used as the donor, whereas they were slightly lower for aldol products formed when using dihydroxyacetone (**b**) and significantly lower for (**c**), suggesting that IR-259 exhibits a lower tolerance for substitution adjacent to the carbonyl moiety. Conversions were higher for the smaller amine donors (**i**–**iii** and **vi**) in comparison to benzylamine (**iv**) and propargylamine (**v**), which gave no conversion (Supporting Information).

Reaction conditions were then optimized for the IR-259-catalyzed transformation of **1a** to **1ai** (Table S4 and Figure S2 in the Supporting Information). Amine loading, IRED concentration, and substrate loading were varied while GDH, glucose, and NADP^+^ concentrations were kept constant. High amine concentrations gave the highest conversions with a >90% yield when 200 mM cyclopropylamine was used with 5 mM substrate and 2 mg mL^–1^ of IRED lysate. Good yields were observed at 25 mM **1a** concentration (conversion dropping from 75% to 32% with 50 mM **1a** and 500 mM cyclopropylamine). The overall conversion improved with an increase in IR-259 enzyme concentration, with the highest level being achieved at 10 mg mL^–1^. The optimal pH of the reaction was found to be pH 8. Reaction conditions were also optimized for the conversion of **1a** to **1aiii** using allylamine (**iii**) as the amine donor (Table S5 and Figure S3 in the Supporting Information) leading to 39% conversion.

Enzymatically prepared products (**1ai**–**2ci**) were compared to diastereomeric mixtures generated by chemical reduction of the intermediate dihydroxy ketones, which confirmed that only one diastereomer was formed (Supporting Information).

To test the feasibility of the cascade in one pot, conversions of **1** to **1ai**–**ci**, respectively, were investigated. Aldol adduct formation reached a steady state within 1–2 h and was then consumed by either the reverse aldol reaction or reductive amination with cyclopropylamine. The subsequent reductive amination of the aldol adducts (**1a**–**c**) appeared to be slower and was further limited by unwant,ed reductive amination of **1** forming side product **7** (Figure S4 in the Supporting Information).

To overcome this side-product formation, while minimizing isolation of intermediates, a two-step approach was investigated. In step 1, the FSA components were added to the reaction mixture and after steady-state formation of the aldol product (6 h) the FSA was removed by filtering with centrifugation using a Vivaspin with a 10000 MW cutoff, thereby preventing the reverse aldol reaction from occurring. In step 2, the crude filtrate was treated with cyclopropylamine, IR-259, CDX-901 GDH, glucose, and NADP^+^. This two-step approach improved the formation of the desired products (**1ai**–**6ci**), over side product **7**, while still avoiding the need to purify intermediates (**1a**–**6c**).

This two-step, three-component protocol was then applied to aldehydes **1**–**6** (Table 4). The aldehyde concentration was kept constant (10 mM) while cyclopropylamine concentrations were varied (100, 200, and 500 mM). In general, conversions ranged from 30% to 88% over both steps. Using the optimized conditions, preparative-scale 50 mL reactions for the synthesis of **1ai**–**2ci** were carried out using the previously established two-step approach (Supporting Information). High conversions were recorded for all reactions (85–94%). Isolated yields were considerably lower, again attributed to the strong hydrophilic nature of the products.

**Table 4:**
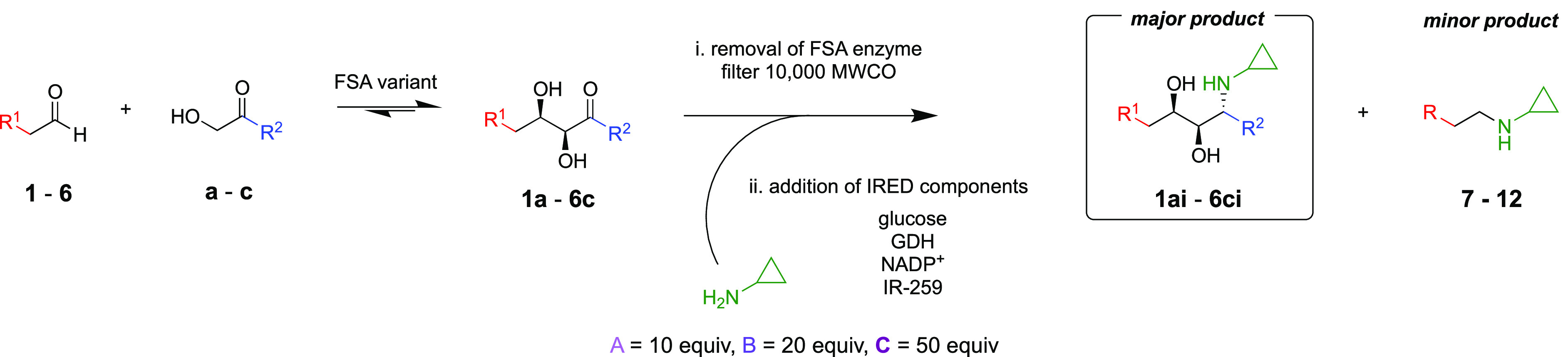

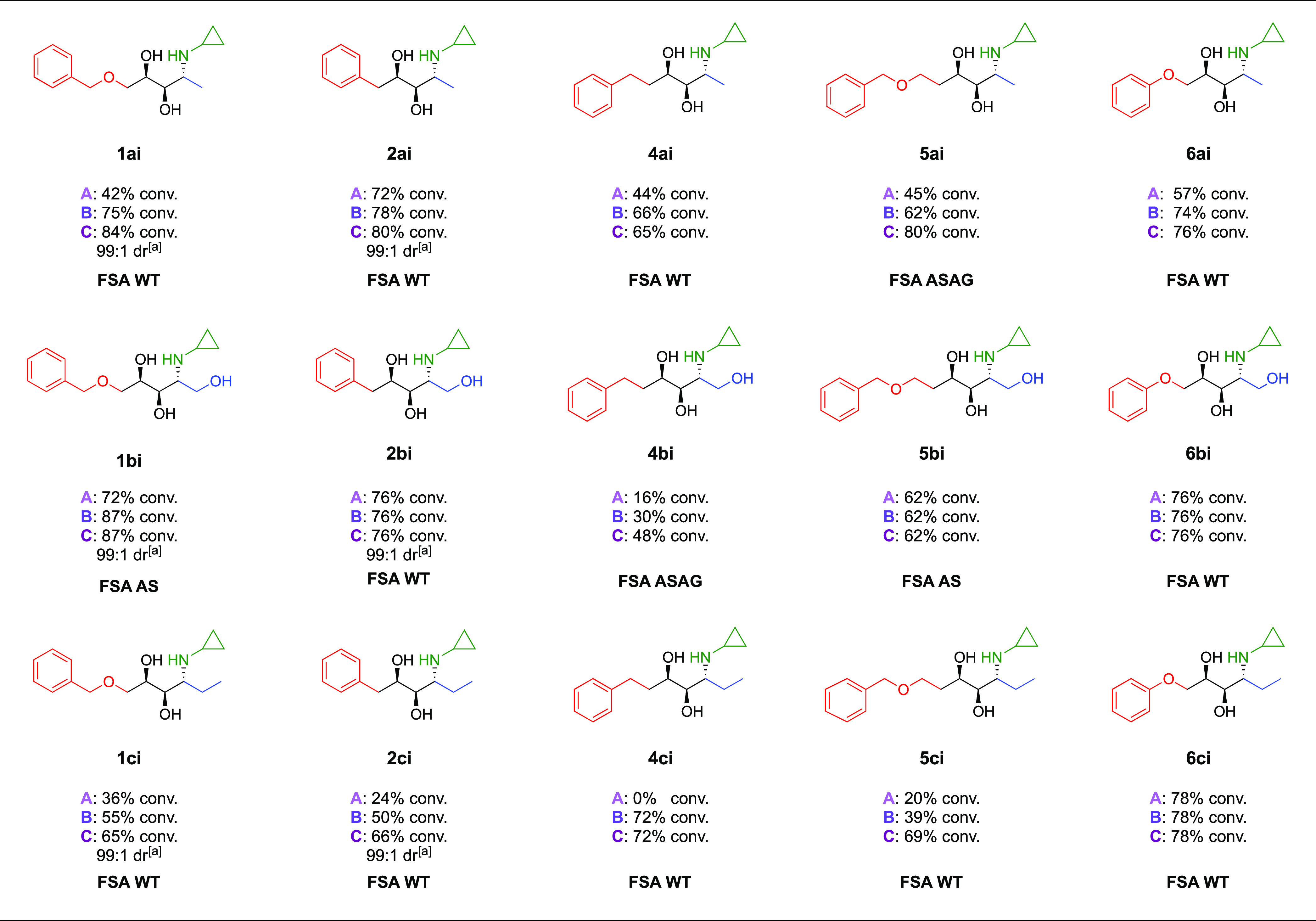
Two-Step Biocatalytic FSA-IRED Cascade toward Amino-Diol and Polyol Products^b^

a dr determined by UPLC-QDa, by comparison of biocatalytically prepared products with chemically synthesized diastereomeric mixtures.

b Reaction conditions: 20 mM aldehyde, 20 mM HA/DHA/HB, 2 mg mL^–1^ FSA variant lysate, 20% vol/vol DMSO, 100 mM TEA buffer (pH 8), 500 μL reaction volume, 250 rpm, 30 °C, 6 h. The FSA was then removed by centrifugation (13000 rpm, 10 min) and the supernatant filtered by Vivaspin (MWCO 10000) by centrifugation (4000 rpm, 10 min). The supernatant was then diluted with the second-step components to a final concentration of ∼10 mM aldol adducts, 100 mM/200 mM/500 mM cyclopropylamine, 10 mg mL^–1^ IR-259 lysate, 0.25 mg mL^–1^ CDX-901 GDH, 1 mM NADP+, 50 mM glucose, 100 mM TEA buffer (pH 8), 10% vol/vol DMSO, 1 mL reaction volume, 30 °C, 200 rpm, 24 h. Conversion was determined by UPLC-QDa.

Products **1ai**–**2ci** were characterized by NMR spectroscopy; **1bi** was recrystallized from MeOH, and single X-ray crystallography data were recorded (CCDC deposition number 2154768, Supporting Information), demonstrating an absolute stereochemistry of 2*R*,3*R*,4*R*. This suggested that the stereochemistry of the aldol product had been conserved and that the previously uncharacterized IR-259 is *R* selective and the reaction can be assumed to form the 2*R*,3*R*,4*R* product. Furthermore, a comparison of optical rotations of the biocatalytically prepared products (**1ai**–**2ci**) with those of the chemically prepared products further suggests that only one diastereomer has formed (Supporting Information).

Given the unusual substrate scope of IR-259, molecular docking studies were performed to better understand substrate specificity. An IR-259 homology model was created based on the reported *Asp*RedAm (PDB 5G6R). Product **1bi** was used as the ligand for the docking simulation (Figure S5 in the Supporting Information). The active site of the model was highly conserved with *Asp*RedAm^26^ containing residues D174, Y182, and W212 previously thought to have a role in catalysis. S237 and S99 are nonconserved and therefore may be responsible for assisting substrate binding through the formation of hydrogen bonds. The IR-259 active site contains a hydrophobic pocket consisting of I178, F185, M181, F223, W212, A219. and I127 which presumably assist in the binding of the phenyl ring of the substrate. Moreover, the docking pose positions the electrophilic carbon of the amine product 4.3 Å from C4 of the NADP(H) cofactor for optimal hydride transfer.

In conclusion, we have successfully established a direct two-step, three-component reaction that converts simple precursors to dihydroxy amine products containing three asymmetric centers with high stereoselectivity. Key to the success was the identification of novel IREDs by screening of a metagenomic panel, which identified an imine reductase (IRED-259) able to tolerate hydroxylated substrates and provide high *R* selectivity. The IRED showed a preference for cyclopropylamine and exhibited modest conversion with other amine donors screened, providing a good platform for further mutagenesis studies. This work demonstrates that biocatalysis is an attractive methodology for the generation of highly functionalized polar amino-polyols using streamlined one-pot sequences without the need for any protecting groups.

## Methods

### Preparation of the Chemically Synthesized Substrates 2-Phenoxyacetaldehyde (**6**)

Sodium periodate (0.65 M in water, 20 mL) was added to a vigorously stirred suspension of silica gel (20 g) in dichloromethane (160 mL), followed by a solution of 3-phenoxy-1,2-propanediol (1.68 g, 10.0 mmol) in dichloromethane (20 mL). After it was stirred for 10 min, the mixture was filtered and the filtrate was concentrated to give the title compound as a clear liquid (1.36 g,100%).

### General Procedure for Reductive Amination for the Synthesis of Amine Standards (**7**–**12**)

To a stirred solution of THF (20 mL) at room temperature were added aldehyde (2.0 mmol) and amine (2.5 mmol). Glacial acetic acid (114 μL, 2.0 mmol) and sodium triacetoxyborohydride (0.636 g, 3.0 mmol) were added sequentially, and the solution was stirred for 16 h. The reaction mixture was quenched with saturated NaHCO_3_ solution (15 mL) and extracted into EtOAc (2 × 10 mL). The organic extracts were washed with HCl solution (1 M, 3 × 15 mL), and the aqueous extracts were then basified to pH 12 with NaOH solution (10 M). The combined aqueous layers were then extracted with EtOAc (2 × 20 mL) and then dried over MgSO_4_. The solvent was removed under rotary evaporation to yield the title compound.

### Alternate General Procedure for Reductive Amination for the Synthesis of Amine Standards (**1ai**–**1ci**)

The aldol product formed and purified from scaled-up FSA reactions (2 mmol) was dissolved in methanol (20 mL) followed by the addition of glacial acetic acid (2 mmol), amine (4 mmol), and sodium triacetoxyborohydride (3 mmol) added portionwise. The mixture was stirred for 16 h, and the crude solution was loaded onto an SCX cartridge. The cartridge was washed with methanol (3 × 20 mL) followed by elution with 7 N ammonina in methanol. The solvent was removed by rotary evaporation to yield the title compound.

### General Procedure for Analytical-Scale Biocatalytic Aldol Addition of Donor Molecules with Aldehydes

Analytical-scale reactions were carried out in 1.5 mL Eppendorf tubes with a reaction volume of 500 μL. Each reaction contained 100 mM aldehyde substrate, 100 mM aldol donor (**a**, **b**, or **c**), 2 mg mL^–1^ FSA variant, 100 mM triethanolamine (TEA) buffer pH 7, and 20% v/v DMSO. Reaction mixtures were shaken at 250 rpm at 25 °C for 24 h before a 3 μL biotransformation sample was taken and diluted with 297 μL of MeOH/0.1% HCl (50/50) to achieve a final concentration of approximately 1 mM. The sample was centrifuged for 10 min at 14000 rpm and placed in a Thompson filter vial for UPLC analysis.

### General Procedure for Preparative-Scale Biocatalytic Aldol Addition of Donor Molecules with Aldehydes

In a 50 mL Falcon tube were placed the components for the FSA reactions (same conditions used in the previous section) with a total reaction volume of 20 mL. Each reaction was set up in triplicate. For each unique aldehyde/donor combination the FSA that gave the highest conversion in the initial screen was chosen. The reaction mixtures were incubated at 25 °C at 250 rpm for 24 h. The reaction mixtures were filtered through Celite to remove protein and then extracted with EtOAc (3 × 80 mL). The organic layer was washed with brine (10 mL), dried over anhydrous MgSO_4_, and concentrated by rotary evaporation. The crude residue was resuspended in minimal EtOAc and purified by flash chromatography (hexane/ethyl acetate 95/5–5/95).

### General Procedure for Analytical-Scale IR-259-Mediated Reductive Amination of Aldol Products

Analytical-scale reactions were carried out in 1.5 mL Eppendorf tubes with a total reaction volume of 500 μL, each containing 10 mM aldol substrate, 200 mM amine donor (from 1 M stock adjusted to pH 8), 0.25 mg mL^–1^ CDX-901 GDH, 50 mM glucose, 0.5 mM NADP^+^, 2 mg mL^–1^ IR-259, 10% vol/vol DMSO, and 100 mM TEA buffer pH 8. Reaction tubes were incubated at 30 °C with shaking at 200 rpm for 24 h before a 30 μL sample was taken from the biotransformation and diluted with MeOH/0.1% HCl (50/50) followed by centrifugation. The sample was then transferred to a Thompson filter vial before UPLC analysis. The pH of the solution was recorded before and after the reaction time, and on each occasion the pH was 8.

### Analytical-Scale Sequential FSA-IR-259 Cascade

The first part of the reactions was performed in a 500 μL reaction mixture containing 20 mM aldehyde, 20 mM aldol donor (**a**, **b**, or **c**), 2 mg mL^–1^ FSA variant lysate, and 20% vol/vol DMSO in TEA pH 8 buffer. Reaction mixtures were incubated at 30 °C at 200 rpm for 6–8 h. Reaction mixtures were then filtered at 4000 rpm for 10 min using a 10000 M_WCO_ Vivaspin prewashed with water. The flowthrough was then diluted up to a total reaction volume of 1 mL so that the components from the first part were diluted by a factor of 2 and now also contained 100/200/500 mM amine, 10 mg mL^–1^ IR-259, 1 mM NADP^+^, 0.25 mg mL^–1^ CDX-801 GDH, 50 mM glucose, 10% vol/vol DMSO, and 100 mM TEA pH 8 buffer in a 1.5 mL Eppendorf tube. Reaction mixtures were incubated at 30 °C with shaking at 200 rpm for 24 h. A 30 μL sample of the reaction mixture was quenched with MeOH/0.1%HCl (50/50), centrifuged at 14000 rpm for 10 min, and added to a Thompson filter vial for UPLC analysis.

### Preparative-Scale Sequential FSA-IR-259 Cascade

A 20 mM portion of aldehyde, 20 mM HA/DHA/HB, 2 mg mL^–1^ FSA variant lysate, 20% vol/vol DMSO, and 100 mM TEA buffer (pH 8) were placed in a 50 mL Falcon tube (25 mL reaction volume) and placed in an orbital shaker at 250 rpm and 30 °C for 6 h. The FSA was then removed by centrifugation (13000 rpm, 10 min) and the supernatant filtered by Vivaspin (M_WCO_ 10000) by centrifugation (4000 rpm, 10 min). The supernatant was then diluted with the second-step components to final concentrations of ∼10 mM aldol product, 100/200/500 mM cyclopropylamine, 10 mg mL^–1^ IR-259 lysate, 0.25 mg mL^–1^ CDX-901 GDH, 1 mM NADP^+^, 50 mM glucose, 100 mM TEA buffer (pH 8), 10% vol/vol DMSO with a 50 mL reaction volume, 30 °C, 200 rpm, and 24 h. The enzymes were precipitated with MeOH and the solvents removed by rotary evaporation. The crude residues were redissolved in minimal H_2_O and purified by a reverse-phase silica column (H_2_O/MeOH 95/5–5/95).

## Supporting Information Available

Experimental procedures including characterization of compounds, UPLC-MS traces of analytical biotransformations, optimization data, control experiments, and NMR spectra (PDF)
