# Supplementary material for: Three-Component Stereoselective Enzymatic Synthesis of Amino-Diols and Amino-Polyols
Source: JACS Au. 2022 Sep 5;2(10):2251–8. doi: 10.1021/jacsau.2c00374 (PMC9597598; doi:10.1021/jacsau.2c00374)
Supplement: Supplementary file 1 — au2c00374_si_001.pdf [file au2c00374_si_001.pdf]

Three-component stereoselective enzymatic synthesis of amino-diols and amino-polyols

Supporting Information

Grayson J. Ford,[a] Christopher R. Swanson,[a] Ruth T. Bradshaw Allen,[a] James R. Marshall,[a] Ashley P. Mattey,[a] Nicholas J. Turner,[a] Pere Clapés,[b] and Sabine L. Flitsch[a] *

[a] Manchester Institute of Biotechnology (MIB) & School of Chemistry, The University of Manchester, 131 Princess Street, Manchester, M1 7DN (UK)

[b] Biological Chemistry Department, Institute for Advanced Chemistry of Catalonia, IQAC−CSIC, 08034, Barcelona, Spain.

*Email: sabine.flitsch@manchester.ac.uk

DOI: XXXX

Table of Contents

Materials & Equipment 5

Analytical Methods 5

Biocatalyst sequences 6

Biocatalyst Production 7

Lysate Preparation 7

Purification 7

Investigation into the substrate scope of FSA to perform aldol addition on aldehyde substrates 7

Identification of IRED to perform the reductive amination of aldol products 9

IREDy-to-go (Reverse-direction screening) 9

General procedure for screening IRED panel (Forward direction) 10

Investigation into the ability of IR-259 to perform reductive amination of aldol products 11

Optimization of reductive amination of 1a with cyclopropylamine and IR-259 12

Investigation into a one-pot FSA-IRED cascade for the reductive amination of aldol compounds 14

Investigation into a two-step sequential addition FSA-IRED cascade for the reductive amination of aldol compounds 15

Investigation into the potential of GDH catalyzed reductive amination of aldehyde or aldol substrates 15

Preparative scale sequential FSA-IR-259 cascade Isolated yields 16

Procedures & Characterization data of preparative biocatalytic formation of Aldol products from aldehydes with donor molecules 16

(3S,4R)-5-(benzyloxy)-3,4-dihydroxypentan-2-one (1a) 16

(3S,4R)-3,4-dihydroxy-5-phenylpentan-2-one (2a) 16

(3S,4R)-3,4-dihydroxy-5-phenylhexan-2-one (3a) 17

(3S,4R)-3,4-dihydroxy-6-phenylhexan-2-one (4a) 17

(3S,4R)-6-(benzyloxy)-3,4-dihydroxyhexan-2-one (5a) 17

(3S,4R)-3,4-dihydroxy-5-phenoxypentan-2-one (6a) 18

(3S,4R)-1,3,4-trihydroxy-5-phenylpentan-2-one (2b) 18

(3S,4R)-1,3,4-trihydroxy-5-phenylhexan-2-one (3b) 18

(3S,4R)-1,3,4-trihydroxy-6-phenylhexan-2-one (4b) 19

(3S,4R)-6-(benzyloxy)-1,3,4-trihydroxyhexan-2-one (5b) 19

(3S,4R)-1,3,4-trihydroxy-5-phenoxypentan-2-one (6b) 19

(4S,5R)-6-(benzyloxy)-4,5-dihydroxyhexan-3-one (1c) 20

(4S,5R)-4,5-dihydroxy-6-phenylhexan-3-one (2c) 20

(4S,5R)-4,5-dihydroxy-6-phenylheptan-3-one (3c) 20

(4S,5R)-4,5-dihydroxy-7-phenylheptan-3-one (4c) 21

(4S,5R)-7-(benzyloxy)-4,5-dihydroxyheptan-3-one (5c) 21
(4S,5R)-4,5-dihydroxy-6-phenoxyhexan-3-one (6c) 21
Procedure & Characterization Data for Preparative biocatalytic reductive amination of amines with aldol products 22
(2R,3R,4R)-1-(benzyloxy)-4-(cyclopropylamino)pentane-2,3-diol (1ai) 22
(2R,3R,4R)-5-(benzyloxy)-2-(cyclopropylamino)pentane-1,3,4-triol (1bi) 22
(2R,3R,4R)-1-(benzyloxy)-4-(cyclopropylamino)hexane-2,3-diol (1ci) 23
(2R,3R,4R)-4-(cyclopropylamino)-1-phenylpentane-2,3-diol (2ai) 23
(2R,3R,4R)-2-(cyclopropylamino)-5-phenylpentane-1,3,4-triol (2bi) 23
(2R,3R,4R)-4-(cyclopropylamino)-1-phenylhexane-2,3-diol (2ci) 24
Characterization Data for chemically synthesized standards 24
Compounds used as substrates in biocatalytic cascade 24
2-phenoxyacetaldehyde(6) 24
Side products of the two-step biocatalytic cascade 24
N-(2-benzyloxy)ethyl)cyclopropanamine (7) 24
N-phenethylcyclopropanamine (8) 24
N-(2-phenylpropyl)cyclopropanamine (9) 25
N-(3-phenylpropyl)cyclopropanamine (10) 25
N-(3-(benzyloxy)propyl)cyclopropanamine (11) 25
N-(2-phenoxyethyl)cyclopropanamine (12) 25
Product standards for two-step biocatalytic cascade 26
(3R,4R)-1-(benzyloxy)-4-(cyclopropylamino)pentane-2,3-diol (1ai) 26
(3R,4R)-5-(benzyloxy)-2-(cyclopropylamino)pentane-1,3,4-triol (1bi) 26
(3R,4R)-1-(benzyloxy)-4-(cyclopropylamino)hexane-2,3-diol (1ci) 26
(3R,4R)-4-(cyclopropylamino)-1-phenylpentane-2,3-diol (2ai) 27
(3R,4R)-2-(cyclopropylamino)-5-phenylpentane-1,3,4-triol (2bi) 27
(3R,4R)-4-(cyclopropylamino)-1-phenylhexane-2,3-diol (2ci) 27
Determination of absolute stereochemistry of biocatalytically prepared 1bi by X-Ray Crystallography 28
Docking Studies 29
Determination of diastereomeric ratio of preparative scale reactions 31
UPLC QDa Chromatograms for determination of conversion 35
Example chromatograms for screening FSAs for the aldol addition reactions 37
Example Chromatograms for determination of dr for FSA mediated aldol reactions with aldehyde 3 46
Example Chromatograms for the IR-259 mediated reductive amination of aldol adduct substrates . 47

Example Chromatograms of one-pot two-step FSA-IR259 cascade. First step (FSA step only)........... *62*

Example Chromatograms of one-pot two-step FSA-IR259 cascade. Second step (IR259 step only).... *69*

Example Chromatograms of preparative scale FSA-IR259 cascade.. *74*

NMR Spectra of Chemically Synthesized Standards and substrates.. *77*

NMR Spectra of Biocatalytically synthesized compounds.. *90*

Materials & Equipment

Commercially available chemicals used were purchased from Sigma-Aldrich (Poole, Dorset, UK), Acros Organics (Loughborough, UK), Alfa Aesar (Heysham, Lancashire, UK), Fluorochem (Hadfield, Derbyshire, UK) and used without further purification.

Commercially available 384 metagenomic IRED panel was purchased from Prozomix (Building 4, West End Ind. Estate, Haltwhistle, UK)

A Bruker Avance 400 spectrometer was used to record NMR spectra with chemical shifts reported in ppm relative to tetramethylsilane (TMS). Coupling constants (J) are reported in Hz.

High-resolution mass spectrometry (HRMS) was recorded using a Waters LCT time-of-flight mass spectrometer, connected to a Waters Alliance LC (Waters, Milford, MA, USA).

Reverse Phase HPLC was carried out on an Agilent 1260 Infinity II Series system equipped with a G1379A degasser, G1312A binary pump, G1367A well plate auto sampler, G1316A temperature-controlled compartment and a diode array detector. A Waters C18, 5 μ m, 4.6 x 250 mm column was used as a stationary phase (Massachusetts, USA).

Reverse phase UPLC was recorded using a Waters Acquity UPLC H-class-QDA System is equipped with a quaternary solvent manager (QSM), autosampler, photodiode array (PDA) detector and single quadrupole QDa mass detector. A Waters Acquity UPLC HSS C18, 1.8 μm, 2.1 x 100 mm column was used as a stationary phase.

Reverse phase LC-MS was recorded using an Agilent 1260 Infinity II Series system with a G1379A degasser, G1312A binary pump, G1367A well plate auto sampler, G1316A temperature-controlled compartment and a diode array detector. This was coupled to an agilent single quadruple LC/MSD.

Analytical Methods

HPLC methods:

Method 1

Time [min]	H_2O (0.1% TFA) [%]	MeOH (0.1% TFA) [%]
0	90	10
20	10	90
20.1	5	95
22	95	5
25	90	10

Method 2

Time [min]	H_2O (0.1% TFA) [%]	MeOH (0.1% TFA) [%]
0	40	60
5	40	60

UPLC methods:

Method 1

Time [min]	H_2O (0.1% DFA) [%]	MeOH (0.1% DFA) [%]
0	90	10
15	10	90
15.1	5	95
16	95	5
18	90	10

Method 2

Time [min]	H_2O (0.1% DFA) [%]	MeOH (0.1% DFA) [%]
0	90	10
25	10	90
25.1	5	95
26	95	5
30	90	10

Method 3

Time [min]	H_2O (0.1% DFA) [%]	MeOH (0.1% DFA) [%]
0	95	5
7	5	95
8	5	95
9	95	5
10	95	5

Biocatalyst sequences

sp|P78055|FSAA_ECOLI Fructose-6-phosphate aldolase 1 OS=Escherichia coli (strain K12) OX=83333 GN=fsaA PE=1 SV=2
MELYLDTSDVVAVKALSRIFPLAGVTTNPSIIAAGKKPLDVVLPQLHEAMGGQGRLFAQV
MATTAEGMVNDALKLRSIIADIVVKVPVTAEGLAAIKMLKAEGIPTLGTAVYGAAQGLLS
ALAGAEYVAPYVNRIDAQGGSGIQTVTDLHQLLKMHAPQAKVLAASFKTPRQALDCLLAG
CESITLPLDVAQQMISYPAVDAAVAKFEQDWQGAFGRTSI

>259
MNVTTPEPTTVAVIGLGNLGQVLARTLLDQGHKVTVWNRSQDKADDLVARGATRAATPAD
AIRASDLVIICVLDYTTVRDLLTPAADALAGRVVVNVTSGIPEPARELATRVNGSGAAYV
DGAVYAIPQTIGTPEAFVLYSGDEEAFARHRALLETLGTAEFVGADAGLAAVHDVALLSG
MYGMFAGFFQTVAVSGSAGIEAVRVTELLVRWLKEAIAALPAFAAEIDAGDYRTQTSNLD
INAVGLANILAATRAQGVGVELLAPLHALFEQQVSAGHGAESLSRTIESLR

>81
MTKTPVSVIGLGRMGATIAEIYLREGHPTTVWNRTPAKAEPLLAKGARPAATVAEAVAAS
PVSVIMLADDTVVRASLESATSELDGRTIVNLTTGRPDEARELGDWLAGHGAEYLDGGVL
GVPQTLATPESVIIYSGSTAANQRHGEIVAALGTARYLGADHGLASLNDMAILSGMYGLF
SGYFHSVAMVDSEGFKAAEFTKDLLIPWLRSIVDVLPTLADEIDSGDYPVNFSNLDVNVA
GIENIMRTSRSQGVTDAPLVPLRDALAAQQEKGHGEASFSRAVEELKSSE

Biocatalyst Production

Chemically competent *E. coli* were transformed with DNA plasmid vectors containing the genes for the relevant enzyme and grown on LB agar plates containing 30 mg mL^{-1} antibiotic (Table S1**.**)

Protein	Organism	Genbank accession	Plasmid	Cell-Strain
FSA WT	*E. coli*	P78055	pET28-a	BL-21
FSA A129S	*E. coli*	-	pET28-a	BL-21
FSA A165G	*E. coli*	-	pET28-a	BL-21
FSA A129S/A165G	*E. coli*	-	pET28-a	BL-21
IR – 81	*Stackebrandtia nassauensis*	-	pET28-a	BL-21
IR – 259	*Streptomyces aureocirculatus*	-	pET28-a	BL-21

A single colony harboring a desired gene (FSA or IRED) was added to 20 mL LB medium containing 20 µL kanamycin (from 35 mg ml^{-1} stock) and incubated overnight at 250 rpm at 37 °C. The preculture was used to inoculate a 2 L baffled flask containing 400 mL of autoclaved TB media and 400 µL kanamycin and incubated at 37°C, 250rpm until an optical cell density (OD_{600}) of 0.6 was reached. Protein expression was induced by inoculating the flask with 400 µL of isopropyl-beta-D-1-thiogalactopyranoside (IPTG, 0.1 M). The flask was further incubated at 23 °C at 200 rpm for 20h. The cells were harvested by centrifugation at 4000 rpm and 4 °C for 30 minutes. The cell pellets were washed with KPi (100mM, pH 7), pelleted again using the same conditions and the supernatant was discarded. The cell pellets were stored at -20 °C in 50 mL falcon tubes until further use.

Lysate Preparation

Cell pellets were re-suspended in KPi (100mM, pH 7), submerged in an ice bath and lysed by ultrasonication (60 sec ON, 90 sec OFF, 40% amplitude, 4 – 6 cycles using a Soniprep 150 (MSE UK Ltd.). The solution was clarified by ultra-centrifugation at 18,000 rpm at 4 °C for 60 min. The clarified supernatant was filtered through a cellulose membrane (0.45 µm) into a 50 mL falcon tube and snap froze using liquid N_2 before freeze drying on a Heto-PowerDry LL1500 (ThermoScientific). The resulting lyophilized powder was stored at -20 °C until further use.

Purification

Lyophilized powder of the desired enzyme was resuspended in 10% buffer B (100 mM KPi, 300 mM NaCl, 30 mM imidazole, pH 7) and loaded onto a His-Trap Crude FF column (GE Healthcare) charged with 0.1 M nickel sulphate, pre-equilibrated with 10% buffer B. The column was washed with 15 – 25 mL of 10 % buffer B and then 15 – 25 mL of 20 % buffer B (100 mM KPi, 300 mM NaCl, 60 mM imidazole, pH 7). The His-tagged protein was then eluted with 100 % buffer B (100 mM KPi, 300 mM NaCl, 300 mM imidazole, pH 7) and 1 – 2 mL fractions were collected. Protein concentration was determined using a thermofisher NanoDrop™ microvolume spectrophotometer and the fractions containing pure protein were combined. The pure purified was added to a membrane 30,000 MWCO centrifugal concentrator (Sartoius, UK) to remove excess imidazole. Buffer A (100 mM NaPi buffer, 300 mM NaCl, pH 7.5) was added to the samples until desired concentration was achieved. The pure protein was divided into 1 mL aliquots, snap frozen and stored at – 80 °C until further use.

Investigation into the substrate scope of FSA to perform aldol addition on aldehyde substrates

A panel of six aldehydes were chosen for testing with FSA variants (WT, A129S, A165G and A129S/A165G) to determine their ability to form aldol products with three separate donors: hydroxyacetone (HA, **a**), dihydroxyacetone (DHA, **b**) and hydroxybutanone (HB, **c**). Aldehyde **1** and **2** is an already known substrate and **3** – **6** were chosen based on structure similarity. Reactions exhibiting high substrate conversions were scaled up.

Table S1: Results of variant FSA screening with aldehydes and donors

Substrate	Donor	Product	FSA Variant used	Isolated Yield (%)
1	a	1a	WT	57
2	a	2a	WT	37
3	a	3a	WT	36
4	a	4a	A165G	66
5	a	5a	WT	60
6	a	6a	WT	28
1	b	1b	A129S	21
2	b	2b	WT	14
3	b	3b	A165G	17
4	b	4b	A129S	36
5	b	5b	A129S	38
6	b	6b	A129S	48
1	c	1c	WT	44
2	c	2c	WT	25
3	c	3c	A165G	21
4	c	4c	WT	44
5	c	5c	WT	45
6	c	6c	WT	56

Identification of IRED to perform the reductive amination of aldol products

IREDy-to-go (Reverse-direction screening)

A previously described colorimetric screen was used to identify an IRED capable of mediating reductive amination with aldol product **1a** and cyclopropylamine **i** to form product **1ai**.

The assay is based on running the reaction in the reverse direction using the amine product of interest as a substrate which is oxidized to the corresponding imine. This is coupled with a reaction forming a red compound formazan which is formed from the NADPH dependent oxidation of diaphorase. The formation of formazan can be monitored by a plate reader at 490 nm which gives an indication of activity of the reductive amination process.

Protocol:

A 384 well plate was used to carry out the assay. Each well contained lyophilized IRED lysate and IREDy-to-go components ($NADP^+$ and Diaphorase). 50 µL of the assay master mix (0.125 mg ml^{-1} INT, 10 mM amine in Tris-HCl buffer pH 9) was added to each well, changing tips in between each addition to minimize contamination between enzymes. The plate was centrifuged at 1,500 rpm for 1 minute. A plate reading measurement was taken as a 0 hr measurement and then the plate was covered in foil and incubated at 30 °C for 24 hr. Another plate reading was taken at 24 hr. A blank plate was also run under the same conditions, but without the amine to eliminate false positives. (**Figure S1**)

The IREDy-to-go yielded a lot of results for amine **1ai**, but when tested in the forward direction many of the hits from the assay showed no activity. We hypothesized that the hydroxyl groups present on our substrate may interfere with the screen as there may be endogenous alcohol dehydrogenases within the IRED lysate.

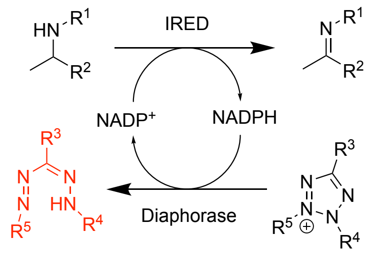

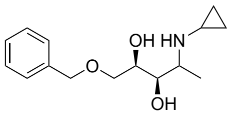

1ai

<>	1	2	3	4	5	6	7	8	9	10	11	12	13	14	15	16	17	18	19	20	21	22	23	24
A	0.144	0.390	0.313	0.590	0.379	0.552	0.262	0.630	0.380	0.955	0.516	0.766	0.434	0.877	1.230	0.727	0.885	1.051	0.574	0.961	0.815	1.028	1.901	1.578
B	0.199		0.242	0.241	0.304	0.519	-0.094	0.291	0.054	0.554	0.240	0.298	0.075	0.332	0.445	0.613	0.450	0.451	0.615	0.610	0.343	0.877	1.280	0.611
C	0.243	0.042	0.215	-0.069	0.139	0.000	0.278	0.098	0.465	0.203	0.548	0.125	0.548	0.196	0.392	0.211	0.394	0.319	0.300	0.271	0.466	0.674	0.457	0.373
D	0.162	0.043	-0.290	0.052	0.300	0.107	0.305		0.812	0.245	0.688	0.271	0.505	0.442	0.540	0.101	0.437	0.269	0.576	0.275	0.589	0.000	0.668	0.120
E	0.389	0.158	0.177	0.108	0.203	0.313	0.322	0.116	0.433	-0.121	0.317	-0.360	0.559	0.063	0.588	-0.069	0.261	-0.419	0.278	0.364	0.524	0.051	0.584	-0.281
F	0.262	0.220	0.149	0.224	0.004	0.130	0.687	0.204	0.103	0.335	0.306		0.195	0.672	0.019	0.540	-0.080	0.618	0.330	0.835	0.154	1.040	0.340	0.638
G	0.412	0.607	0.201	0.250	0.069	0.502	0.193	0.408	0.259	0.238	0.170	0.717	0.143	0.850	0.502	0.475	0.643	1.171	0.363	0.653	0.377	1.157	0.512	1.334
H	0.387	0.542	0.197	0.638	0.149	1.092	0.251	0.327	0.245	0.515	0.588	0.719	0.708	0.804	0.394	0.728	0.356	0.465	0.432	1.000		0.574	0.879	0.435
I	0.888		1.799	1.025	0.312	0.975		0.850	0.336	0.951	0.060	0.488	0.309	0.509	0.717	0.631	0.634	0.586	0.439	0.857	1.144	1.388	0.950	1.046
J	0.855	0.563	1.005	0.702	0.860	0.765	0.613	0.647	0.804	0.565	1.113	0.465	0.548	0.803	0.874	0.581	1.205	1.037	0.604	0.546	0.503	0.907	0.641	0.906
K	0.429	0.869	0.421	0.434	0.365	-0.006	0.101	-0.027	0.393	0.087	0.560	0.619	0.833	1.406	0.157		0.245	0.546	0.449	0.963	0.498	0.995	0.726	1.330
L	0.788	0.437	0.541	0.707	1.295	0.710	0.584	0.236	0.484	0.403	0.815	0.686	0.795	0.365	0.582	0.592	0.724	0.478	0.379	0.292	1.376	0.513	1.349	0.549
M	1.357	0.541	0.472	0.306	0.350	0.398	0.461	-0.200	0.547	0.924	0.156	0.473	0.387	0.272	0.421	-0.501	0.320	0.300	0.351	0.058	0.645	0.340	0.720	0.347
N	0.495	0.384	0.416	0.178	0.434	0.316	0.404	0.537	0.347	0.546	0.806	0.362	0.661	1.055	0.568	0.692	0.946	0.435	0.796	0.861	0.549	0.564	1.181	0.921
O		0.423	0.184	0.513	0.543	0.279	0.599		0.276	1.199	0.558	0.811	0.991	0.603	0.546	0.874	0.636	0.324	0.875	0.432	0.782	0.439	1.015	0.637
P	0.359	0.557	0.269	0.565	0.828	1.255	1.075	0.426	1.106	1.094	1.071	1.472	0.495	0.818	0.969	1.355		1.850	0.849	0.704	0.720	1.552	1.168	1.429

Figure S1: Top: picture of diaphorase reaction coupled to reductive aminase oxidation, resulting in production of red formazan dye. Bottom: Results of IREDY-2GO screen for compound **1ai** after 24 h incubation. False Positives often reported at B02, D08, F12, H21, I02, I07, K16, O01, O08 and P17.

General procedure for screening IRED panel (Forward direction)

As an alternative, forward direction reductive aminations were setup for substrates **1a**, **1b** and **1c** with cyclopropylamine as an amine donor. To a 96 well plate, each well contained 10 mM aldol substrate, 200 mM cyclopropylamine, 0.25 mg ml^{-1} CDX-801 GDH, 50 mM glucose, 0.5 mM NADP$^+$, 5 mg ml^{-1} IRED, 10 % vol/vol DMSO and 100 mM TEA buffer pH 8 to a total reaction volume of 100 µL. Samples were placed on a plate shaker at 30 °C, 700 rpm for 24 hr. 20 µL of the biotransformations filtered with a 96-well filter plate and centrifuged at 4,000 rpm for 10 min. The filtered biotransformation was then diluted with 180 µL MeOH: 0.1 % HCl (50:50) and run on reverse phase HPLC directly from the plate. Results presented below in **Table S2**.

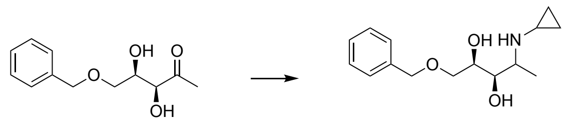

1a **1ai**

Table S2: Results of variant FSA screening with aldehydes and donors

IRED	Conversion (%)
25	57
81	57
102	85
186	36
195	19
201	25
236	26
259	94
246	18
358	28
361	23

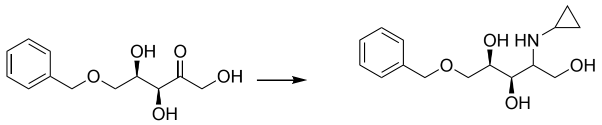

1b **1bi**

IRED	Conversion (%)
1	31
189	47
238	34
259	92

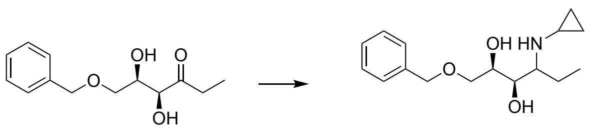

1c **1ci**

IRED	Conversion (%)
16	33
28	4
81	54
201	52
236	48
259	65
358	34
379	15

Investigation into the ability of IR-259 to perform reductive amination of aldol products

Table S3:Initial screening results for the reductive amination of aldol product substrates

**Substrate generated by aldol reaction**	Conversion with amine donor	
	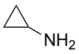	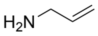
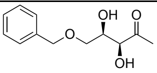	91%	22%
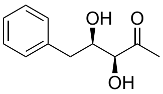	66%	24%
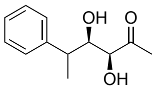	5%	0%
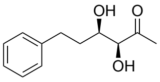	40%	12%
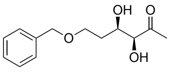	60%	10%
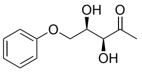	n.d.	n.d.
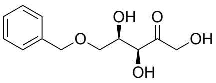	90%	0%
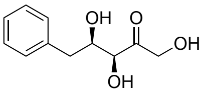	68%	0%
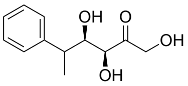	14%	4%
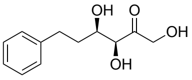	38%	0%
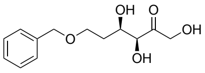	27%	0%
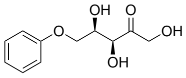	46%	0%
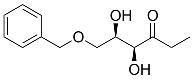	47%	0%
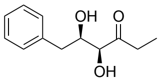	32%	0%
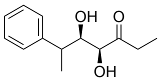	13%	0%
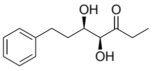	47%	0%
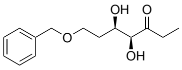	32%	0%
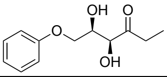	40%	0%

Optimization of reductive amination of 1a with cyclopropylamine and IR-259

Taking the biotransformation of **1a** to **1ai** and **1a** to **1aiii** as a model reaction, components were varied to investigate which components have the largest influence over conversion.

Table S4: Optimization of reductive amination of substrate **1a** with cyclopropylamine (**i***)*

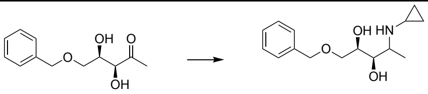

1a **1ai**

Entry	[Aldol] [mM]	[Amine] [mM]	[IRED] [mg ml^{-1}]	Buffer/pH	Temperature [ºC]	Conversion [%]
1	5	50	2	TEA (8)	30	75
2	10	100	2	TEA (8)	30	76
3	25	250	2	TEA (8)	30	75
4	50	500	2	TEA (8)	30	32
5	5	5	2	TEA (8)	30	22
6	5	25	2	TEA (8)	30	44
7	5	50	2	TEA (8)	30	75
8	5	250	2	TEA (8)	30	88
9	5	500	2	TEA (8)	30	71
10	5	100	0.5	TEA (8)	30	54
11	5	100	1	TEA (8)	30	65
12	5	100	2	TEA (8)	30	87
13	5	100	4	TEA (8)	30	86
14	10	100	2	TEA (7)	30	39
15	10	100	2	TEA (7.5)	30	68
16	10	100	2	TEA (8)	30	76
17	10	100	2	Tris-HCl (9)	30	64
18	10	200	2	TEA (8)	30	91
19	10	200	10	TEA (8)	30	96

Figure S2:Optimisation data for the reductive amination of **1a** with **i**.

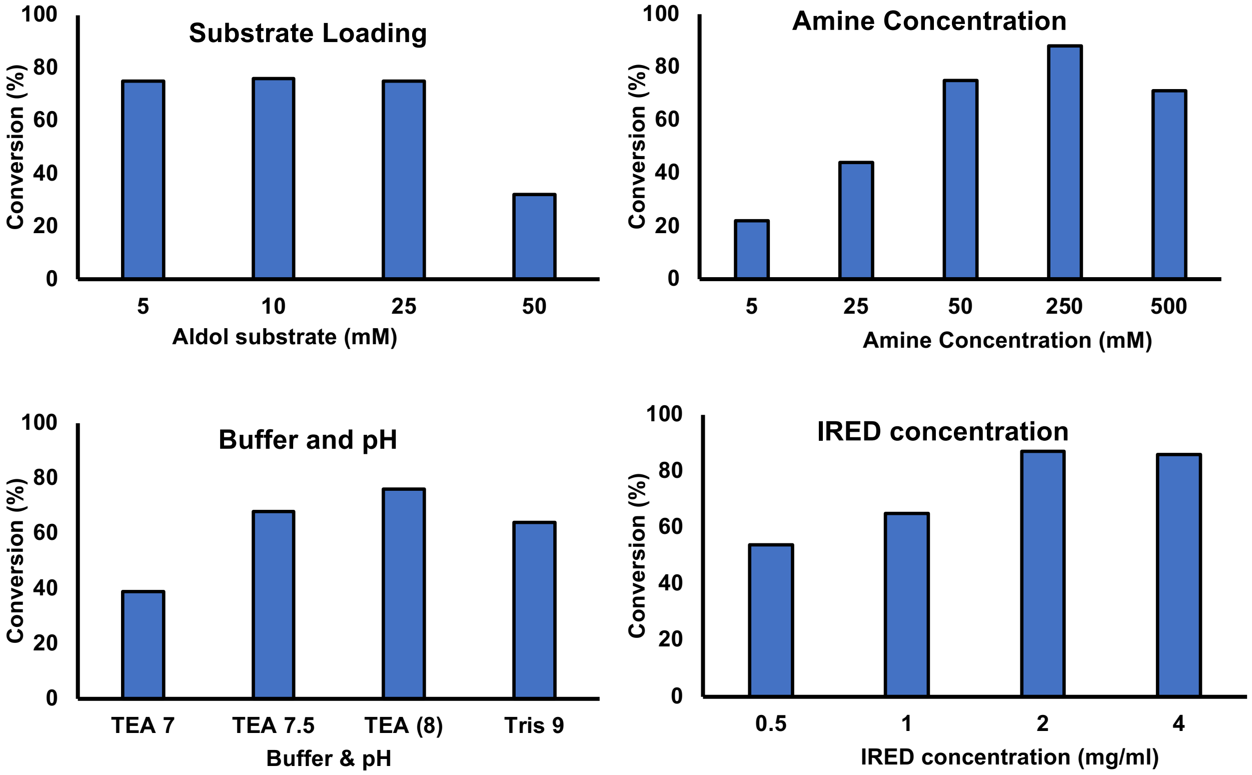

Table S5: Optimization of reductive amination of substrate **1a** with allylamine (**iii**)

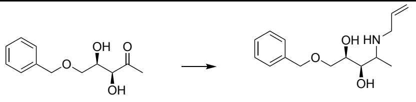

1a **1aiii**

Entry	[Aldol] [mM]	[Amine] [mM]	[IRED] [mg ml^{-1}]	Buffer/pH	Temperature [°C]	Conversion [%]
1	5	100	2	TEA (8)	30	7
2	5	150	2	TEA (8)	30	6
3	5	200	2	TEA (8)	30	6
4	5	250	2	TEA (8)	30	5
5	5	500	2	TEA (8)	30	5
6	5	100	5	TEA (8)	30	16
7	5	150	5	TEA (8)	30	18
8	5	200	5	TEA (8)	30	19
9	5	250	5	TEA (8)	30	18
10	5	500	5	TEA (8)	30	17
11	5	100	10	TEA (8)	30	22
12	5	150	10	TEA (8)	30	31
13	5	200	10	TEA (8)	30	35
14	5	250	10	TEA (8)	30	36
15	5	500	10	TEA (8)	30	39

Figure S3: Optimisation data for the reductive amination of **1a** with **iii**, altering amine concentration and IR-259 lysate

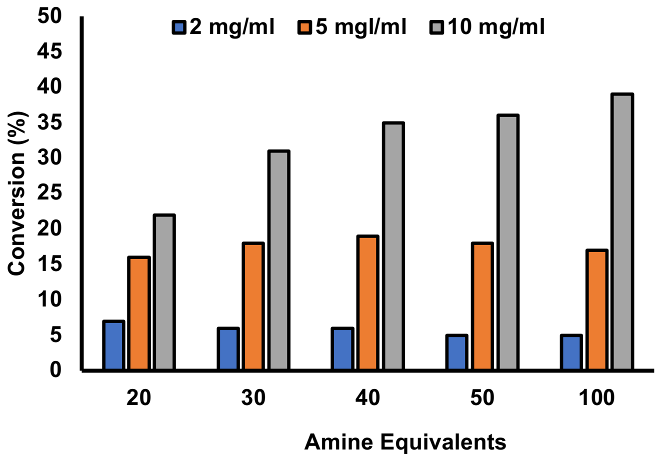

Investigation into a one-pot FSA-IRED cascade for the reductive amination of aldol compounds

The transformation of aldehyde **1** to desired amine product **1ai** was tested first to determine the feasibility of the cascade in one-pot. Reactions were performed in a 500 µL reaction mixture containing 10mM aldehyde, 10mM aldol donor (**a**, **b** or **c**), 2mg ml^{-1} FSA variant lysate, 200mM amine, 2mgml^{-1} IR-259, 1mM $NADP^{+}$, 0.25mg ml^{-1} CDX-801 GDH, 50mM glucose, 10% vol/vol dmso and 100mM TEA pH 8 buffer in a 1.5mL Eppendorf tube. Reactions were incubated at 30ºC with shaking at 200rpm for 24h. 30µL of reaction sample was quenched with MeOH:0.1%HCl (50:50), centrifuged at 14,000rpm for 10 min and added to a Thompson filter vial for UPLC analysis.

Figure S4: Time course of one-pot FSA-IR259 cascades.

A. FSA Variant used = WT

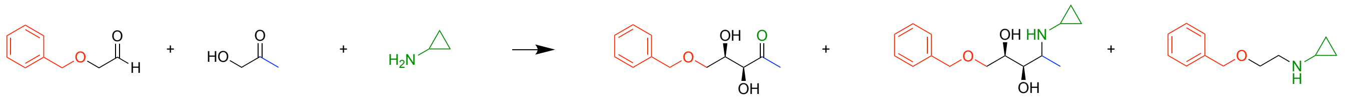

1 **a** **1a** **1ai** **7**

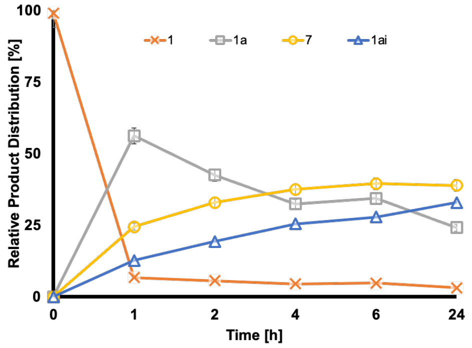

B. FSA Variant used = A129S

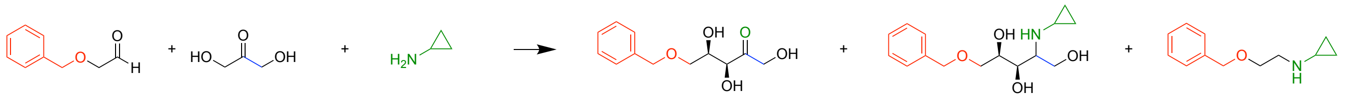

1 **b** **1b** **1bi** **7**

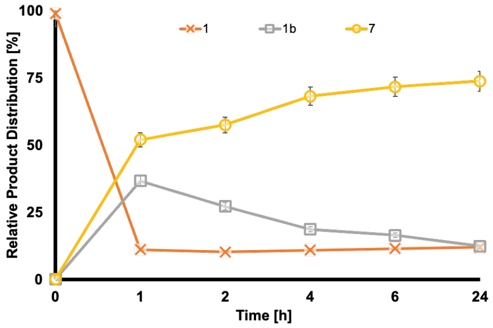

C. FSA Variant used = WT

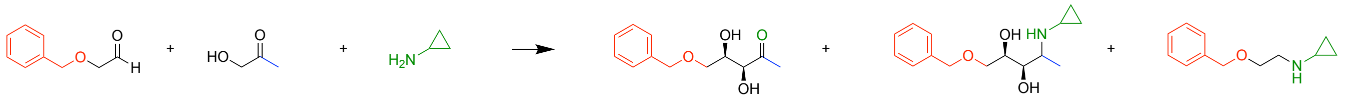

1 **a** **1a** **1ai** **7**

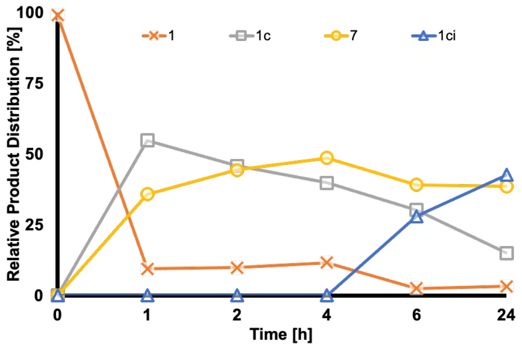

Investigation into a two-step sequential addition FSA-IRED cascade for the reductive amination of aldol compounds

The first part of the reactions was performed in a 500 µL reaction mixture containing 20mM aldehyde, 20mM aldol donor (**a**, **b** or **c**), 2mg ml^{-1} FSA variant lysate and 20% vol/vol DMSO in TEA pH 8 buffer. Reactions were incubated at 30ºC at 200rpm for 6 – 8hr. Reactions were then filtered at 4,000rpm for 10min using a 10,000 MWco Vivaspin pre-washed with water. The flow through was then diluted up to a total reaction volume of 1mL so that the components from part one was diluted by a factor of 2 and now also containing 100/200/500mM amine, 10mgml^{-1} IR-259, 1mM $NADP^+$, 0.25mg ml^{-1} CDX-801 GDH, 50mM glucose, 10% vol/vol dmso and 100mM TEA pH 8 buffer in a 1.5mL Eppendorf tube. Reactions were incubated at 30ºC with shaking at 200rpm for 24h. 30µL of reaction sample was quenched with MeOH:0.1%HCl (50:50), centrifuged at 14,000rpm for 10 min and added to a Thompson filter vial for UPLC analysis.

Investigation into the potential of GDH catalyzed reductive amination of aldehyde or aldol substrates

To investigate the possible interference in the reductive amination step of the cascades of possible imine reduction activity of the CDX-901 GDH enzyme used to recycle glucose, control reactions were set up in which aldehyde or aldol substrates were incubated with GDH lysate and stoichiometric NADPH. Reactions contained 10mM Aldehyde or Aldol, 200mM amine, 10mM NADPH, 0.25 mg mL^{-1} GDH, 10% (v/v) DMSO, 100mM pH 8 TEA buffer, with a reaction volume of 500 µL that were incubated at 30ºC with shaking at 200rpm for 24hr.

Table S6: Data to test for reductive amination of CDX-901 GDH

Entry	Aldehyde or Aldol	Amine	CDX-901 GDH Conversion [%]
1	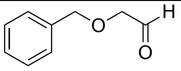	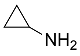	0
2	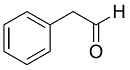	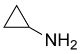	0
3	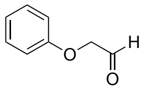	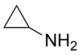	0
4	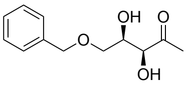	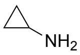	0
5	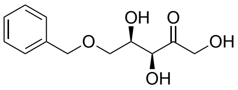	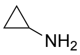	0
6	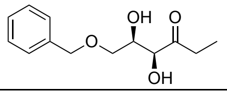	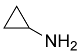	0

Preparative scale sequential FSA-IR-259 cascade Isolated yields

Table S7: Data for preparative scale reactions for the sequential addition FSA-IR259 cascade.

Entry	Aldehyde	Donor	FSA Variant	Amine equivalents	Product	Product	Conversion (%)	Isolated Yield (%)
**1**	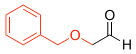	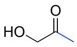	WT	20	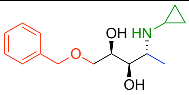	**1ai**	93	24
**2**	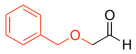	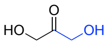	A129S	20	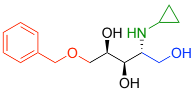	**1bi**	89	16
**3**	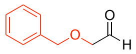	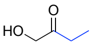	WT	50	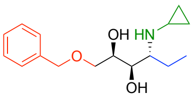	**1ci**	86	30
**4**	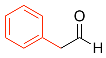	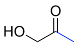	WT	20	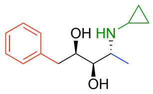	**2ai**	94	12
**5**	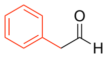	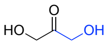	WT	20	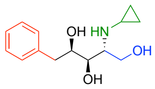	**2bi**	92	26
**6**	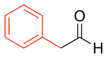	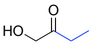	WT	50	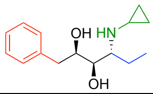	**2ci**	92	14

Procedures & Characterization data of preparative biocatalytic formation of Aldol products from aldehydes with donor molecules

(3S,4R)-5-(benzyloxy)-3,4-dihydroxypentan-2-one (**1a**)

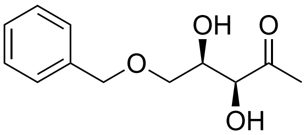

To a 100mL conical flask, 2-(benzyloxy)acetaldehyde (6 mmol), hydroxyacetone (6 mmol), DMSO (12 mL) and 100 mM triethanolamine buffer pH 8 (47 mL) was added. 120 mg of FSA WT lysate was added, and the flask was sealed with a foam bung and incubated at 30 °C in an orbital shaker at 250rpm for 24 hr. The enzyme was precipitated by the addition of EtOAc (20 mL) and removed by filtration through Celite®. The reaction mixture was extracted with ethyl acetate (3 x 30 mL), dried over anhydrous $MgSO_4$ and the solvent evaporated under vacuum. The crude product was purified by flash chromatography on silica gel (EtOAc/Cyclohexane from 1:4 to 1:0) to yield a colorless gel (769mg, 57%).

^{1}H NMR (400 MHz, MeOD) δ 7.39 – 7.21 (m, 5H), 4.55 (s, 2H), 4.20 (d, J = 2.3 Hz, 1H), 4.18 – 4.12 (m, 1H), 3.67 – 3.61 (m, 1H), 3.57 – 3.52 (m, 1H), 2.22 (s, 3H). **^{13}CNMR** (101 MHz, MeOD) δ 211.9, 139.5, 129.3, 128.9, 128.7, 78.7, 74.3, 71.9, 71.8, 26.6. **Optical Rotation** [*α*]D = +35.38 (*c* = 0.52 in DCM) **UPLC-MS** (**QDA**) $[M+Na]^+$ seen 247.04

(3S,4R)-3,4-dihydroxy-5-phenylpentan-2-one (**2a**)

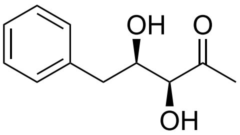

The amounts of reactants used were the following: 2-phenylacetaldehyde (6mmol), hydroxyacetone (6 mmol), DMSO (12 mL) and 120mg of FSA WT lysate dissolved in 100 mM triethanolamine buffer pH 8 (47 mL). The crude product was purified by flash chromatography on silica gel (EtOAc/Cyclohexane from 1:4 to 1:0) to yield a white solid (439mg, 37%).

^{1}H NMR (400 MHz, MeOD) δ 7.32 – 7.10 (m, 5H), 4.13 (td, J = 7.2, 2.1 Hz, 1H), 3.90 (d, J = 2.1 Hz, 1H), 2.98 – 2.78 (m, 2H), 2.14 (s, 3H). **^{13}CNMR** (101 MHz, MeOD) δ 212.3, 139.8, 130.5, 129.39, 127.3, 79.7, 74.7, 40.9, 26.5. **Optical Rotation** [*α*]D = +48.8 (*c* = 0.25 in MeOH) **UPLC-MS** (**QDA**) $[M+Na]^+$ seen 217.04.

(3S,4R)-3,4-dihydroxy-5-phenylhexan-2-one (**3a**)

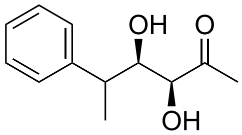

The amounts of reactants used were the following: 2-phenylpropanal (6mmol), hydroxyacetone (6 mmol), DMSO (12 mL) and 120mg of FSA A165G/A129S lysate dissolved in 100 mM triethanolamine buffer pH 8 (47 mL). The crude product was purified by flash chromatography on silica gel (EtOAc/Cyclohexane from 1:4 to 1:0) to yield a clear oil (449mg, 36%) with minor impurities.

^{1}H NMR (400 MHz, MeOD) δ 7.32 – 7.27 (m, 4H), 7.25 – 7.15 (m, 1H), 4.17 – 4.03 (m, 3H), 3.14 – 3.02 (m, 1H), 2.25 (s, 3H), 1.31 (d, *J* = 7.1 Hz, 3H). **^{13}CNMR** (101 MHz, MeOD) δ 211.0, 171.6, 144.7, 129.1, 128, 127.9, 127.7, 125.9, 77.6, 76.3, 60.2, 42.7, 26.6, 24.9, 19.5, 17.6, 13.1. [*α*]D = +90 (*c* = 0.18 in MeOH) **Optical Rotation** [*α*]D = +90 (*c* = 0.18 in MeOH) **UPLC-MS** (**QDA**) $[M+Na]^+$ seen 231.03

(3S,4R)-3,4-dihydroxy-6-phenylhexan-2-one (**4a**)

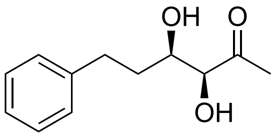

The amounts of reactants used were the following: 3-phenylpropanal (6mmol), hydroxyacetone (6 mmol), DMSO (12 mL) and 120mg of FSA WT lysate dissolved in 100 mM triethanolamine buffer pH 8 (47 mL). The crude product was purified by flash chromatography on silica gel (EtOAc/Cyclohexane from 1:4 to 1:0) to yield a clear oil (825mg, 66%).

^{1}H NMR (400 MHz, MeOD) δ 7.33 – 7.13 (m, 5H), 4.09 (d, J = 2.3 Hz, 1H), 4.01 – 3.91 (m, 1H), 2.89 – 2.65 (m, 2H), 2.21 (s, 3H), 1.98 – 1.80 (m, 2H). **^{13}C NMR** (101 MHz, MeOD) δ 210.8, 141.9, 128.1, 128.11, 125.6, 79.6, 71.2, 35.1, 31.8, 25.3. **Optical Rotation** [*α*]D = +34.5 (*c* = 0.48 in MeOH). **UPLC-MS** (**QDA**) $[M+Na]^+$ seen 231.03

(3S,4R)-6-(benzyloxy)-3,4-dihydroxyhexan-2-one (**5a**)

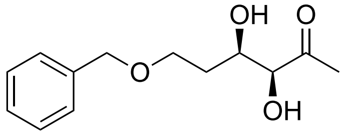

The amounts of reactants used were the following: 3-benzoxypropanal (6mmol), hydroxyacetone (6 mmol), DMSO (12 mL) and 120mg of FSA WT lysate dissolved in 100 mM triethanolamine buffer pH 8 (47 mL). The crude product was purified by flash chromatography on silica gel (EtOAc/Cyclohexane from 1:4 to 1:0) to yield a clear oil (851mg, 60%).

1H NMR (400 MHz, MeOD) δ 7.41 – 7.24 (m, 5H), 4.57 – 4.46 (m, 2H), 4.21 (td, J = 6.8, 2.3 Hz, 1H), 4.14 – 4.03 (m, 1H), 3.73 – 3.58 (m, 2H), 2.23 (s, 3H), 1.92 (q, J = 6.4 Hz, 2H). **^{13}C NMR** (101 MHz, MeOD) δ 210.6, 138.4, 128.2, 127.6, 127.4, 79.7, 72.7, 69.1, 66.9, 33.4, 26.7, 25.4 **Optical Rotation** [*α*]D = +38 (*c* = 0.43 in MeOH) **UPLC-MS** (**QDA**) $[M+Na]^+$ seen 261.05

(3S,4R)-3,4-dihydroxy-5-phenoxypentan-2-one (**6a**)

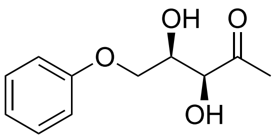

The amounts of reactants used were the following: phenoxyacetaldehyde (4 mmol), hydroxyacetone (4 mmol), DMSO (8 mL) and 120mg of FSA WT lysate dissolved in 100 mM triethanolamine buffer pH 8 (32 mL). The crude product was purified by flash chromatography on silica gel (EtOAc/Cyclohexane from 1:4 to 1:0) to yield a clear oil (235mg, 28%).

^{1}H NMR (400 MHz, MeOD) δ 7.33 – 7.22 (m, 2H), 7.00 – 6.89 (m, 3H), 4.41 – 4.30 (m, 2H), 4.19 – 3.98 (m, 2H), 2.29 (s, 3H). **^{13}C NMR** (101 MHz, MeOD) δ 210.5, 158.7, 129.2, 120.7, 114.3, 77.3, 70.2, 68.1, 25.4. **UPLC-MS (QDA)** [M+Na]$^+$ seen 232.98.

(3S,4R)-5-(benzyloxy)-1,3,4-trihydroxypentan-2-one (**1b**)

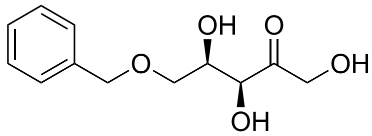

The amounts of reactants used were the following: 2-(benzyloxy)acetaldehyde (6 mmol), dihydroxyacetone (6 mmol), DMSO (12 mL) and 120mg of FSA A129S lysate dissolved in 100 mM triethanolamine buffer pH 8 (47 mL). The crude product was purified by flash chromatography on silica gel (EtOAc/Cyclohexane from 1:4 to 1:0) to yield a clear oil (302mg, 21%).

^{1}H NMR (400 MHz, MeOD) δ 7.42 – 7.24 (m, 5H), 4.65 – 4.42 (m, 4H), 4.38 – 4.30 (m, 1H), 4.17 (td, J = 6.4, 2.4 Hz, 1H), 3.70 – 3.52 (m, 2H).. **^{13}C NMR** (101 MHz, MeOD) δ 212.2, 138.2, 128.1, 127.6, 127.4, 75.8, 73, 70.7, 70.3, 66.6. **Optical Rotation** [*α*]D = +4.6 (*c* = 0.39 in $CHCl_3$). **UPLC-MS** (**QDA**) [M+Na]$^+$ seen 263.04

(3S,4R)-1,3,4-trihydroxy-5-phenylpentan-2-one (**2b**)

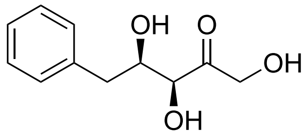

The amounts of reactants used were the following: phenylacetaldehyde (6 mmol), dihydroxyacetone (6 mmol), DMSO (12 mL) and 120mg of FSA WT lysate dissolved in 100 mM triethanolamine buffer pH 8 (47 mL). The crude product was purified by flash chromatography on silica gel (EtOAc/Cyclohexane from 1:4 to 1:0) to yield a clear oil (264mg, 21%).

^{1}H NMR (400 MHz, MeOD) δ 7.40 – 7.09 (m, 5H), 4.62 – 4.41 (m, 2H), 4.18 (td, J = 7.2, 2.1 Hz, 1H), 4.12 – 4.05 (m, 1H), 3.07 – 2.82 (m, 2H). **^{13}C NMR** (101 MHz, MeOD) δ 212.7, 138.4, 129.2, 128.1, 126, 76.9, 73.6, 66.5, 39.3 **Optical Rotation** [*α*]D = +12.3 (*c* = 0.39 in $CHCl_3$). **UPLC-MS** (**QDA**) [M+Na]$^+$ seen 233.02

(3S,4R)-1,3,4-trihydroxy-5-phenylhexan-2-one (**3b**)

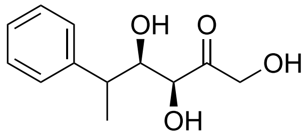

The amounts of reactants used were the following: 2-2-phenylpropanal (6 mmol), dihydroxyacetone (6 mmol), DMSO (12 mL) and 120mg of FSA A165G lysate dissolved in 100 mM triethanolamine buffer pH 8 (47 mL). The crude product was purified by flash chromatography on silica gel (EtOAc/Cyclohexane from 1:4 to 1:0) to yield a clear oil (228mg, 17%).

^{1}H NMR (400 MHz, MeOD) δ 7.36 – 7.11 (m, 5H), 4.57 – 4.45 (m, 3H), 4.12 – 4.03 (m, 1H), 3.15 – 3.03 (m, 1H), 1.29 (d, J = 7.1 Hz, 3H). **^{13}C NMR** (101 MHz, MeOD) δ 213.0, 144.6, 128.1, 127.8, 126.1, 76.6, 76.2, 66.5, 42.4, 17.8. **Optical Rotation** [*α*]D = +14 (*c* = 0.23 in $CHCl_3$). **UPLC-MS** (**QDA**) [M+Na]$^+$ seen 247.02

*(3*S*,4*R*)-1,3,4-trihydroxy-6-phenylhexan-2-one* (**4b**)

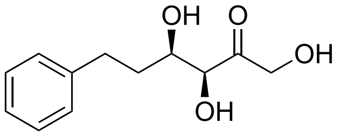

The amounts of reactants used were the following: 2-phenylpropanal (6 mmol), dihydroxyacetone (6 mmol), DMSO (12 mL) and 120mg of FSA A165G lysate dissolved in 100 mM triethanolamine buffer pH 8 (47 mL). The crude product was purified by flash chromatography on silica gel (EtOAc/Cyclohexane from 1:4 to 1:0) to yield a clear oil (484mg, 36%).

^{1}H NMR (400 MHz, MeOD) δ 7.35 – 7.07 (m, 5H), 4.67 – 4.41 (m, 2H), 4.21 (d, J = 2.4 Hz, 1H), 4.02 – 3.92 (m, 1H), 2.91 – 2.65 (m, 2H), 2.03 – 1.84 (m, 2H). **^{13}C NMR** (101 MHz, MeOD) δ 212.5, 141.9, 128.2, 128.2, 125.6, 78, 71.6, 66.6, 39.1, 34.9, 31.8. **Optical Rotation** [*α*]D = +5.76 (*c* = 0.59 in $CHCl_3$). **UPLC-MS** (**QDA**) $[M+Na]^+$ seen 247.02.

*(3*S*,4*R*)-6-(benzyloxy)-1,3,4-trihydroxyhexan-2-one* (**5b**)

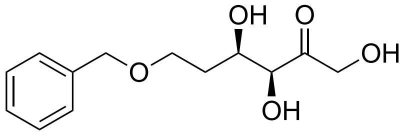

The amounts of reactants used were the following: 3-benzoxypropanal (6 mmol), dihydroxyacetone (6 mmol), DMSO (12 mL) and 120mg of FSA A129S lysate dissolved in 100 mM triethanolamine buffer pH 8 (47 mL). The crude product was purified by flash chromatography on silica gel (EtOAc/Cyclohexane from 1:4 to 1:0) to yield a clear oil (579mg, 38%).

^{1}H NMR (400 MHz, MeOD) δ 7.41 – 7.22 (m, 1H), 4.64 – 4.42 (m, 4H), 4.19 – 4.15 (m, 2H), 3.71 – 3.56 (m, 2H), 1.97 – 1.83 (m, 2H). **^{13}C NMR** (101 MHz, MeOD) δ 212.3, 138.5, 128.2, 127.6, 127.4, 78.2, 72.7, 69.5, 66.9, 66.9, 39.2, 33.1. **Optical Rotation** [*α*]D = +5.56 (*c* = 0.79 in $CHCl_3$). **UPLC-MS** (**QDA**) $[M+Na]^+$ seen 277.05.

*(3*S*,4*R*)-1,3,4-trihydroxy-5-phenoxypentan-2-one* (**6b**)

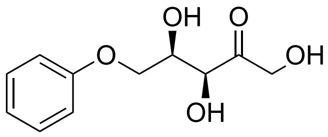

The amounts of reactants used were the following: phenoxyacetaldehyde (6 mmol), dihydroxyacetone (6 mmol), DMSO (12 mL) and 120mg of FSA A129S lysate dissolved in 100 mM triethanolamine buffer pH 8 (47 mL). The crude product was purified by flash chromatography on silica gel (EtOAc/Cyclohexane from 1:4 to 1:0) to yield a clear oil (651mg, 48%).

^{1}H NMR (400 MHz, MeOD) δ 7.37 – 7.18 (m, 2H), 7.03 – 6.87 (m, 3H), 4.70 – 4.53 (m, 2H), 4.49 – 4.42 (m, 1H), 4.39 – 4.30 (m, 1H), 4.20 – 3.95 (m, 2H). **^{13}C NMR** (101 MHz, MeOD) δ 212.1, 158.7, 129.2, 120.7, 114.3, 75.7, 70.4, 67.7, 66.6, 39.1. **Optical Rotation** [*α*]D = -3.44 (*c* = 0.58 in $CHCl_3$). **UPLC-MS** (**QDA**) $[M+Na]^+$ seen 249.00

(4S,5R)-6-(benzyloxy)-4,5-dihydroxyhexan-3-one (**1c**)

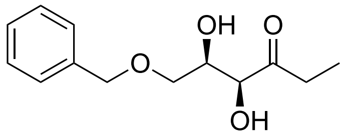

The amounts of reactants used were the following: 2-benzoxyacetaldehyde(6 mmol), hydroxybutanone (6 mmol), DMSO (12 mL) and 120mg of FSA WT lysate dissolved in 100 mM triethanolamine buffer pH 8 (47 mL). The crude product was purified by flash chromatography on silica gel (EtOAc/Cyclohexane from 1:4 to 1:0) to yield a clear oil (629mg, 44%).

^{1}H NMR (400 MHz, MeOD) δ 7.38 – 7.21 (m, 5H), 4.61 – 4.48 (m, 2H), 4.18 (d, *J* = 2.4 Hz, 1H), 4.12 (td, *J* = 6.3, 2.4 Hz, 1H), 3.69 – 3.42 (m, 2H), 2.74 – 2.47 (m, 3H), 1.02 (t, *J* = 7.3 Hz, 3H). **^{13}C NMR** (101 MHz, MeOD) δ 213.1, 138.2, 128, 127.5, 127.3, 77.0, 73.0, 70.7, 70.6, 31.6, 6.2. **Optical Rotation** [*α*]D = +10.98 (*c* = 0.728 in $CHCl_3$). **UPLC-MS** (**QDA**) $[M+Na]^+$ seen 261.05.

(4S,5R)-4,5-dihydroxy-6-phenylhexan-3-one (**2c**)

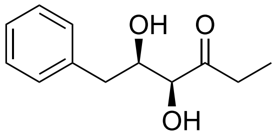

The amounts of reactants used were the following: phenylacetaldehyde (6 mmol), hydroxybutanone (6 mmol), DMSO (12 mL) and 120mg of FSA WT lysate dissolved in 100 mM triethanolamine buffer pH 8 (47 mL). The crude product was purified by flash chromatography on silica gel (EtOAc/Cyclohexane from 1:4 to 1:0) to yield a clear oil (312mg, 25%).

^{1}H NMR (400 MHz, MeOD) δ 7.31 – 7.07 (m, 5H), 4.14 (td, J = 7.2, 2.1 Hz, 1H), 3.92 (d, J = 2.0 Hz, 1H), 3.00 – 2.79 (m, 2H), 2.74 – 2.44 (m, 2H), 0.99 (dt, J = 9.7, 7.2 Hz, 3H). **^{13}C NMR** (101 MHz, MeOD) δ 213.43, 138.5, 129.3, 129.1, 128.0, 127.8, 126.0, 77.9, 73.6, 39.6, 31.4, 6.1. **Optical Rotation** [*α*]D = +15.5 (*c* = 0.36 in $CHCl_3$)**. UPLC-MS** (**QDA**) $[M+Na]^+$ seen 231.01.

(4S,5R)-4,5-dihydroxy-6-phenylheptan-3-one (**3c**)

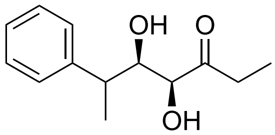

The amounts of reactants used were the following: 2-phenylpropanal (6 mmol), hydroxybutanone (6 mmol), DMSO (12 mL) and 120mg of FSA A165G lysate dissolved in 100 mM triethanolamine buffer pH 8 (47 mL). The crude product was purified by flash chromatography on silica gel (EtOAc/Cyclohexane from 1:4 to 1:0) to yield a clear oil (280mg, 21%).

^{1}H NMR (400 MHz, MeOD) δ 7.34 – 7.10 (m, 5H), 4.33 (d, J = 2.0 Hz, 1H), 4.02 (dd, J = 9.6, 2.0 Hz, 1H), 3.05 (dq, J = 9.6, 7.0 Hz, 1H), 2.72 – 2.53 (m, 2H), 1.28 (d, J = 7.1 Hz, 3H), 1.03 (t, J = 7.3 Hz, 3H). **^{13}C NMR** (101 MHz, MeOD) δ 213.5, 144.7, 127.9, 127.7, 125.9, 77.2, 76.5, 42.7, 31.2, 17.6, 6.2.**Optical Rotation** [*α*]D = +21.4 (*c* = 0.28 in $CHCl_3$)**. UPLC-MS** (**QDA**) $[M+Na]^+$ seen 245.04

(4S,5R)-4,5-dihydroxy-7-phenylheptan-3-one (**4c**)

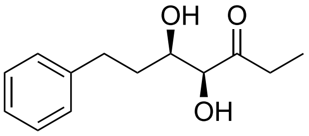

The amounts of reactants used were the following: 3-phenylpropanal (6 mmol), hydroxybutanone (6 mmol), DMSO (12 mL) and 120mg of FSA A165G lysate dissolved in 100 mM triethanolamine buffer pH 8 (47 mL). The crude product was purified by flash chromatography on silica gel (EtOAc/Cyclohexane from 1:4 to 1:0) to yield a clear oil (586 mg, 44%).

^{1}H NMR (400 MHz, MeOD) δ 7.29 – 7.10 (m, 5H), 4.13 – 4.02 (m, 1H), 3.91 (td, *J* = 6.8, 2.4 Hz, 1H), 2.85 – 2.72 (m, 1H), 2.69 – 2.49 (m, 3H), 1.94 – 1.80 (m, 2H), 1.02 (t, *J* = 7.3 Hz, 3H). **^{13}C NMR** (101 MHz, MeOD) δ 213.2, 141.9, 128.1, 128.0, 125.5, 79.1, 71.35, 35.1, 31.7, 31.5, 6.2. **Optical Rotation** [*α*]D = +22.5 (*c* = 0.88 in $CHCl_3$)**.** **UPLC-MS** (**QDA**) $[M+Na]^+$ seen 245.06

(4S,5R)-7-(benzyloxy)-4,5-dihydroxyheptan-3-one (**5c**)

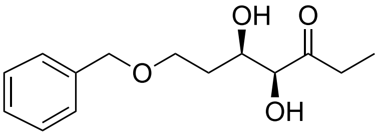

The amounts of reactants used were the following: 3-benzoxyacetaldehyde (6 mmol), hydroxybutanone (6 mmol), DMSO (12 mL) and 120mg of FSA WT lysate dissolved in 100 mM triethanolamine buffer pH 8 (47 mL). The crude product was purified by flash chromatography on silica gel (EtOAc/Cyclohexane from 1:4 to 1:0) to yield a clear oil (680mg, 45%).

^{1}H NMR (400 MHz, MeOD) δ 7.40 – 7.18 (m, 5H), 4.56 – 4.45 (m, 2H), 4.21 – 4.10 (m, 1H), 4.01 (d, J = 2.3 Hz, 1H), 3.70 – 3.55 (m, 2H), 2.73 – 2.46 (m, 3H), 1.94 – 1.80 (m, 2H), 1.03 (t, J = 7.3 Hz, 3H). **^{13}C NMR** (101 MHz, MeOD) δ 213.0, 138.4, 128.0, 127.5, 127.3, 79.2, 72.7, 69.3, 66.8, 33.3, 31.5, 6.2. **UPLC-MS** (**QDA**) $[M+Na]^+$ seen 275.04.

(4S,5R)-4,5-dihydroxy-6-phenoxyhexan-3-one (**6c**)

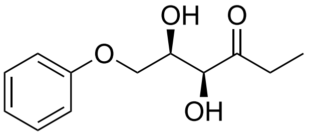

The amounts of reactants used were the following: phenoxyacetaldehyde (6 mmol), hydroxybutanone (6 mmol), DMSO (12 mL) and 120mg of FSA WT lysate dissolved in 100 mM triethanolamine buffer pH 8 (47 mL). The crude product was purified by flash chromatography on silica gel (EtOAc/Cyclohexane from 1:4 to 1:0) to yield a clear oil (752mg, 56%).

^{1}H NMR (400 MHz, MeOD) δ 7.30 – 7.21 (m, 2H), 6.97 – 6.87 (m, 3H), 4.35 – 4.26 (m, 2H), 4.16 – 3.93 (m, 2H), 2.84 – 2.61 (m, 2H), 1.06 (t, *J* = 7.3 Hz, 3H). **^{13}C NMR** (101 MHz, MeOD) δ 212.9, 158.8, 129.1, 120.6, 114.2, 76.8, 70.6, 68.1, 39.0, 31.6, 6.2. **UPLC-MS** (**QDA**) $[M+Na]^+$ seen 247.02.

Procedure & Characterization Data for Preparative biocatalytic reductive amination of amines with aldol products

To a 50 mL falcon tube, 20mM aldehyde, 20mM aldol donor (**a**, **b** or **c**), 2mg ml^{-1} FSA variant lysate and 20% vol/vol DMSO in TEA pH 8 buffer was added so that the total reaction volume was 25 mL. Reactions were incubated at 30ºC at 200rpm for 6 – 8hr. Reactions were then filtered at 4,000rpm for 10min using a 10,000 MWCO Vivaspin pre-washed with water. The flow through was then diluted up to a total reaction volume of 50 mL so that the components from part one were diluted by a factor of 2 and now also containing 200 or 500mM amine, 10mgml^{-1} IR-259, 1mM $NADP^+$, 0.25mg ml^{-1} CDX-801 GDH, 50mM glucose, 10% vol/vol DMSO and 100mM TEA pH 8 buffer. Reactions were incubated at 30ºC with shaking at 200rpm for 24h. The reactions were quenched with 50 mL MeOH and centrifuged at 4,000 rpm for 5 min and the supernatant was filtered through Celite®. The solution was concentrated by rotary evaporation and purified by reverse-phase flash column using C18 cartridge (H_2O:MeOH 95:5 to 20:80) and concentrated under reduced pressure to yield the desired amine product.

(2R,3R,4R)-1-(benzyloxy)-4-(cyclopropylamino)pentane-2,3-diol (**1ai**)

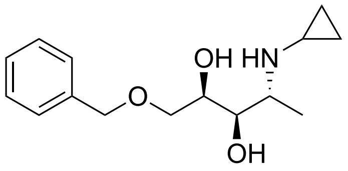

The title compound was obtained by the methodology described above for **1ai**. The amounts of reactants used were the following: 2-benzoxyacetaldehyde (0.5 mmol), hydroxyacetone (0.5 mmol), DMSO (5 mL) and 50mg of FSA WT lysate dissolved in 100 mM triethanolamine buffer pH 8 (20 mL). After filtration the supernatant was diluted to 50 mL with 200mM cyclopropylamine, 10mgml^{-1} IR-259, 1mM $NADP^+$, 0.25mg ml^{-1} CDX-801 GDH, 50mM glucose and 100mM TEA pH 8 buffer. The crude product was purified by reverse-phase flash column using C18 cartridge (H_2O:MeOH 95:5 to 20:80) to yield an orange/brown oil (31.2mg, 24%).

^{1}H NMR (400 MHz, MeOD) δ 7.22 – 6.87 (m, 5H), 3.72 – 3.61 (m, 1H), 3.29 – 3.14 (m, 1H), 3.08 (p, *J* = 1.6 Hz, 2H), 2.83 – 2.72 (m, 1H), 2.69 – 2.41 (m, 2H), 2.03 – 1.88 (m, 1H), 0.93 (d, *J* = 6.6 Hz, 2H), 0.38 – 0.01 (m, 3H). **^{13}C NMR** (101 MHz, MeOD) δ 138.3, 129.1, 128.0, 127.9, 127.5, 127.3, 73.0, 72.4, 71.6, 70.6, 55.4, 27.7, 14.5, 5.5, 4.6. **UPLC-MS** (**QDA**) $[M+H]^+$ seen 266.10. **Optical Rotation** [*α*]D = -21.3 (*c* = 0.03 in MeOH).

(2R,3R,4R)-5-(benzyloxy)-2-(cyclopropylamino)pentane-1,3,4-triol (**1bi**)

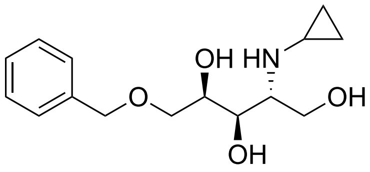

The title compound was obtained by the methodology described above for **1ai**. The amounts of reactants used were the following: 2-benzoxyacetaldehyde (0.5 mmol), dihydroxyacetone (0.5 mmol), DMSO (5 mL) and 50mg of FSA A129S lysate dissolved in 100 mM triethanolamine buffer pH 8 (20 mL). After filtration the supernatant was diluted to 50 mL with 200mM cyclopropylamine, 10mgml^{-1} IR-259, 1mM $NADP^+$, 0.25mg ml^{-1} CDX-801 GDH, 50mM glucose and 100mM TEA pH 8 buffer. The crude product was purified by reverse-phase flash column using C18 cartridge (H_2O:MeOH 95:5 to 20:80) to yield an amorphous brown solid was then recrystallized in MeOH to form pale brown single crystals (22.8mg, 16%).

^{1}H NMR (400 MHz, MeOD) δ 7.46 – 7.17 (m, 5H), 4.57 (s, 2H), 4.00 – 3.91 (m, 1H), 3.87 – 3.71 (m, 3H), 3.68 – 3.52 (m, 2H), 2.92 – 2.83 (m, 1H), 2.32 – 2.21 (m, 1H), 0.56 – 0.30 (m, 4H). **^{13}C NMR** (101 MHz, MeOD) δ 138.3, 128.0, 127.5, 127.3, 73.0, 71.8, 70.3, 70.2, 60.9, 60.0, 28.1, 5.7, 5.2.**UPLC-MS** (**QDA**) $[M+H]^+$ seen 282.10. **Optical Rotation** [*α*]D = -9.3 (*c* = 0.03 in MeOH).

(2R,3R,4R)-1-(benzyloxy)-4-(cyclopropylamino)hexane-2,3-diol (**1ci**)

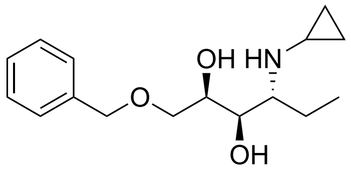

The title compound was obtained by the methodology described above for **1ai**. The amounts of reactants used were the following: 2-benzoxyacetaldehyde (0.5 mmol), hydroxybutanone (0.5 mmol), DMSO (5 mL) and 50mg of FSA WT lysate dissolved in 100 mM triethanolamine buffer pH 8 (20 mL). After filtration the supernatant was diluted to 50 mL with 200mM cyclopropylamine, 10mgml^{-1} IR-259, 1mM NADP$^+$, 0.25mg ml^{-1} CDX-801 GDH, 50mM glucose and 100mM TEA pH 8 buffer. The crude product was purified by reverse-phase flash column using C18 cartridge (H_2O:MeOH 95:5 to 20:80) to yield a brown oil (139mg, 30%).

^{1}H NMR (400 MHz, MeOD) δ 7.46 – 7.13 (m, 5H), 4.62 – 4.50 (m, 2H), 3.99 – 3.89 (m, 1H), 3.72 – 3.64 (m, 1H), 3.63 – 3.50 (m, 2H), 2.79 (q, J = 6.0 Hz, 1H), 2.28 – 2.13 (m, 1H), 1.70 – 1.55 (m, 2H), 0.96 (t, J = 7.5 Hz, 3H), 0.55 – 0.29 (m, 4H)**.** **^{13}C NMR** (101 MHz, MeOD) δ 138.3, 127.9, 127.5, 127.3, 73.0, 71.7, 70.5, 70.1, 61.8, 28.4, 22.5, 9.3, 5.9, 5.1. **UPLC-MS** (**QDA**) [M+H]$^+$ seen 280.12. **Optical Rotation** [*α*]D = -22 (*c* = 0.03 in MeOH).

(2R,3R,4R)-4-(cyclopropylamino)-1-phenylpentane-2,3-diol (**2ai**)

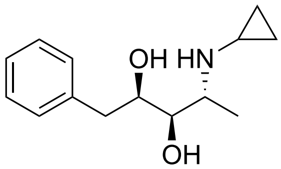

The title compound was obtained by the methodology described above for **1ai**. The amounts of reactants used were the following: 2-phenylacetaldehyde (0.5 mmol), hydroxyacetone (0.5 mmol), DMSO (5 mL) and 50mg of FSA WT lysate dissolved in 100 mM triethanolamine buffer pH 8 (20 mL). After filtration the supernatant was diluted to 50 mL with 200mM cyclopropylamine, 10mgml^{-1} IR-259, 1mM NADP$^+$, 0.25mg ml^{-1} CDX-801 GDH, 50mM glucose and 100mM TEA pH 8 buffer. The crude product was purified by reverse-phase flash column using C18 cartridge (H_2O:MeOH 95:5 to 20:80) to yield a brown oil (117.6mg, 12%).

^{1}H NMR (400 MHz, MeOD) δ 7.15 – 6.89 (m, 5H), 3.72 – 3.59 (m, 1H), 3.24 – 3.10 (m, 1H), 3.10 – 3.06 (m, 2H), 2.70 – 2.49 (m, 2H), 2.04 – 1.88 (m, 1H), 0.93 (d, J = 6.6 Hz, 2H), 0.33 – 0.05 (m, 3H). **^{13}C NMR** (101 MHz, MeOD) δ 139.0, 129.2, 127.9, 125.7, 73.4, 72.9, 55.7, 39.5, 39.0, 27.7, 14.6, 5.7, 4.5. **UPLC-MS** (**QDA**) [M+H]$^+$ seen 236.10. **Optical Rotation** [*α*]D = -3.3 (*c* = 0.03 in MeOH).

(2R,3R,4R)-2-(cyclopropylamino)-5-phenylpentane-1,3,4-triol (**2bi**)

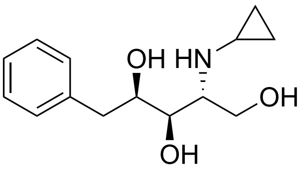

The title compound was obtained by the methodology described above for **1ai**. The amounts of reactants used were the following: 2-phenylacetaldehyde (0.5 mmol), dihydroxyacetone (0.5 mmol), DMSO (5 mL) and 50mg of FSA WT lysate dissolved in 100 mM triethanolamine buffer pH 8 (20 mL). After filtration the supernatant was diluted to 50 mL with 200mM cyclopropylamine, 10mgml^{-1} IR-259, 1mM NADP$^+$, 0.25mg ml^{-1} CDX-801 GDH, 50mM glucose and 100mM TEA pH 8 buffer. The crude product was purified by reverse-phase flash column using C18 cartridge (H_2O:MeOH 95:5 to 20:80) to yield a brown oil (125.6mg, 26%)

^{1}H NMR (400 MHz, MeOD) δ 7.35 – 7.13 (m, 5H), 4.04 – 3.93 (m, 1H), 3.79 (app. qd, J = 11.1, 4.9 Hz, 2H), 3.59 (dd, J = 6.6, 2.7 Hz, 1H), 3.01 – 2.76 (m, 2H), 2.29 – 2.20 (m, 1H), 0.52 – 0.29 (m, 4H). **^{13}C NMR** (101 MHz, MeOD) δ 139.2, 129.2, 127.9, 125.7, 73.1, 71.1, 61.1, 60.0, 39.6, 28.0, 5.6, 5.2. **UPLC-MS** (**QDA**) [M+H]$^+$ seen 252.09. **Optical Rotation** [*α*]D = -5.3 (*c* = 0.03 in MeOH).

(2R,3R,4R)-4-(cyclopropylamino)-1-phenylhexane-2,3-diol (**2ci**)

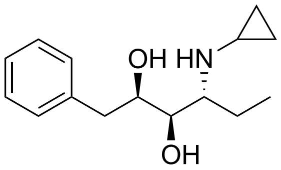

The title compound was obtained by the methodology described above for **1ai**. The amounts of reactants used were the following: 2-phenylacetaldehyde (0.5 mmol), hydroxybutanone (0.5 mmol), DMSO (5 mL) and 50mg of FSA WT lysate dissolved in 100 mM triethanolamine buffer pH 8 (20 mL). After filtration the supernatant was diluted to 50 mL with 200mM cyclopropylamine, 10mgml^{-1} IR-259, 1mM NADP^{+}, 0.25mg ml^{-1} CDX-801 GDH, 50mM glucose and 100mM TEA pH 8 buffer. The crude product was purified by reverse-phase flash column using C18 cartridge (H_2O:MeOH 95:5 to 20:80) to yield an orange/brown oil (17mg, 14%). **Optical Rotation** [*α*]D =--10 (*c* = 0.03 in MeOH).

^{1}H NMR (400 MHz, MeOD) δ 7.07 – 6.88 (m, 5H), 3.75 – 3.65 (m, 1H), 2.70 – 2.61 (m, 1H), 2.60 – 2.52 (m, 2H), 2.51 – 2.29 (m, 1H), 1.49 – 1.24 (m, 2H), 0.62 (t, J = 7.5 Hz, 3H), 0.52 – 0.40 (m, 1H), 0.27 – 0.03 (m, 4H). **^{13}C NMR** (101 MHz, MeOD) δ 139.0, 129.1, 127.9, 125.7, 72.6, 70.7, 62.2, 39.6, 28.4, 22.6, 9.1, 5.9, 5, 4.9. **UPLC-MS** (**QDA**) [M+H]$^+$ seen 250.11.

Characterization Data for chemically synthesized standards

Compounds used as substrates in biocatalytic cascade

2-phenoxyacetaldehyde(**6**)

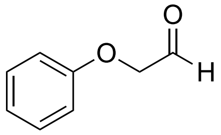

^{1}H NMR (400 MHz, MeOD) δ 7.32 – 7.17 (m, 2H), 6.94 (m, 3H), 2.63 (s, 2H). **^{13}C NMR** (101 MHz, MeOD) δ 158.7, 129.2, 120.7, 114.4, 114.3, 114.3, 114.3, 39.1 **UPLC-MS** (**QDA**) seen 190.99.

Side products of the two-step biocatalytic cascade

N-(2-benzyloxy)ethyl)cyclopropanamine (**7**)

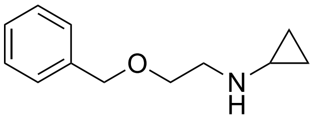

Amount of reactants are as follows: 2-benzoxyaldehyde (2 mmol) and cyclopropylamine (2.5 mmol) followed by the general procedure to yield title compound as a brown oil (64 mg, 17% yield).
^{1}H NMR (400 MHz, DMSO) δ 7.17 – 7.04 (m, 5H), 4.27 (s, 2H), 3.29 (t, J = 5.8 Hz, 2H), 2.56 (t, J = 5.8 Hz, 2H), 1.93 – 1.77 (m, 1H), 0.19 – -0.08 (m, 4H). **^{13}CNMR** (101 MHz, DMSO) δ 139.1, 128.7, 128.0, 127.8, 72.4, 69.8, 48.9, 30.5, 26.8, 6.6. **UPLC-MS** (**QDA**) [M+H]$^+$ seen 192.06.

N-phenethylcyclopropanamine (**8**)

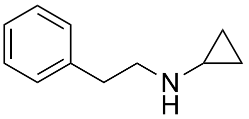

Amount of reactants are as follows: phenylacetaldehyde (2 mmol) and cyclopropylamine (2.5 mmol) followed by the general procedure to yield title compound as a brown oil (116 mg, 36% yield) with minor impurities (contaminated with starting material).
^{1}H NMR (400 MHz, MeOD) δ 7.17 – 6.78 (m, 5H), 2.71 – 2.50 (m, 4H), 1.95 – 1.85 (m, 1H), 0.26 – 0.03 (m, 3H). **^{13}C NMR** (101 MHz, MeOD) δ 139.8, 128.3, 128.2, 128.1, 125.9, 50.6, 35.4, 29.8, 4.8.

N-(2-phenylpropyl)cyclopropanamine (**9**)

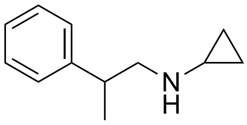

Amount of reactants are as follows: 2-phenylpropanal (2 mmol) and cyclopropylamine (2.5 mmol) followed by the general procedure to yield title compound as a yellow liquid (134 mg, 38% yield).
^{1}H NMR (400 MHz, MeOD) δ 7.14 – 6.93 (m, 5H), 2.84 – 2.56 (m, 3H), 1.88 (m, 1H), 1.09 – 0.95 (m, 3H), 0.28 – 0.03 (m, 4H). **^{13}C NMR** (101 MHz, MeOD) δ 145.1, 128.4, 126.6, 126.2, 56.1, 39.3, 29.8, 19.4, 4.9, 4.9.

N-(3-phenylpropyl)cyclopropanamine (**10**)

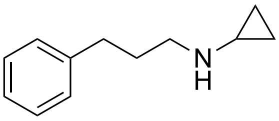

Amount of reactants are as follows: 3-phenylpropanal (2 mmol) and cyclopropylamine (2.5 mmol) followed by the general procedure to yield title compound as a yellow liquid (160 mg, 45% yield).
^{1}H NMR ^{1}H NMR (400 MHz, MeOD) δ 7.16 – 6.96 (m, 5H), 2.59 – 2.36 (m, 4H), 2.00 – 1.90 (m, 1H), 1.74 – 1.59 (m, 2H), 0.35 – 0.15 (m, 3H).**^{13}C NMR** (101 MHz, MeOD) δ 142.0, 128.1, 128.0, 125.5, 125.5, 48.7, 33.4, 31.0, 29.9, 4.7.

N-(3-(benzyloxy)propyl)cyclopropanamine (**11**)

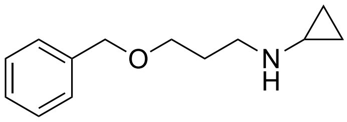

Amount of reactants are as follows: 3-(benzyloxy)propanal (2 mmol) and cyclopropylamine (2.5 mmol) followed by the general procedure to yield title compound as a yellow liquid (218 mg, 53% yield).
^{1}H NMR (400 MHz, MeOD) δ 7.23 – 7.07 (m, 5H), 4.34 (s, 2H), 3.40 (t, J = 6.2 Hz, 2H), 2.65 – 2.50 (m, 2H), 1.97 (tt, J = 6.8, 3.6 Hz, 1H), 1.74 – 1.60 (m, 2H), 0.35 – 0.15 (m, 4H). **13CNMR** (101 MHz, MeOD) δ 138.5, 128.1, 128.0, 127.4, 127.4, 127.3, 127.3, 72.6, 68.6, 29.9, 29.1, 4.7.

N-(2-phenoxyethyl)cyclopropanamine (**12**)

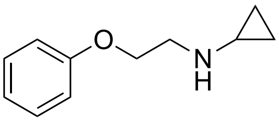

Amount of reactants are as follows: 2-phenoxyacetaldehyde (2 mmol) and cyclopropylamine (2.5 mmol) followed by the general procedure to yield title compound as a yellow liquid (241 mg, 68% yield).
^{1}H NMR (400 MHz, MeOD) δ 7.13 – 7.02 (m, 2H), 6.79 – 6.68 (m, 3H), 3.89 – 3.83 (m, 2H), 2.83 (t, J = 5.4 Hz, 2H), 2.06 – 1.92 (m, 1H), 1.04 (t, J = 7.1 Hz, 1H), 0.34 – 0.14 (m, 4H). **^{13}C NMR** (101 MHz, MeOD) δ 158.9, 129.2, 120.6, 114.2, 66.3, 29.7, 4.9.

Product standards for two-step biocatalytic cascade

(3R,4R)-1-(benzyloxy)-4-(cyclopropylamino)pentane-2,3-diol (**1ai**)

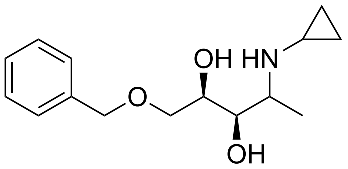

The title compound was obtained as a mixture of diastereomers by the methodology described above. Amount of reactants are as follows: Aldol product **1a** (1 mmol) and cyclopropylamine (1.5 mmol) followed by the general procedure to yield title compound as a brown solid (140mg, 53% yield).^{1}**H NMR** (400 MHz, MeOD) δ 7.39 – 7.32 (m, 5H), 4.64 – 4.50 (m, 3H), 3.93 – 3.82 (m, 1H), 3.66 – 3.48 (m, 3H), 3.03 – 2.90 (m, 1H), 2.24 – 2.13 (m, 1H), 1.25 – 1.11 (m, 4H), 0.64 – 0.27 (m, 4H). ^{13}**C NMR** (101 MHz, MeOD) δ 138.3, 128.0, 127.5, 127.3, 73.0, 71.8, 70.9, 56.2, 27.7, 15.3, 6.2, 4.2. **Optical Rotation** [*α*]D = +5.78 (*c* = 0.19 in MeOH).

(3R,4R)-5-(benzyloxy)-2-(cyclopropylamino)pentane-1,3,4-triol (**1bi**)

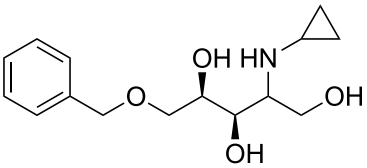

The title compound was obtained as a mixture of diastereomers by the methodology described above. Amount of reactants are as follows: Aldol product **1b** (1 mmol) and cyclopropylamine (1.5 mmol) followed by the general procedure to yield title compound as a brown solid (109mg, 28% yield).^{1}**H NMR** (400 MHz, MeOD) δ 7.40 – 7.25 (m, 5H), 4.57 (s, 2H), 4.05 – 3.90 (m, 1H), 3.84 – 3.42 (m, 2H), 3.38 – 3.30 (m, 1H), 2.99 – 2.78 (m, 1H), 2.38 – 2.20 (m, 1H), 0.54 – 0.39 (m, 4H). **Optical Rotation** [*α*]D = +4.97 (*c* = 0.19 in MeOH).

(3R,4R)-1-(benzyloxy)-4-(cyclopropylamino)hexane-2,3-diol (**1ci**)

The title compound was obtained as a mixture of diastereomers by the methodology described above. Amount of reactants are as follows: Aldol product **1c** (1 mmol) and cyclopropylamine (1.5 mmol) followed by the general procedure to yield title compound as a brown solid (50mg, 18% yield).

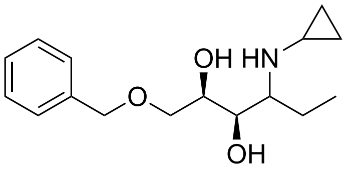

^{1}**H NMR** (400 MHz, MeOD) δ 7.46 – 7.12 (m, 5H), 3.87 (s, 1H), 3.67 (dd, *J* = 11.2, 4.7 Hz, 1H), 3.52 (dtd, *J* = 15.7, 12.3, 4.5 Hz, 2H), 2.90 – 2.26 (m, 2H), 1.87 – 1.51 (m, 2H), 1.00 (hept, *J* = 6.3 Hz, 4H), 0.56 (d, *J* = 19.4 Hz, 4H). ^{13}**C NMR** (101 MHz, MeOD) δ 138.3, 128.0, 127.5, 127.5, 127.3, 96.2, 79.4, 73, 71.9, 69.6, 65.4, 30.2, 28.3, 24.0, 22.7, 9.0, 5.3, 4.0. **Optical Rotation** [*α*]D = -3.72 (*c* = 0.1 in MeOH).

(3R,4R)-4-(cyclopropylamino)-1-phenylpentane-2,3-diol (**2ai**)

Amount of reactants are as follows: Aldol product **2a** (1 mmol) and cyclopropylamine (1.5 mmol) followed by the general procedure to yield title compound as a brown solid (48mg, 20% yield) with minor impurities.

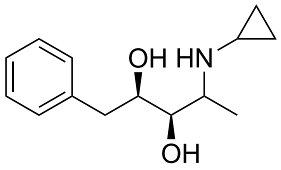

^{1}H NMR (400 MHz, MeOD) δ 7.38 – 7.09 (m, 5H), 3.90 (t, *J* = 7.1 Hz, 1H), 3.45 – 3.30 (m, 1H), 3.14 (dd, *J* = 6.0, 1.4 Hz, 1H), 2.98 – 2.65 (m, 3H), 2.21 – 2.01 (m, 1H), 1.14 (t, *J* = 6.7 Hz, 2H), 0.59 – 0.26 (m, 4H).**^{13}C NMR** (101 MHz, MeOD) δ 137.7, 127.9, 127.9, 126.8, 126.7, 124.6, 124.6, 94.5, 81.8, 57.8, 55.7, 39.1, 27.6, 20.9, 14.3, 3.9, 1.4. **Optical Rotation** [*α*]D = -3.05 (*c* = 0.09 in MeOH).

(3R,4R)-2-(cyclopropylamino)-5-phenylpentane-1,3,4-triol (**2bi**)

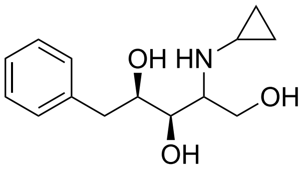

Amount of reactants are as follows: Aldol product **2b** (1 mmol) and cyclopropylamine (1.5 mmol) followed by the general procedure to yield title compound as a brown solid (40 mg, 16% yield).

^{1}H NMR (400 MHz, MeOD) δ 7.07 – 6.91 (m, 5H), 3.78 – 3.67 (m, 1H), 3.65 – 3.45 (m, 2H), 3.45 – 3.29 (m, 1H), 2.76 – 2.50 (m, 3H), 2.13 – 1.96 (m, 1H), 0.39 – -0.03 (m, 3H). **^{13}C NMR** (101 MHz, MeOD) δ 139.2, 138.8, 129.2, 127.9, 127.9, 125.7, 125.7, 74.9, 73.1, 71.1, 69.6, 62.6, 61.1, 60.1, 59.9, 39.9, 39.7, 28.0, 6.2, 5.6, 5.3, 4.8. **Optical Rotation** [*α*]D = +10.2 (*c* = 0.08 in MeOH).

(3R,4R)-4-(cyclopropylamino)-1-phenylhexane-2,3-diol (**2ci**)

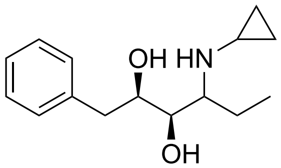

Amount of reactants are as follows: Aldol product **2c** (1 mmol) and cyclopropylamine (1.5 mmol) followed by the general procedure to yield title compound as a brown solid (44 mg, 14% yield). **Optical Rotation** [*α*]D = -8.94 (*c* = 0.08 in MeOH).

^{1}H NMR (400 MHz, MeOD) δ 7.08 – 6.88 (m, 5H), 3.77 – 3.59 (m, 1H), 3.16 – 3.06 (m, 1H), 2.79 – 2.39 (m, 2H), 2.09 – 1.98 (m, 1H), 1.52 – 1.28 (m, 1H), 0.78 – 0.54 (m, 2H), 0.35 – 0.06 (m, 3H). **^{13}C NMR** (101 MHz, MeOD) δ 138.7, 129.2, 129.1, 127.9, 127.8, 125.8, 75.6, 69.1, 63.0, 40.0, 27.7, 22.9, 9.3, 6.3, 4.6.

Determination of absolute stereochemistry of biocatalytically prepared 1bi by X-Ray Crystallography

Crystal growth of $C_{15}H_{23}NO_4$: Grayson Ford (Flitsch Group)
Data collected: Dr. George Whitehead Experimental Officer, Faculty of Natural Sciences XRD facility, University of Manchester.
Report Prepared: Dr. George Whitehead Experimental Officer, Faculty of Natural Sciences XRD facility, University of Manchester.

All chiral centers determined to be (*R*).

CCDC Deposition number: 2154768

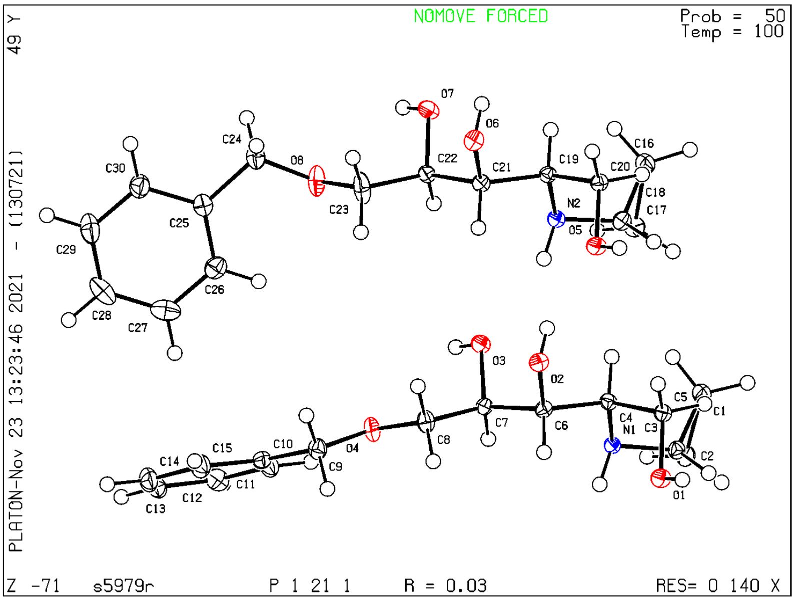

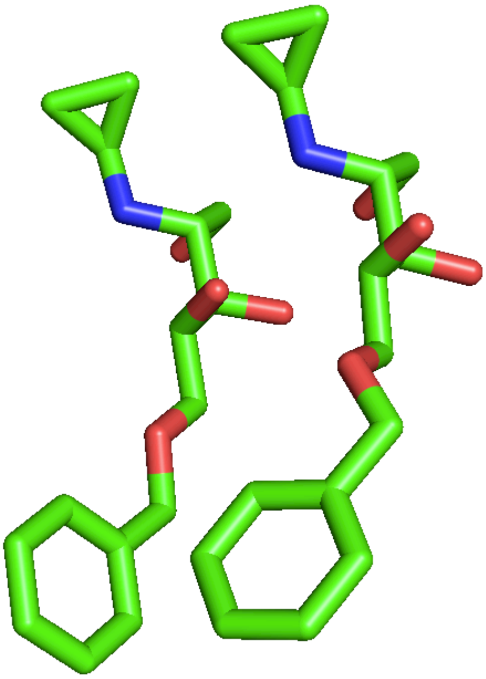

No syntax errors found.   CIF dictionary   Interpreting this report

Datablock: s5979r

```
Bond precision:   C-C = 0.0024 A          Wavelength=1.54184

Cell:             a=6.14824(8)    b=27.5380(3)      c=8.60576(10)
                  alpha=90        beta=97.7254(12)  gamma=90
Temperature:      100 K

                  Calculated                 Reported
Volume            1443.82(3)                 1443.82(3)
Space group       P 21                       P 1 21 1
Hall group        P 2yb                      P 2yb
Moiety formula    C15 H23 N O4               C15 H23 N O4
Sum formula       C15 H23 N O4               C15 H23 N O4
Mr                281.34                     281.34
Dx,g cm-3         1.294                      1.294
Z                 4                          4
Mu (mm-1)         0.763                      0.763
F000              608.0                      608.0
F000'             609.91
h,k,lmax          7,34,10                    7,34,10
Nref              6008[ 3068]                5813
Tmin,Tmax         0.950,0.974                0.883,1.000
Tmin'             0.874

Correction method= # Reported T Limits: Tmin=0.883 Tmax=1.000
AbsCorr = MULTI-SCAN

Data completeness= 1.89/0.97        Theta(max)= 75.664

R(reflections)= 0.0252( 5603)                  wR2(reflections)=
                                               0.0673( 5813)
S = 1.039                  Npar= 393
```

Docking Studies

Docking was performed using VINA default parameters in the YASARA molecular modelling software program. The *Asp*RedAm crystal structure (5G6R) was used to generate a homology model of IR-259 using SWISS-MODEL with model building paramters set to automated mode. The IR-259 model was then used as a receptor for simulations. The model was prepared for simulations using the YASARA ‘clean’ script. To find the correct geometry of the receptor in aqueous environment, a unit cell was defined around the whole model which was filled with water molecules and then the structure was energy-minimizedl by running the ‘Energy Minimization’ script. Product 2ai was considered as the ligand in the simulation experiment using their terminal amine functional group in the unprotonated form. For the docking simulation, a 7 Å simulation cell around the NADPH co-factor was defined for which reasonable functional binding poses were calculated. From the 20 calculated binding poses for each docking run, the most catalytically reasonable, with the highest docking score (strongest binding) was selected based on the requirement for the C-N functional group to be position close to the C4 of cofactor NADPH. The ligand-receptor complex structure was further processed using PyMOL software, including identification of hydrophobic residues responsible for binding the phenyl ring as well as polar residues can aid in substrate binding as well as catalysis.

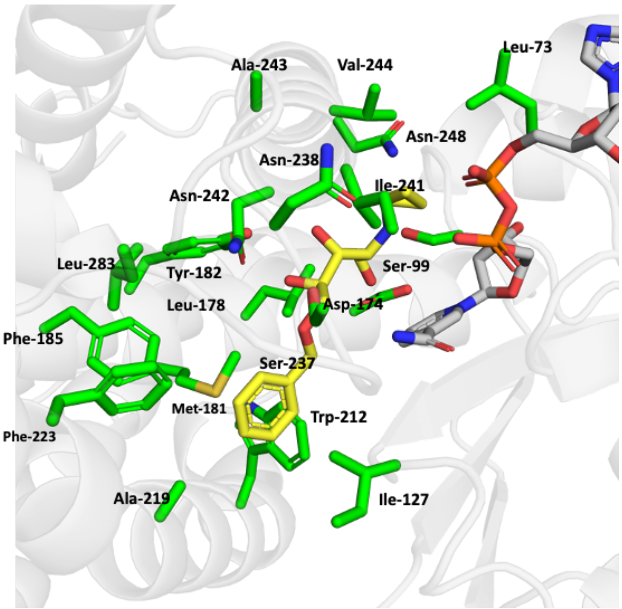

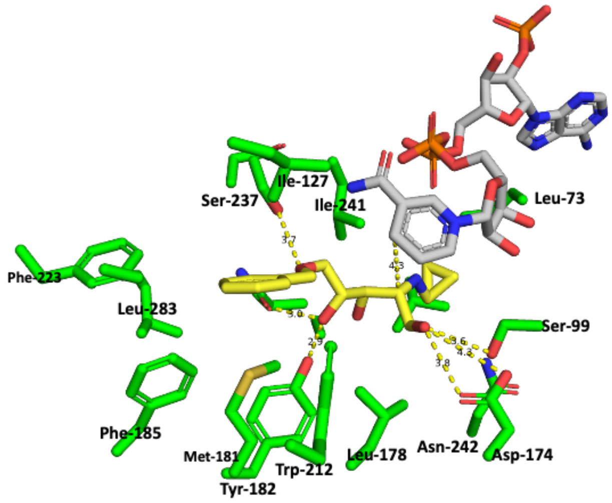

Figure S5: Molecular docking (AutoDock VINA as implemented in YASARA) of **1bi** (in yellow) in IR-259 homology model (in green) based on the *Asp*RedAm crystal structure (5G6R) with NADPH co-crystallized (in grey). Substrate interacting residues are shown and distances indicating possible interactions between the substrate and enzyme are highlighted (yellow-dashed lines) with distances in Angstroms.

Determination of diastereomeric ratio of preparative scale reactions

Following the determination of absolute stereochemistry of product **2ai**, it was assumed that the IRED mediated reaction would follow the same mechanism for the other substrates, resulting in (2*R*,3*R*,4*R*) stereocenters for all products.

Chemical standards synthesized for the preparative scale reaction could form a range of diastereomers. Inspecting the UPLC traces for the chemical preparation, multiple product peaks are shown, suggesting multiple diastereomers have formed, compared to the crude biocatalytic routes which one resulted in one product peak.

Diastereomeric ratios (dr) determined from chemical routes below and was determined by UPLC-QDA.

Substrate	Product	N° of product peaks observed (UPLC)	Diastereomeric ratio
1a	1ai	3	90:7:3
2a	2ai	3	90:2:8
1b	1bi	2	53:47
2b	2bi	2	51:49
1c	1ci	2	99:1
2c	2ci	3	87:10:3

Possible chemical route:

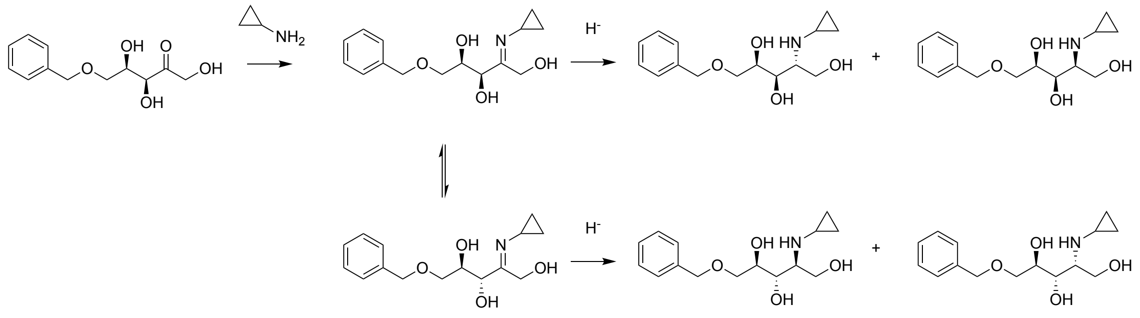

Biocatalytic route:

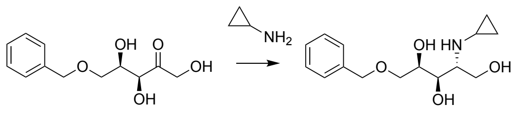

Figure S6: Possible chemical route and biocatalytic route towards product **1bi** from **1b**.

Figure S7: UPLC chromatogram traces with accompanying QDA-MS trace for chemical and biocatalytic (crude) reactions for reactions, **1a** to **1ai**, **1b** to **1bi**, **1c** to **1ci**, **2a** to **2ai**, **2b** to **2bi** and **2c** to **2ci**. Chemical routes showed two peaks indicating the formation of two possible diastereomers and biocatalytic only showed one indicating formation of only one diastereomer.

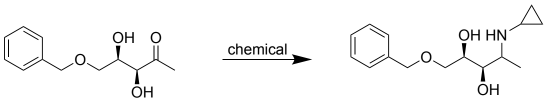

1a **1ai**

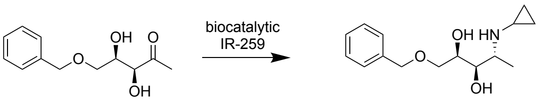

1a **1ai**

Chemical – 3 peaks with product mass

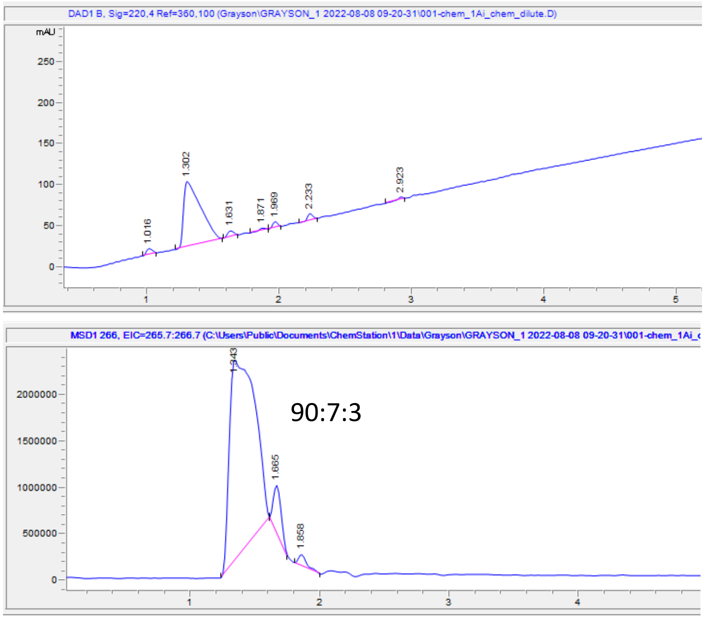

Biocatalytic – 1 peak with product mass

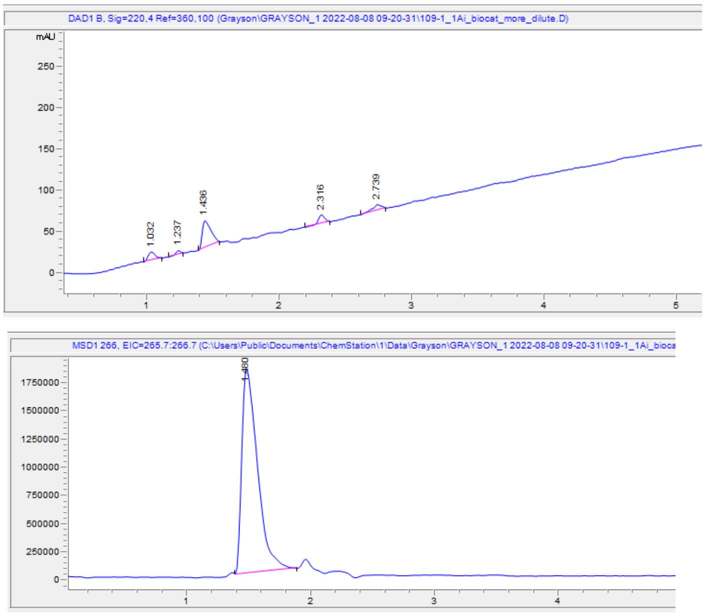

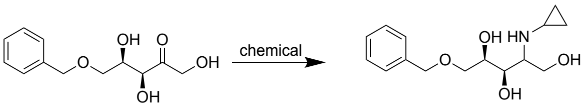

1b **1bi**

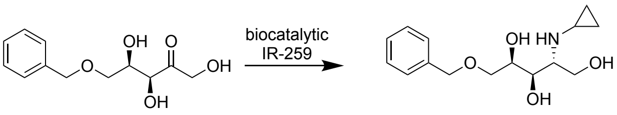

1b **1bi**

Chemical – 2 peaks with product mass

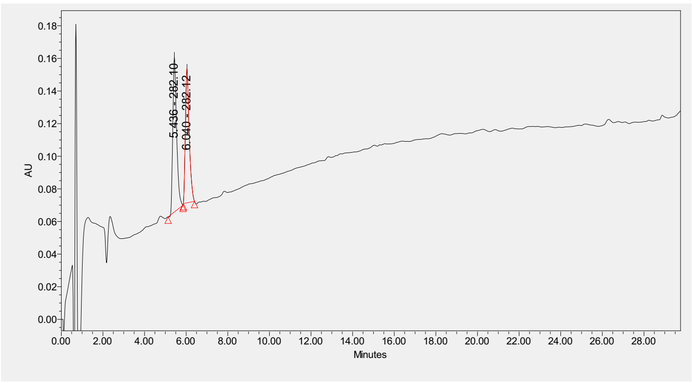

Biocatalytic – 1 peak with product mass

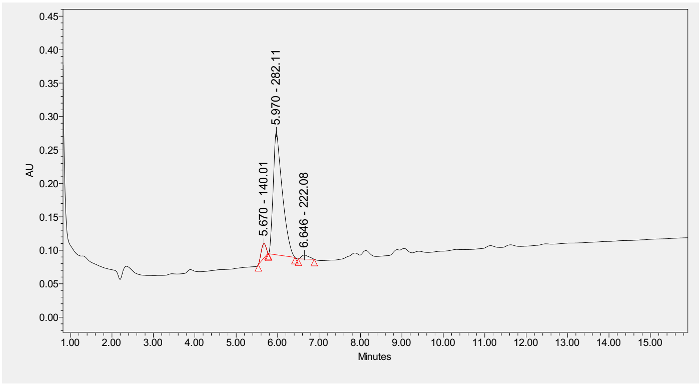

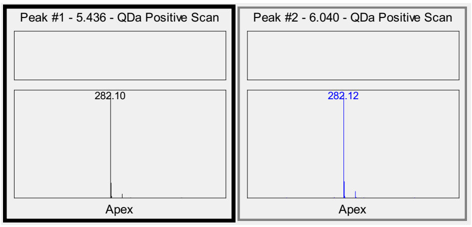

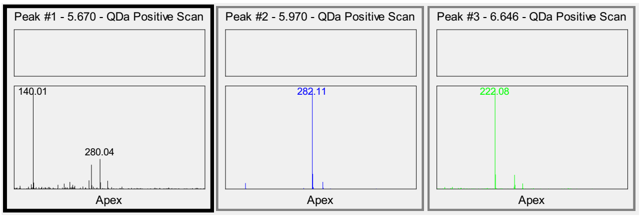

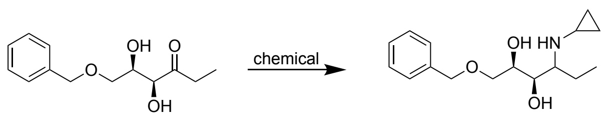

1c 1ci

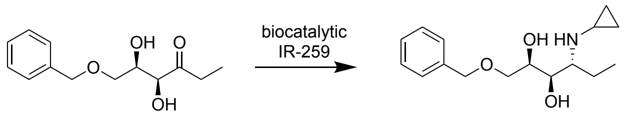

1c 1ci

Chemical – 1 peak1 with product mass

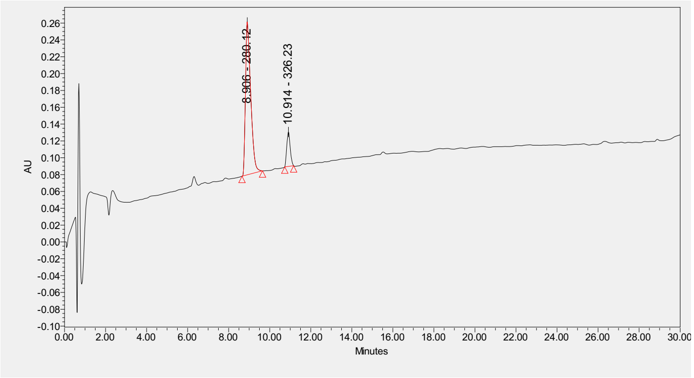

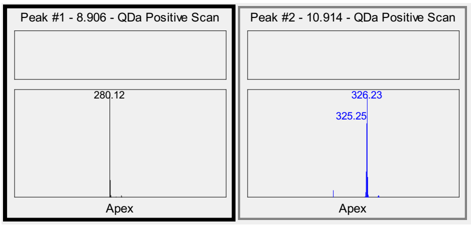

Chemical – 1 peak1 with product mass

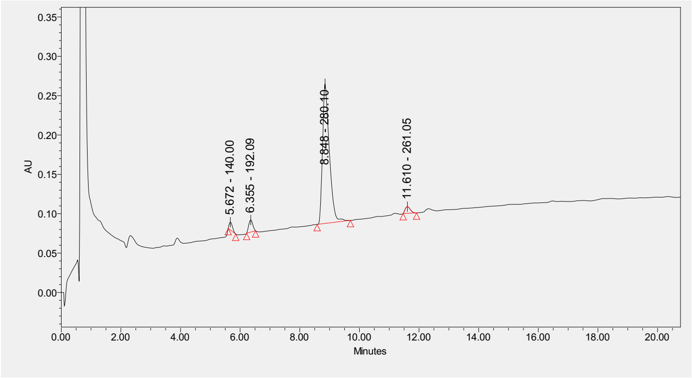

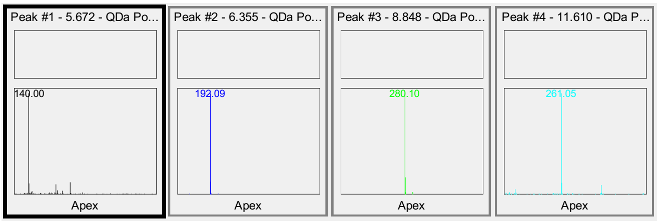

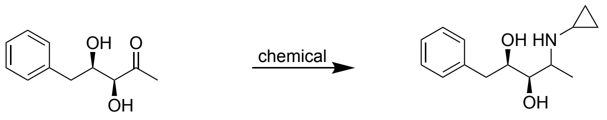

2a 2ai

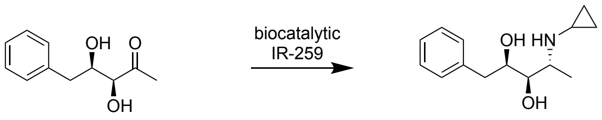

2a 2ai

Chemical – 3 peaks with product mass

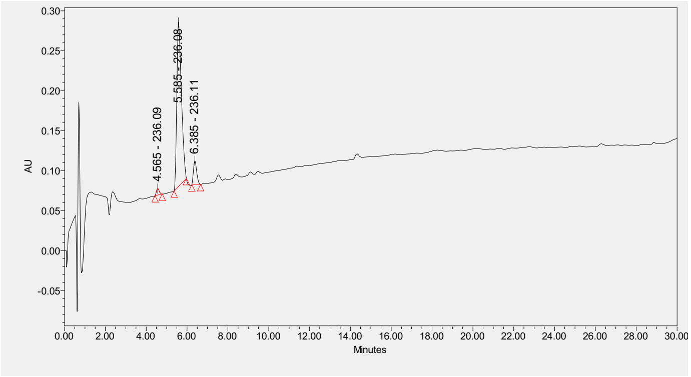

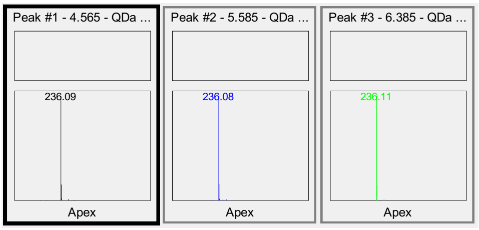

Biocatalytic – 1 peak with product mass

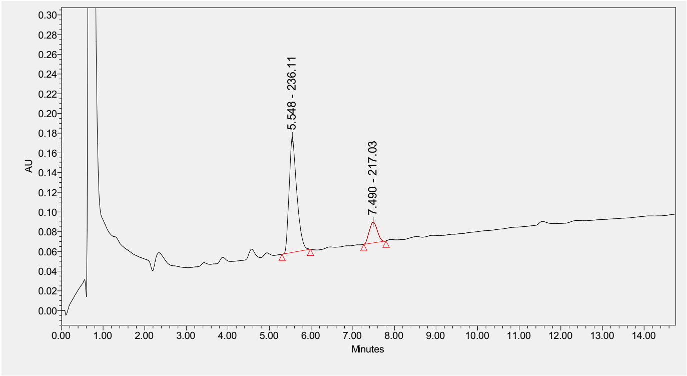

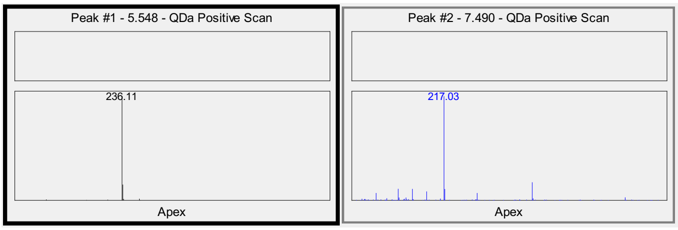

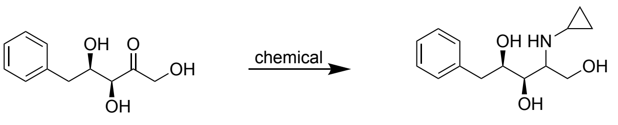

2b 2bi

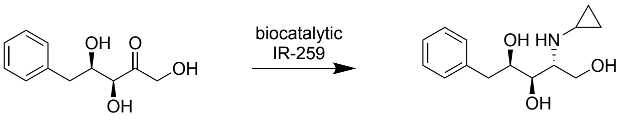

2b 2bi

Chemical – 2 peaks with product mass

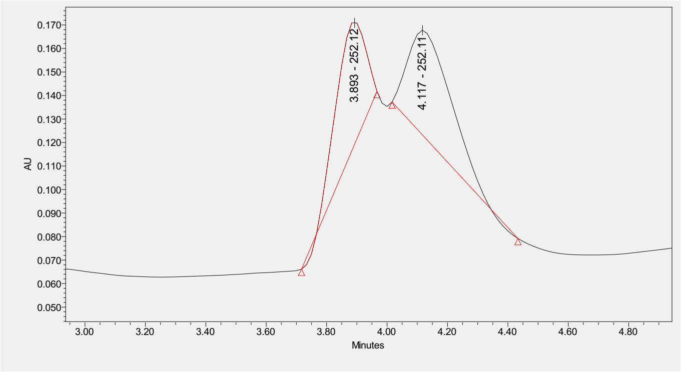

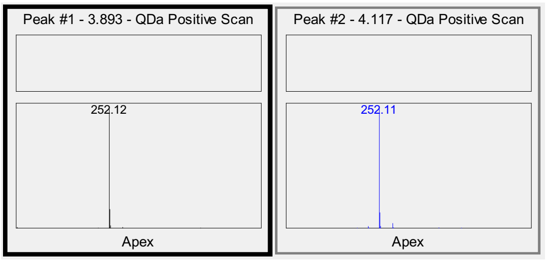

Biocatalytic – 1 peak with product mass

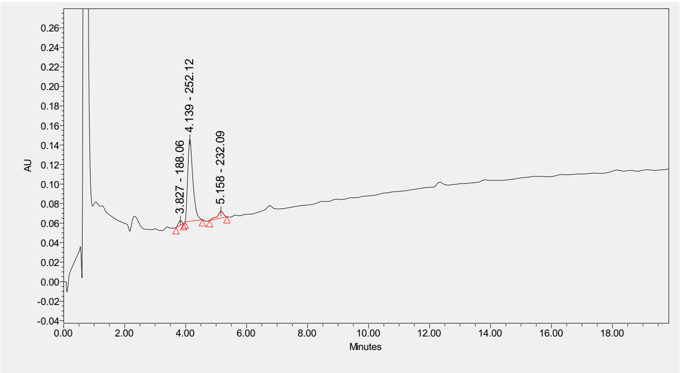

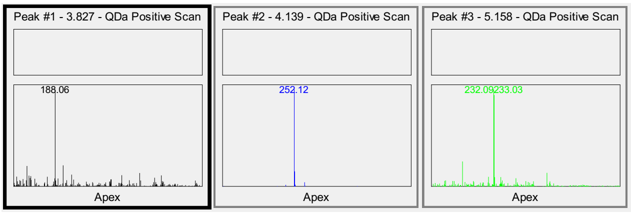

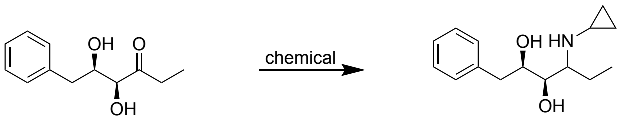

2c 2ci

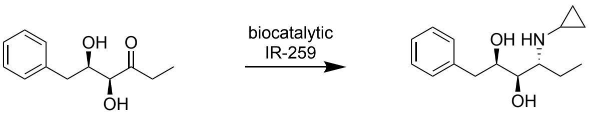

2c 2ci

Chemical – 3 peaks with product mass

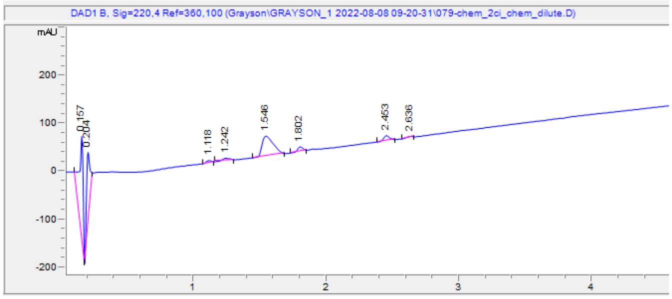

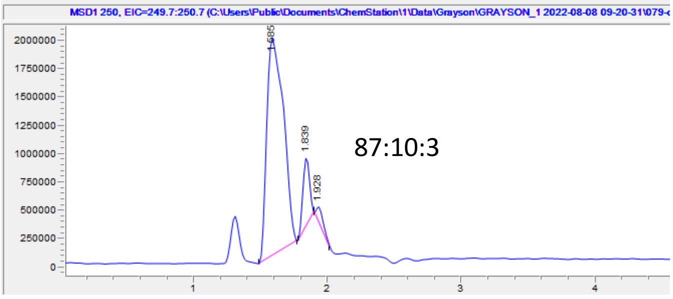

Biocatalytic – 1 peak with product mass

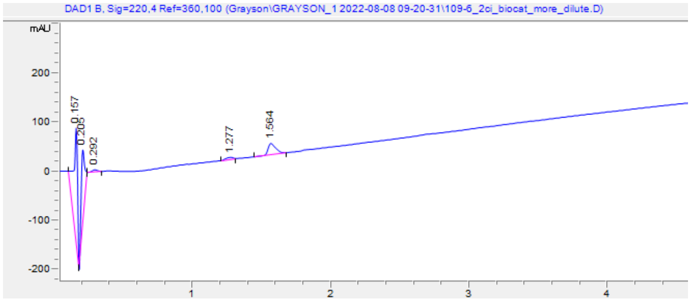

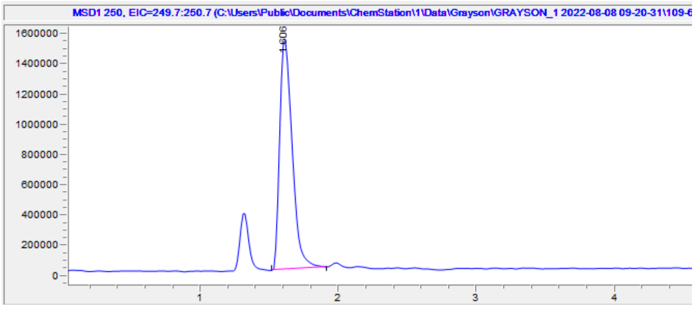

UPLC QDa Chromatograms for determination of conversion

Substrates

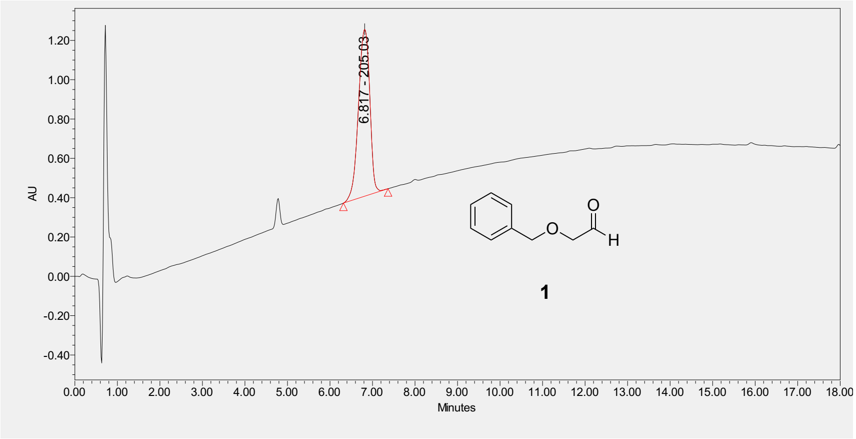

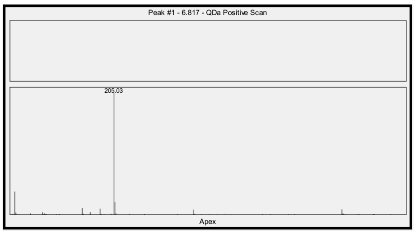

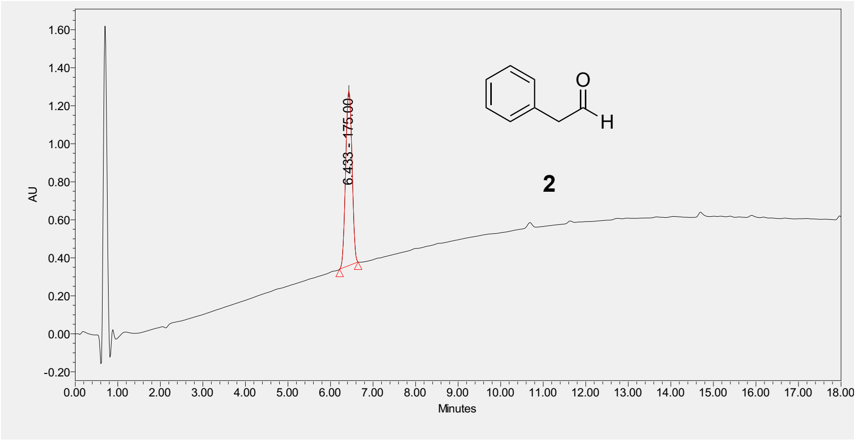

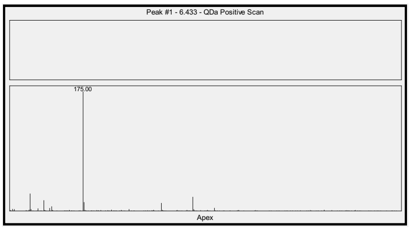

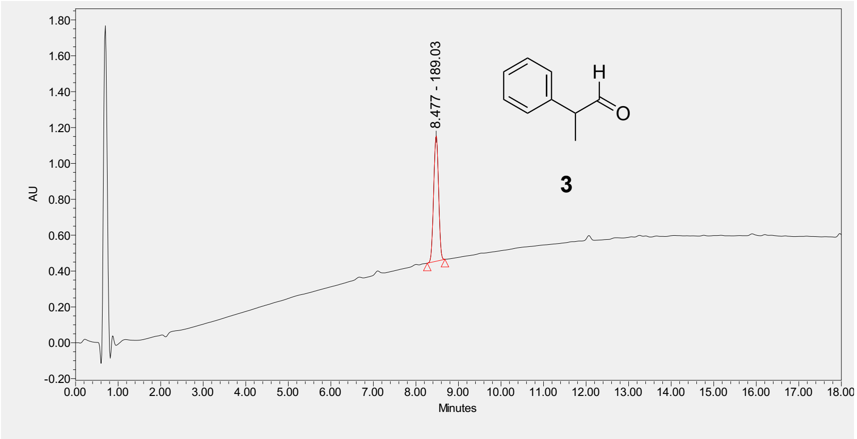

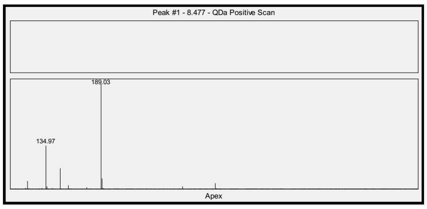

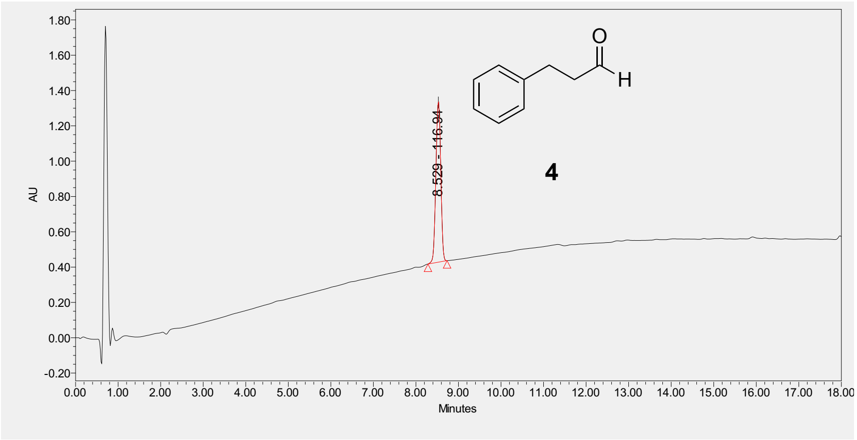

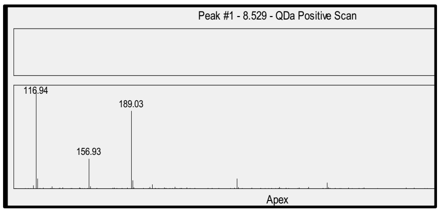

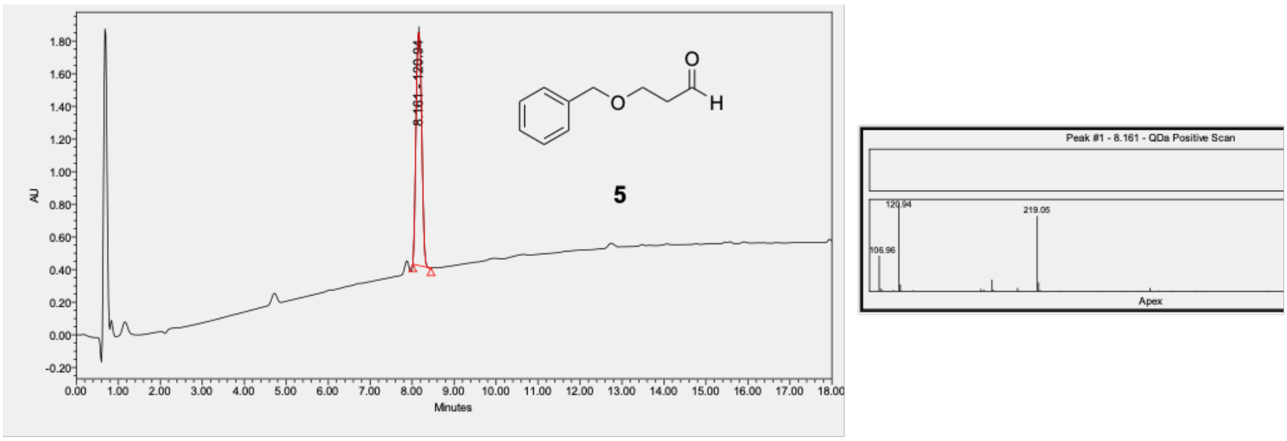
8.161 - 120.94
5
AU
Minutes
Peak #1 - 8.161 - QDa Positive Scan
106.96
120.94
219.05
Apex

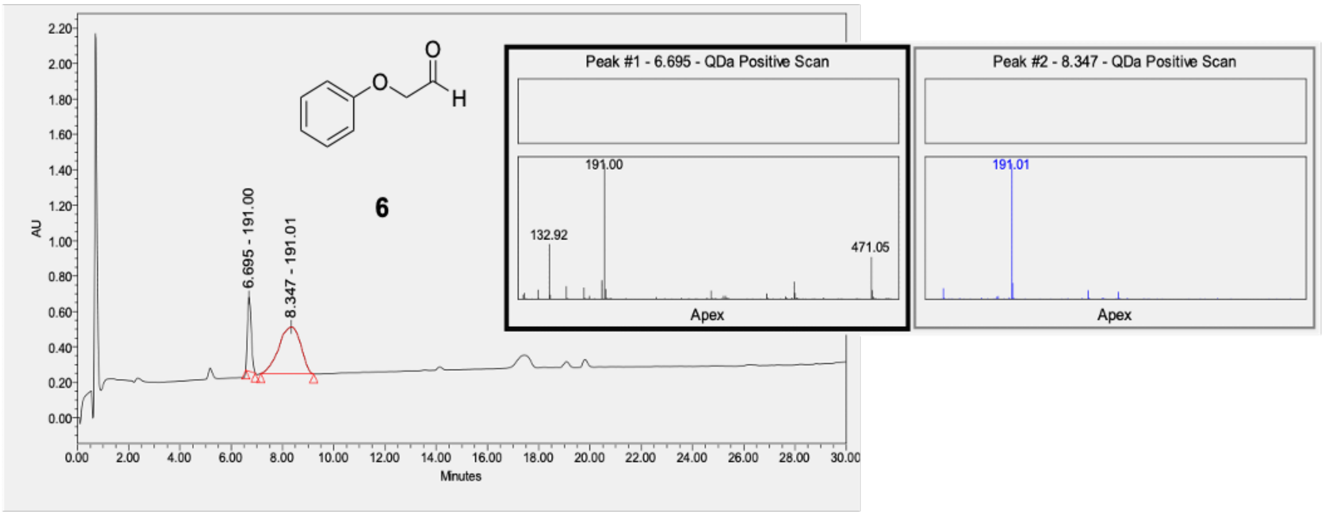
6
6.695 - 191.00
8.347 - 191.01
AU
Minutes
Peak #1 - 6.695 - QDa Positive Scan
191.00
132.92
471.05
Apex
Peak #2 - 8.347 - QDa Positive Scan
191.01
Apex

Example chromatograms for screening FSAs for the aldol addition reactions

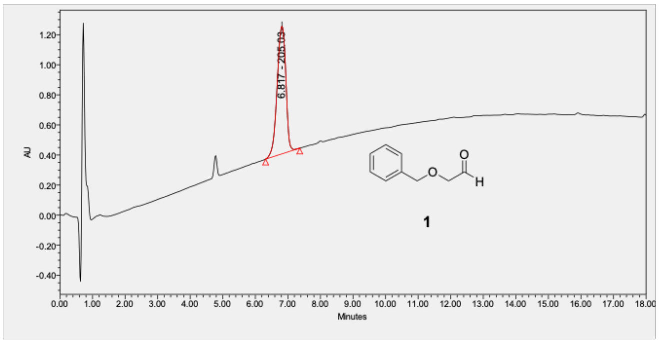

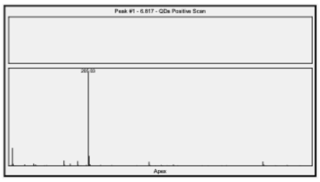

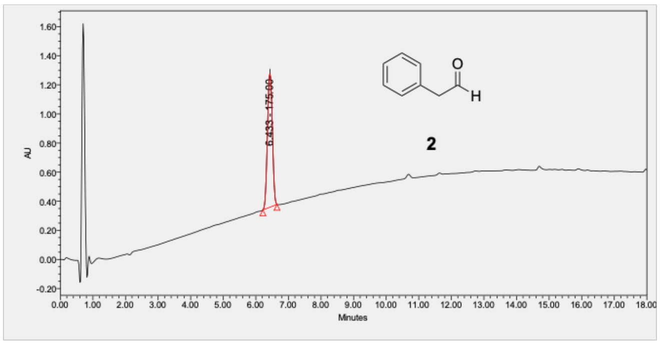

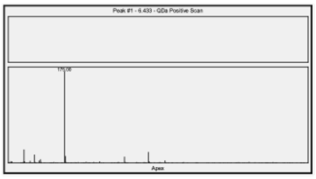

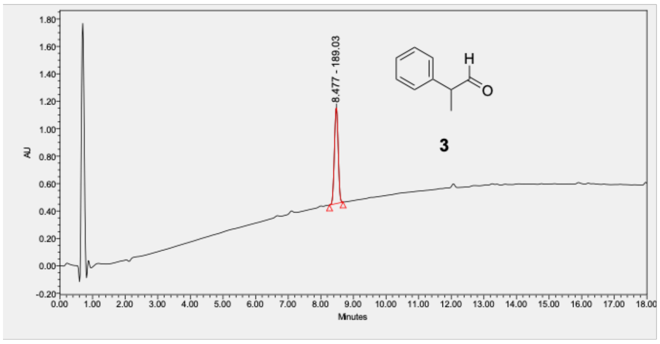

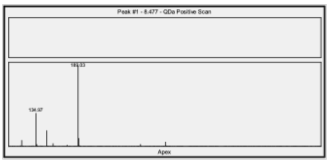

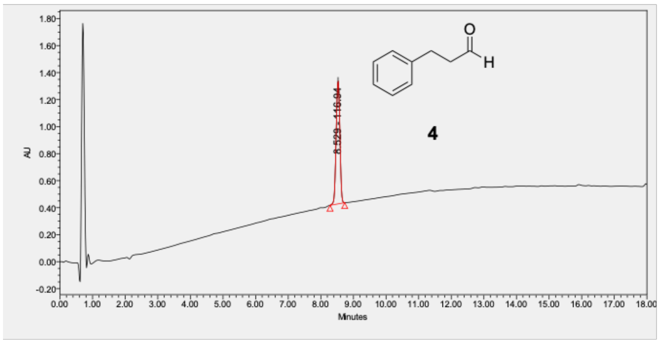

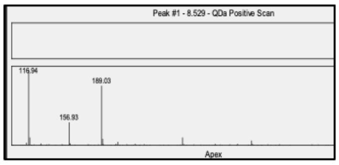

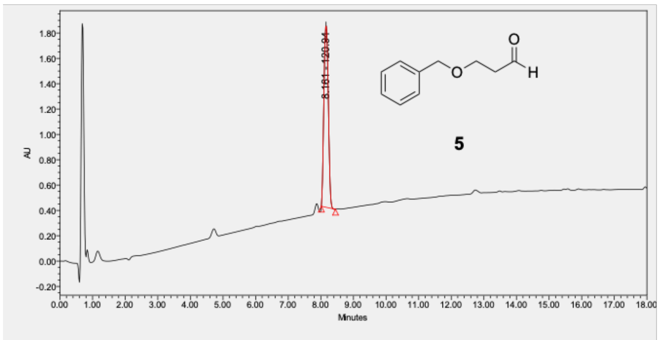

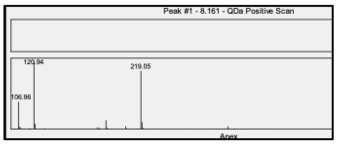

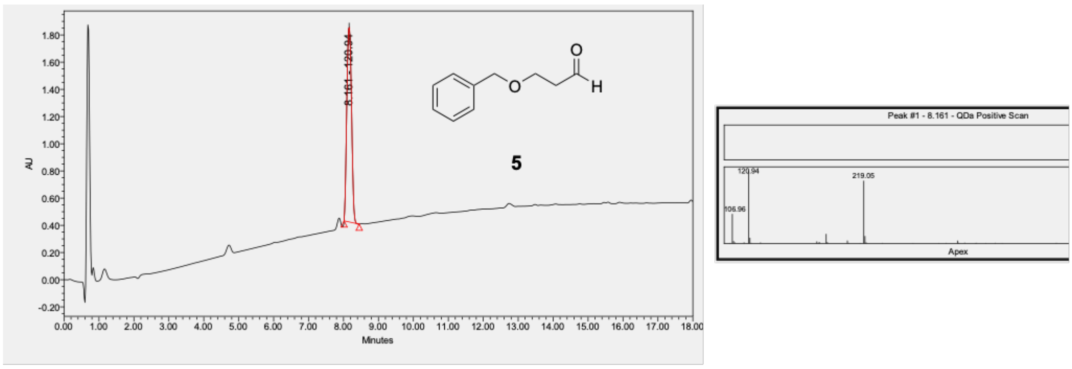
8.161 - 120.94
5
AU
Minutes
Peak #1 - 8.161 - QDa Positive Scan
120.94
219.05
106.96
Apex

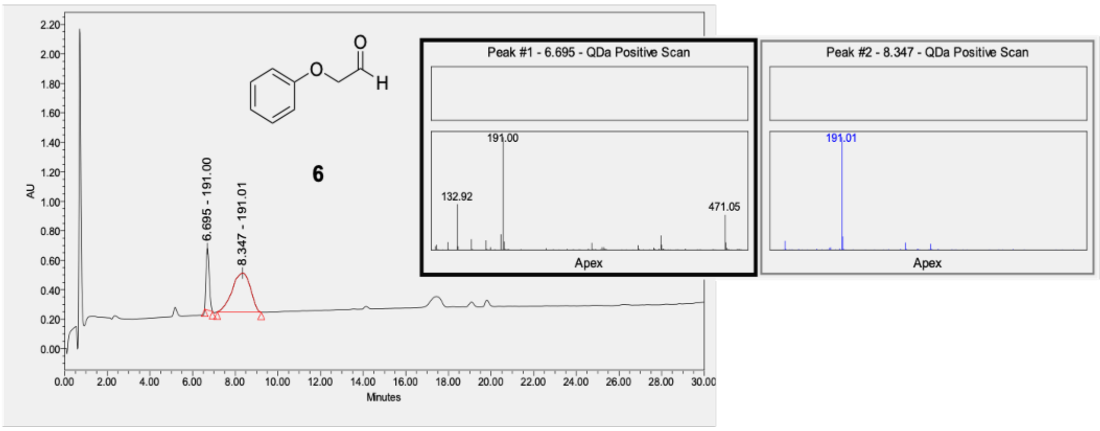
6
AU
6.695 - 191.00
8.347 - 191.01
Minutes
Peak #1 - 6.695 - QDa Positive Scan
191.00
132.92
471.05
Apex
Peak #2 - 8.347 - QDa Positive Scan
191.01
Apex

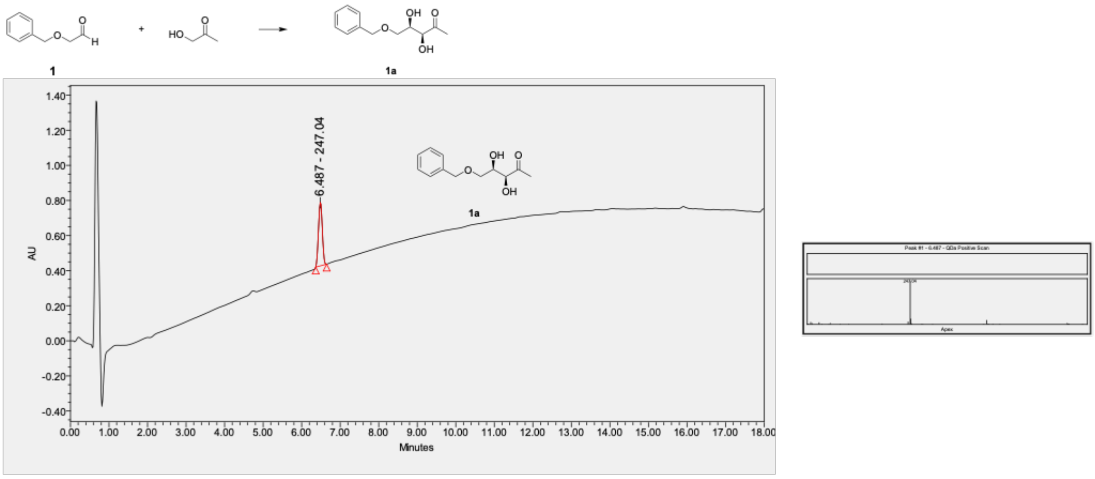

1
1a
6.487 - 247.04
AU
Minutes

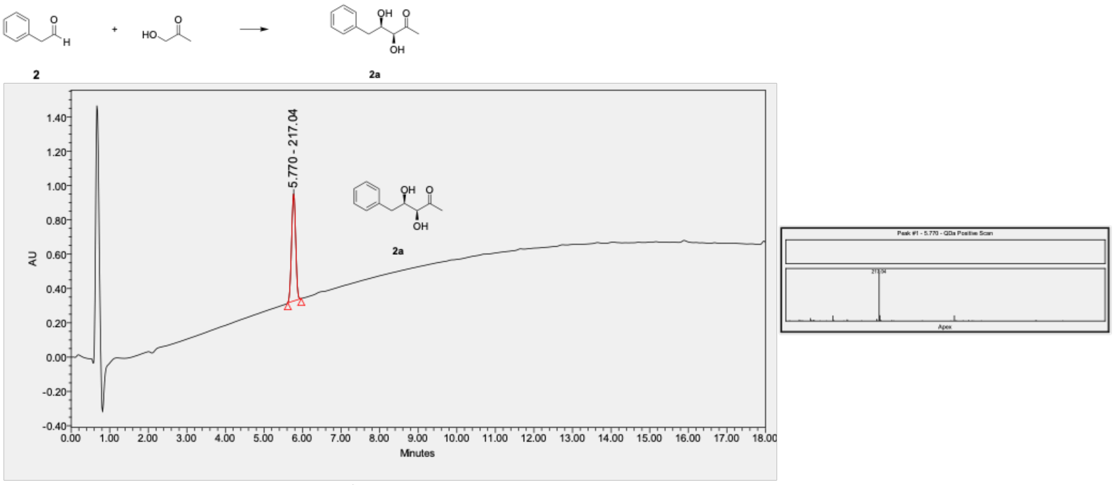

2
2a
5.770 - 217.04
AU
Minutes

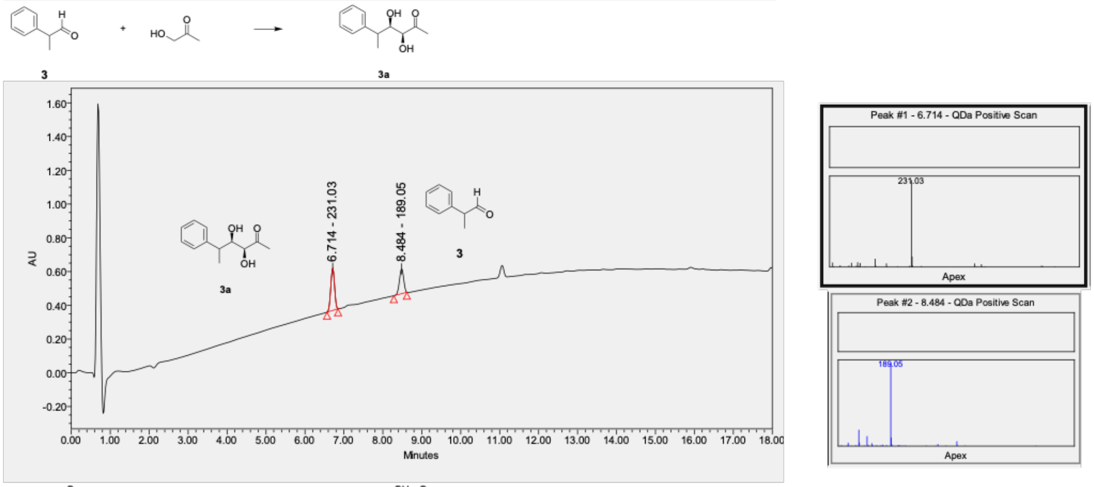

3
3a
6.714 - 231.03
8.484 - 189.05
AU
Minutes
Peak #1 - 6.714 - QDa Positive Scan
Apex
Peak #2 - 8.484 - QDa Positive Scan
Apex

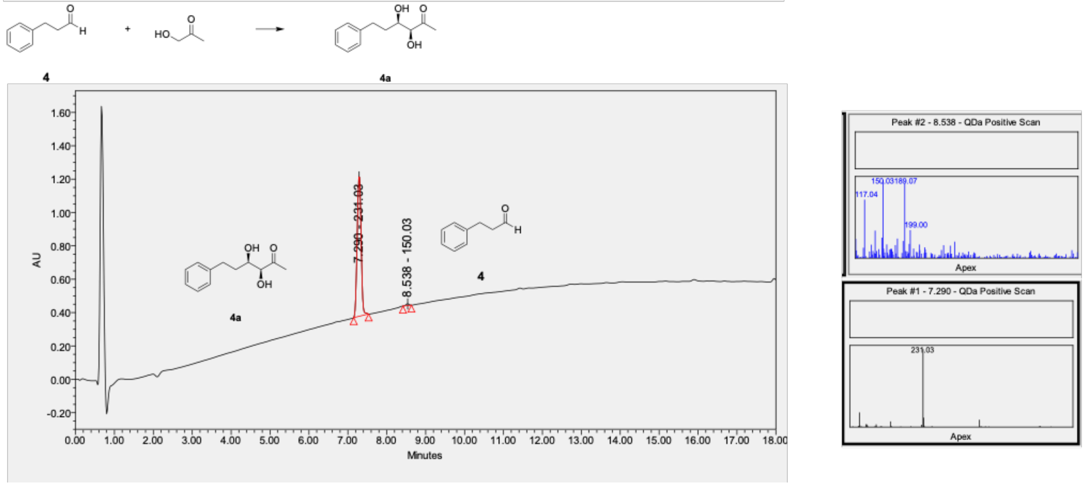

4
4a
7.290 - 231.03
8.538 - 150.03
AU
Minutes
Peak #2 - 8.538 - QDa Positive Scan
Apex
Peak #1 - 7.290 - QDa Positive Scan
Apex

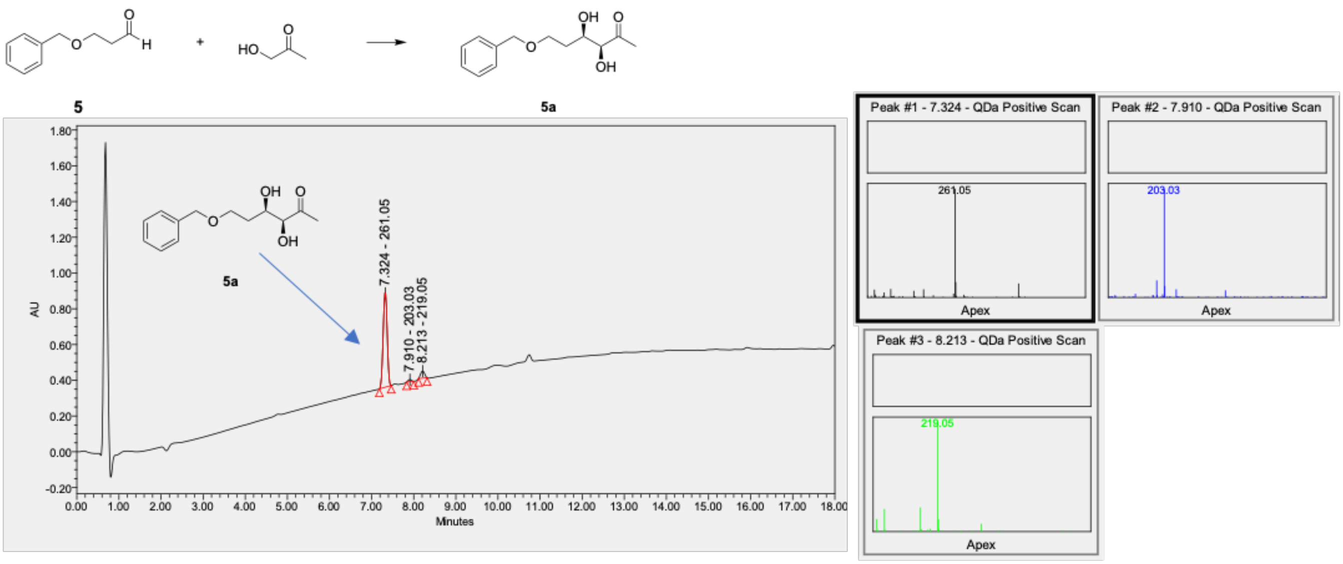
5
5a
Peak #1 - 7.324 - QDa Positive Scan
261.05
Apex
Peak #2 - 7.910 - QDa Positive Scan
203.03
Apex
Peak #3 - 8.213 - QDa Positive Scan
219.05
Apex
7.324 - 261.05
7.910 - 203.03
8.213 - 219.05
AU
Minutes

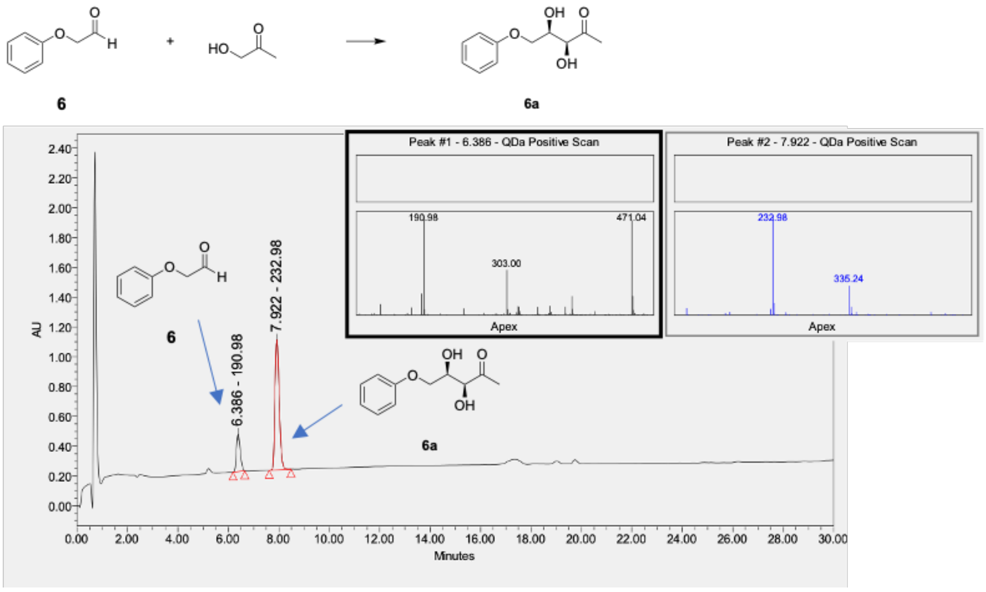
6
6a
Peak #1 - 6.386 - QDa Positive Scan
190.98
303.00
471.04
Apex
Peak #2 - 7.922 - QDa Positive Scan
232.98
335.24
Apex
6.386 - 190.98
7.922 - 232.98
AU
Minutes

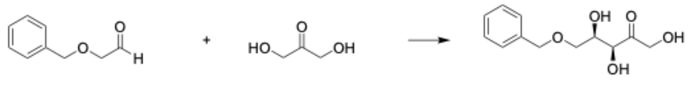

1 **1b**

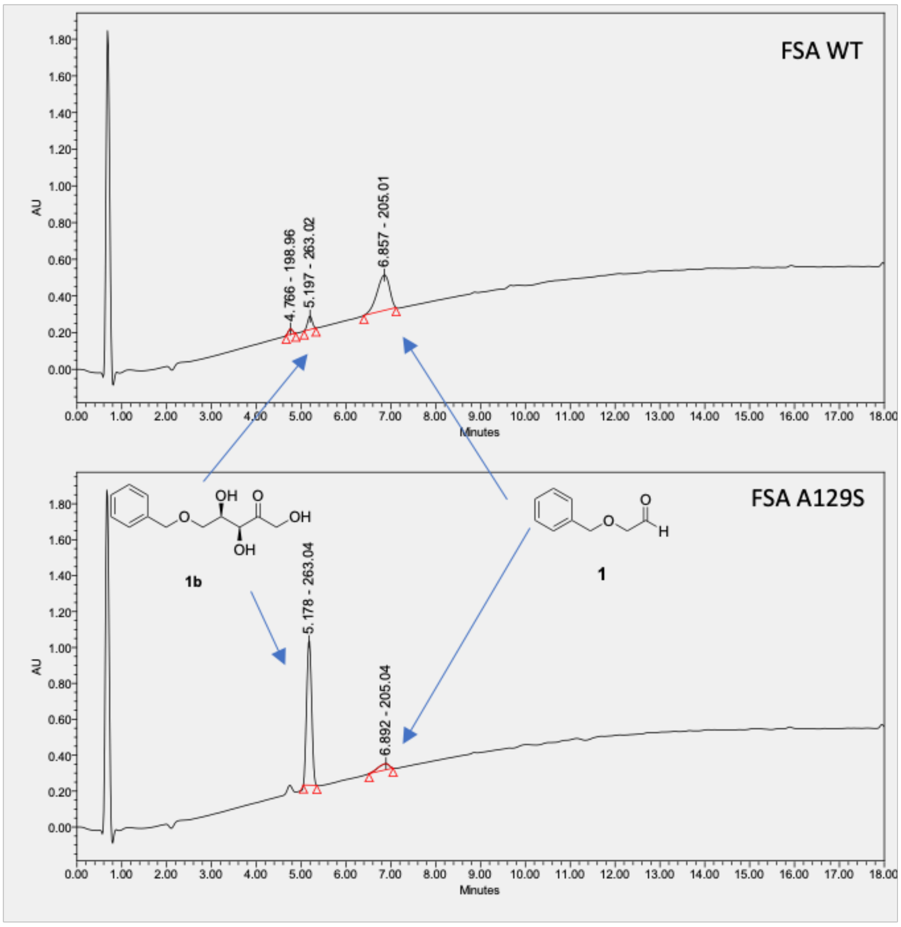
FSA WT
FSA A129S
AU
Minutes
4.766 - 198.96
5.197 - 263.02
6.857 - 205.01
5.178 - 263.04
6.892 - 205.04
1b
1

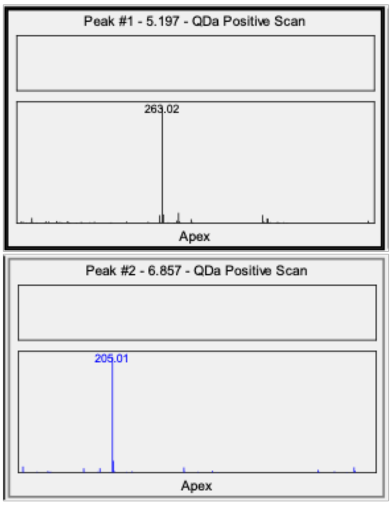
Peak #1 - 5.197 - QDa Positive Scan
263.02
Apex
Peak #2 - 6.857 - QDa Positive Scan
205.01
Apex

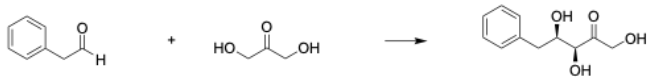

2 **2b**

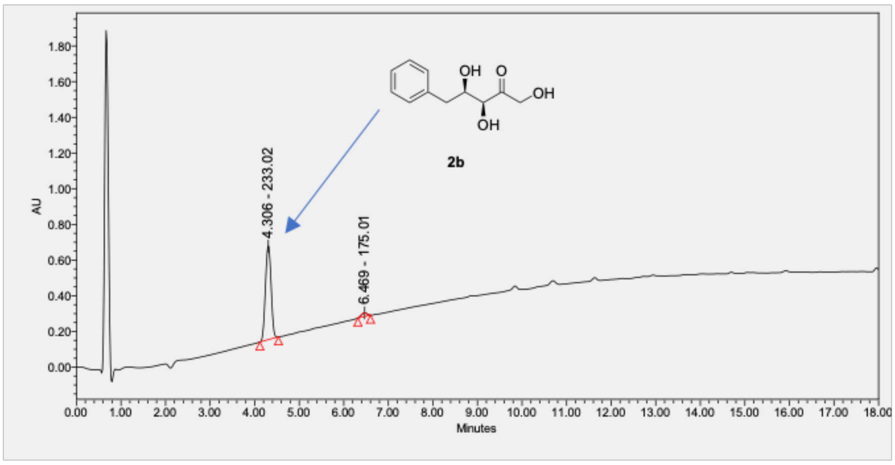
AU
Minutes
4.306 - 233.02
6.469 - 175.01
2b

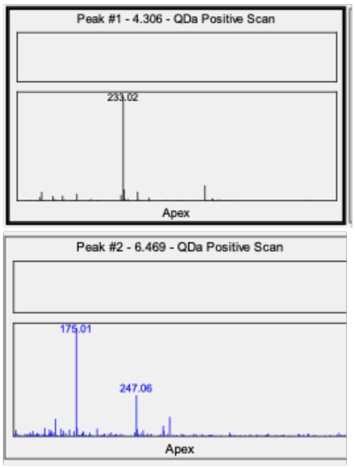
Peak #1 - 4.306 - QDa Positive Scan
233.02
Apex
Peak #2 - 6.469 - QDa Positive Scan
175.01
247.06
Apex

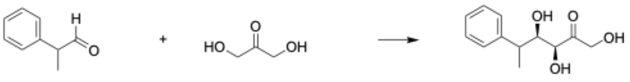

H
O
+
HO
O
OH
OH
O
OH
OH

3 **3b**

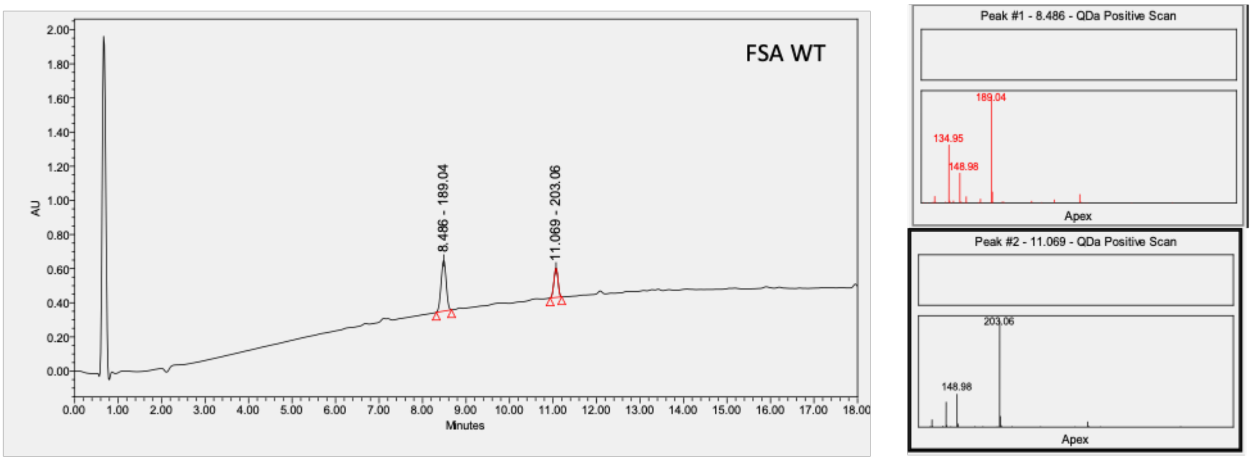

FSA WT
AU
Minutes
8.486 - 189.04
11.069 - 203.06
Peak #1 - 8.486 - QDa Positive Scan
189.04
134.95
148.98
Apex
Peak #2 - 11.069 - QDa Positive Scan
203.06
148.98
Apex

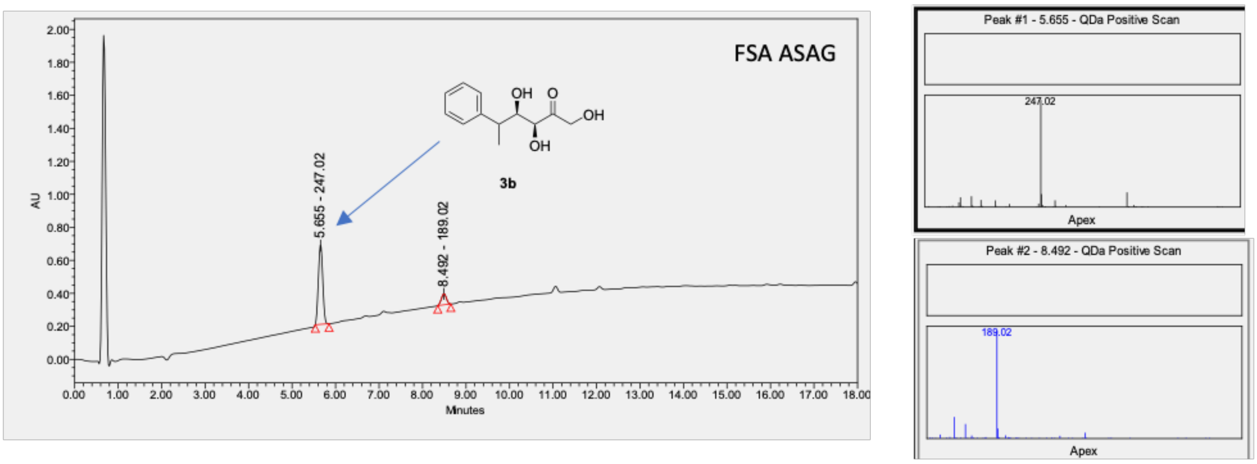

FSA ASAG
AU
Minutes
5.655 - 247.02
8.492 - 189.02
3b
Peak #1 - 5.655 - QDa Positive Scan
247.02
Apex
Peak #2 - 8.492 - QDa Positive Scan
189.02
Apex

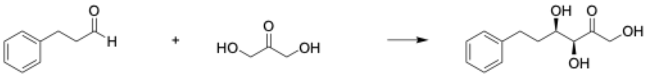

O
H
+
HO
O
OH
OH
O
OH
OH

4 **4b**

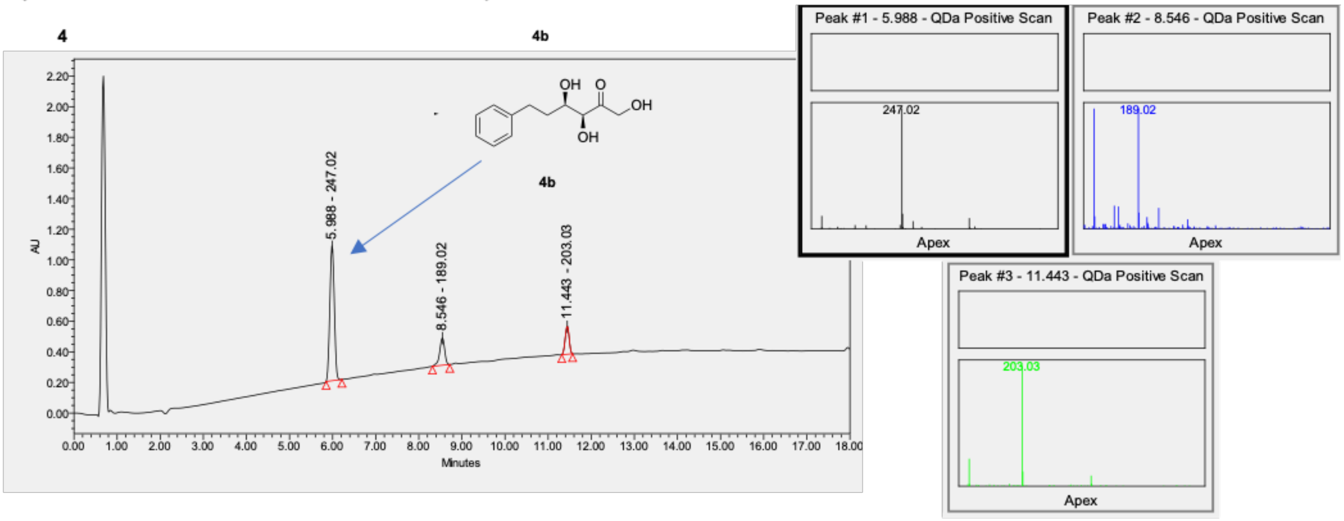

AU
Minutes
5.988 - 247.02
8.546 - 189.02
11.443 - 203.03
4b
Peak #1 - 5.988 - QDa Positive Scan
247.02
Apex
Peak #2 - 8.546 - QDa Positive Scan
189.02
Apex
Peak #3 - 11.443 - QDa Positive Scan
203.03
Apex

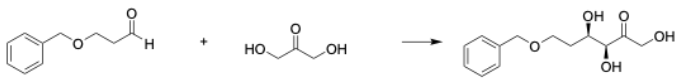

5 **5b**

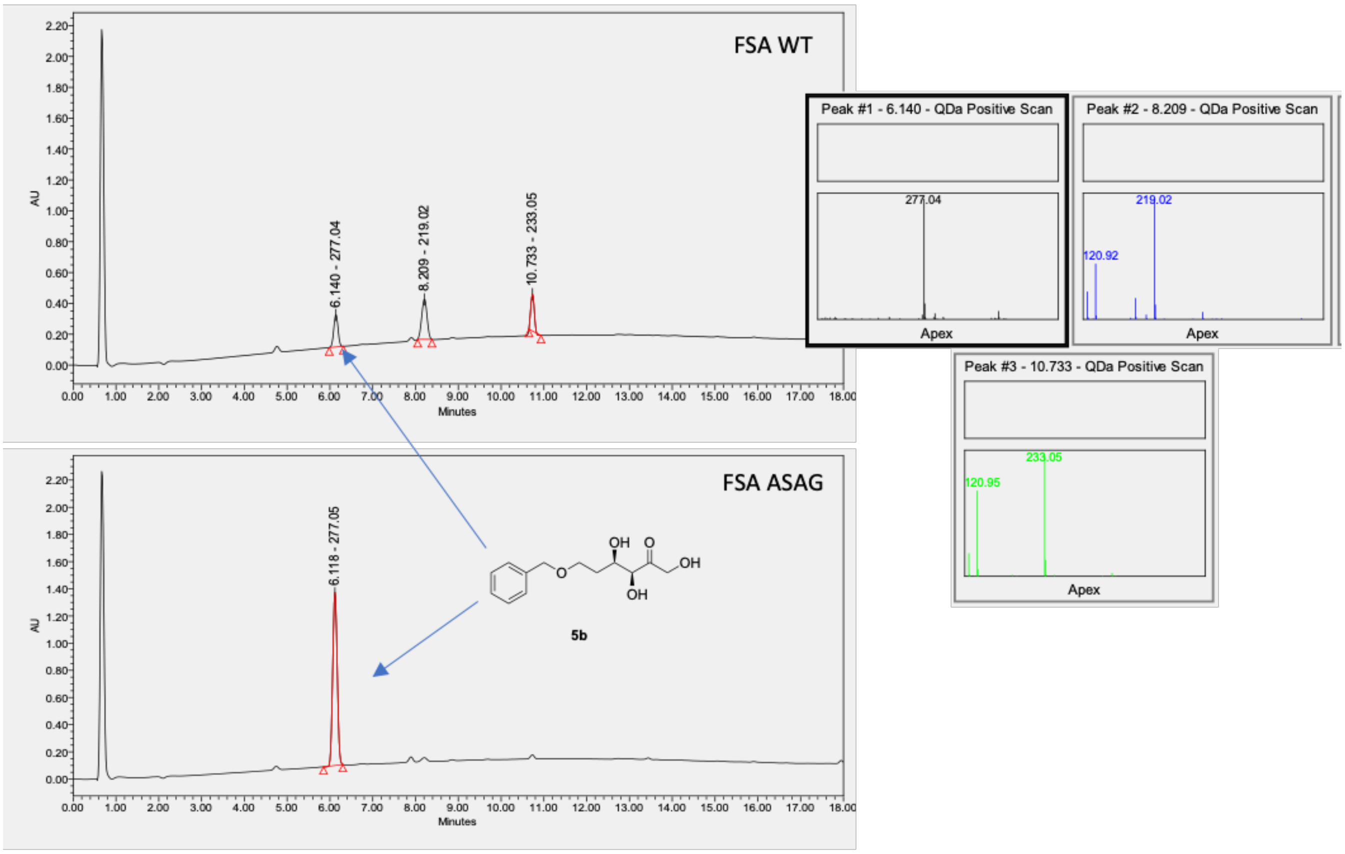
FSA WT
Peak #1 - 6.140 - QDa Positive Scan
277.04
Apex
Peak #2 - 8.209 - QDa Positive Scan
219.02
120.92
Apex
Peak #3 - 10.733 - QDa Positive Scan
233.05
120.95
Apex
6.140 - 277.04
8.209 - 219.02
10.733 - 233.05
AU
Minutes
FSA ASAG
6.118 - 277.05
5b

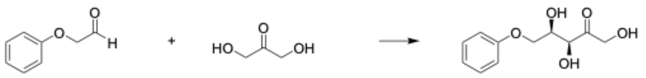

6 **6b**

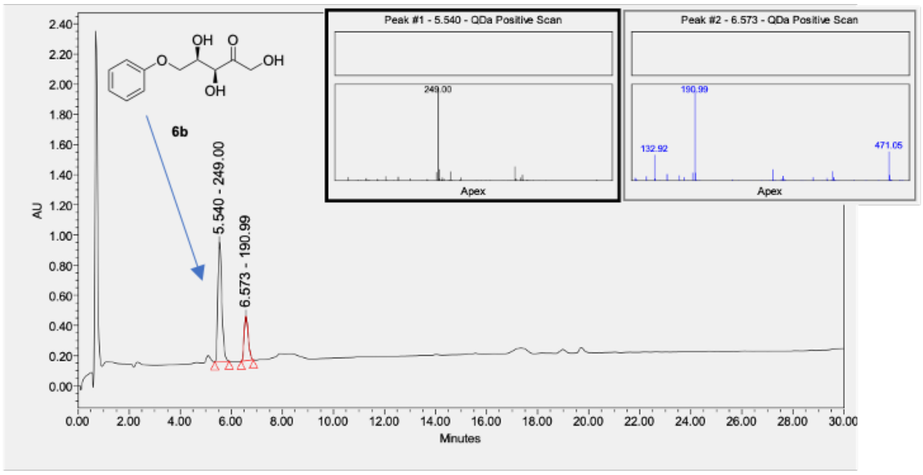
Peak #1 - 5.540 - QDa Positive Scan
249.00
Apex
Peak #2 - 6.573 - QDa Positive Scan
190.99
132.92
471.05
Apex
6b
5.540 - 249.00
6.573 - 190.99
AU
Minutes

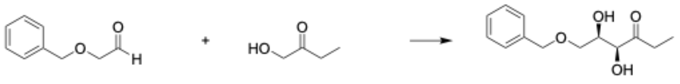

HO
OH
O
OH

1 **1c**

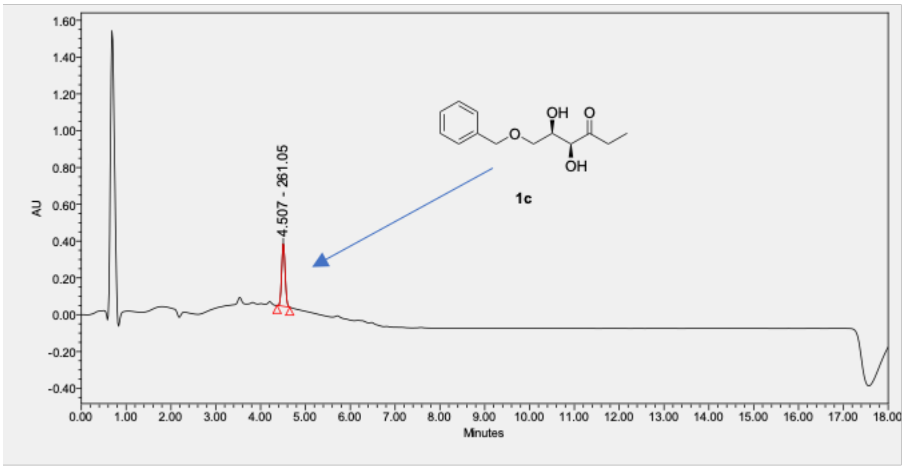

AU
Minutes
4.507 - 261.05
OH
O
OH
1c

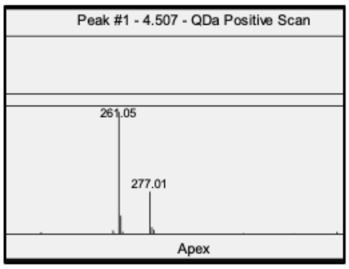

Peak #1 - 4.507 - QDa Positive Scan
261.05
277.01
Apex

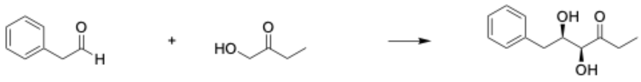

HO
OH
O
OH

2 **2c**

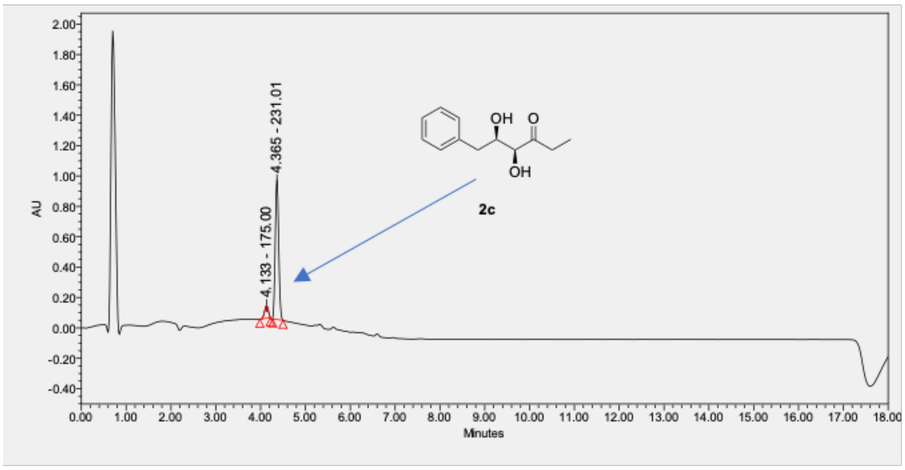

AU
Minutes
4.365 - 231.01
4.133 - 175.00
OH
O
OH
2c

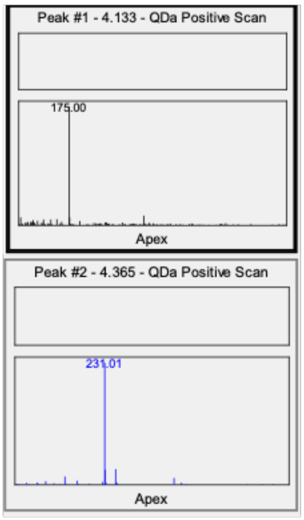

Peak #1 - 4.133 - QDa Positive Scan
175.00
Apex
Peak #2 - 4.365 - QDa Positive Scan
231.01
Apex

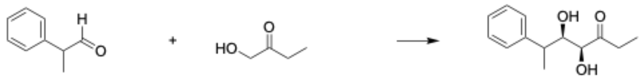

H
O
HO
OH
O
OH

3 **3c**

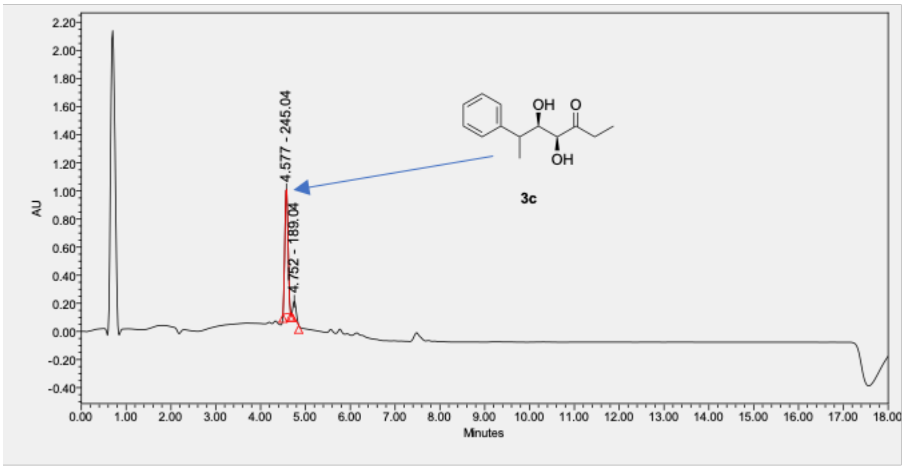

AU
Minutes
4.577 - 245.04
4.752 - 189.04
OH
O
OH
3c

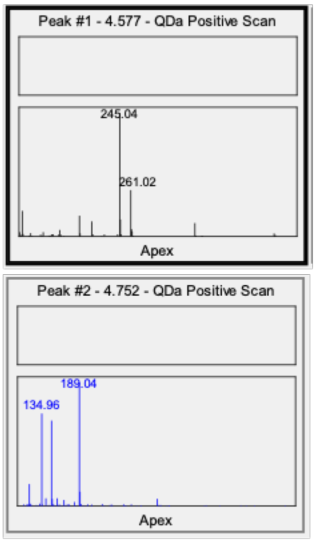

Peak #1 - 4.577 - QDa Positive Scan
245.04
261.02
Apex
Peak #2 - 4.752 - QDa Positive Scan
189.04
134.96
Apex

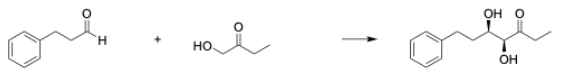

4 **4c**

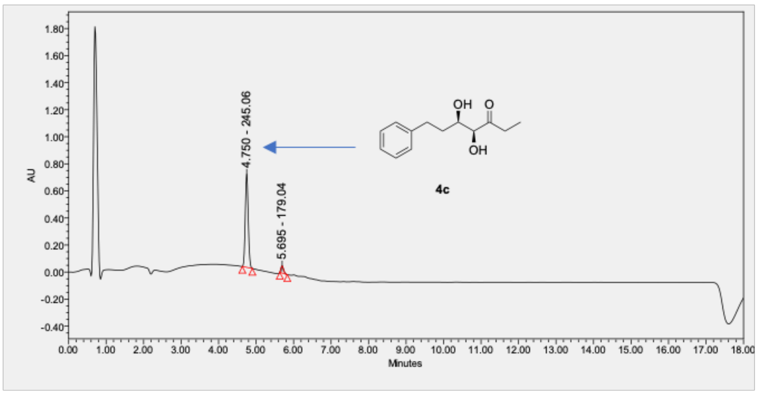

AU
Minutes
4.750 - 245.06
5.695 - 179.04
4c

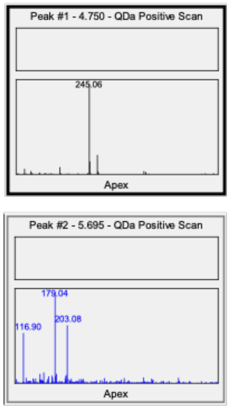

Peak #1 - 4.750 - QDa Positive Scan
245.06
Apex
Peak #2 - 5.695 - QDa Positive Scan
179.04
203.08
116.90
Apex

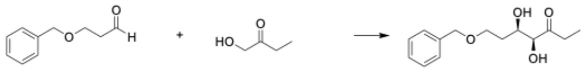

5 **5c**

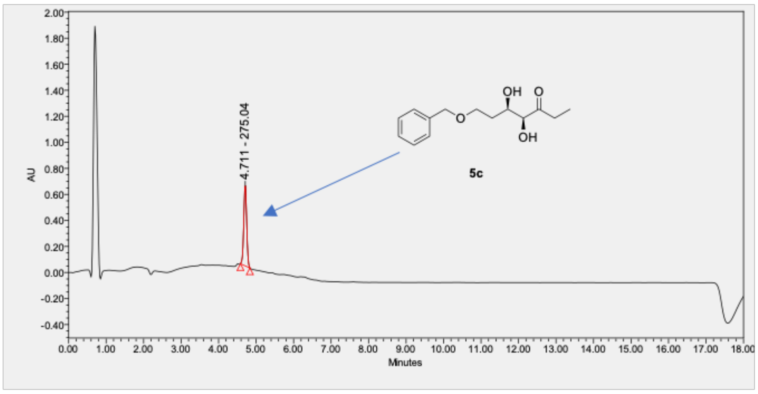

AU
Minutes
4.711 - 275.04
5c

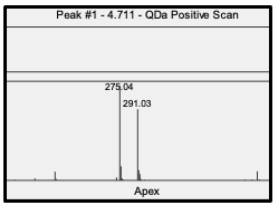

Peak #1 - 4.711 - QDa Positive Scan
275.04
291.03
Apex

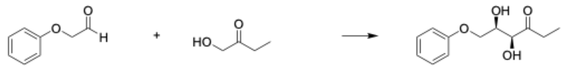

6 **6c**

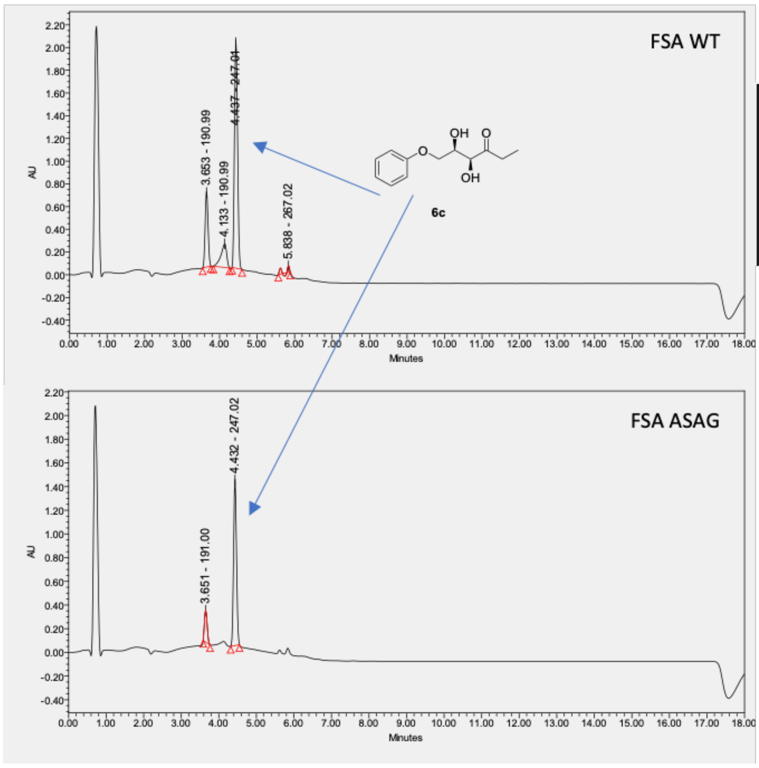

FSA WT
AU
Minutes
3.653 - 190.99
4.133 - 190.99
4.437 - 247.01
5.838 - 267.02
6c
FSA ASAG
3.651 - 191.00
4.432 - 247.02

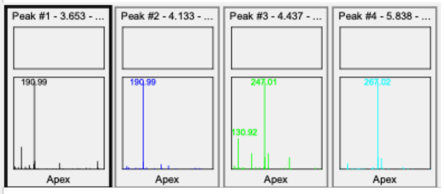

Peak #1 - 3.653 - ...
190.99
Apex
Peak #2 - 4.133 - ...
190.99
Apex
Peak #3 - 4.437 - ...
247.01
130.92
Apex
Peak #4 - 5.838 - ...
267.02
Apex

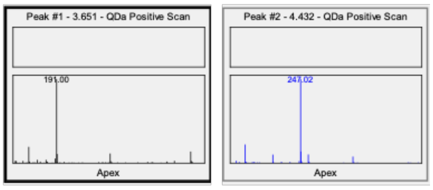

Peak #1 - 3.651 - QDa Positive Scan
191.00
Apex
Peak #2 - 4.432 - QDa Positive Scan
247.02
Apex

Example Chromatograms for determination of dr for FSA mediated aldol reactions with aldehyde 3

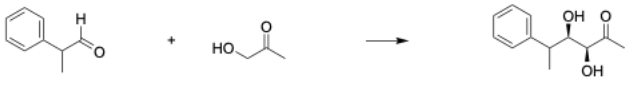

3 3a

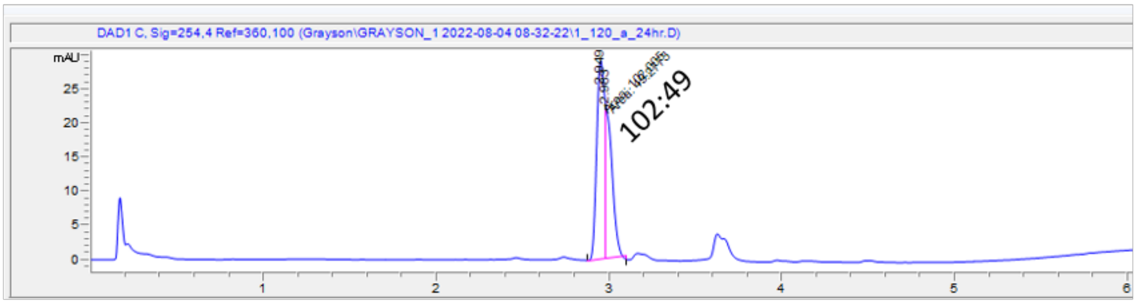

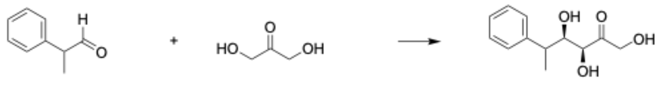

3 3b

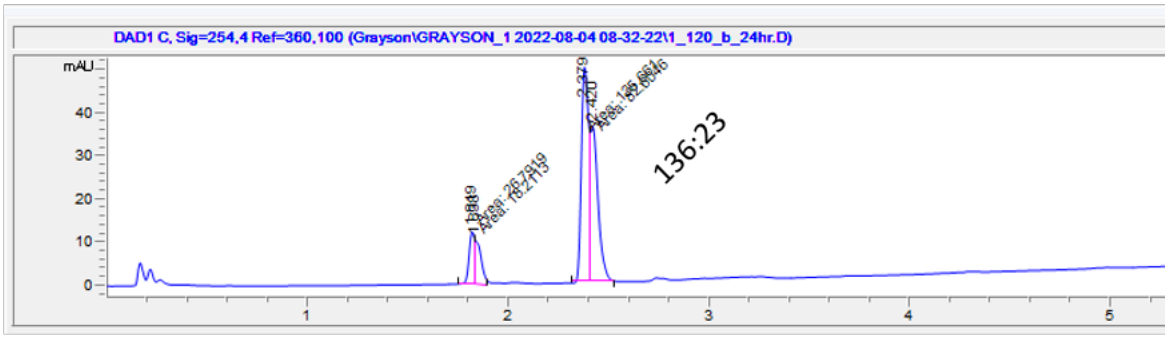

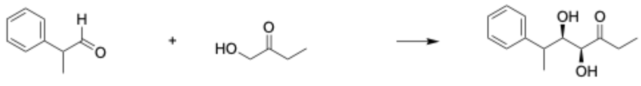

3 3c

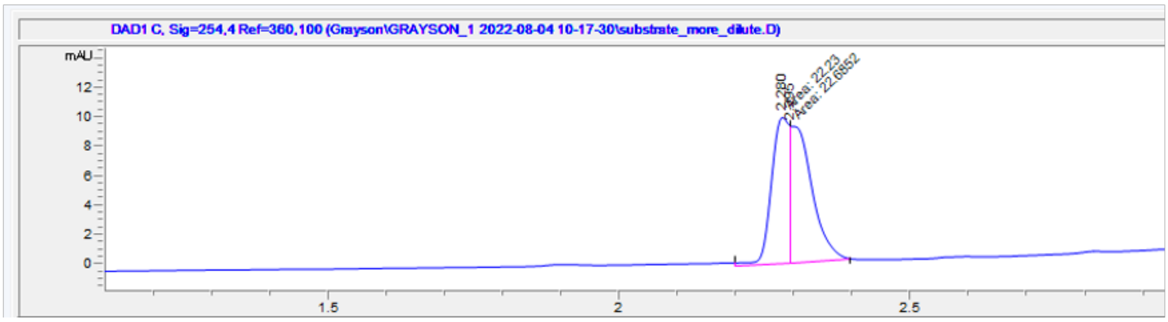

Example Chromatograms for the IR-259 mediated reductive amination of aldol adduct substrates

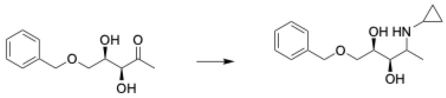

1a 1ai

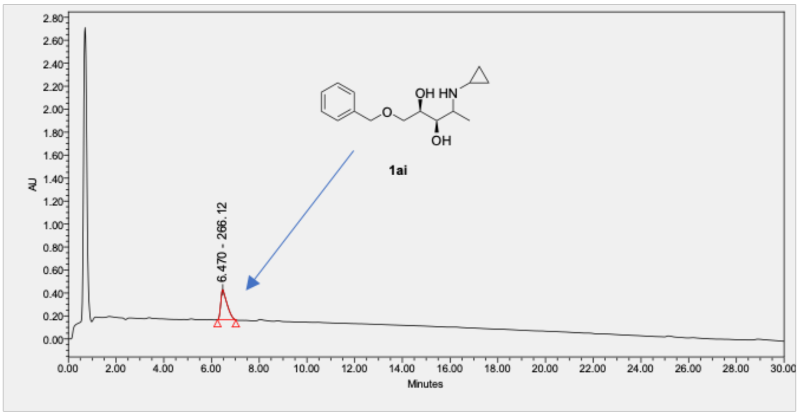

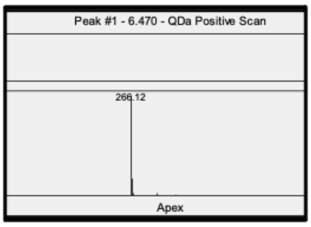

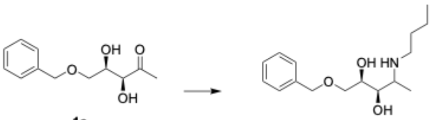

1a 1aii

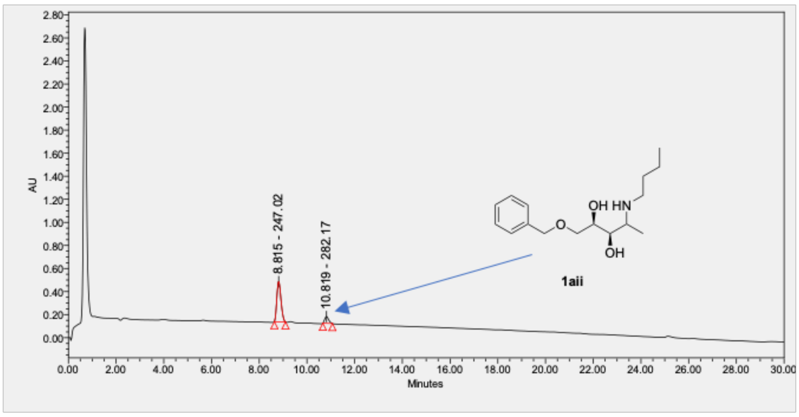

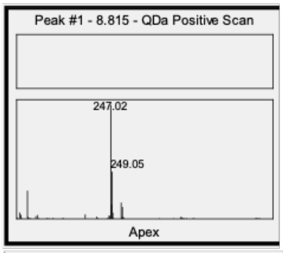

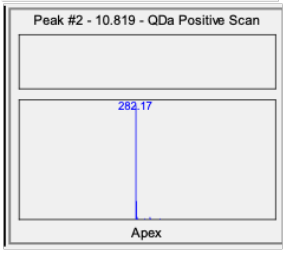

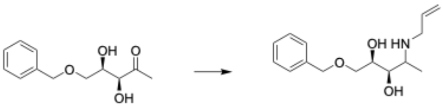

1a 1aiii

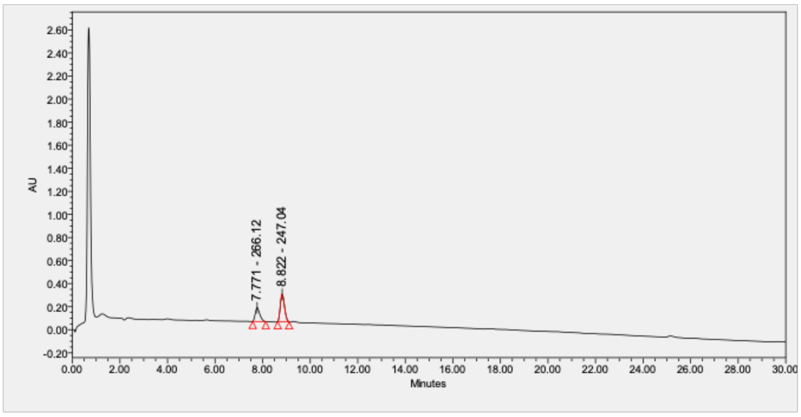

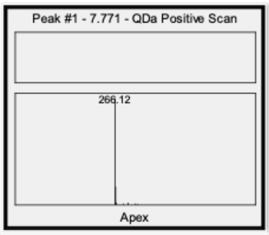

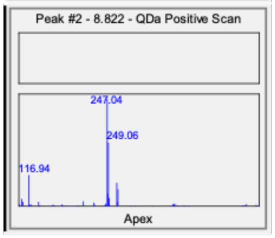

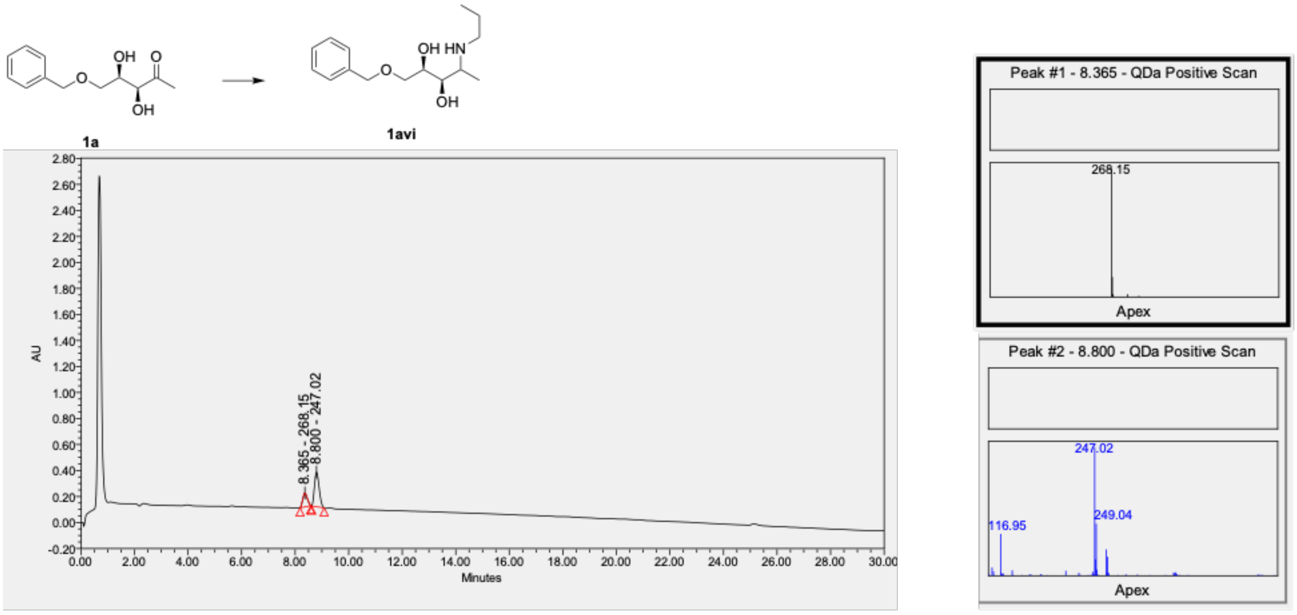
1a
1avi
8.365 - 268.15
8.800 - 247.02
AU
Minutes
Peak #1 - 8.365 - QDa Positive Scan
268.15
Apex
Peak #2 - 8.800 - QDa Positive Scan
247.02
249.04
116.95
Apex

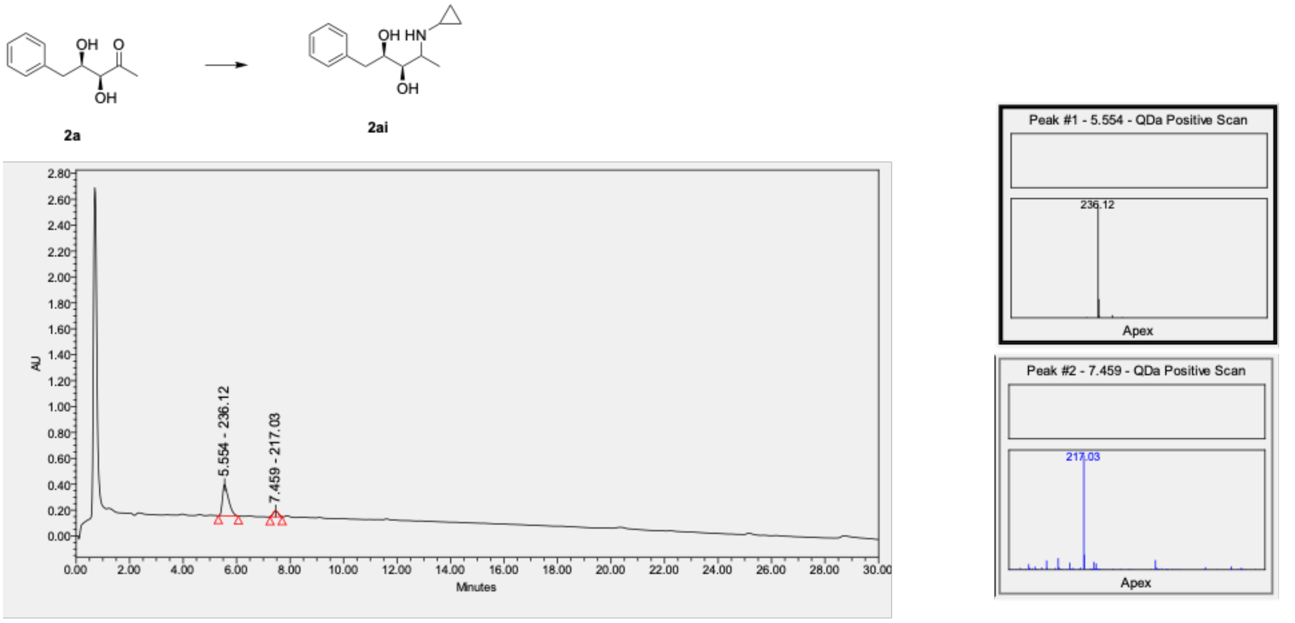
2a
2ai
5.554 - 236.12
7.459 - 217.03
AU
Minutes
Peak #1 - 5.554 - QDa Positive Scan
236.12
Apex
Peak #2 - 7.459 - QDa Positive Scan
217.03
Apex

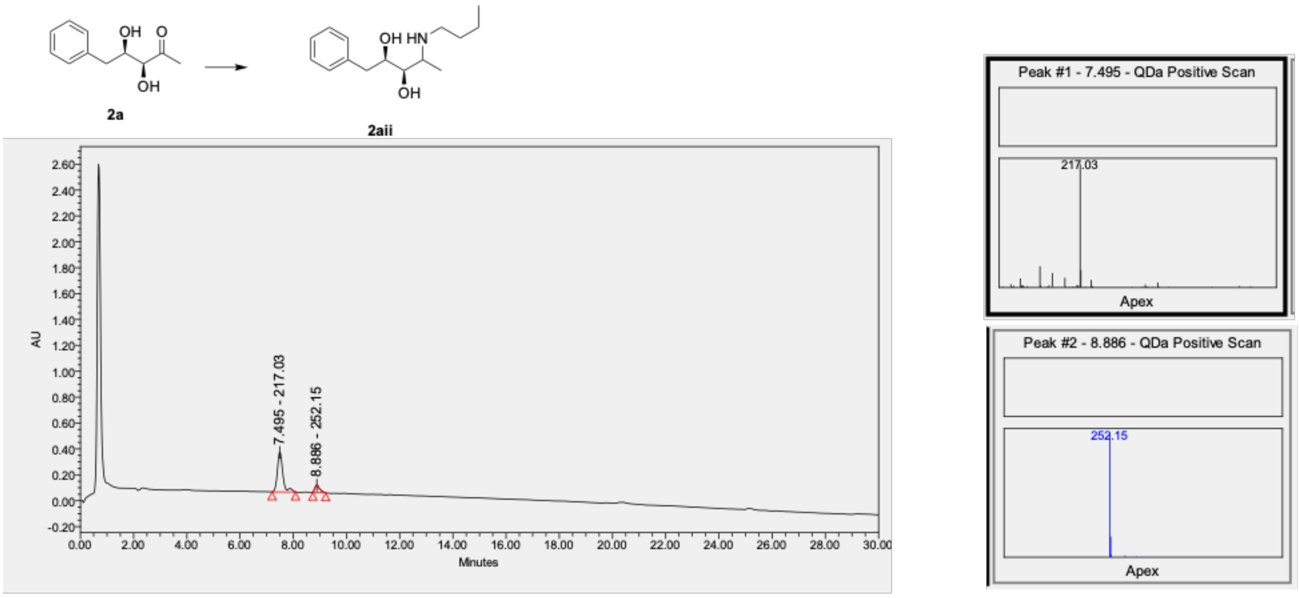
2a
2aii
7.495 - 217.03
8.886 - 252.15
AU
Minutes
Peak #1 - 7.495 - QDa Positive Scan
217.03
Apex
Peak #2 - 8.886 - QDa Positive Scan
252.15
Apex

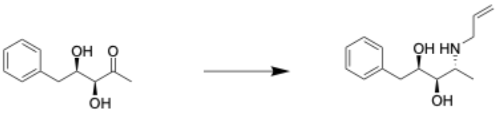

2a **2aiii**

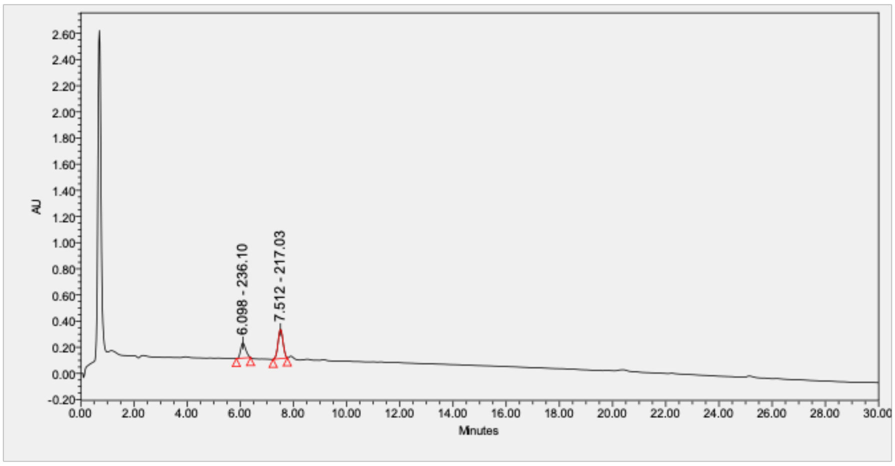

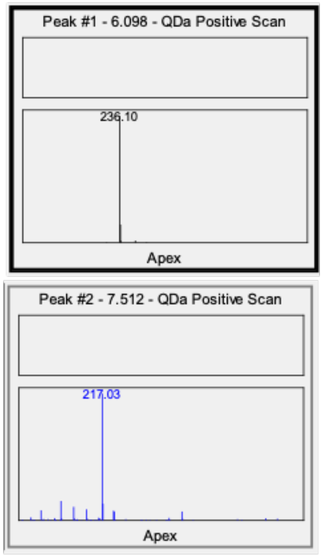

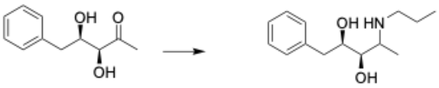

2a **2avi**

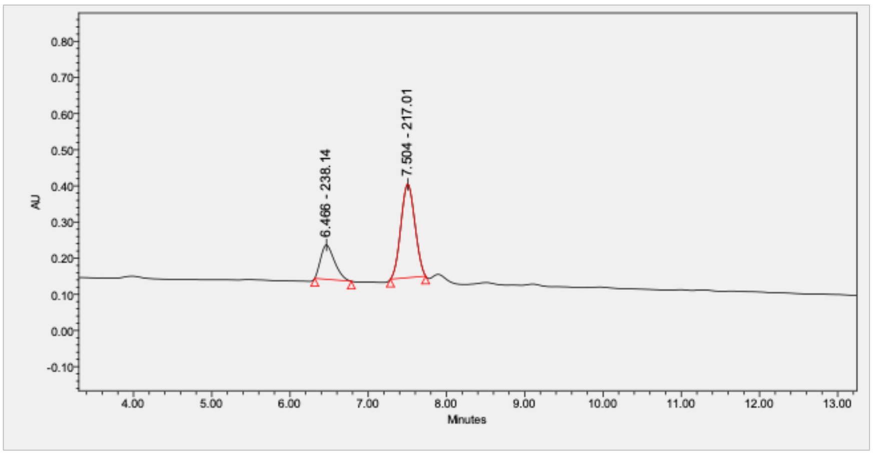

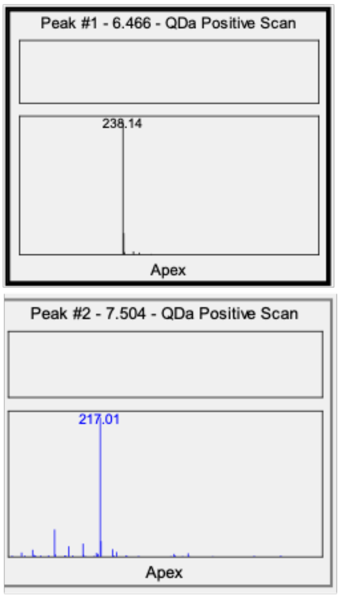

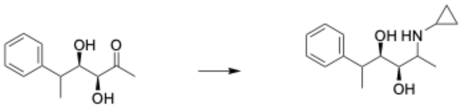

3a **3ai**

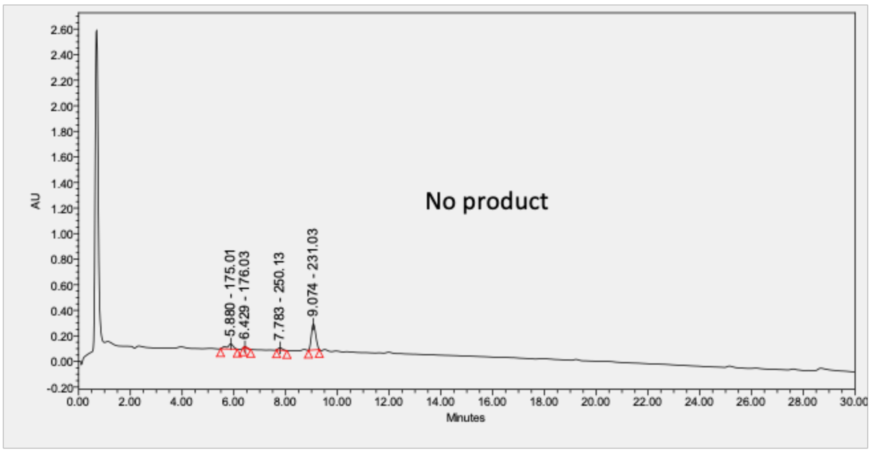

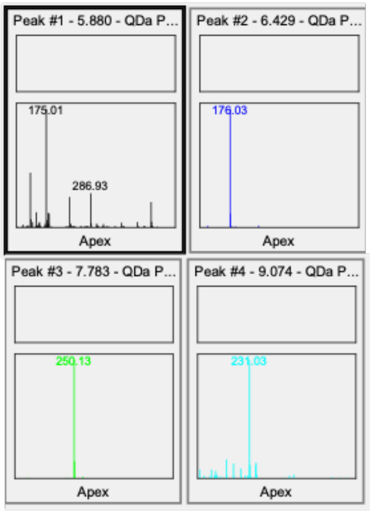

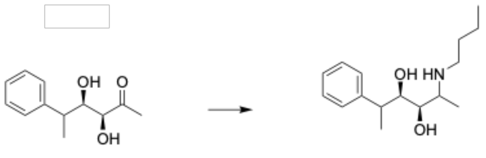

3a **3aii**

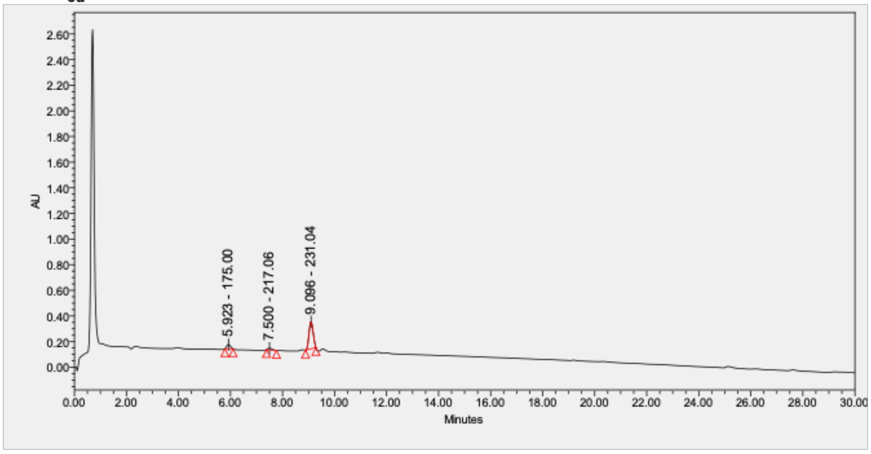

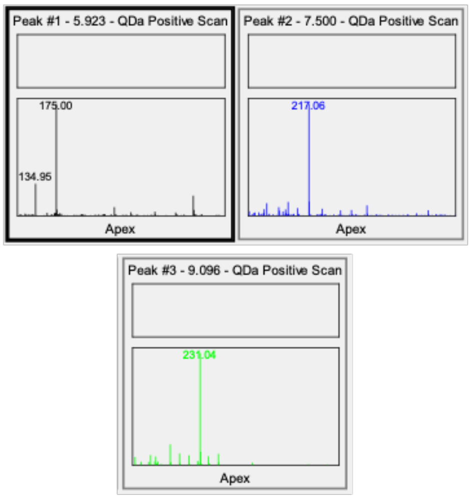

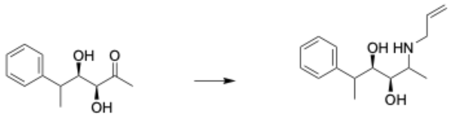

3a **3aiii**

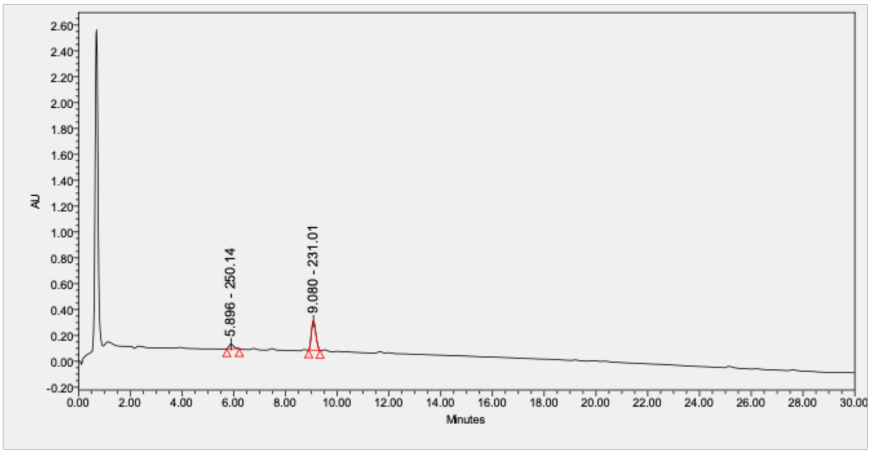

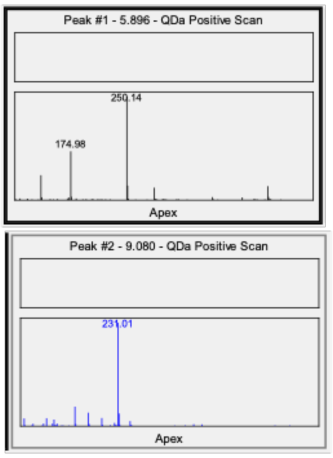

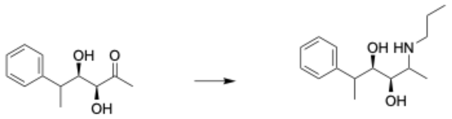

3a **3avi**

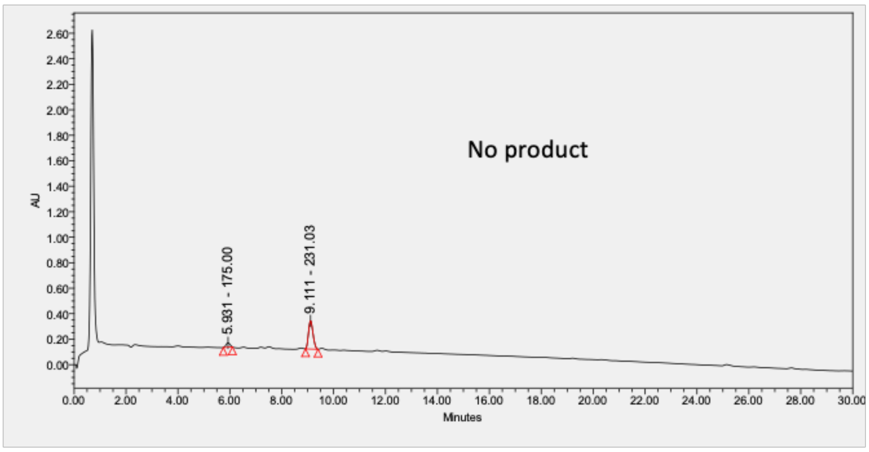

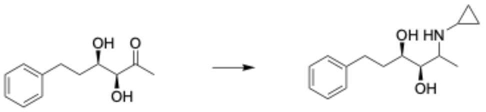

4a **4ai**

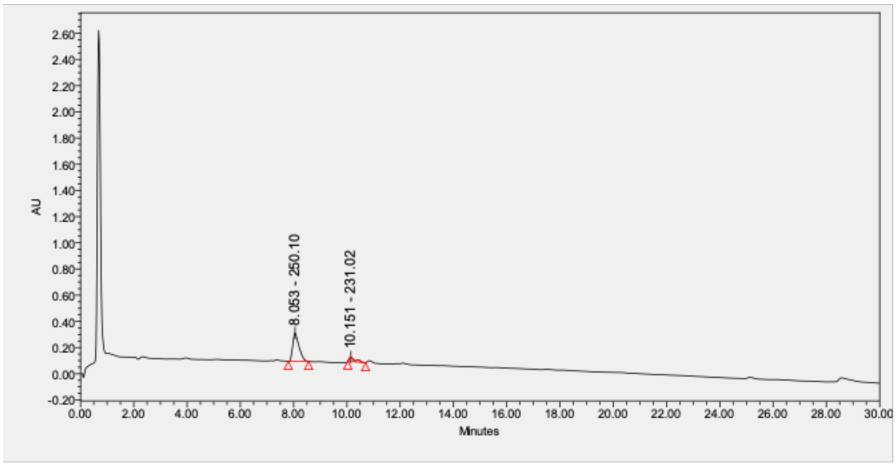

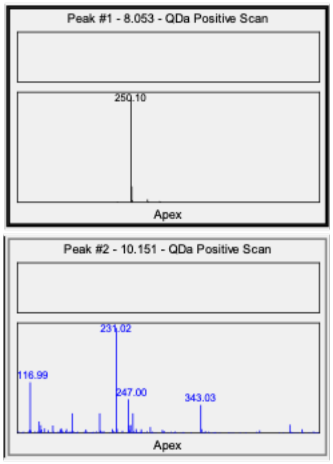

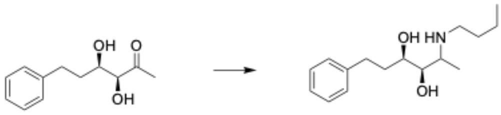

4a **4aii**

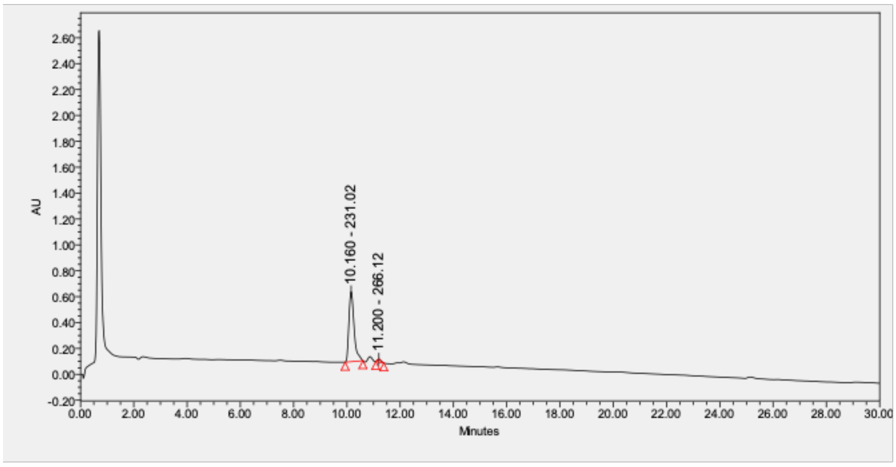

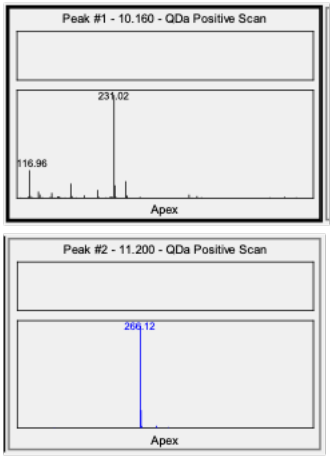

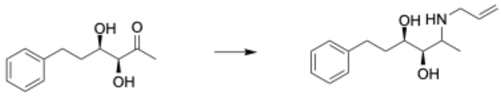

4a **4aiii**

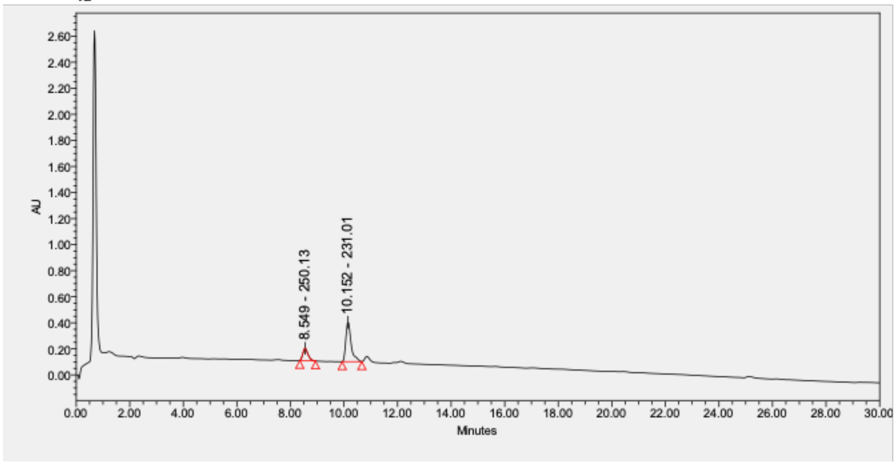

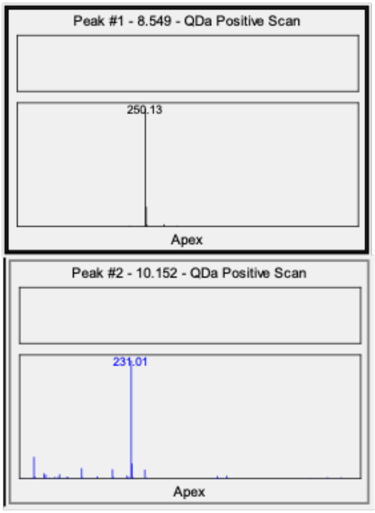

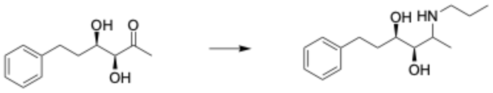

4a **4avi**

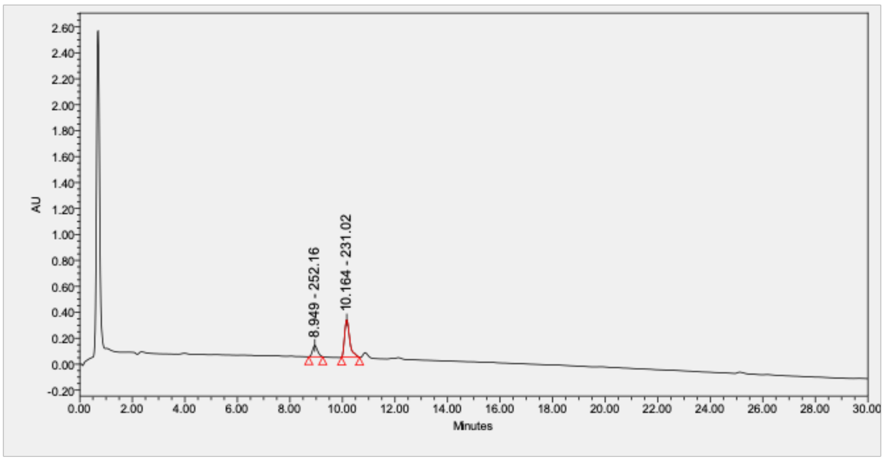

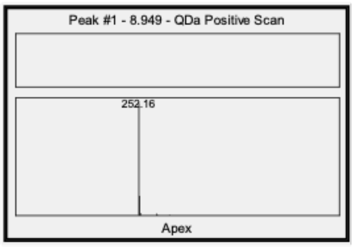

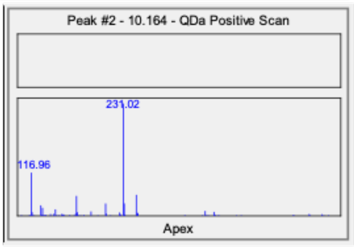

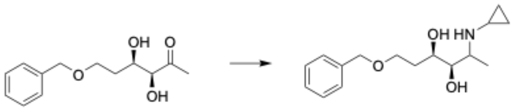

5a **5ai**

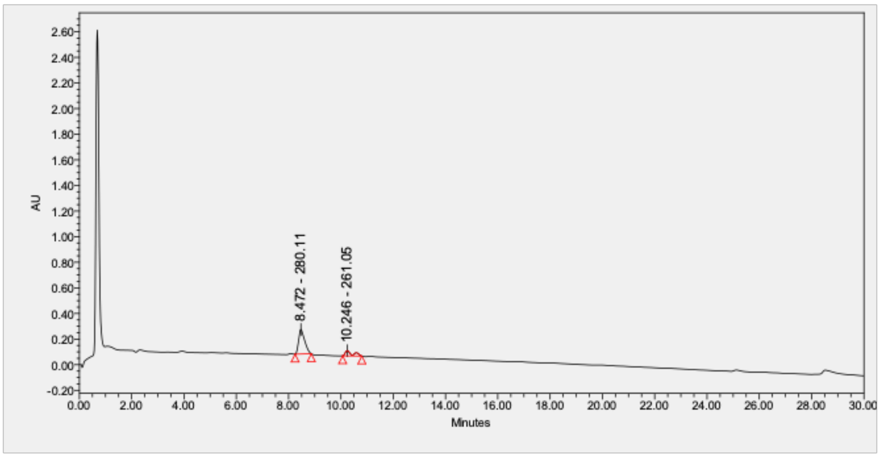

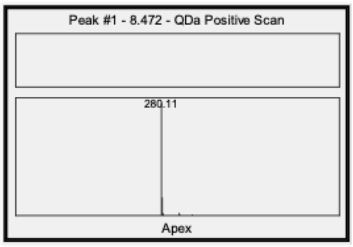

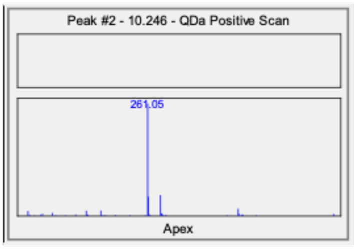

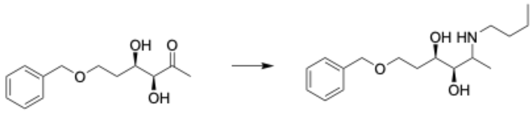

5a **5aii**

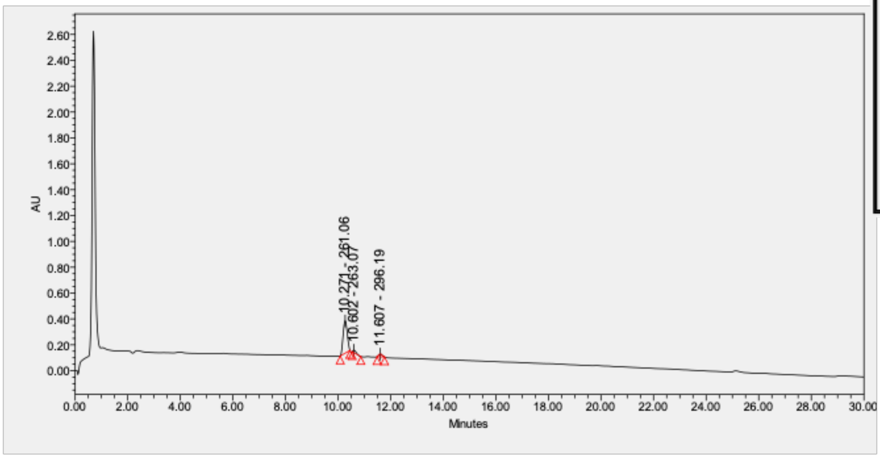

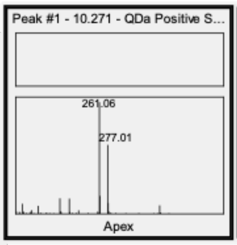

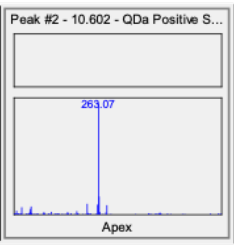

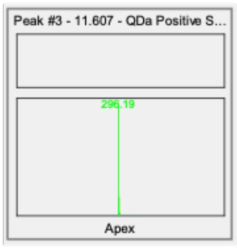

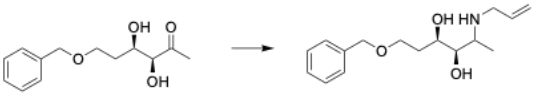

5a **5aiii**

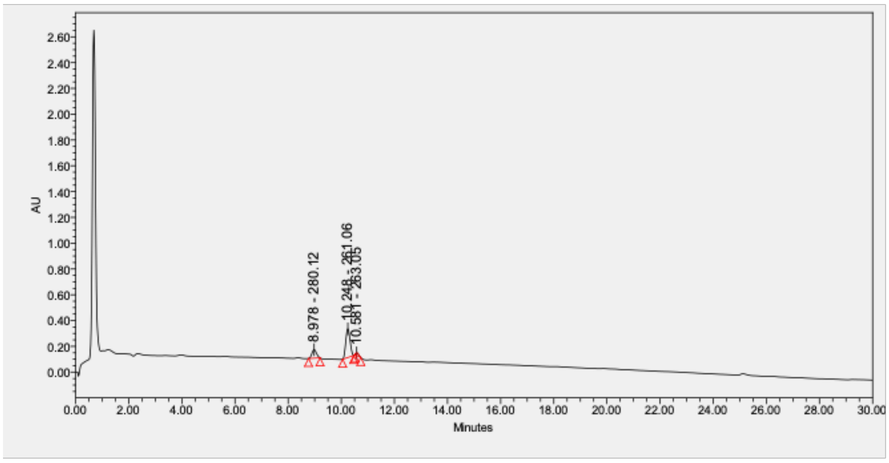

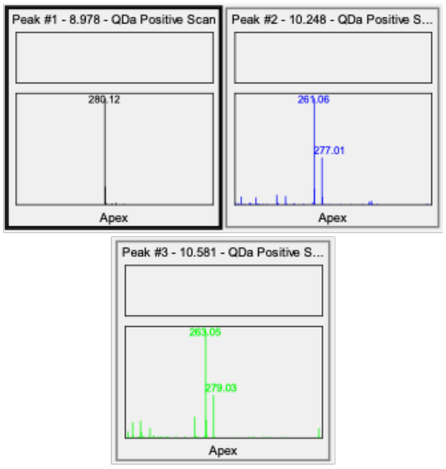

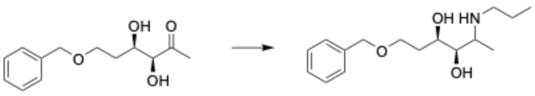

5a **5avi**

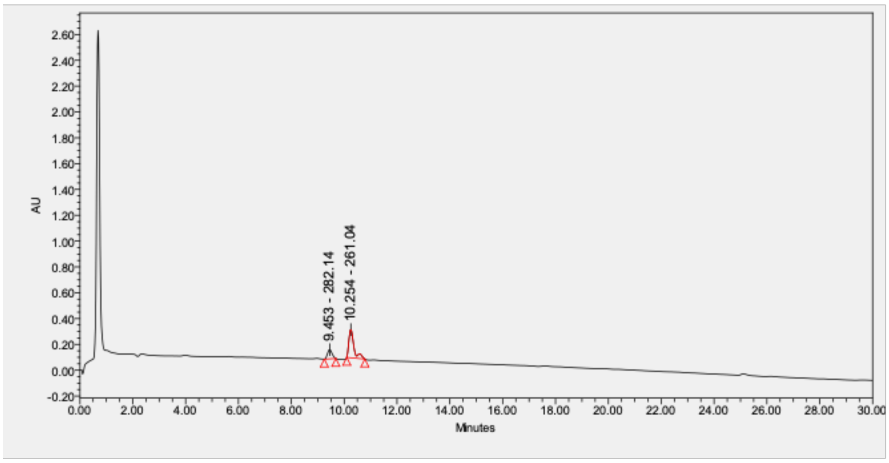

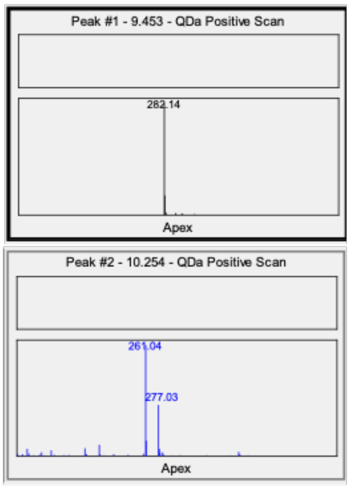

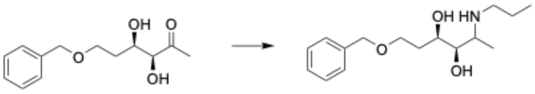

5a **5avi**

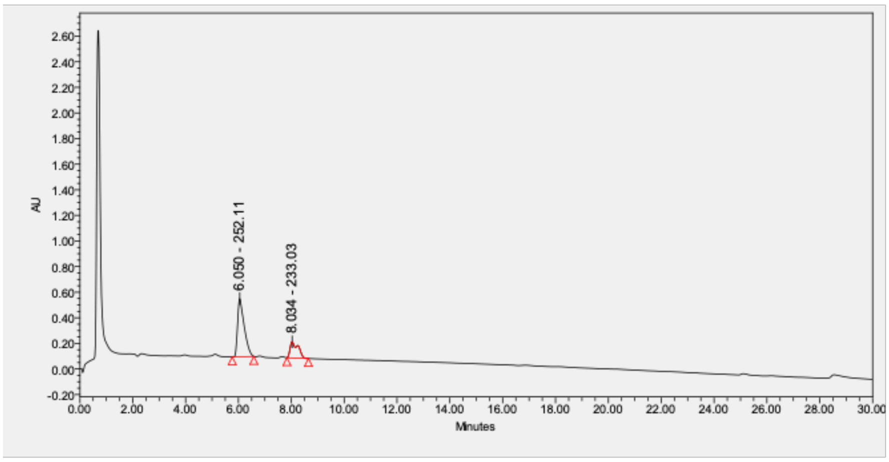

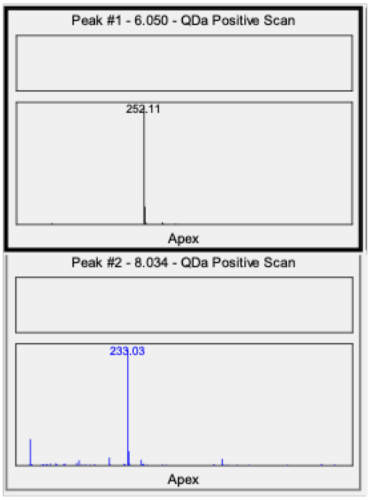

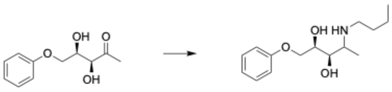

6a **6aii**

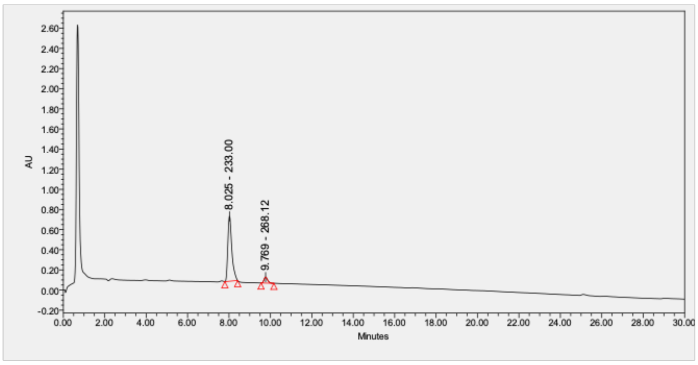

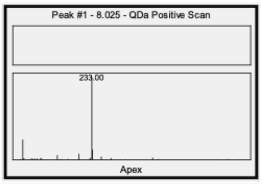

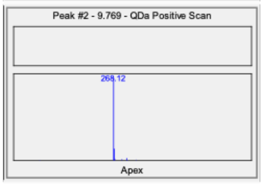

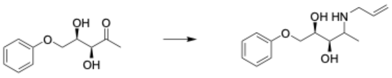

6a **6aiii**

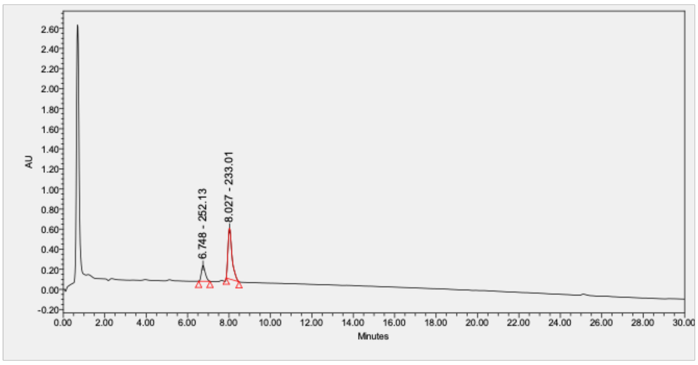

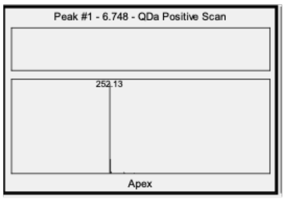

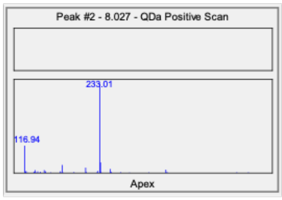

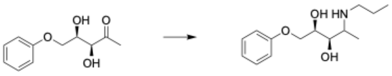

6a **6avi**

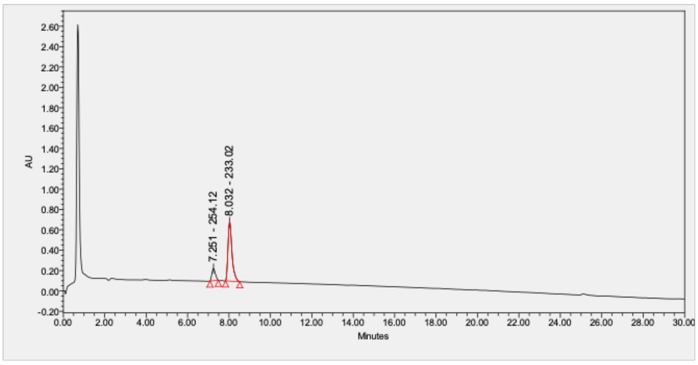

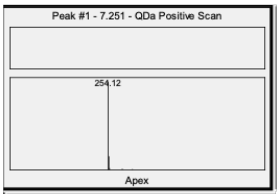

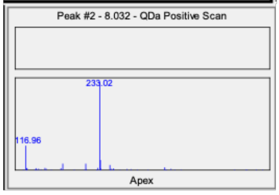

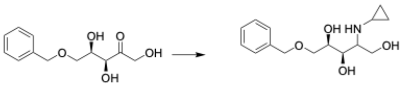

1b **1bi**

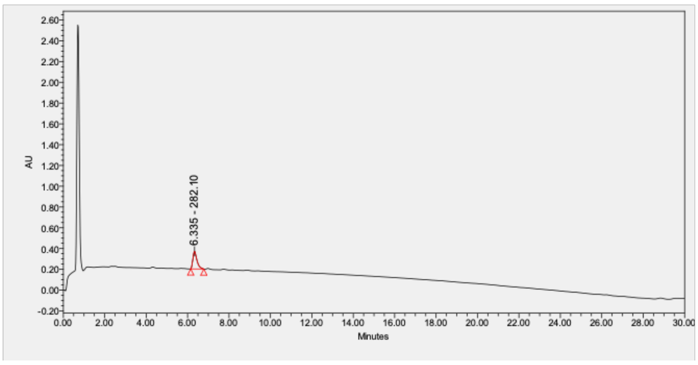

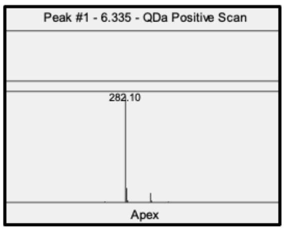

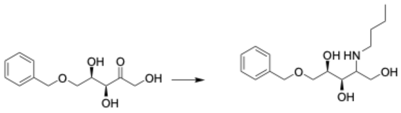

1b **1bii**

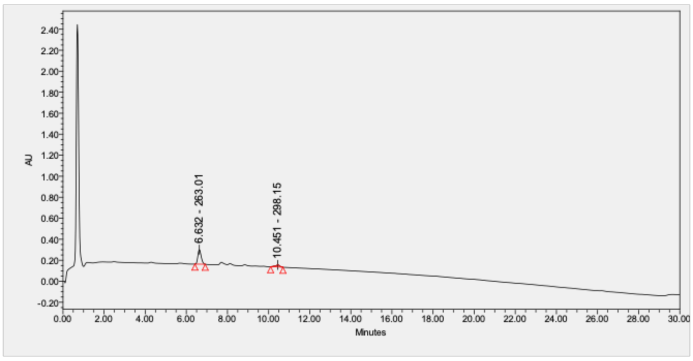

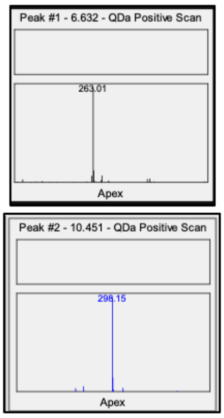

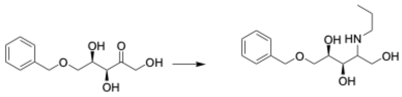

1b **1bvi**

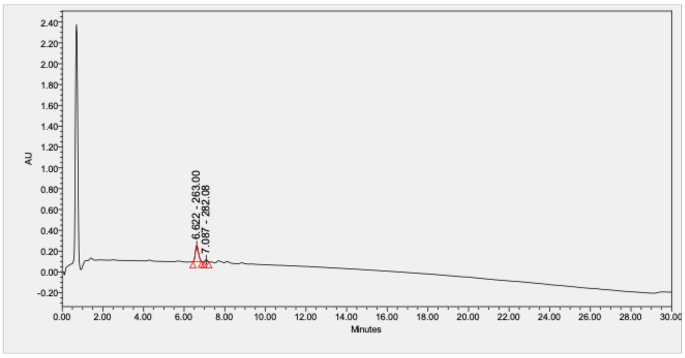

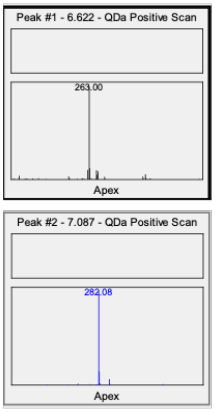

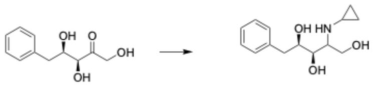

2b **2bi**

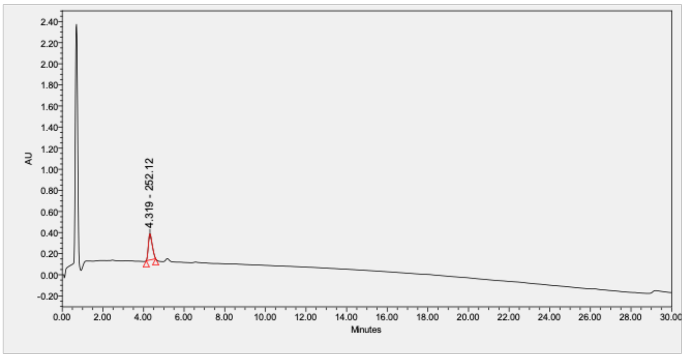

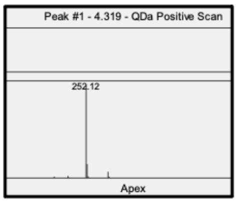

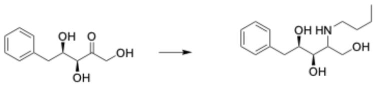

2bii

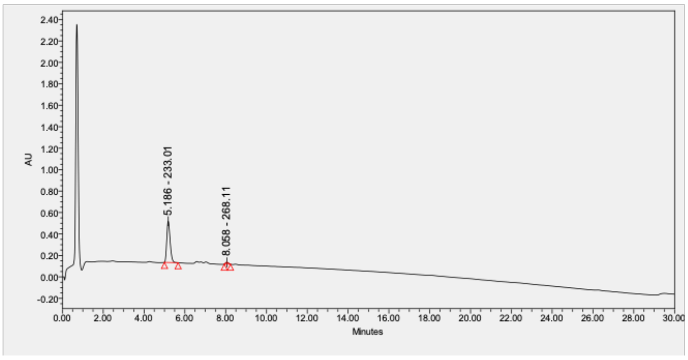

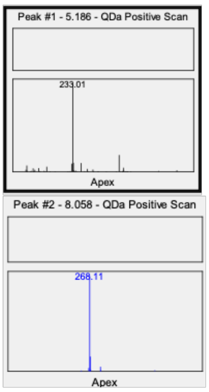

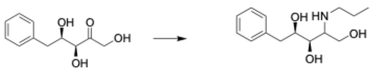

2b 2bvi

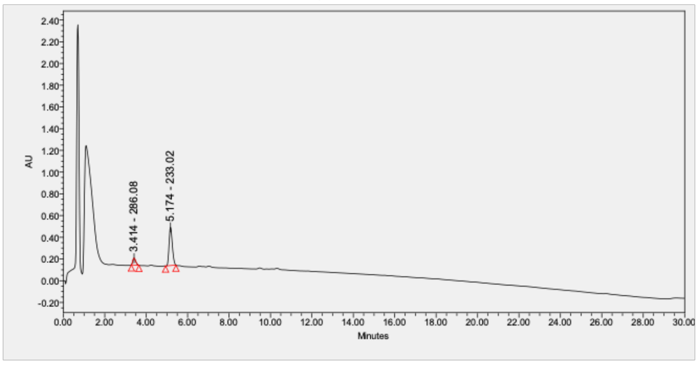

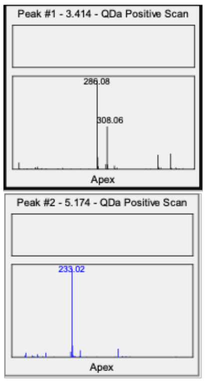

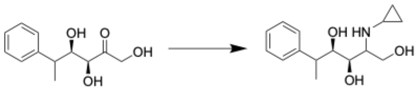

3b 3bi

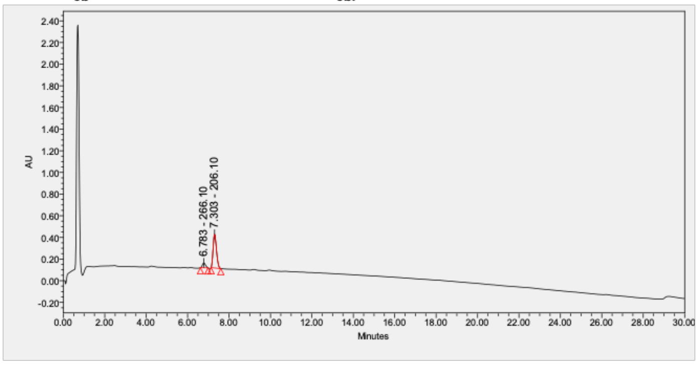

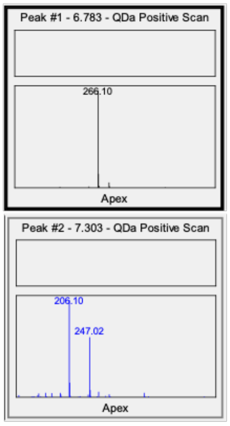

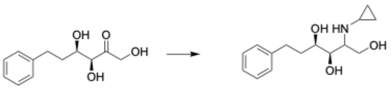

4b 4bi

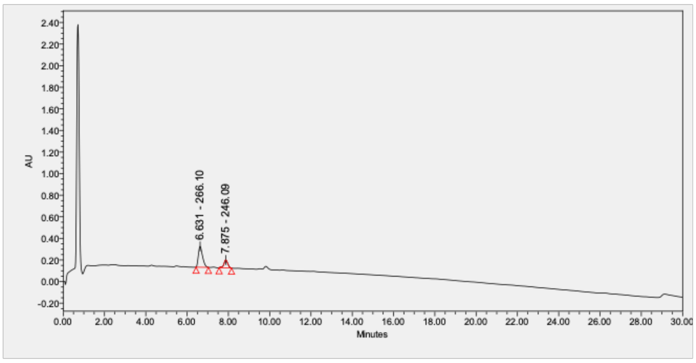

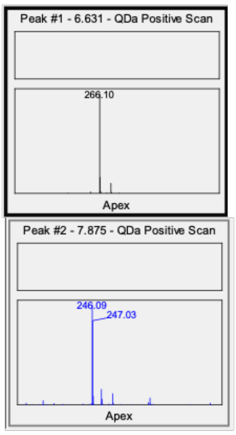

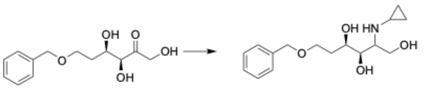

5b 5bi

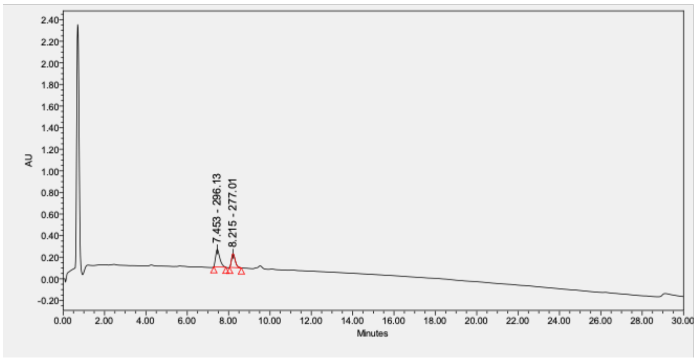

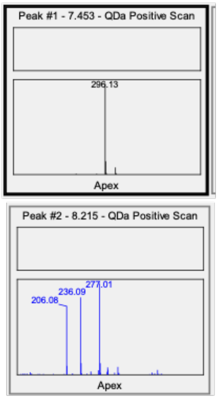

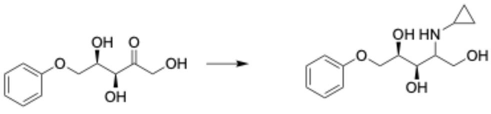

6b **6bi**

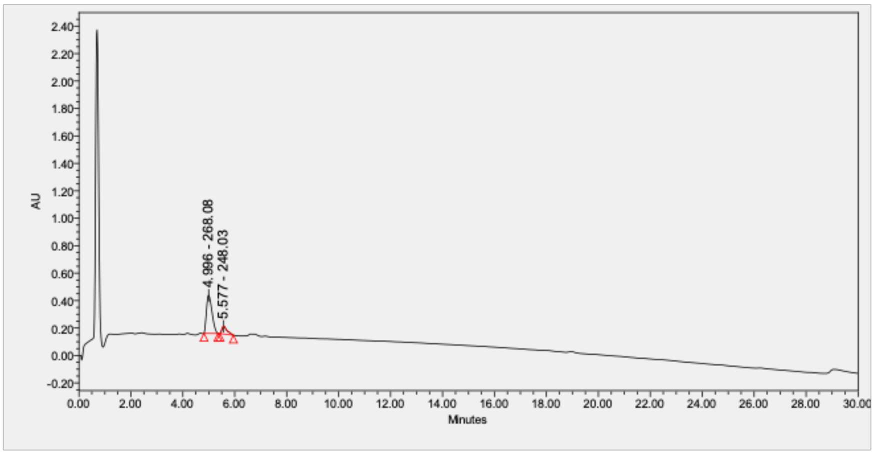

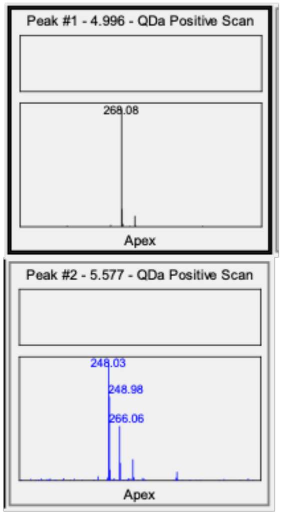

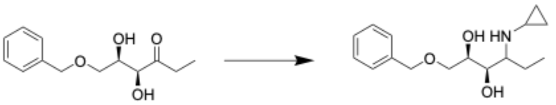

1c **1ci**

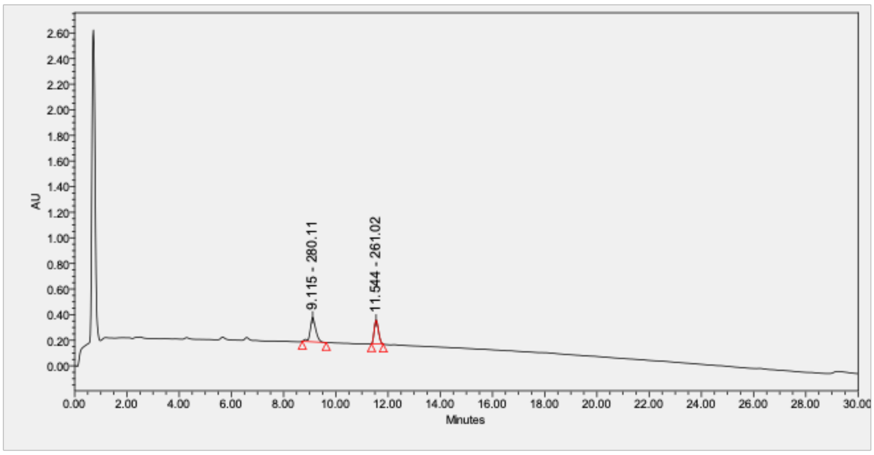

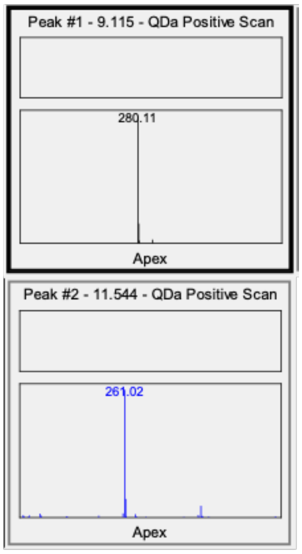

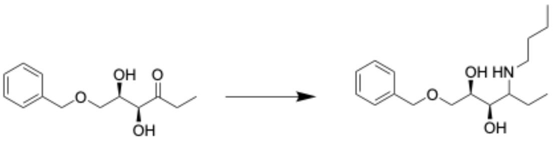

1c **1cii**

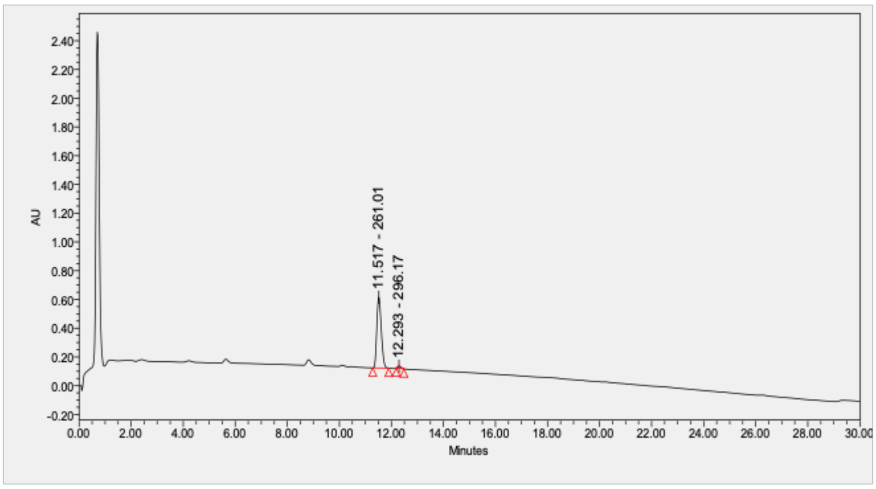

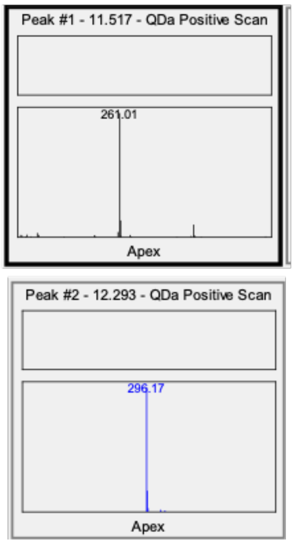

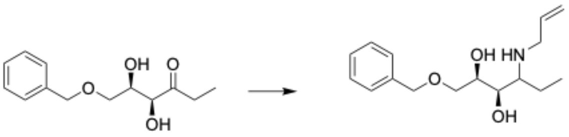

1c **1ciii**

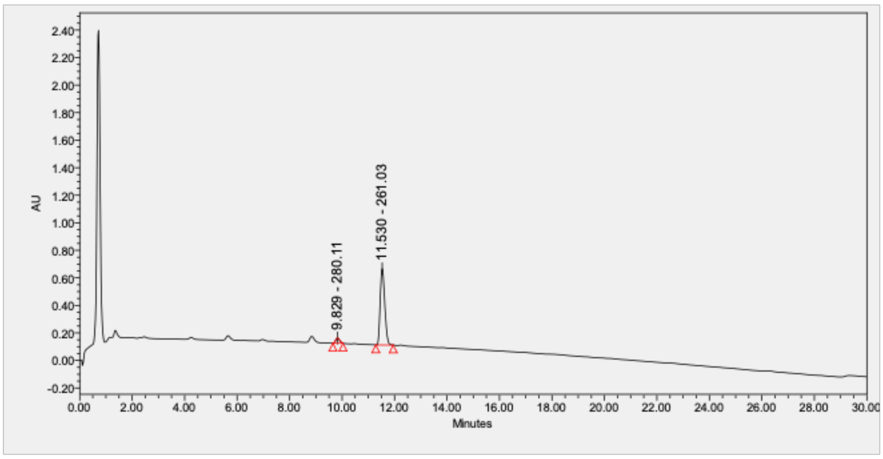

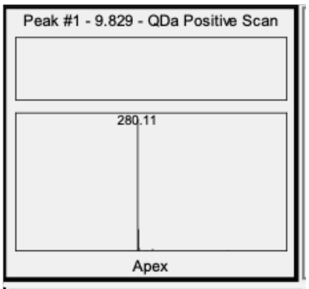

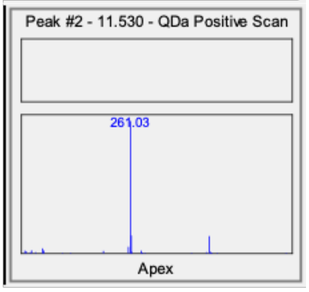

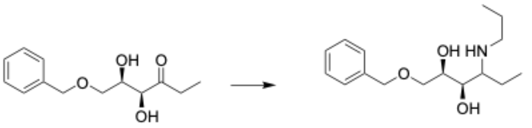

1c **1cvi**

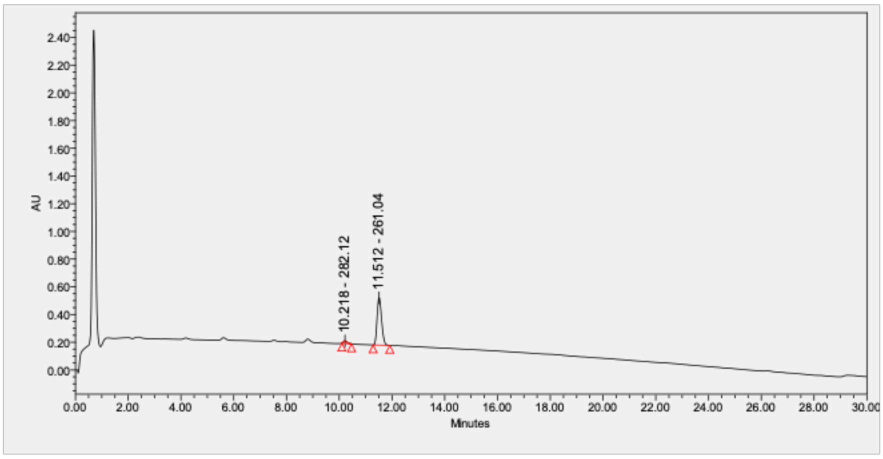

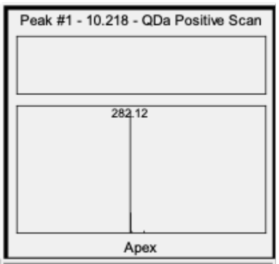

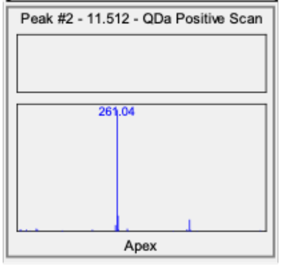

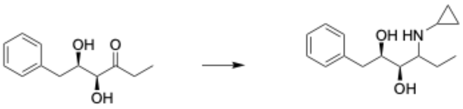

2c **2ci**

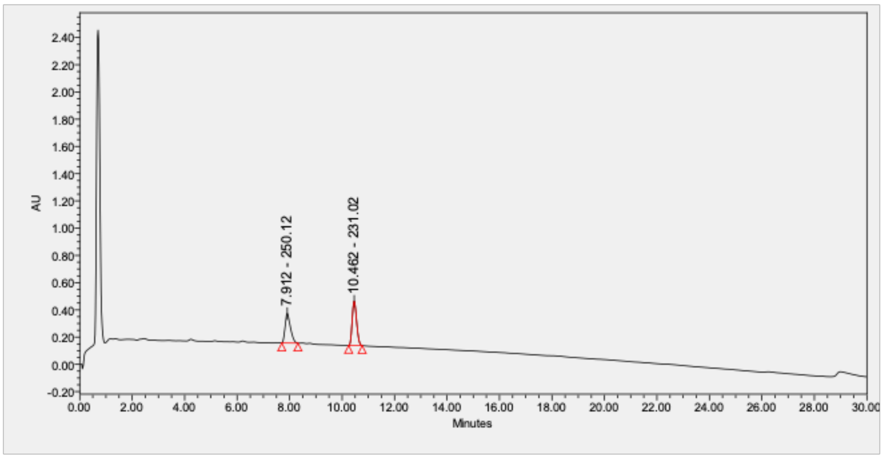

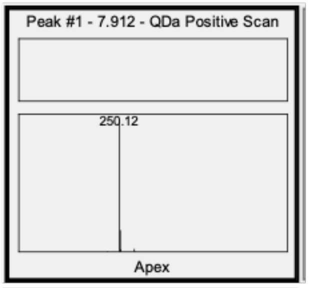

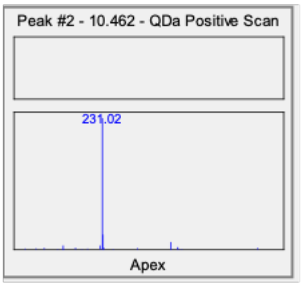

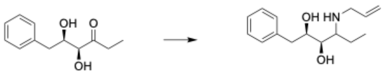

2c 2ciii

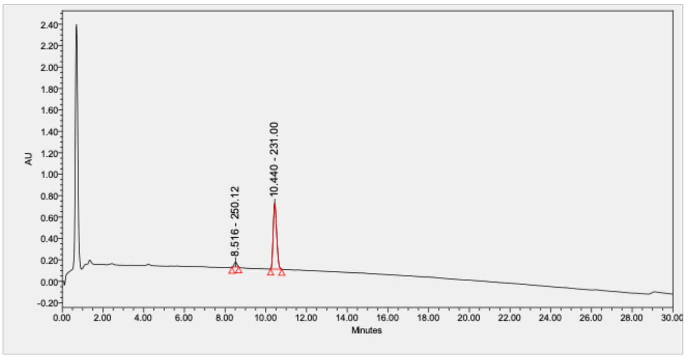

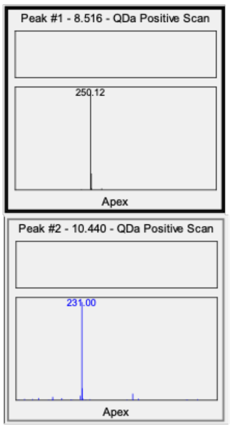

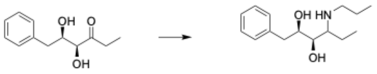

2c 2cvi

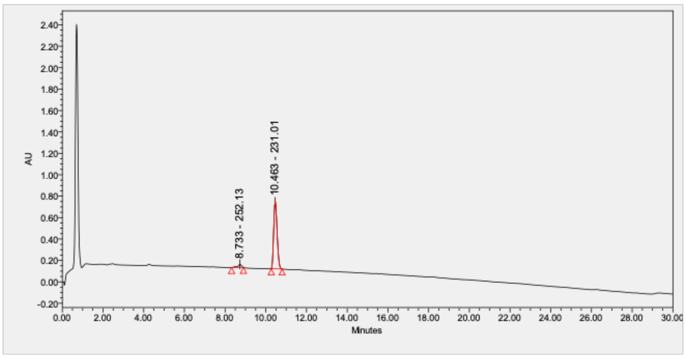

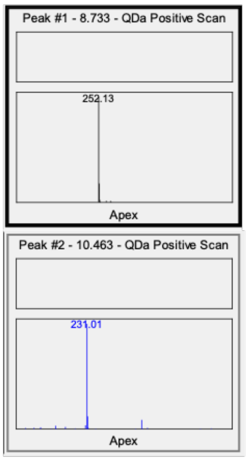

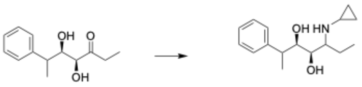

3c 3ci

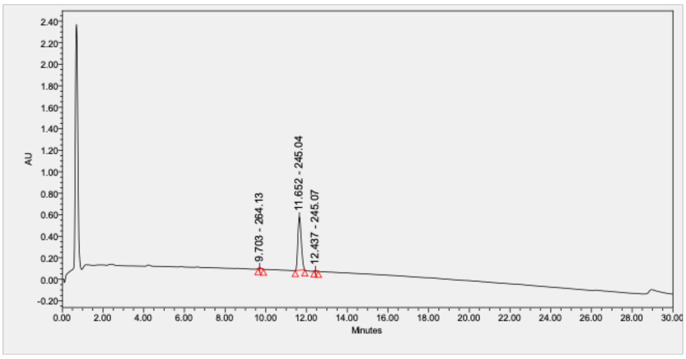

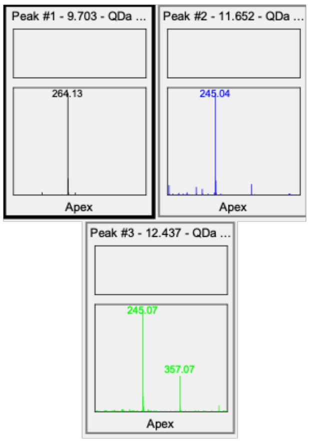

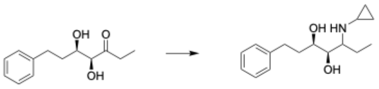

4c 4ci

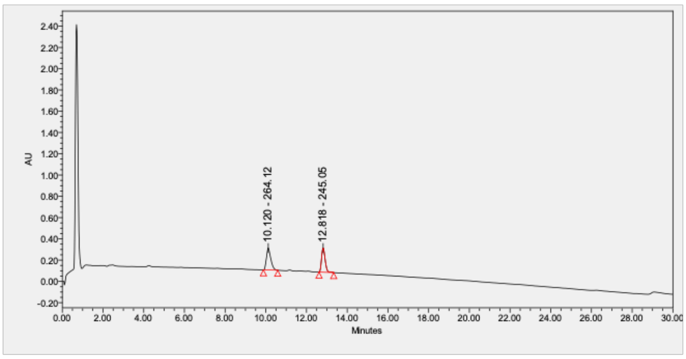

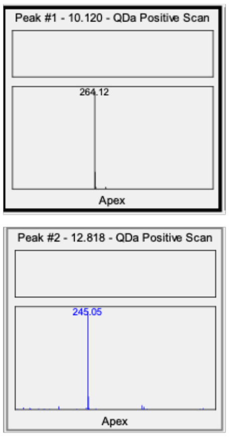

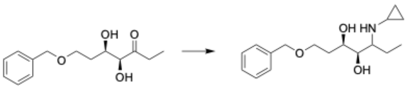

5c 5ci

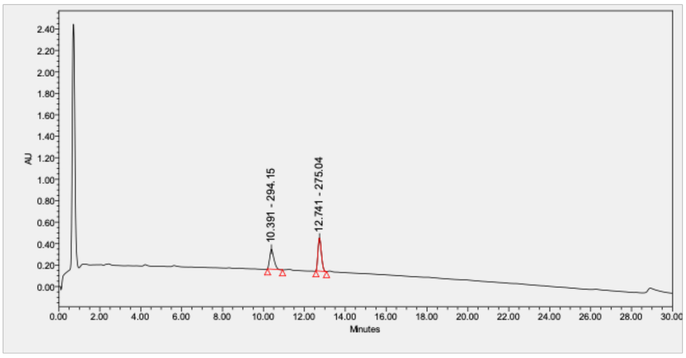

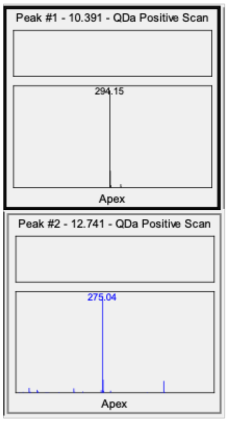

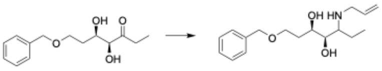

5c 5ciii

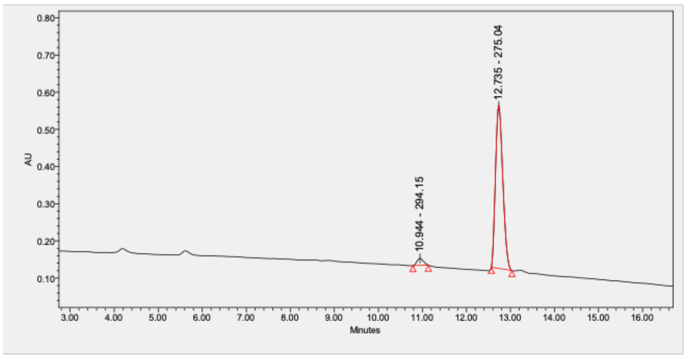

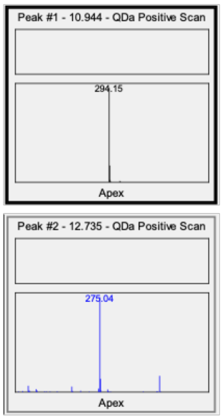

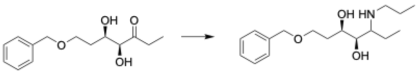

5c 5cvi

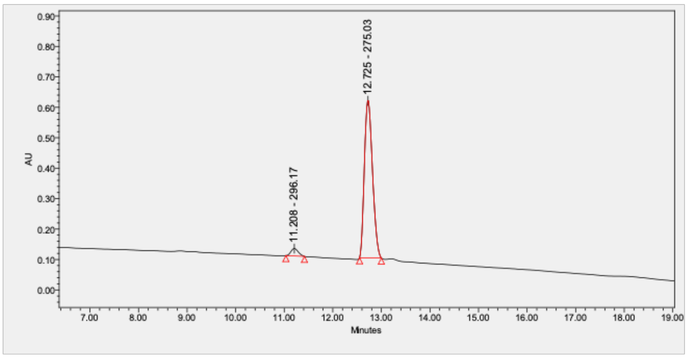

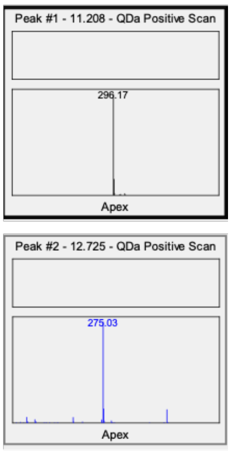

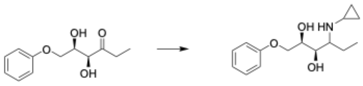

6c 6ci

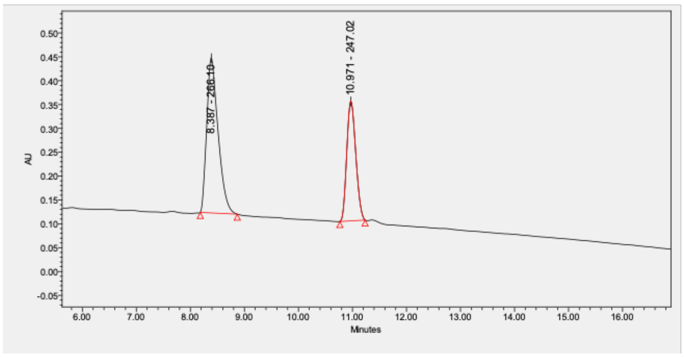

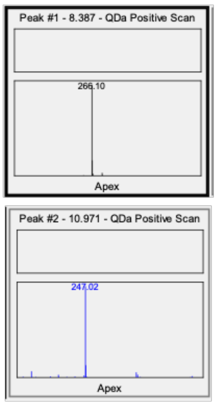

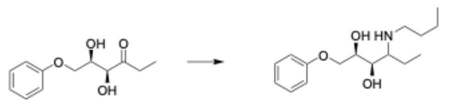

6c 6cii

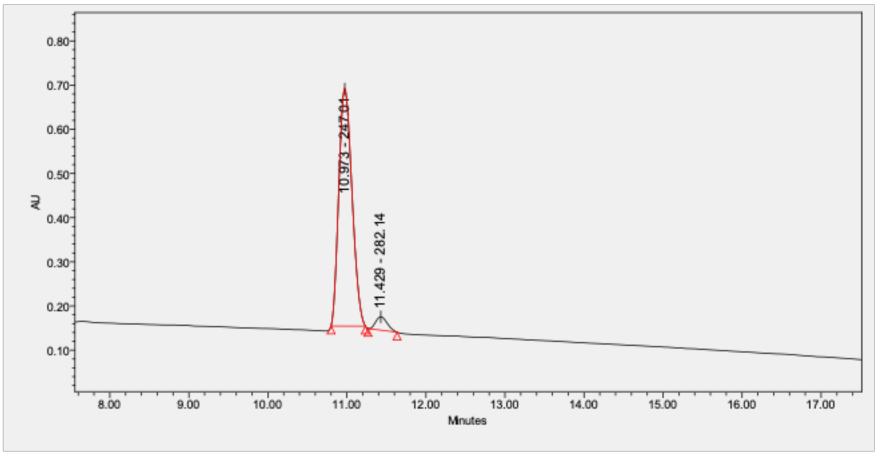

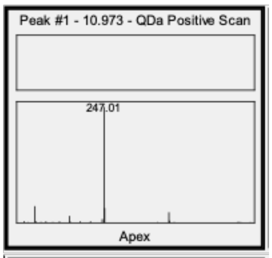

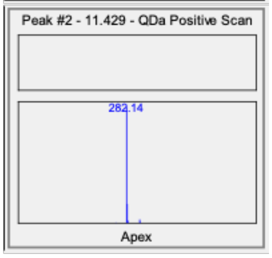

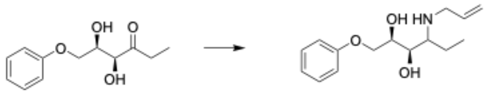

6c 6ciii

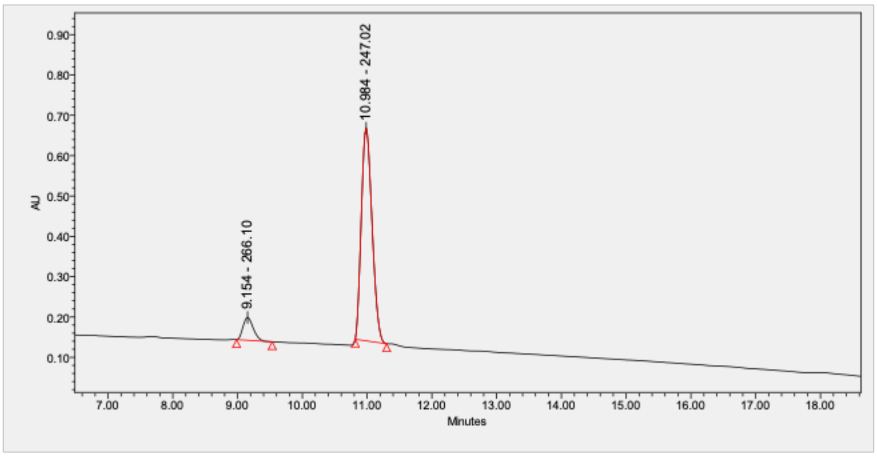

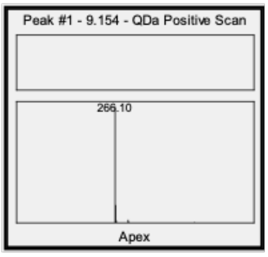

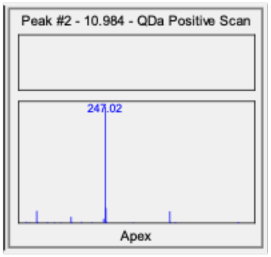

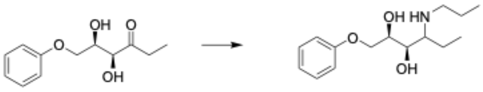

6c 6cvi

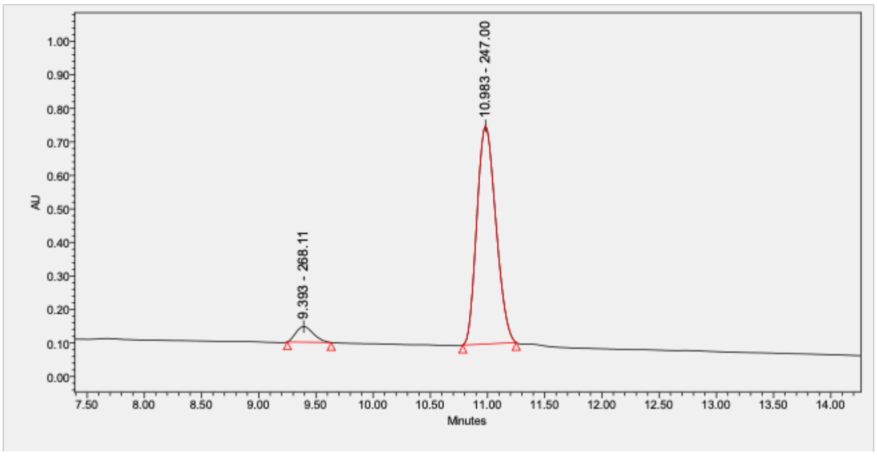

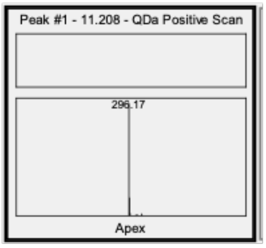

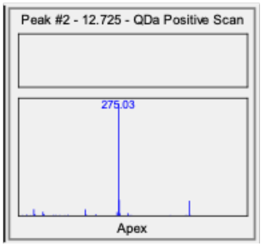

Example Chromatograms of one-pot two-step FSA-IR259 cascade. First step (FSA step only)

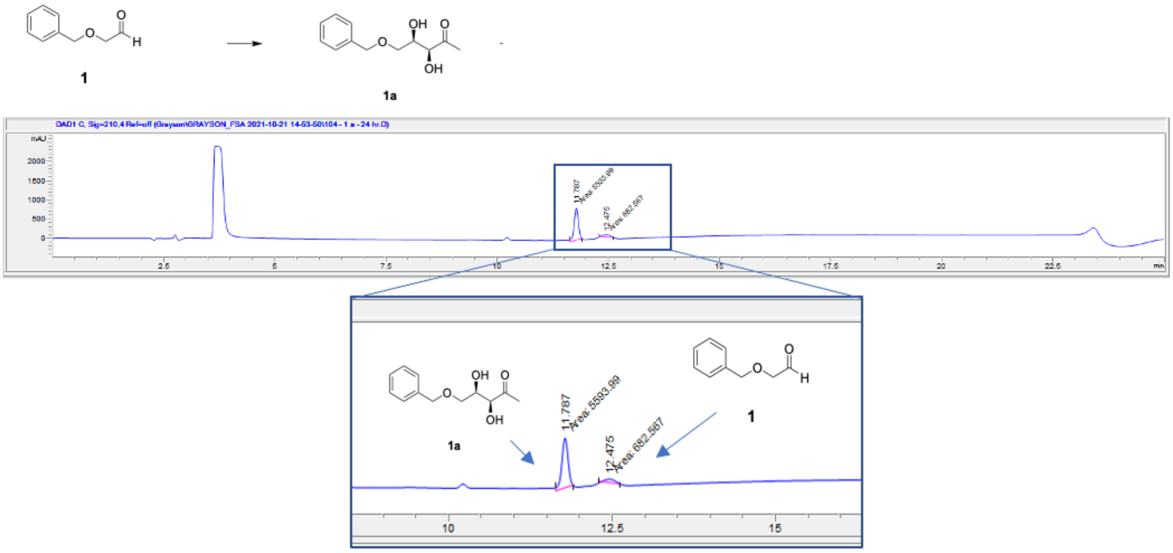

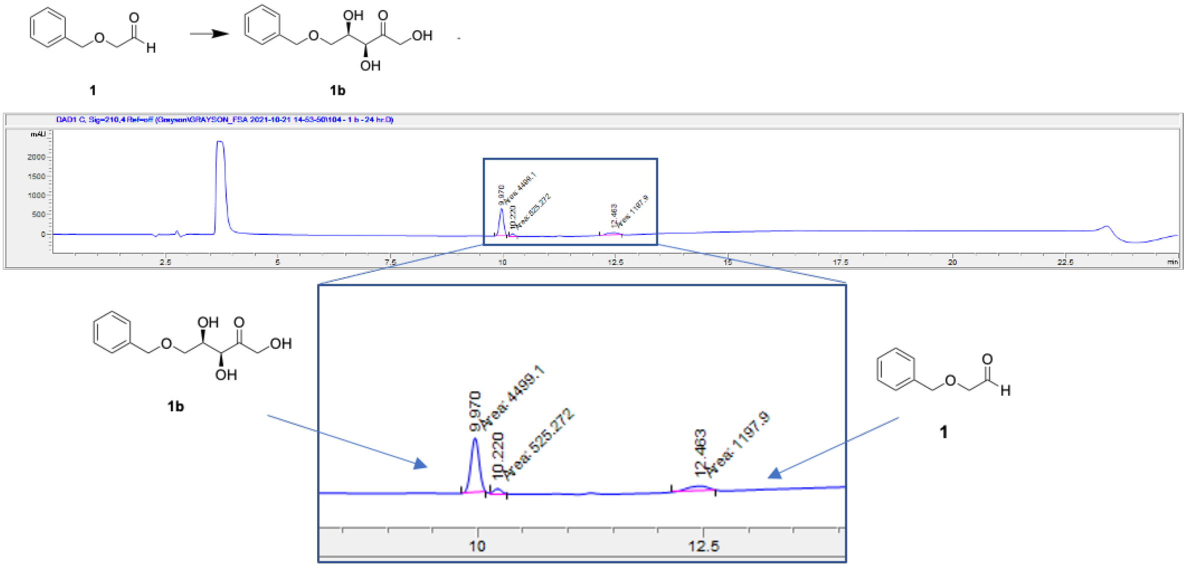

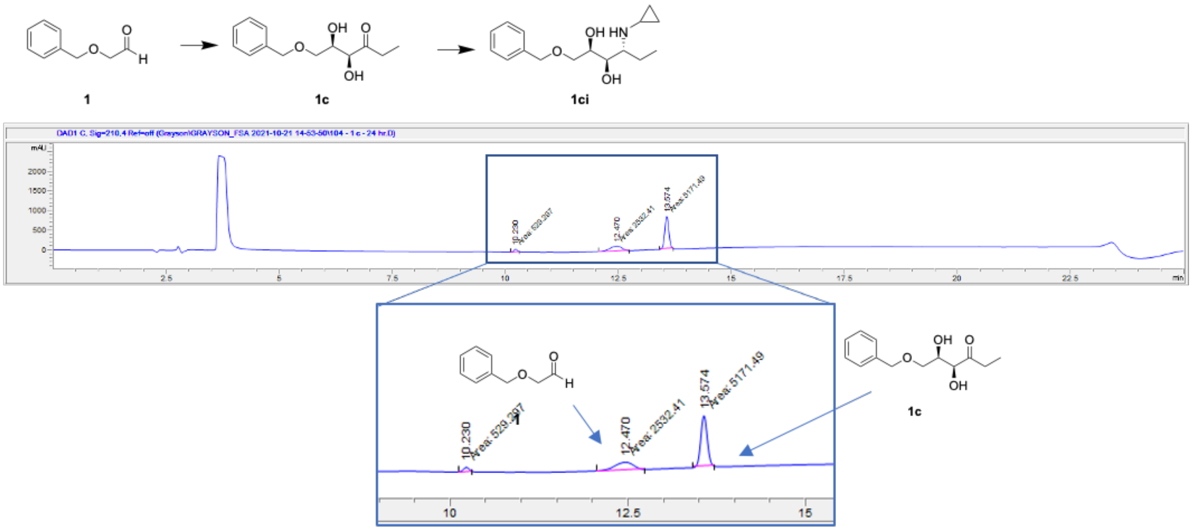

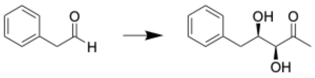

2 2a

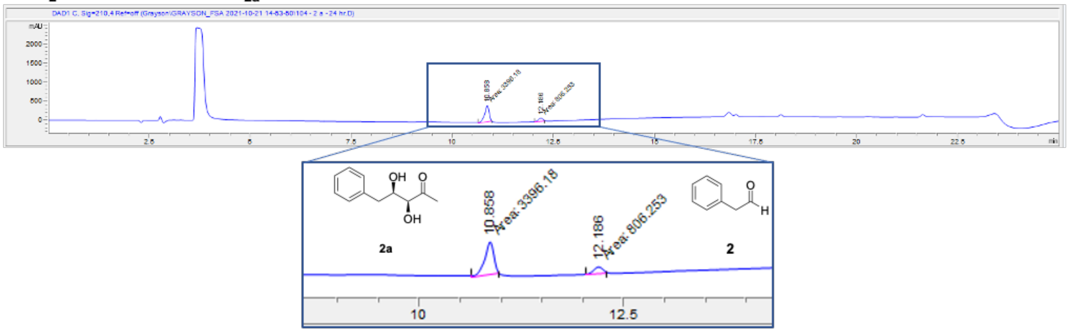

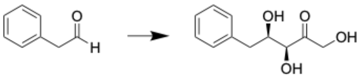

2 2b

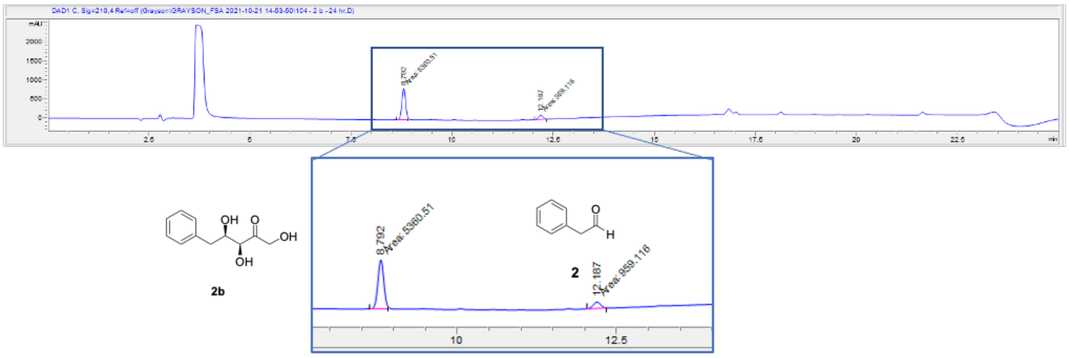

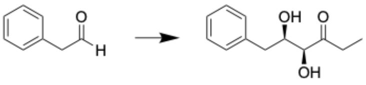

2 2c

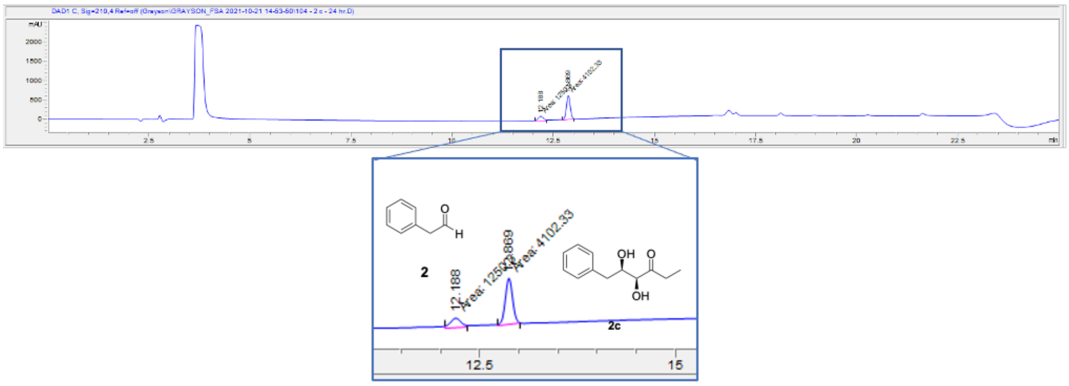

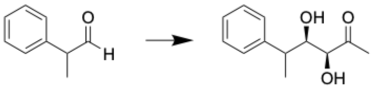

3 3a

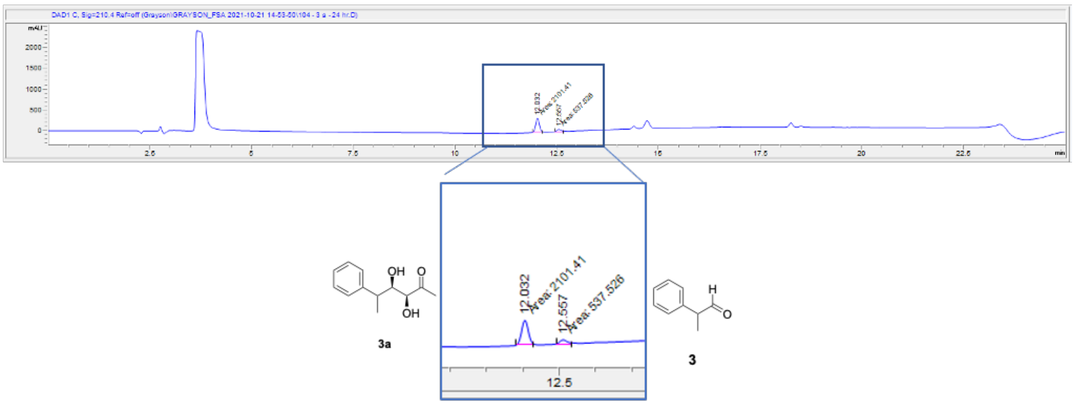

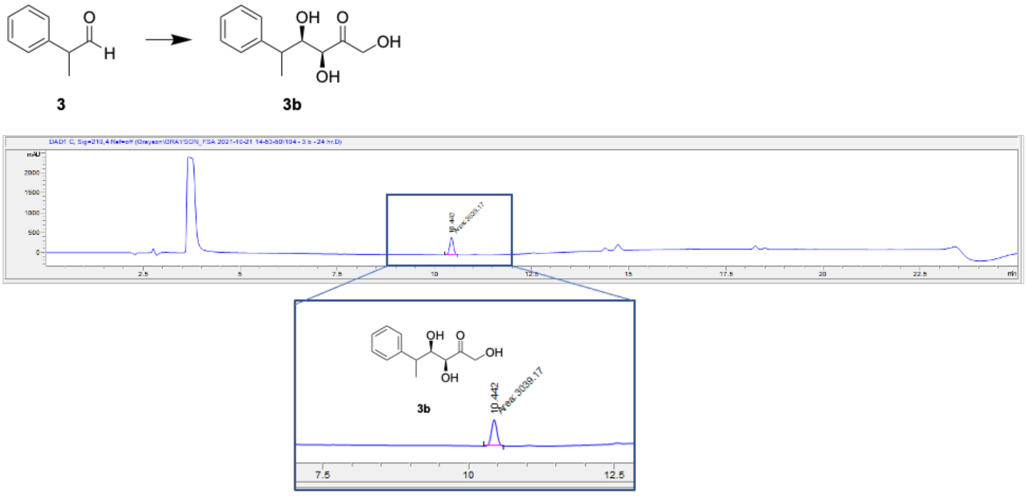
3
3b
10.442
Area: 3039.17
7.5
10
12.5

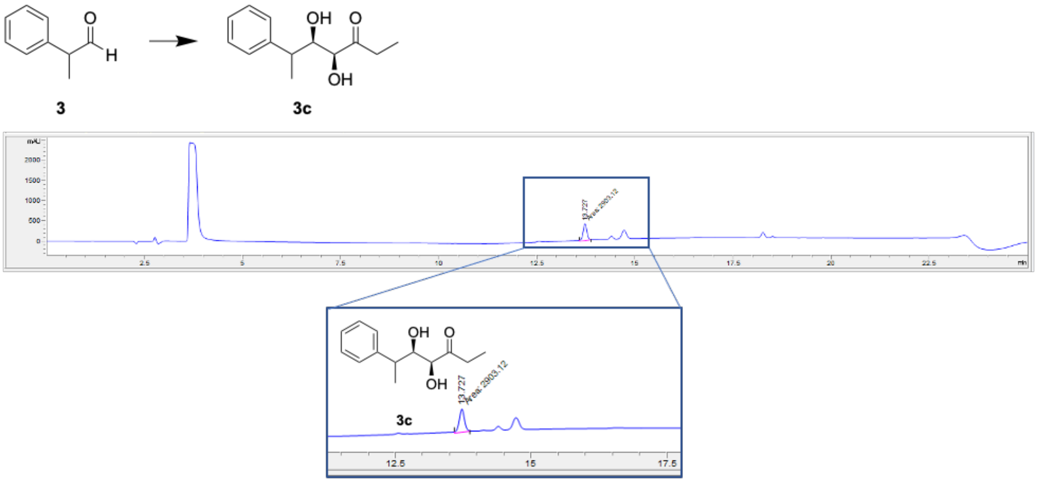
3
3c
13.727
Area: 2903.12
12.5
15
17.5

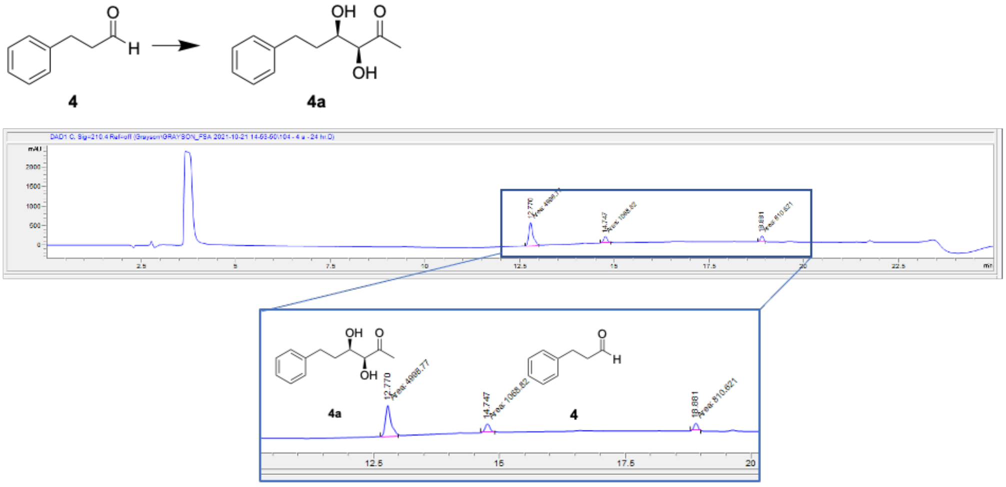
4
4a
12.770
Area: 4988.77
14.747
Area: 1068.62
18.881
Area: 810.621
12.5
15
17.5
20

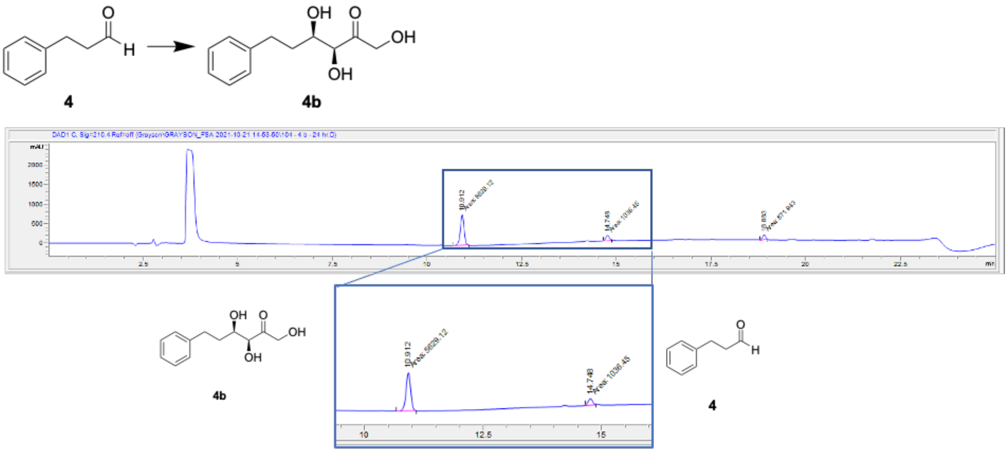
4
4b
10.912
Area: 5609.12
14.749
Area: 1036.45
10
12.5
15

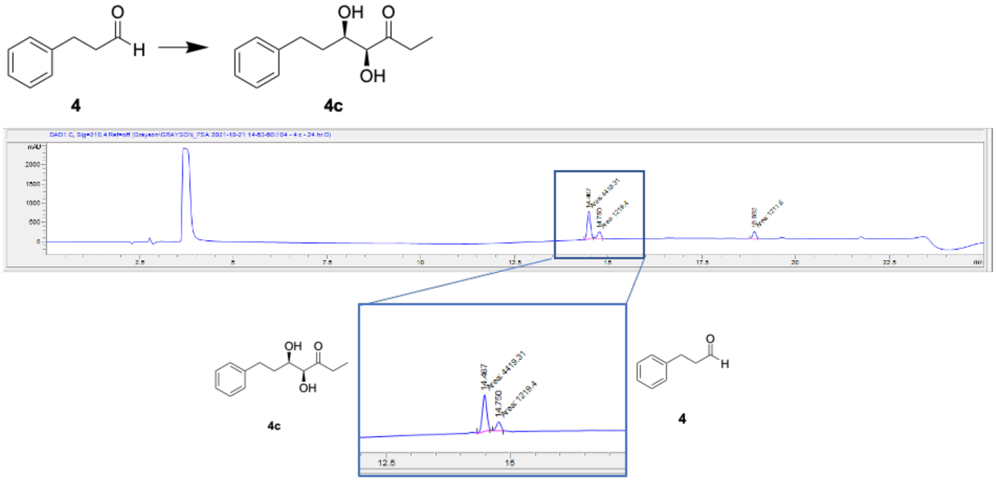
4
4c
14.467
14.750
Area: 4419.31
Area: 1219.4
12.5
15

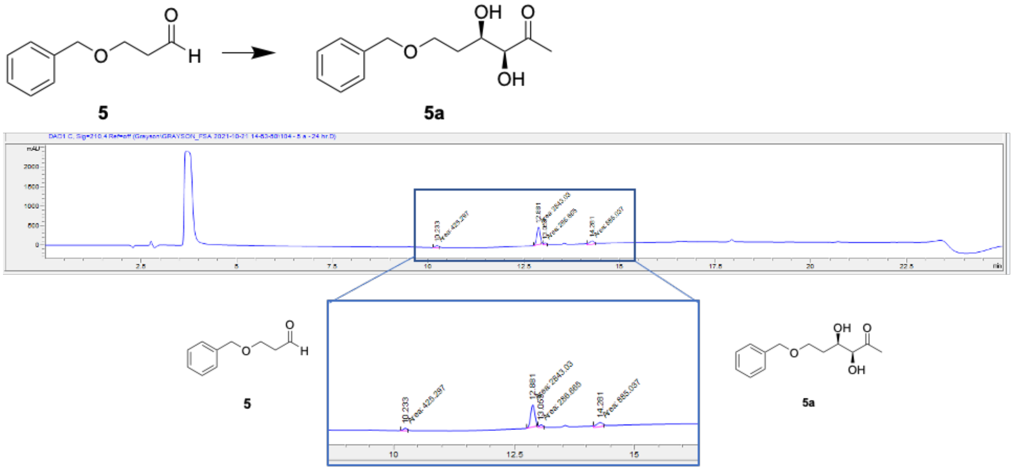
5
5a
10.233
12.881
14.281
10
12.5
15

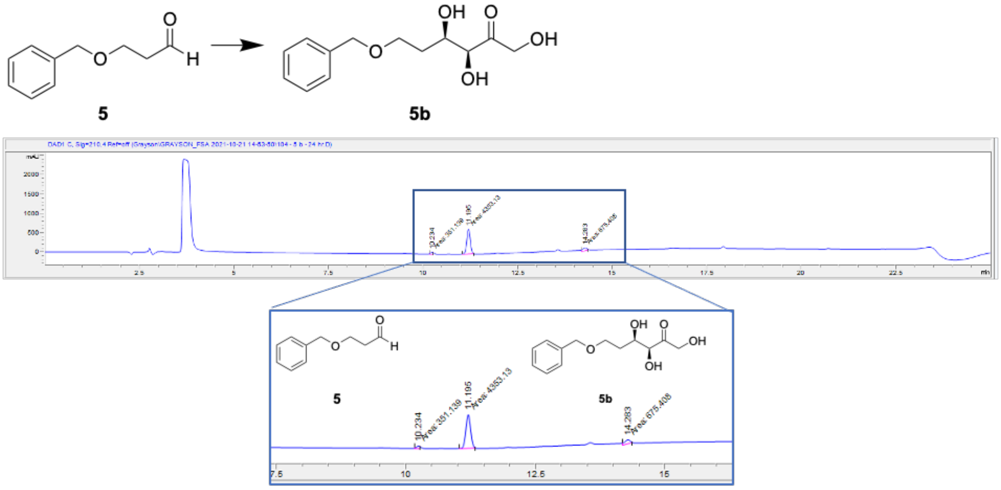
5
5b
10.234
11.195
14.283
Area: 4353.13
Area: 678.408
7.5
10
12.5
15

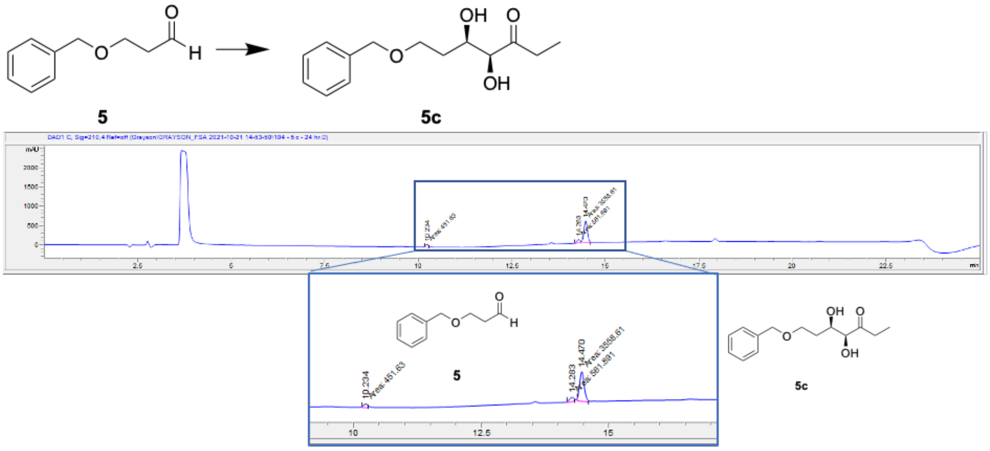
5
5c
10.234
14.283
14.470
Area: 451.63
Area: 3558.61
10
12.5
15

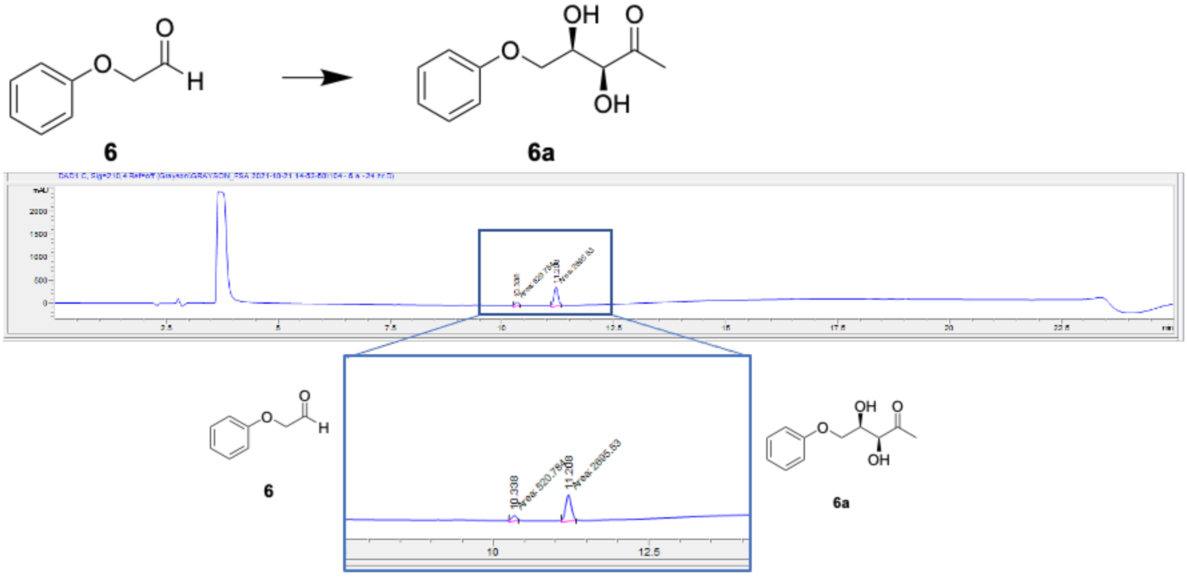
6
6a
10.338
Area: 520.784
11.208
Area: 2695.53
10
12.5

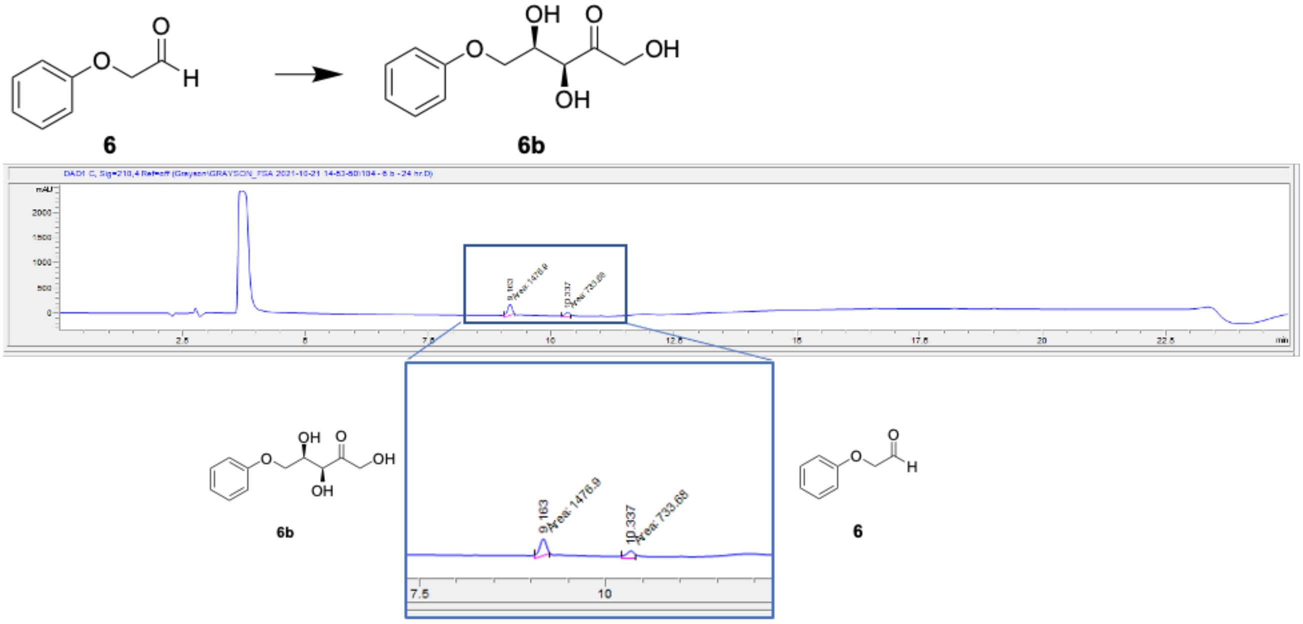
6
6b
9.163
Area: 1476.9
10.337
Area: 733.68
7.5
10

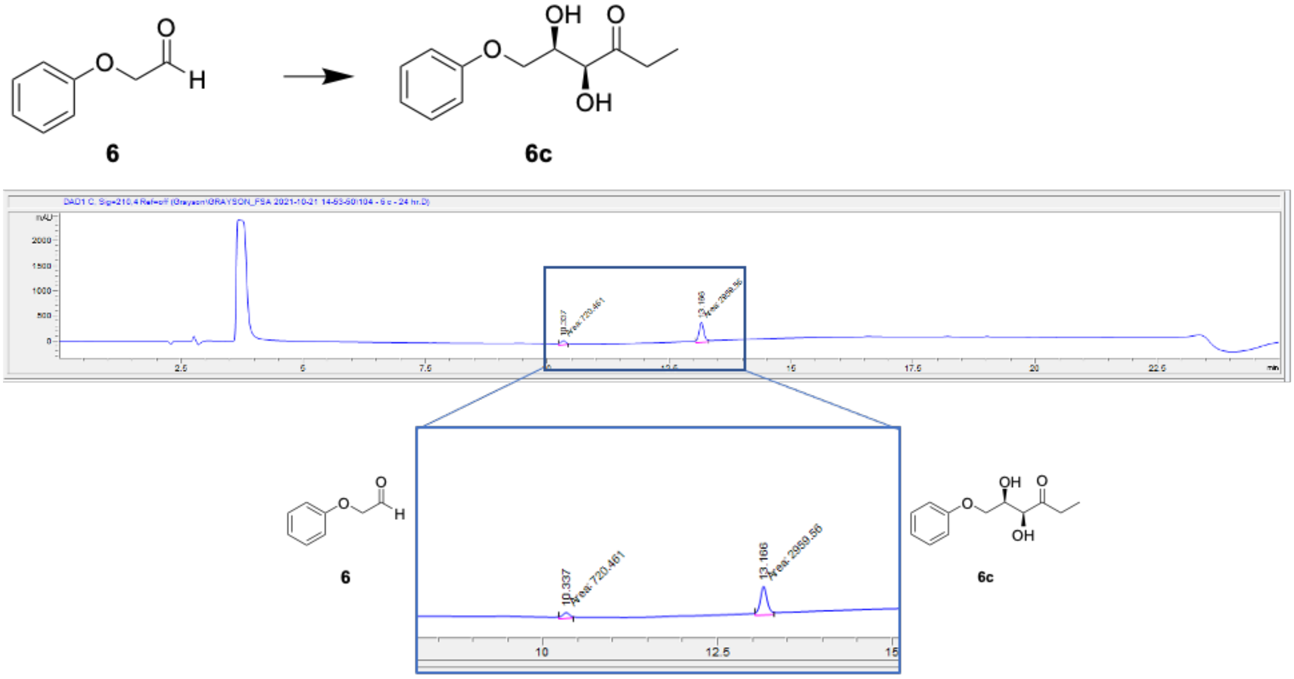
6
6c
10.337
Area: 720.461
13.166
Area: 2959.56
10
12.5

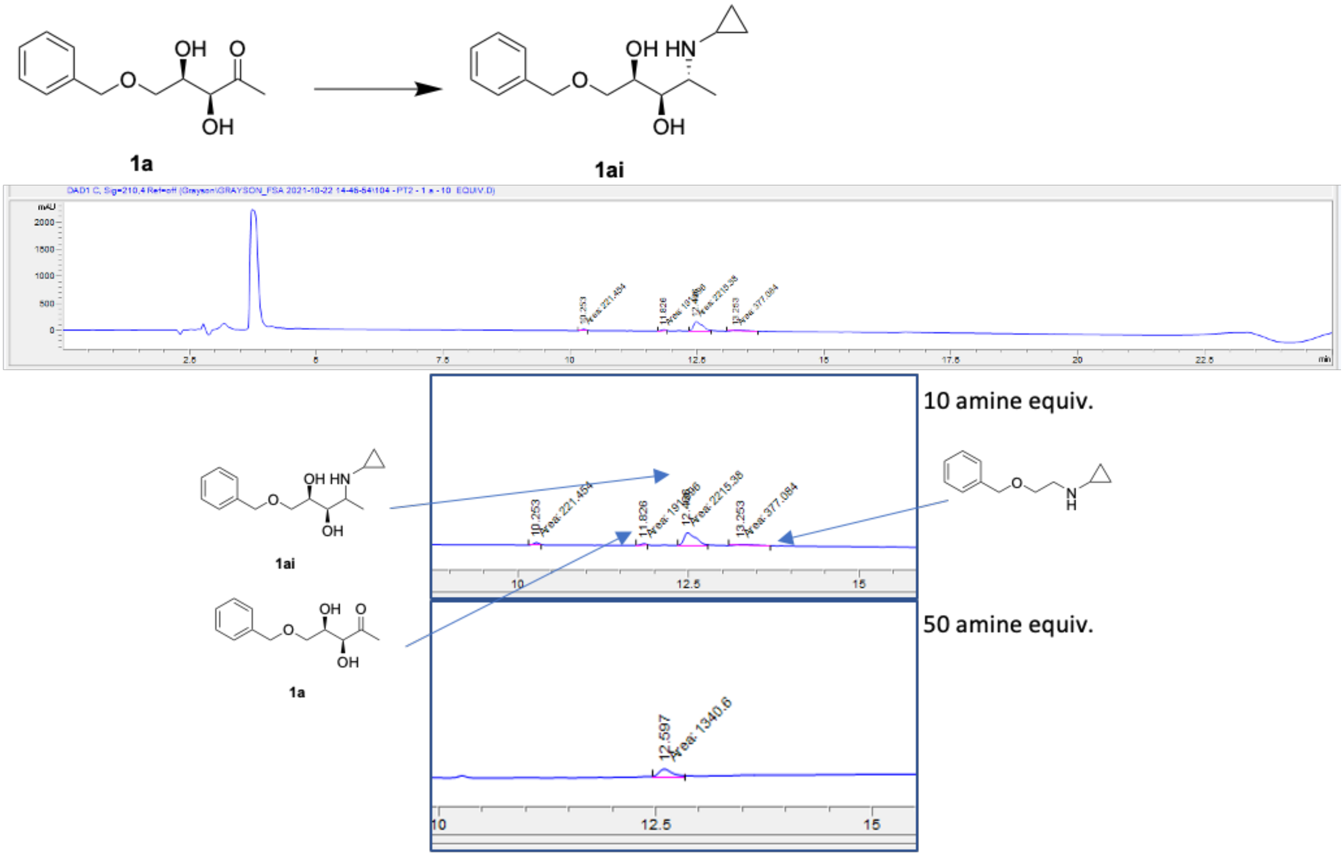
1a
1ai
10 amine equiv.
50 amine equiv.
10.253
Area: 221.454
11.826
12.597
Area: 2215.38
13.253
Area: 377.084
Area: 1340.6
10
12.5
15

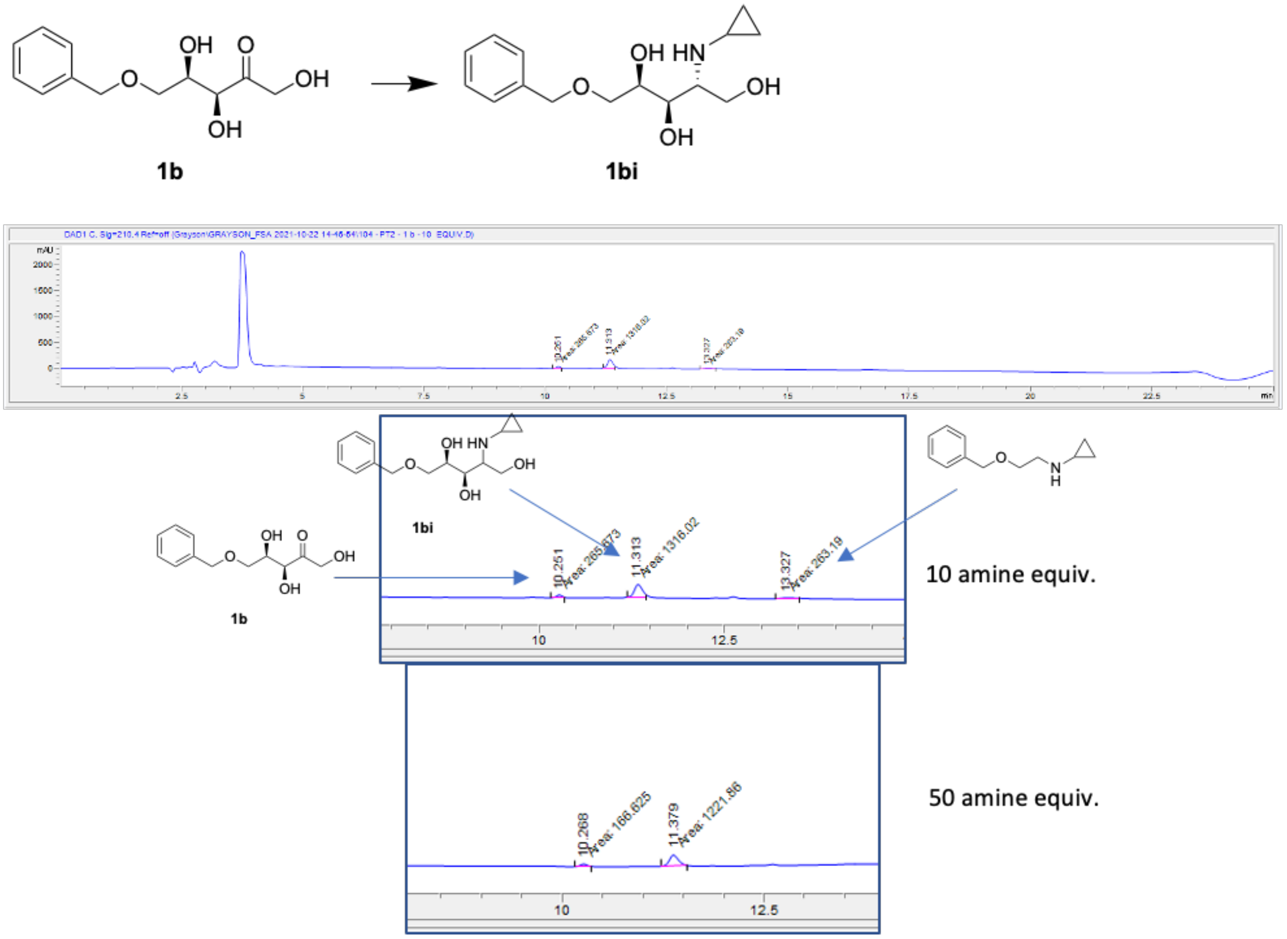
1b
1bi
10 amine equiv.
50 amine equiv.
10.251
Area: 265.673
11.313
Area: 1316.02
13.327
Area: 263.19
10.268
Area: 166.625
11.379
Area: 1221.86
10
12.5

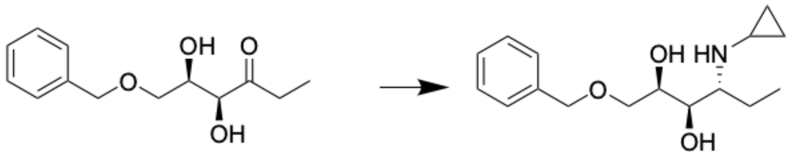

1c **1ci**

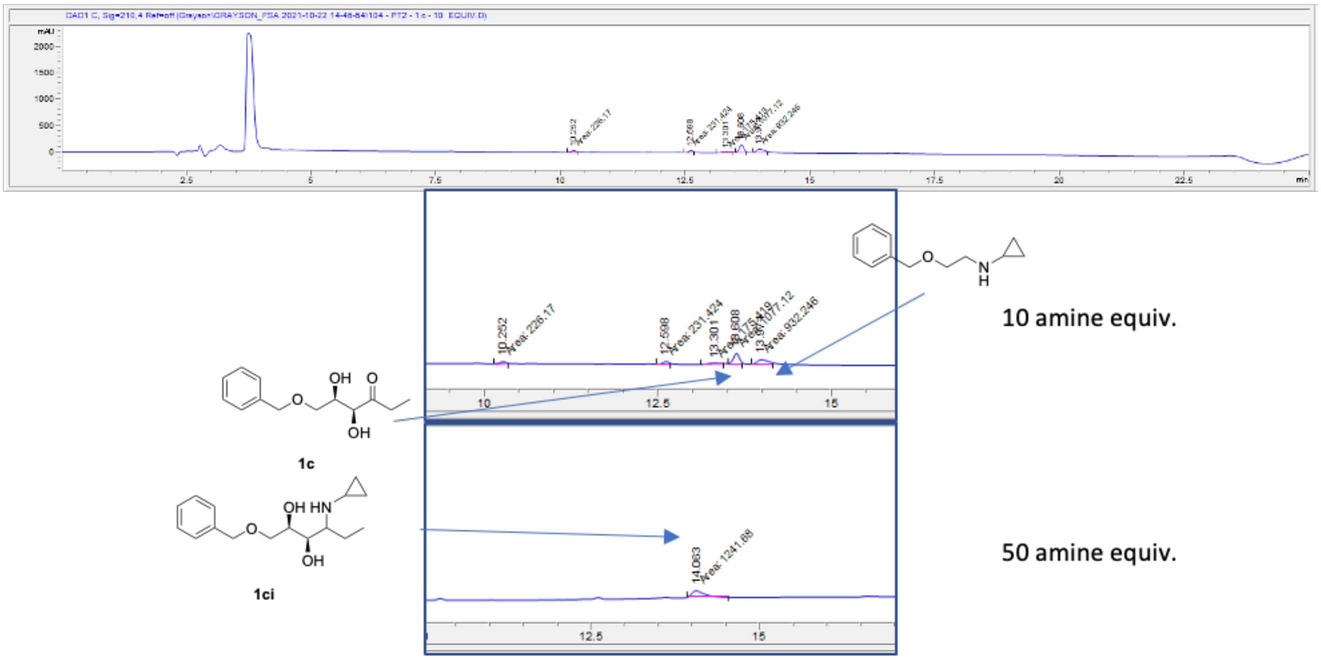
10 amine equiv.
50 amine equiv.
1c
1ci
10.252
Area: 226.17
12.598
Area: 231.424
13.301
13.608
Area: 932.246
14.063
Area: 1241.68

Example Chromatograms of one-pot two-step FSA-IR259 cascade. Second step (IR259 step only)

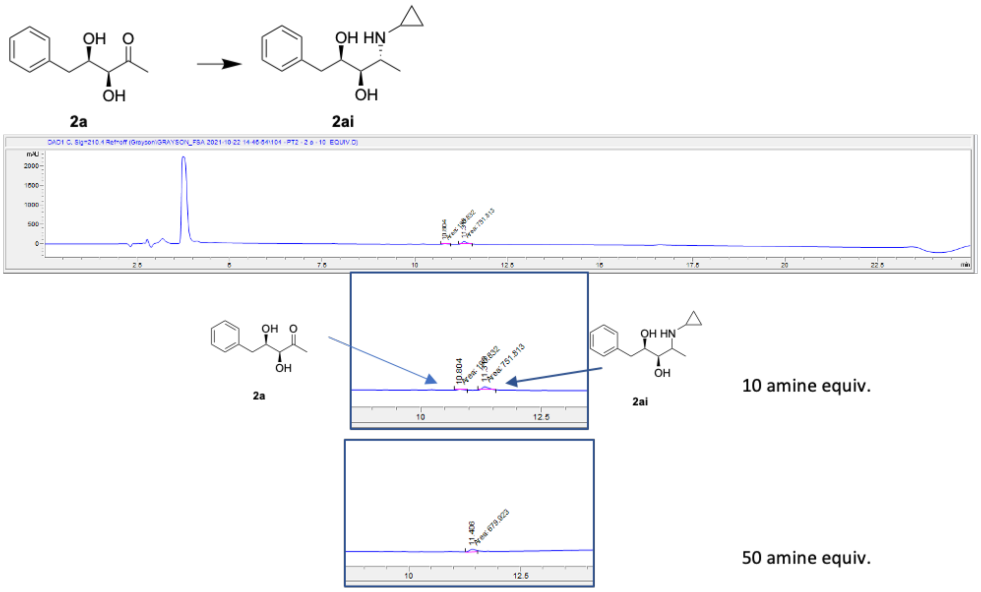

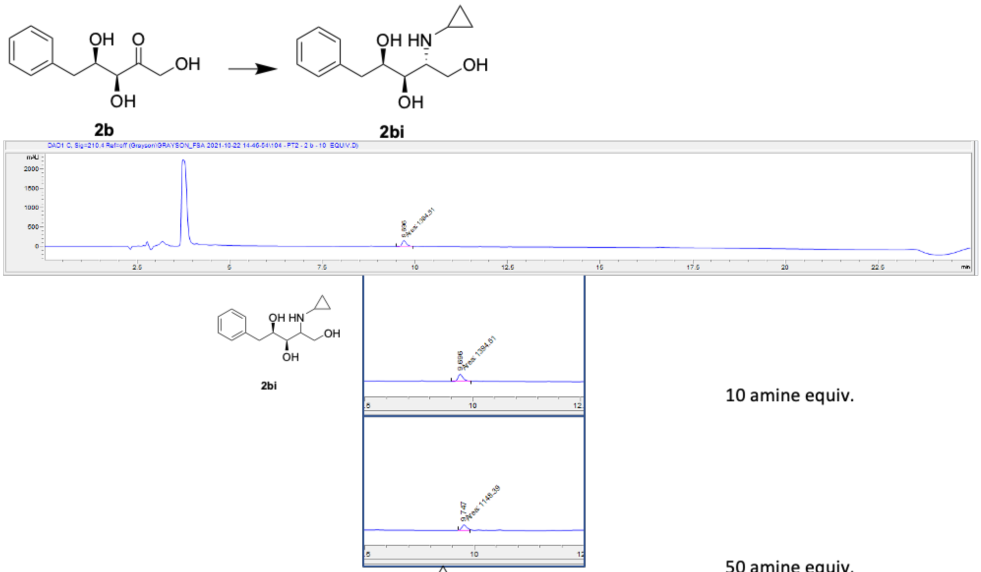

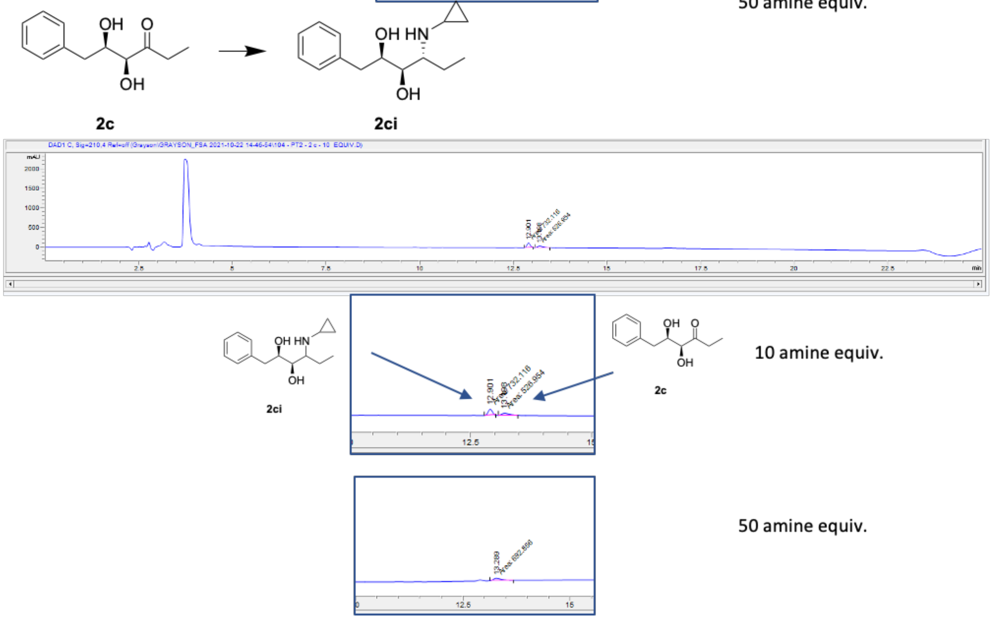

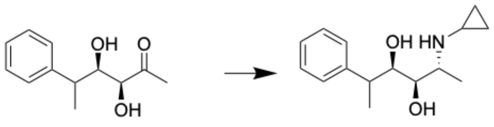

3a **3ai**

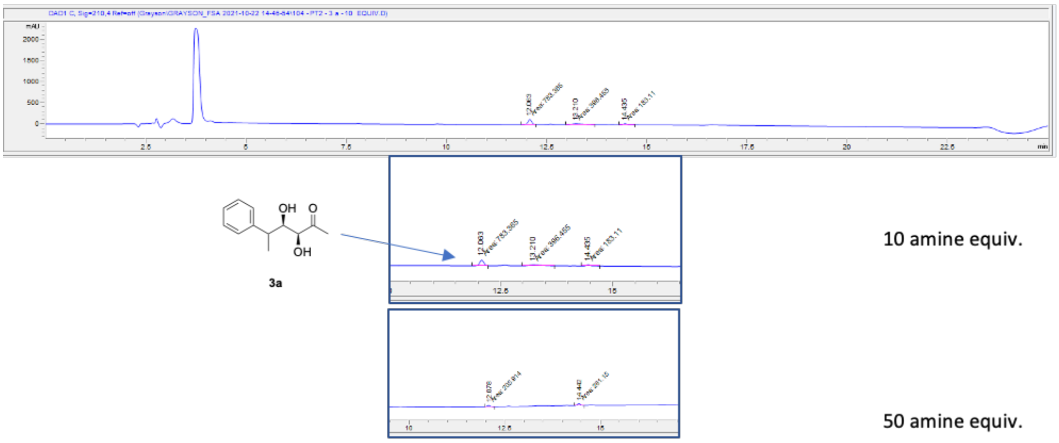

10 amine equiv.

50 amine equiv.

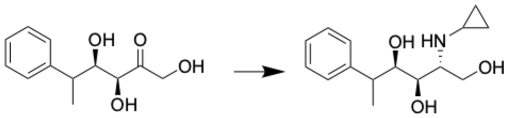

3b **3bi**

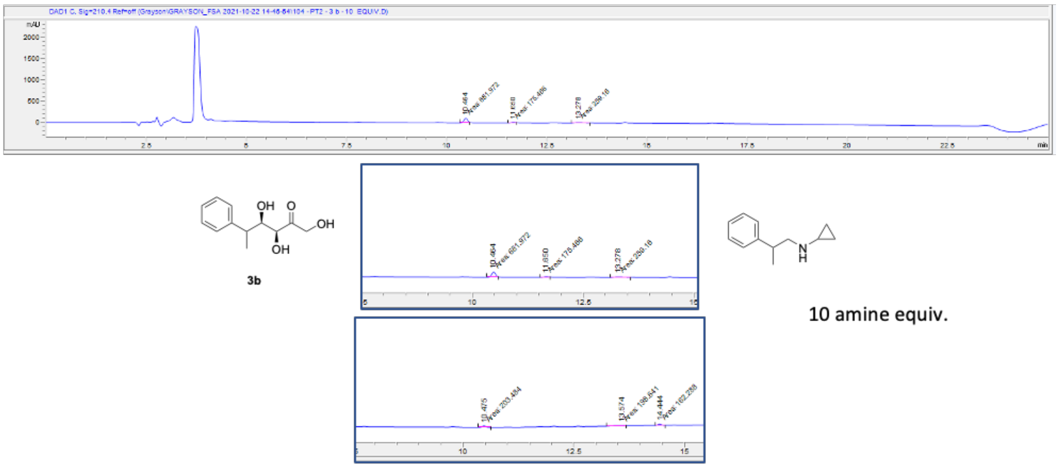

10 amine equiv.

50 amine equiv.

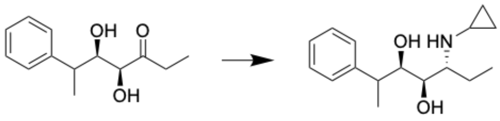

3c **3ci**

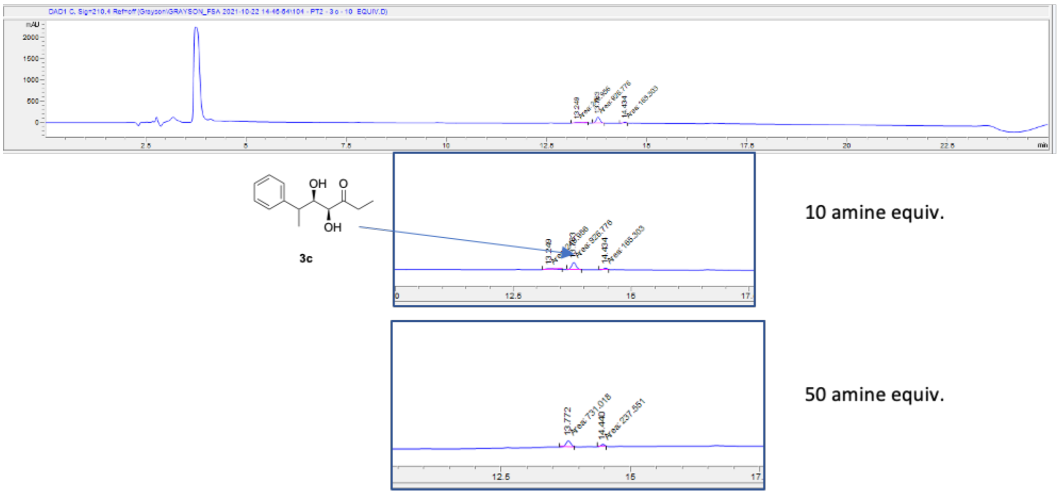

10 amine equiv.

50 amine equiv.

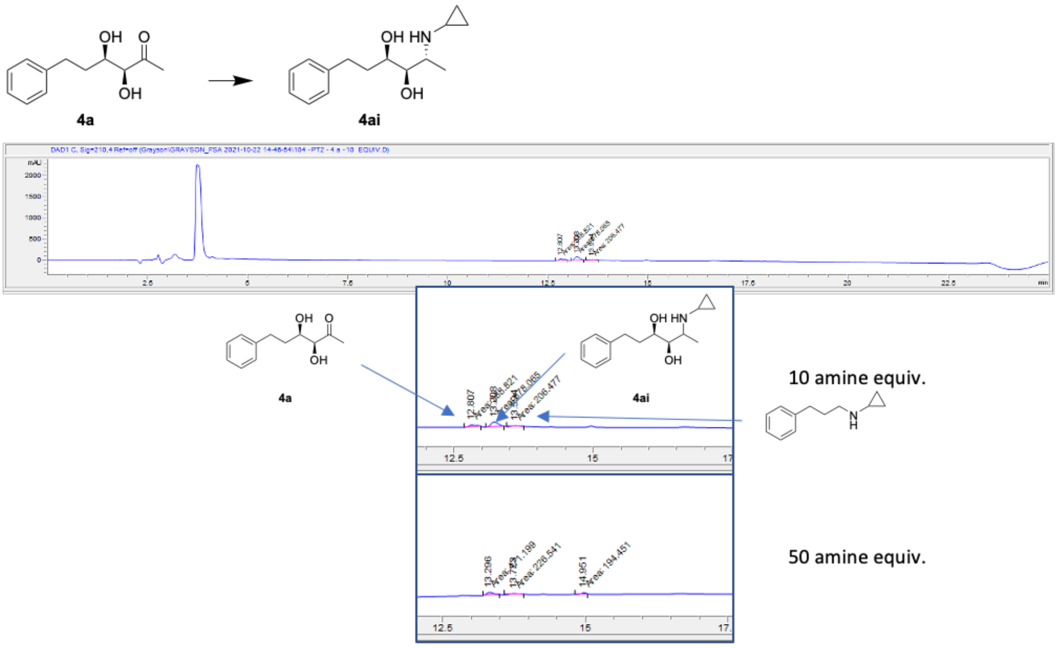
4a
4ai
10 amine equiv.
50 amine equiv.

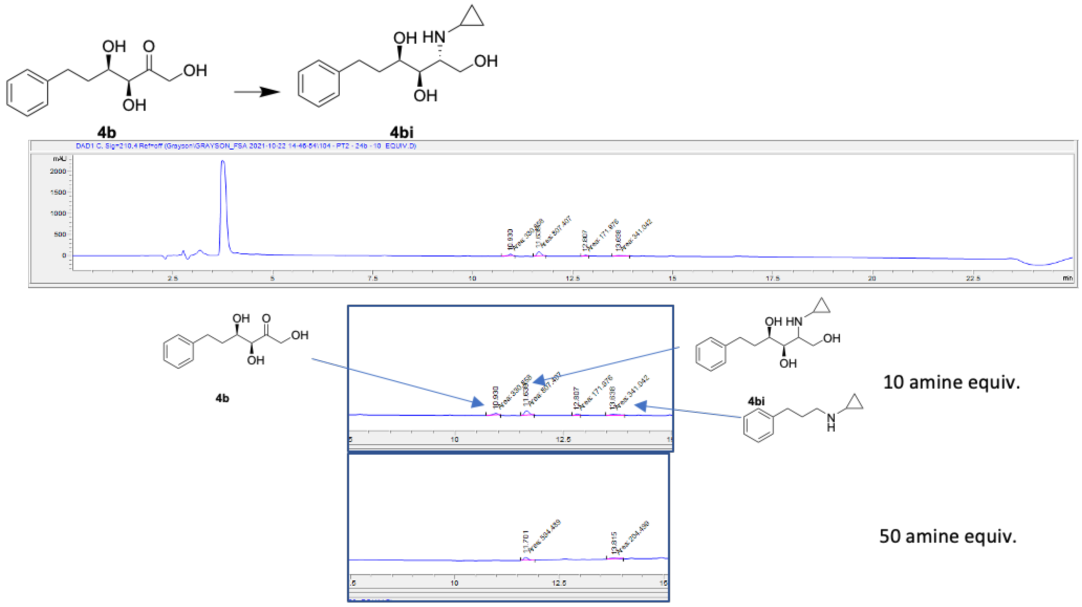
4b
4bi
10 amine equiv.
50 amine equiv.

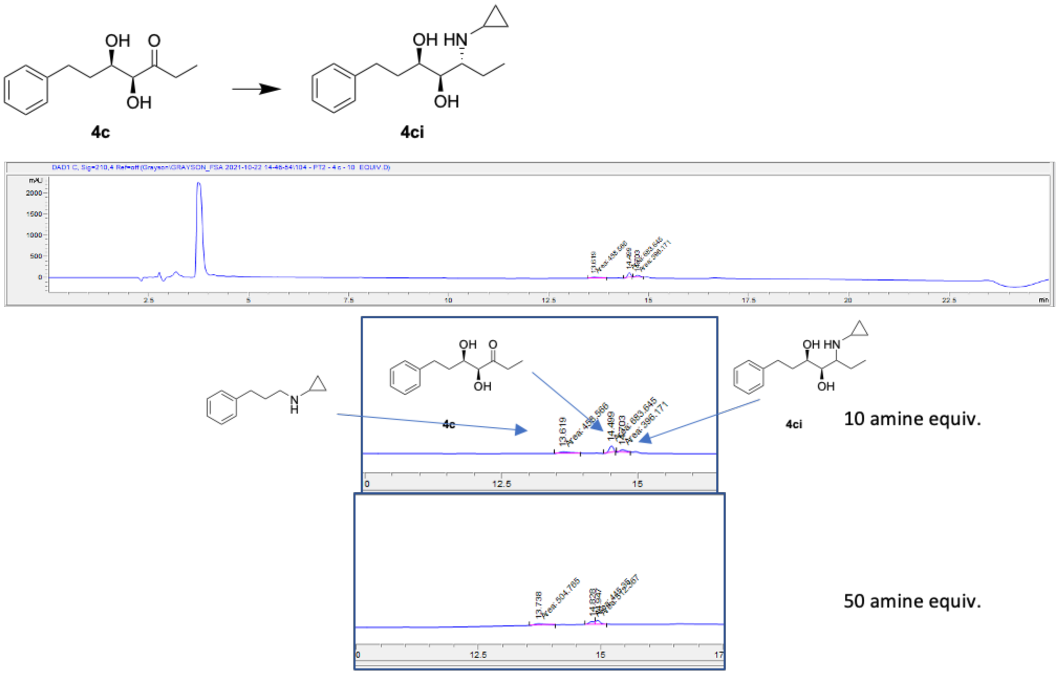
4c
4ci
10 amine equiv.
50 amine equiv.

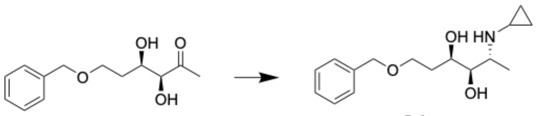

5a **5ai**

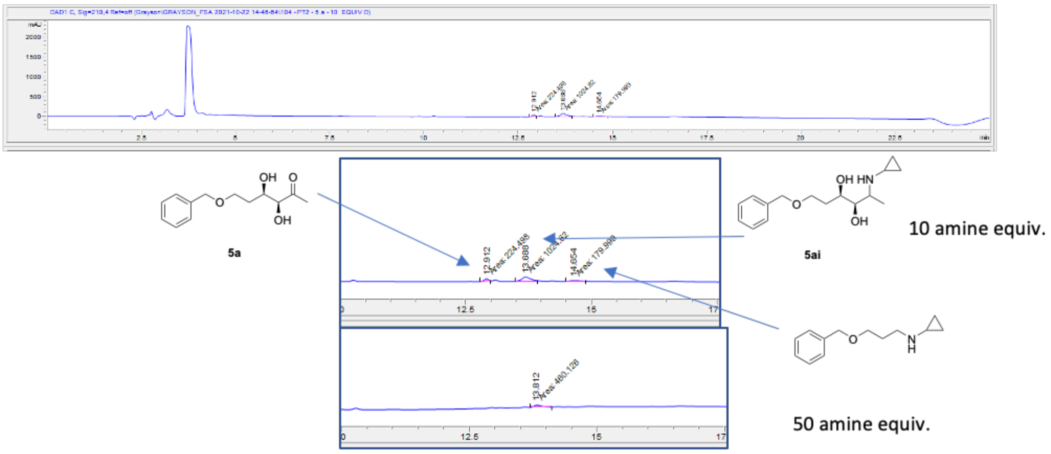

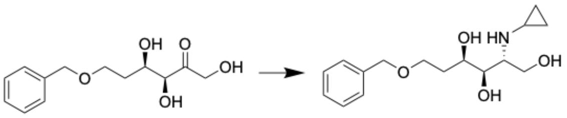

5b **5bi**

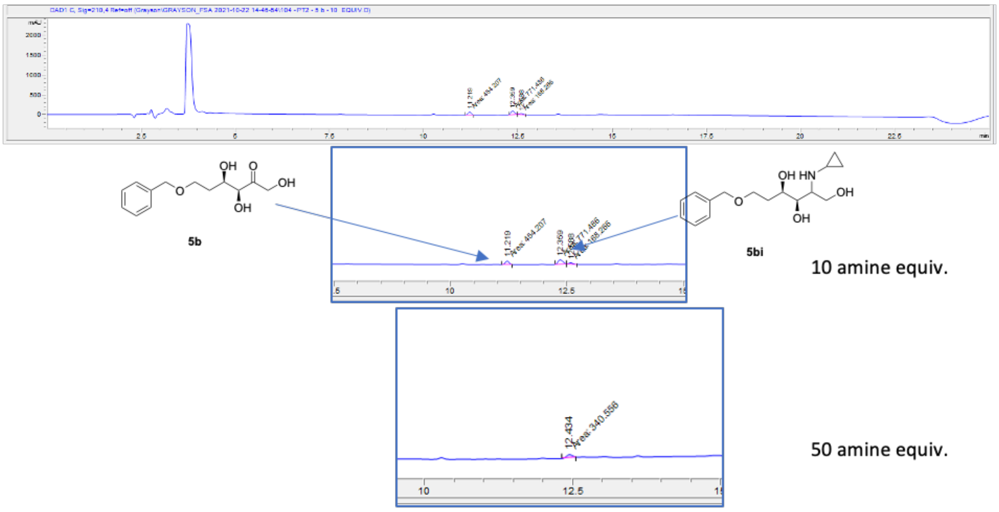

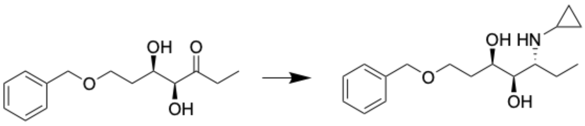

5c **5ci**

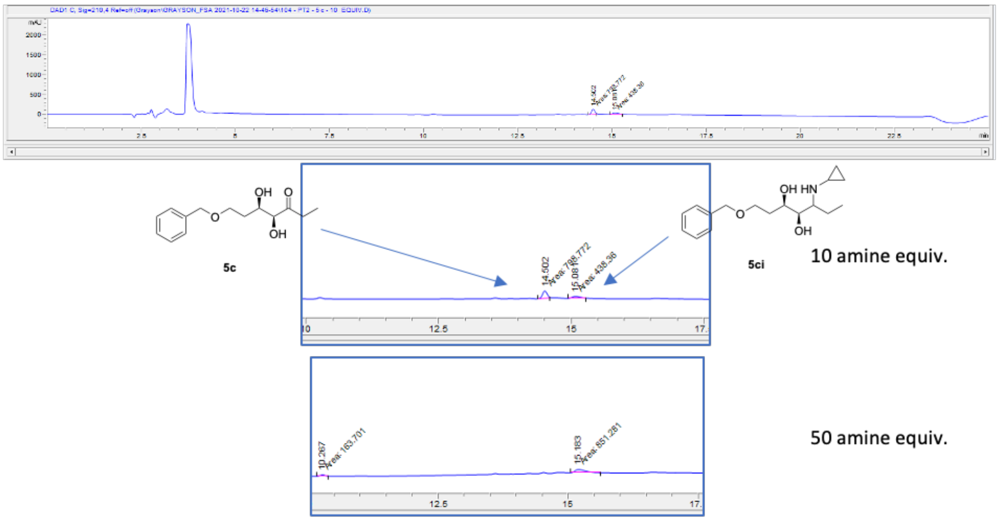

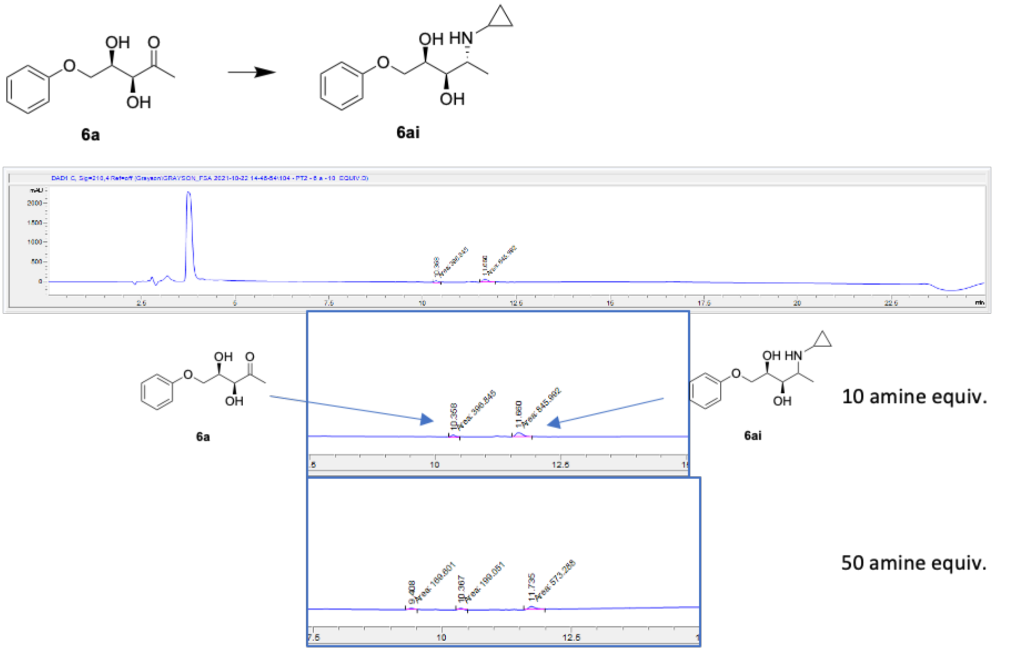
6a
6ai
10 amine equiv.
50 amine equiv.

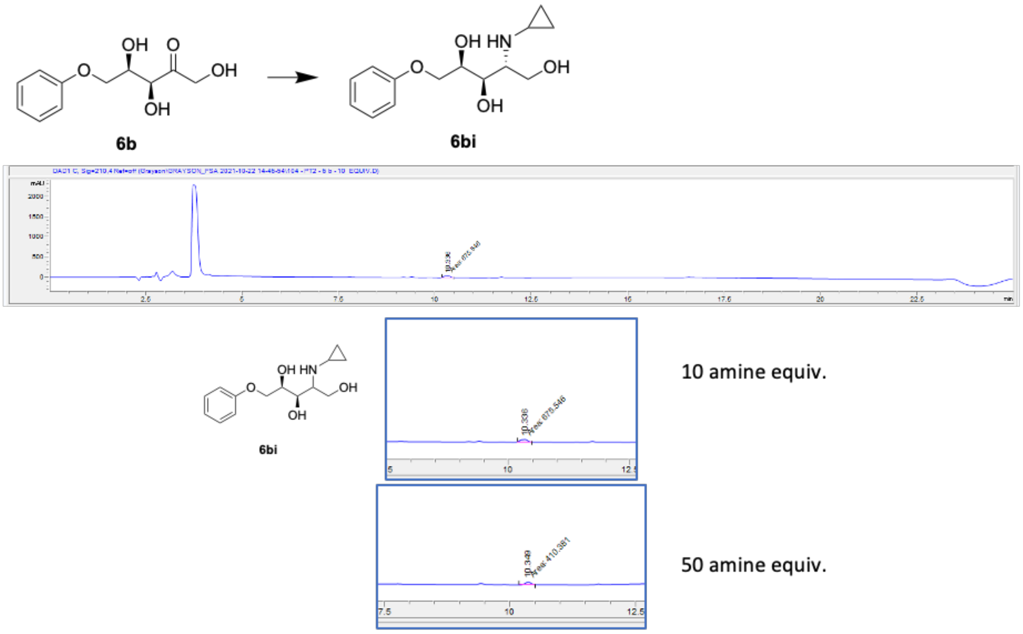
6b
6bi
10 amine equiv.
50 amine equiv.

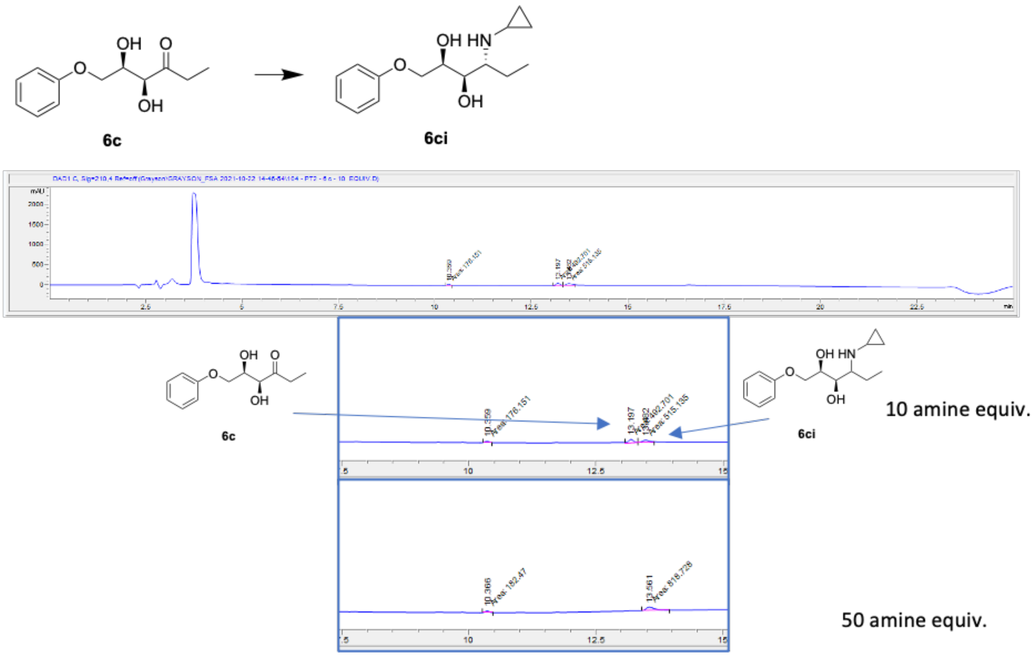
6c
6ci
10 amine equiv.
50 amine equiv.

Example Chromatograms of preparative scale FSA-IR259 cascade

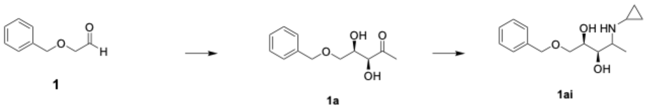

Step one – after FSA reaction

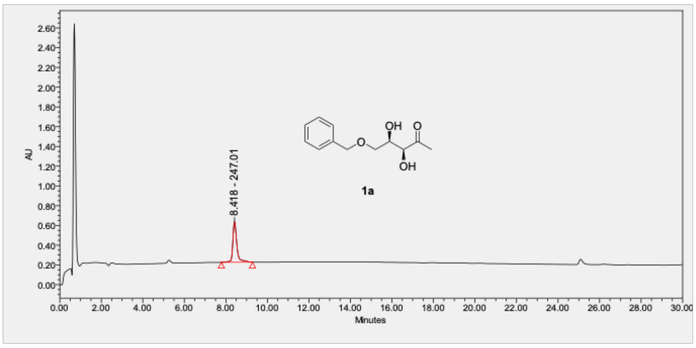

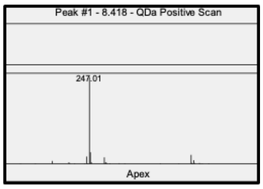

Step two – after IR-259 reaction

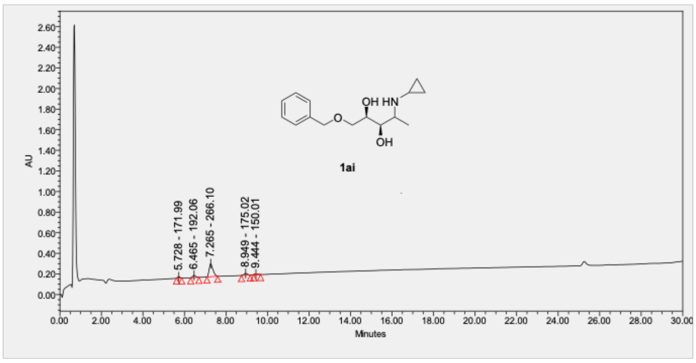

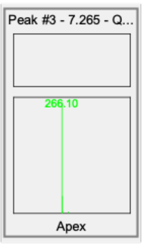

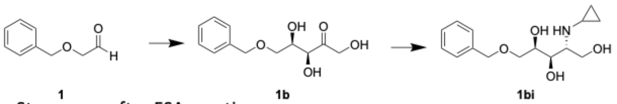

Step one – after FSA reaction

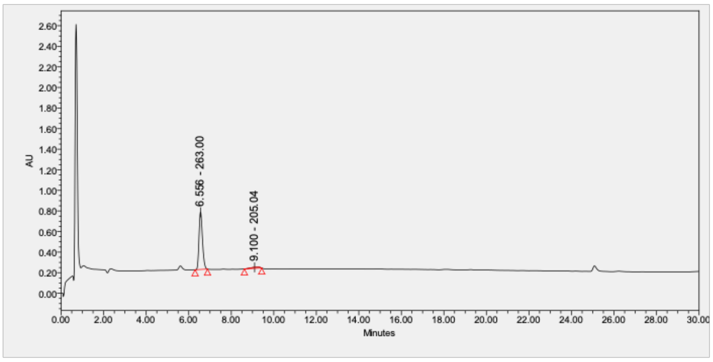

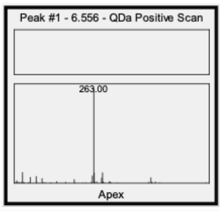

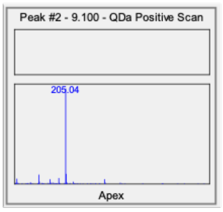

Step two – after IR-259 reaction

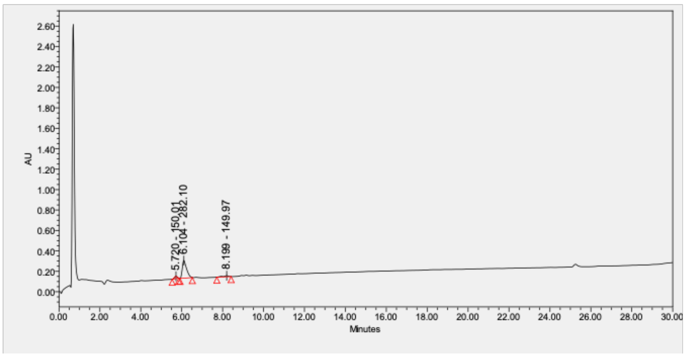

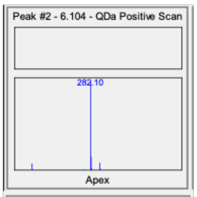

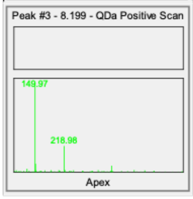

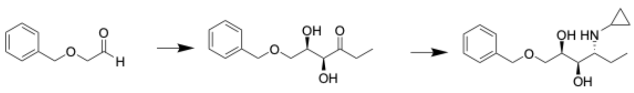

1 1c 1ci

Step one – after FSA reaction

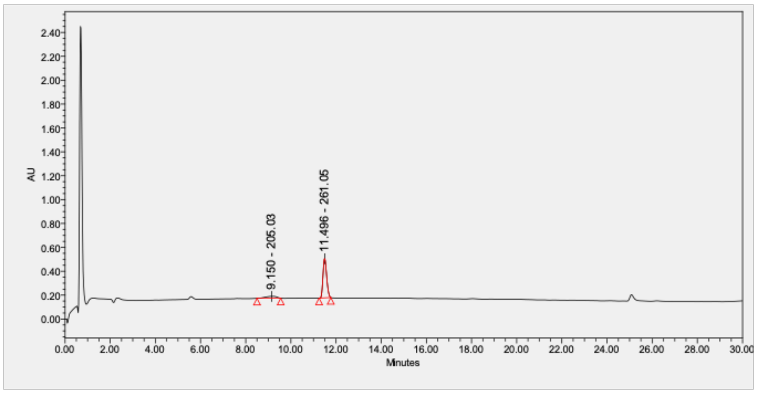

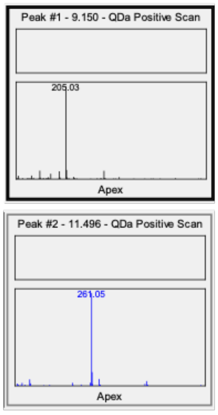

Step two – after IR-259 reaction

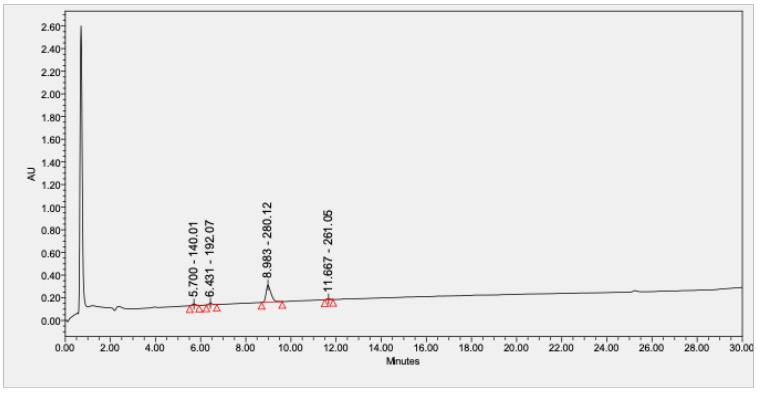

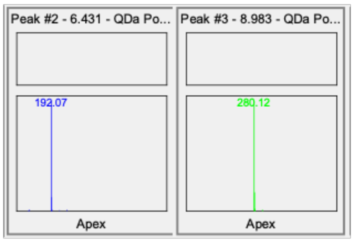

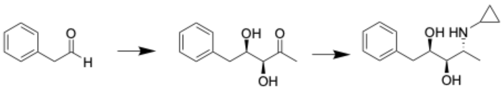

2 2a 2ai

Step one – after FSA reaction

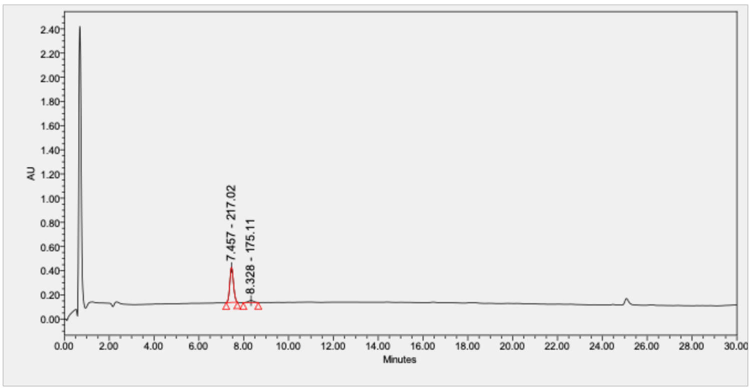

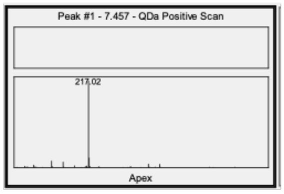

Step two – after IR-259 reaction

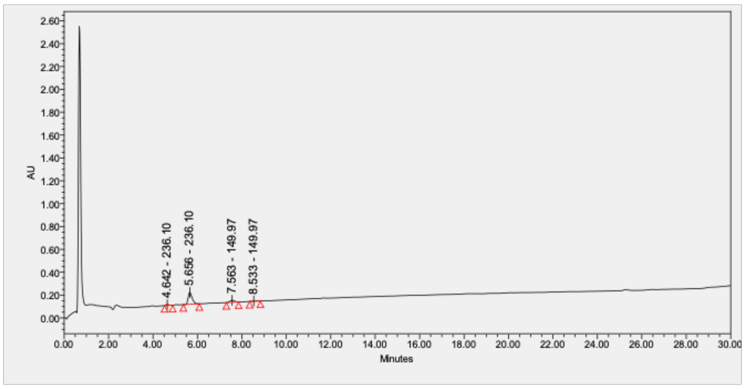

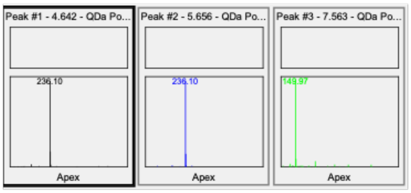

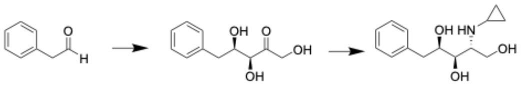

2 **2b** **2bi**

Step one – after FSA reaction

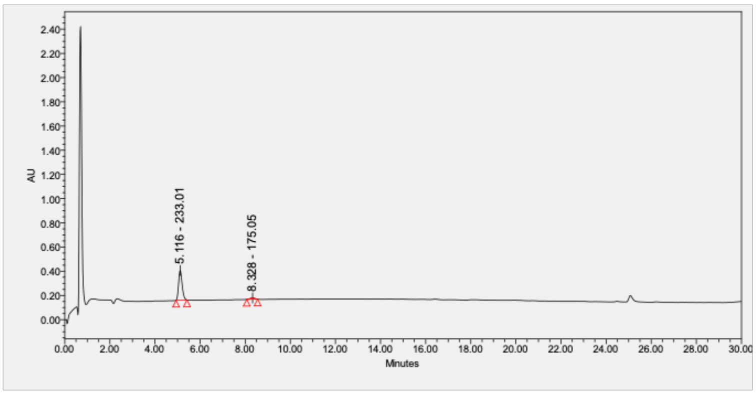

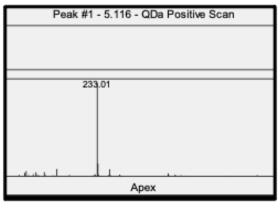

Step two – after IR-259 reaction

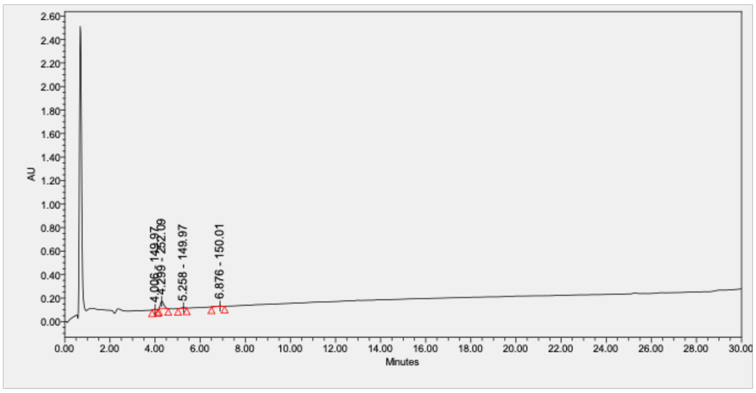

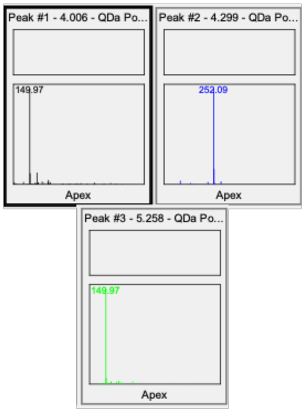

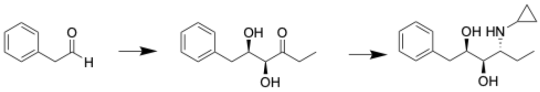

2 **2c** **2ci**

Step one

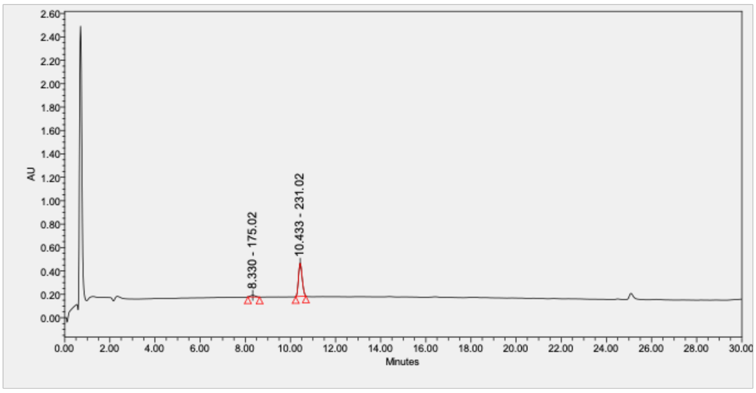

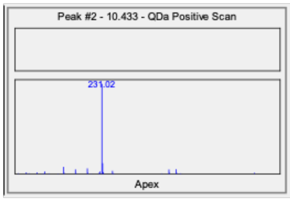

Step two

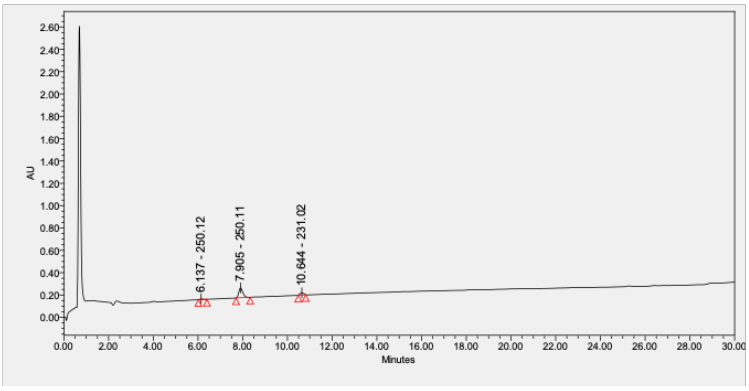

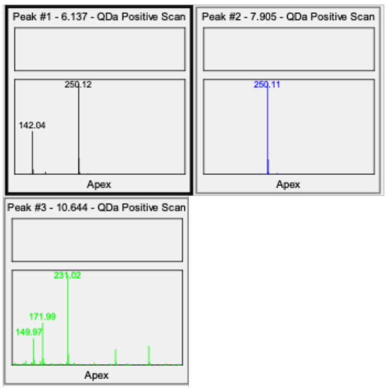

NMR Spectra of Chemically Synthesized Standards and substrates

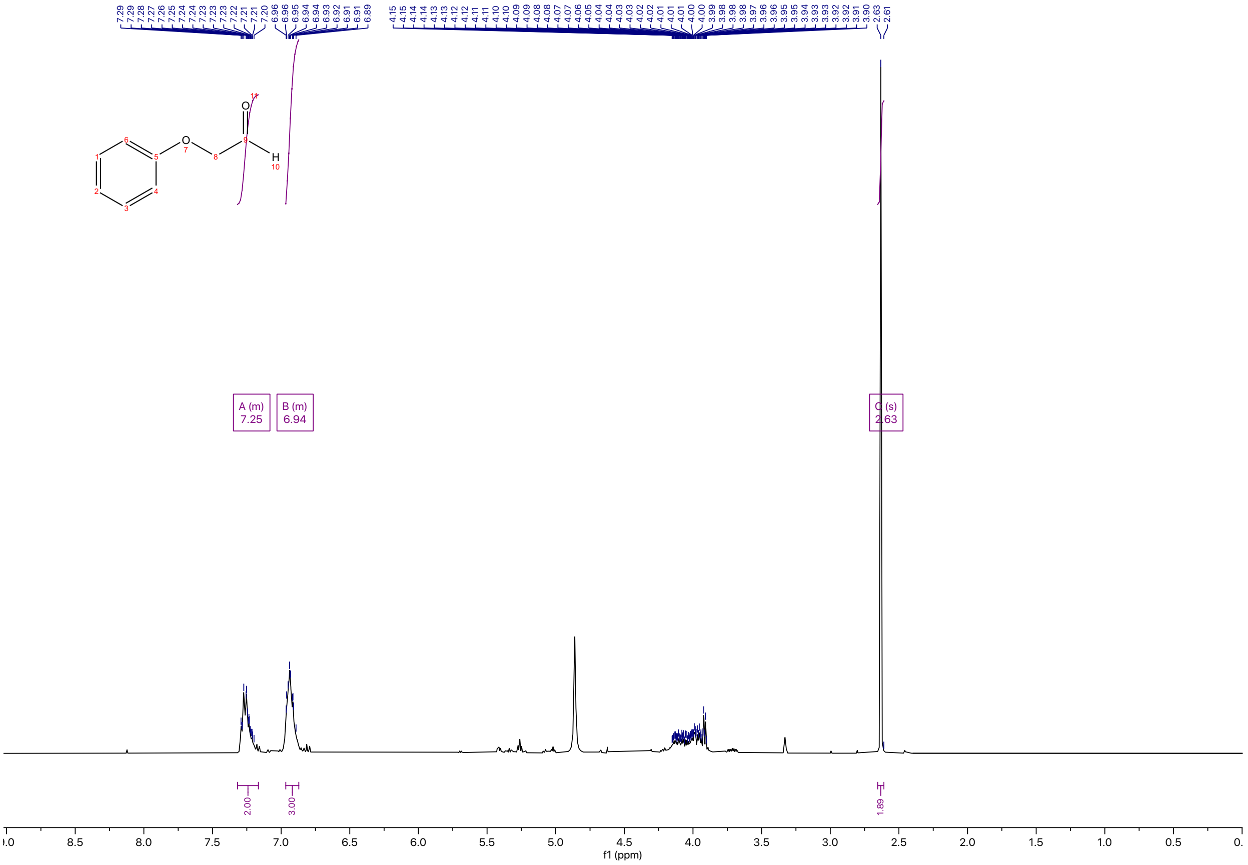

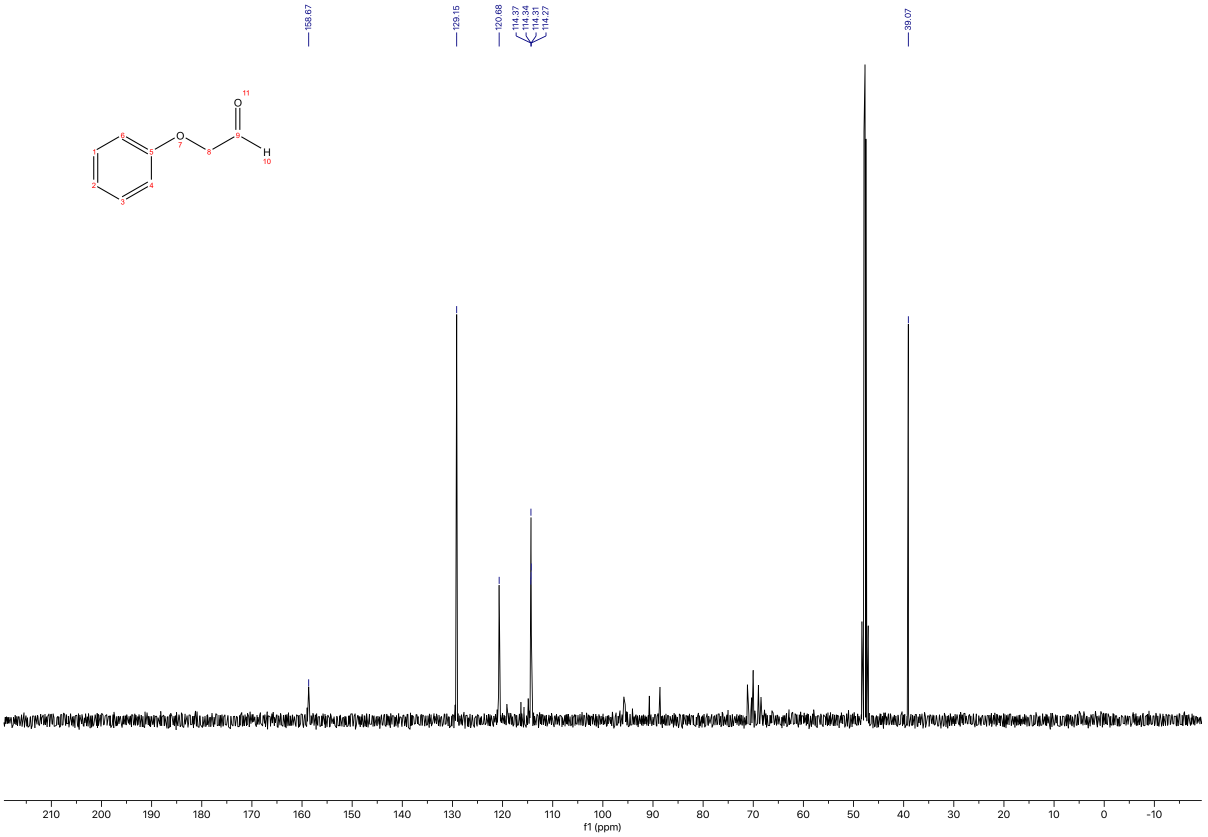

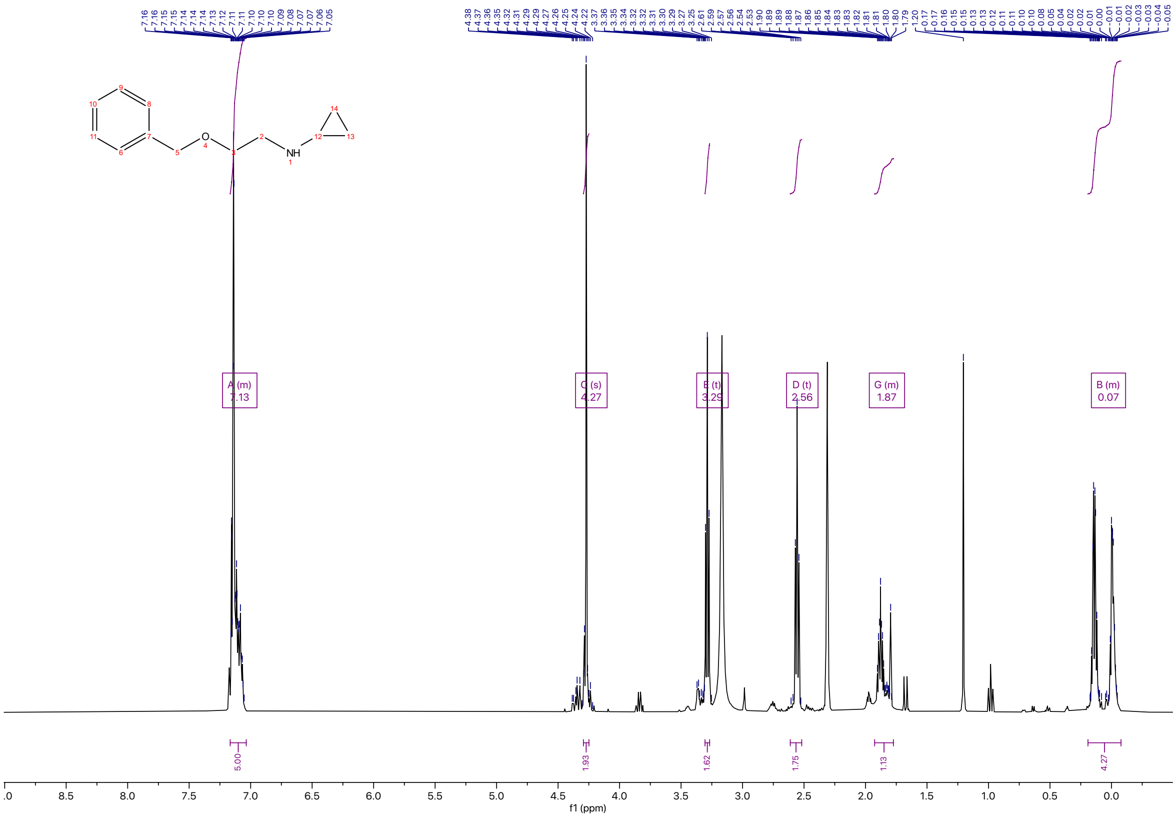

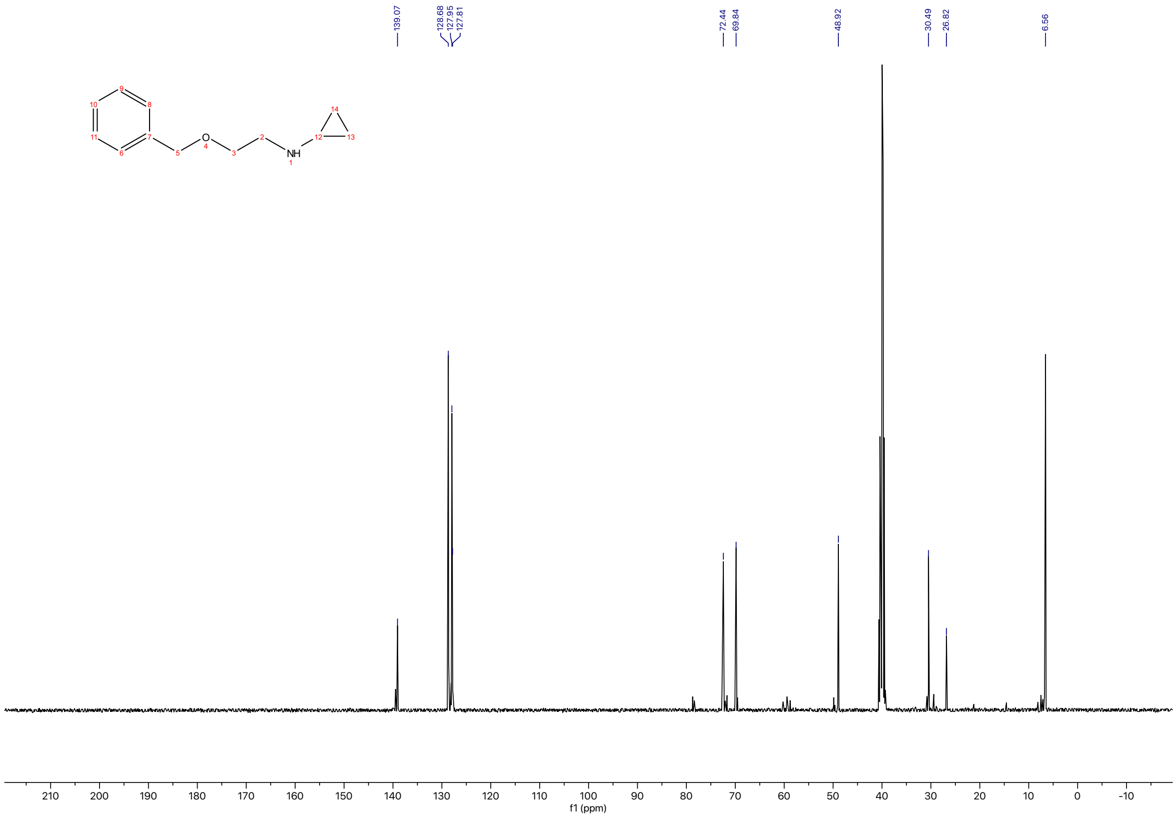

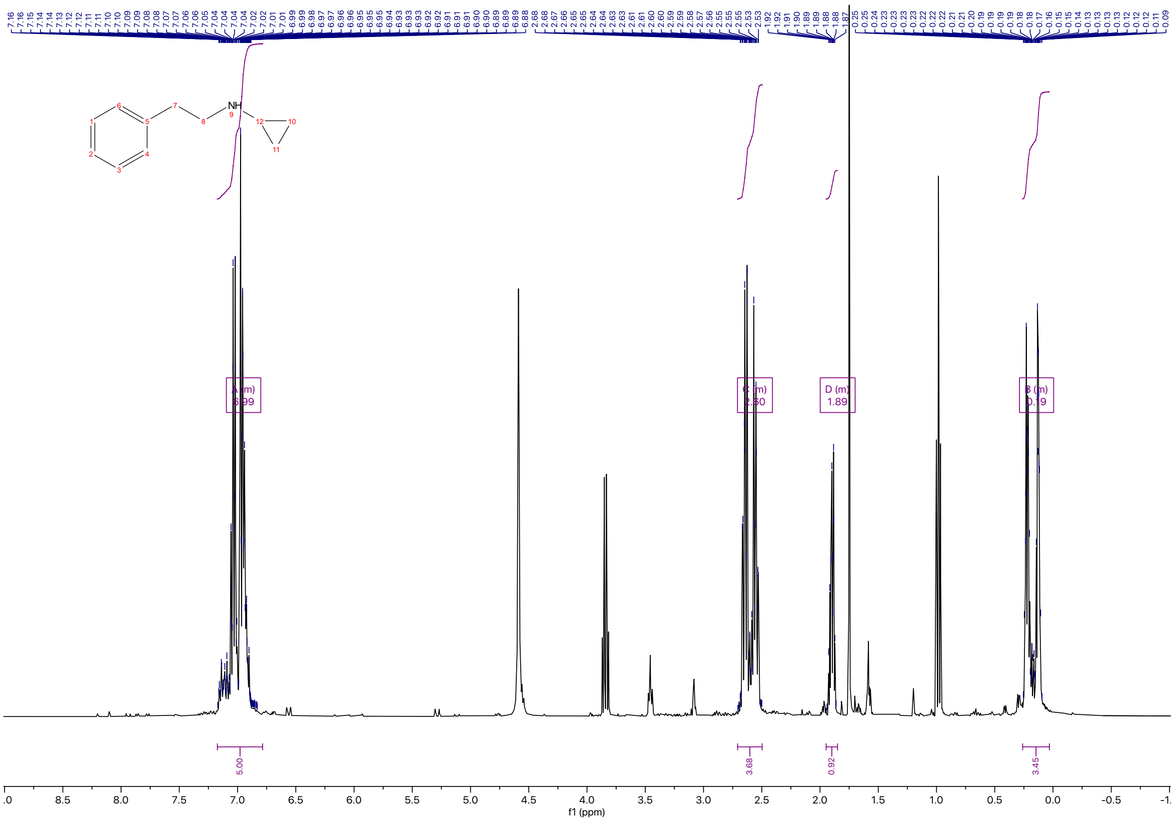

NH
A (m)
6.99
C (m)
2.60
D (m)
1.89
B (m)
0.19
5.00
3.68
0.92
3.45
f1 (ppm)

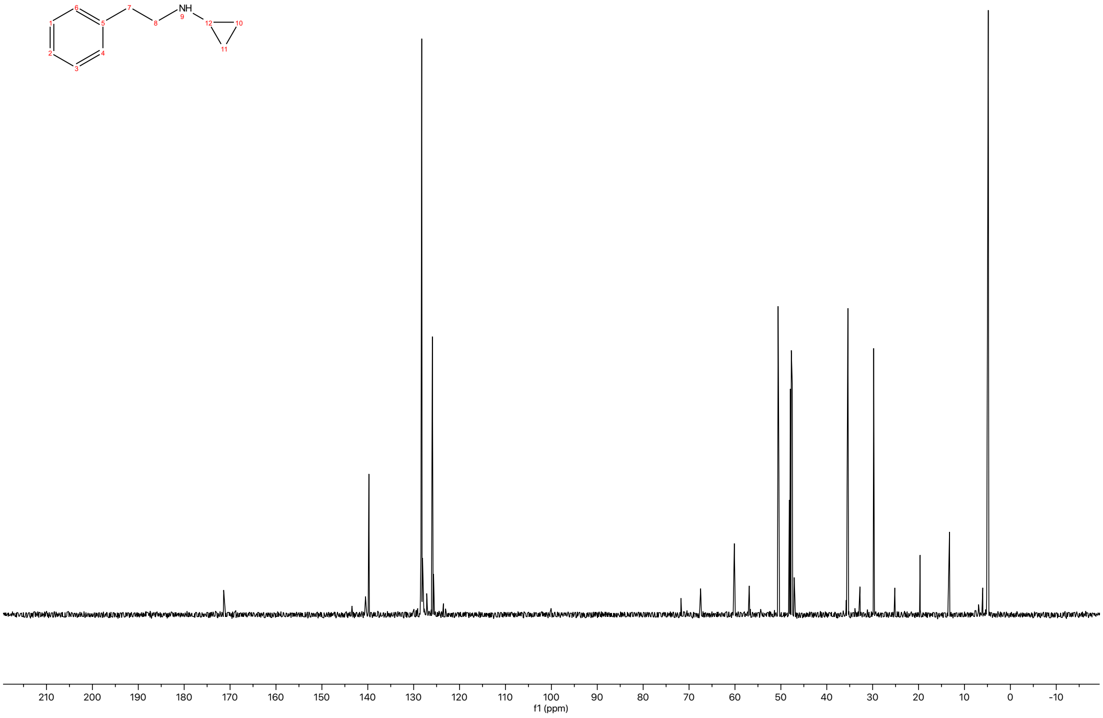

NH
f1 (ppm)

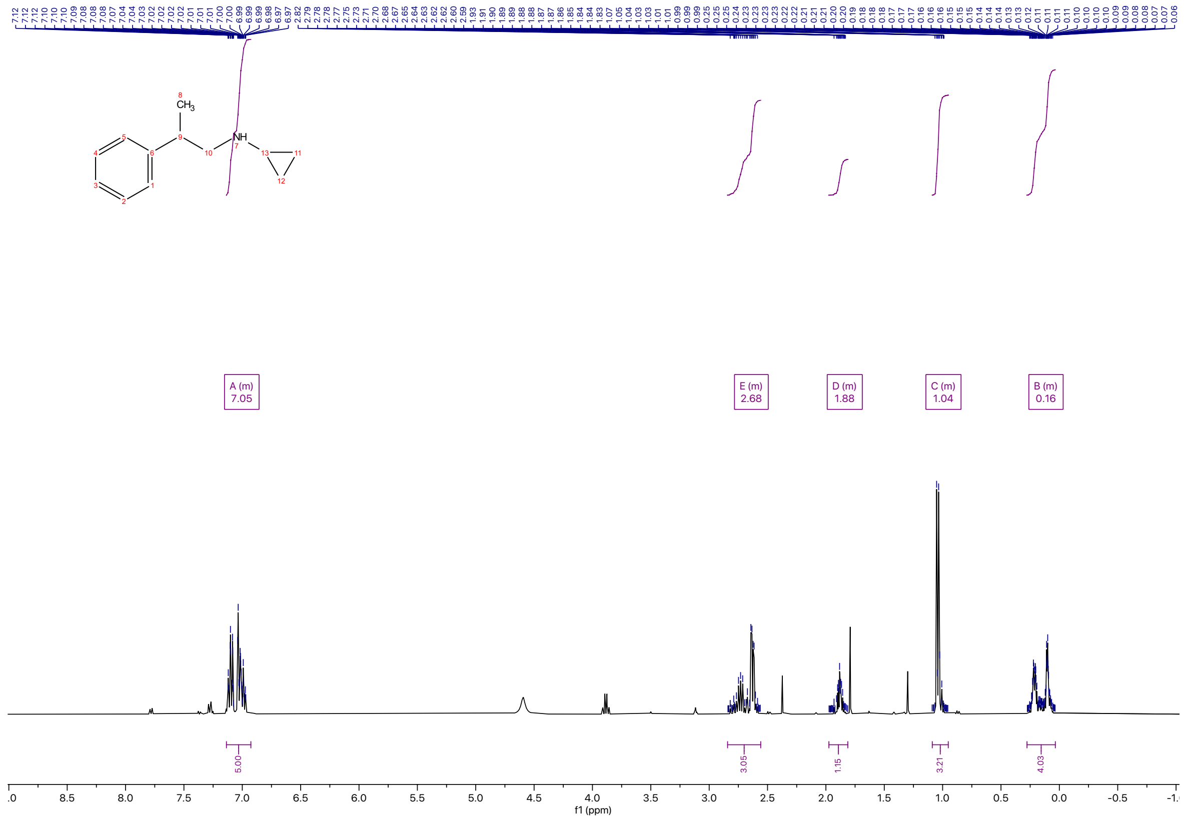

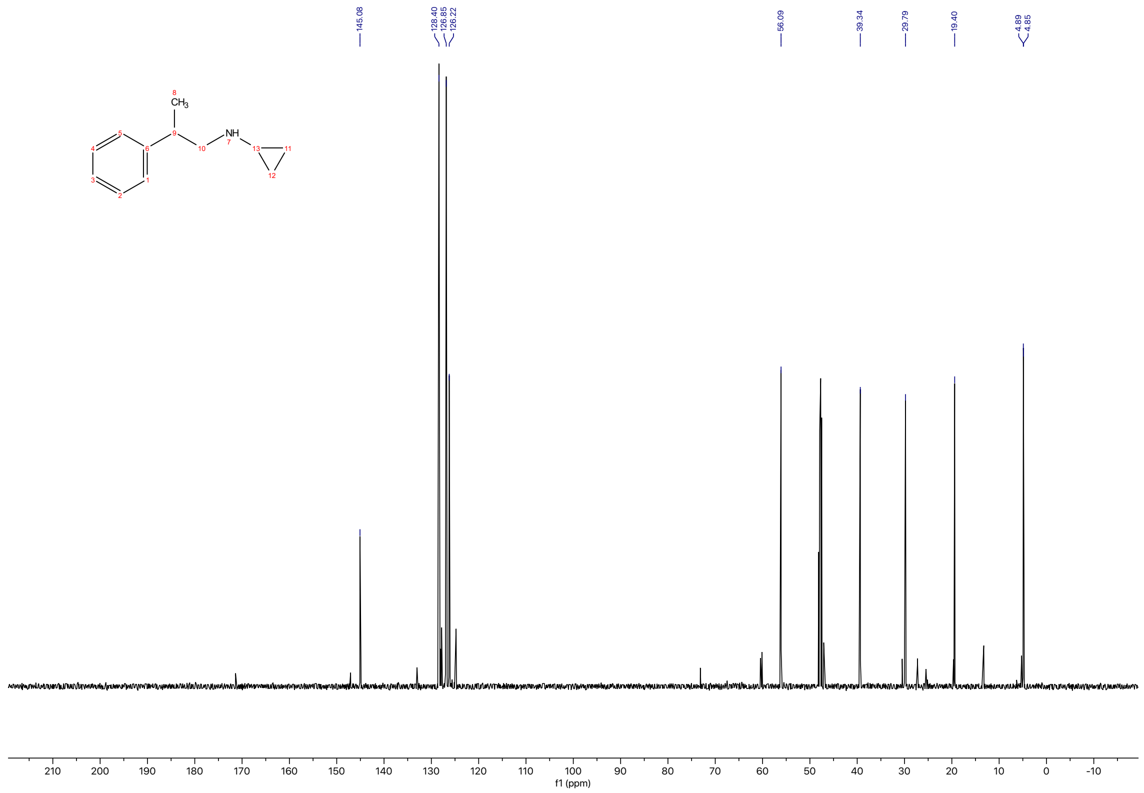

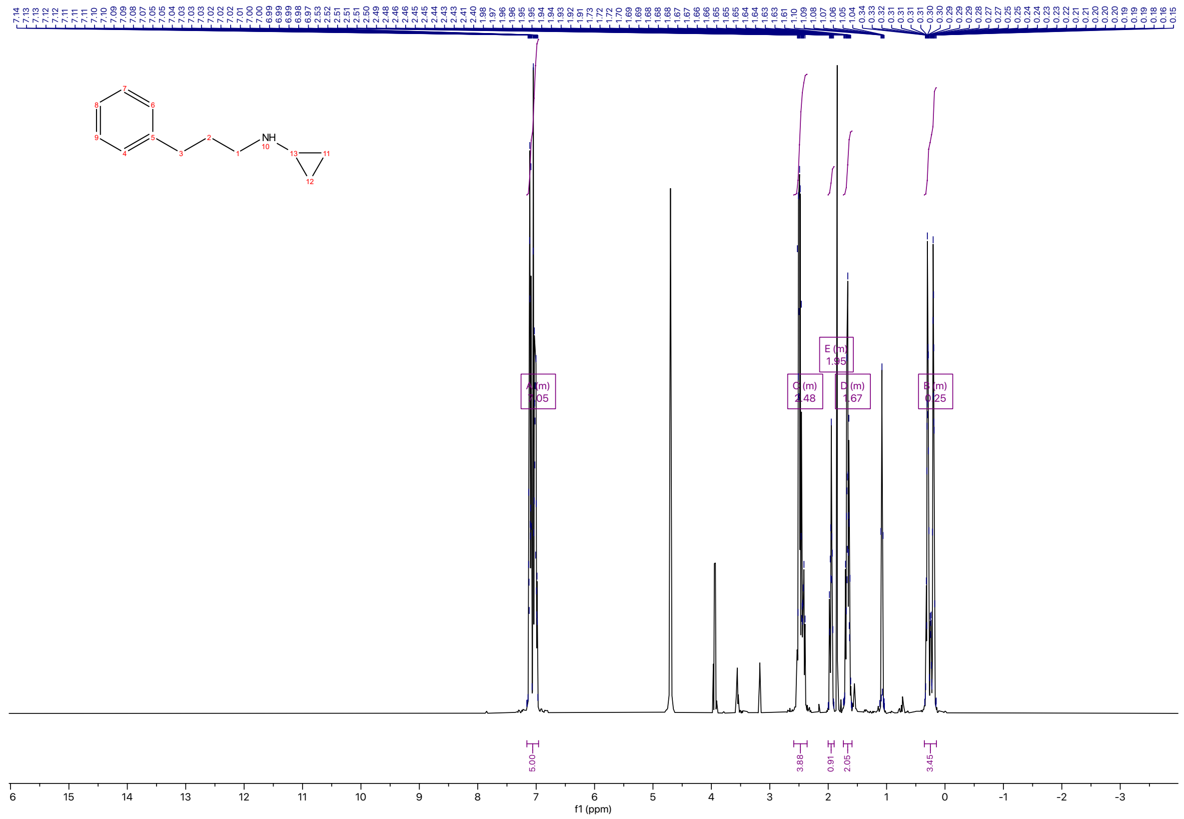

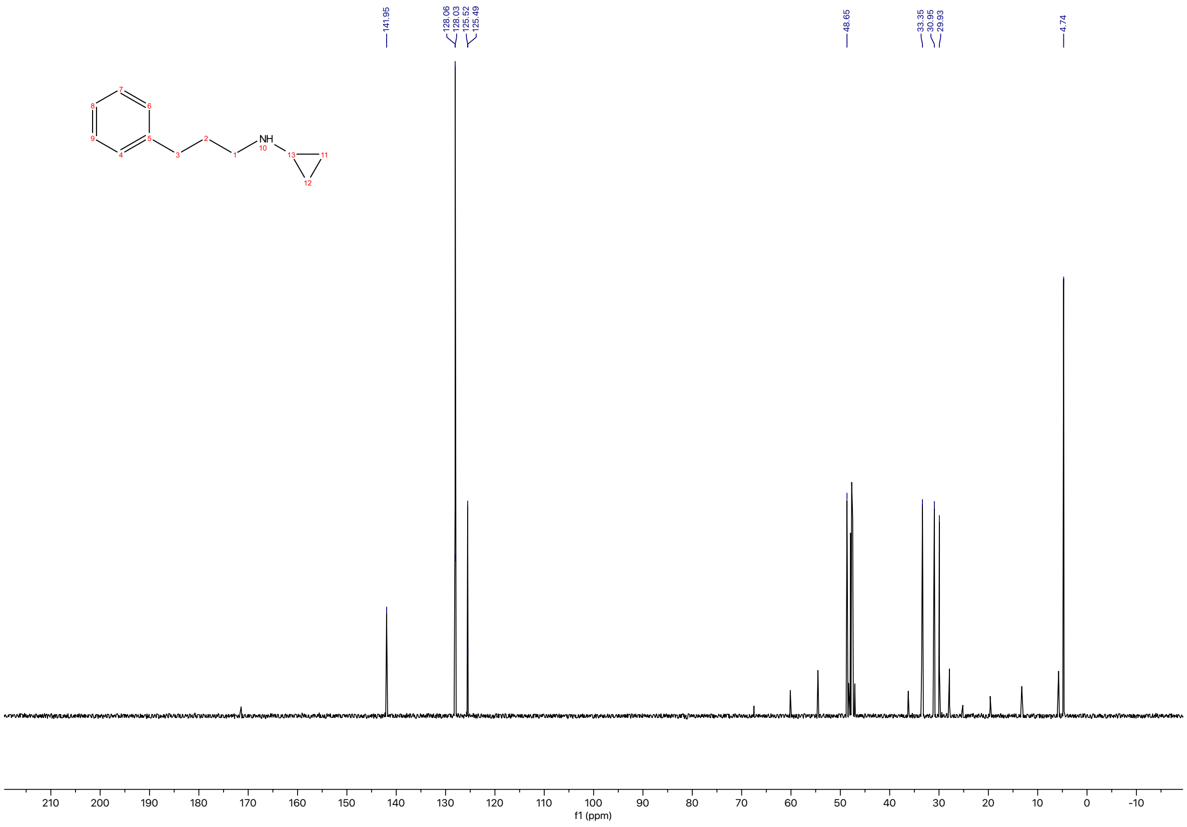
141.95
128.06
128.03
125.52
125.49
48.65
33.35
30.95
29.93
4.74
f1 (ppm)

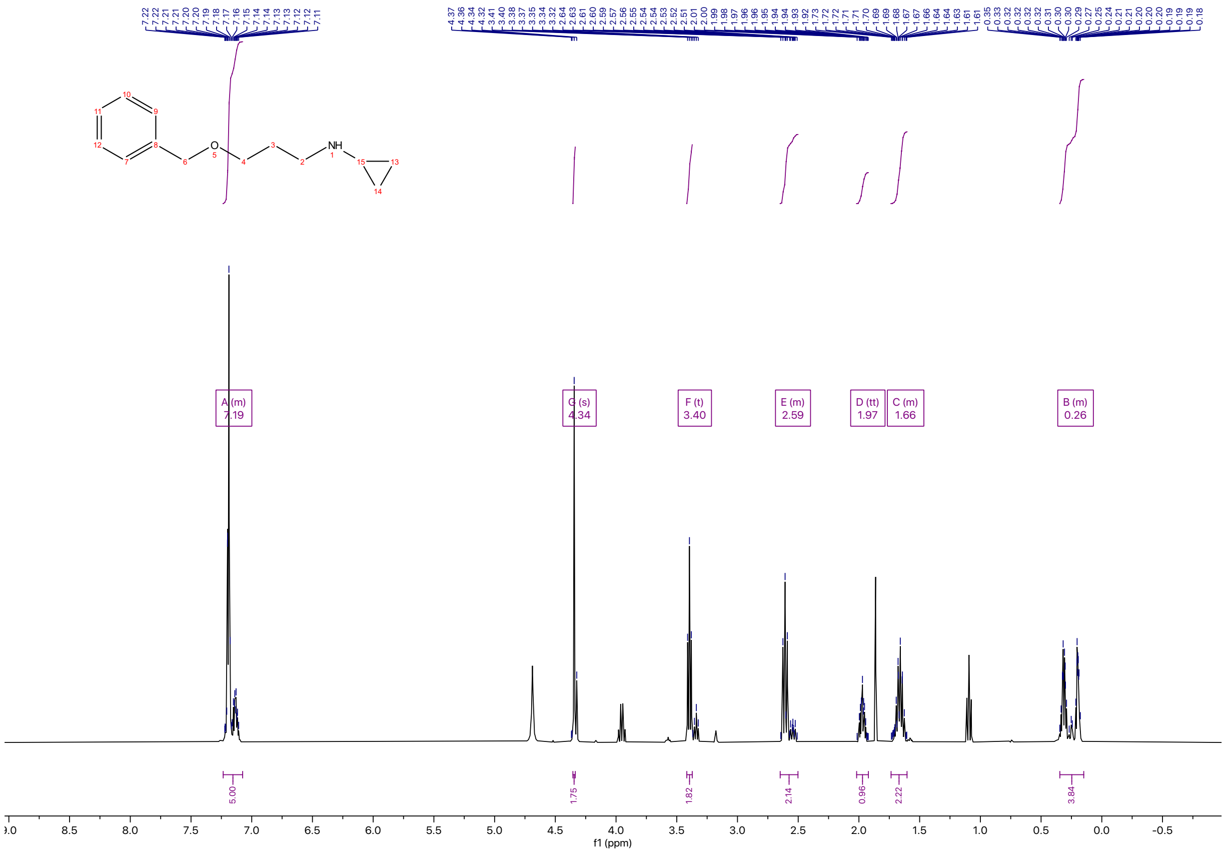

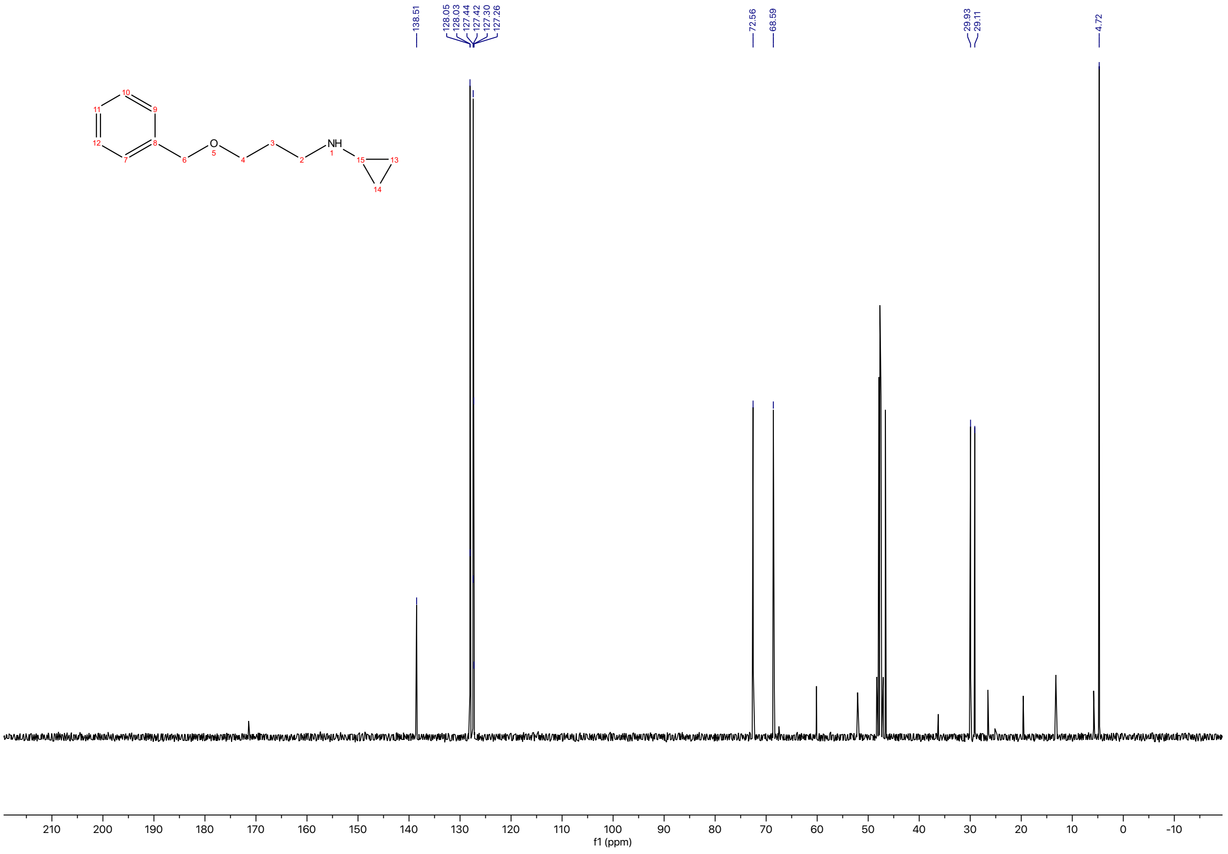

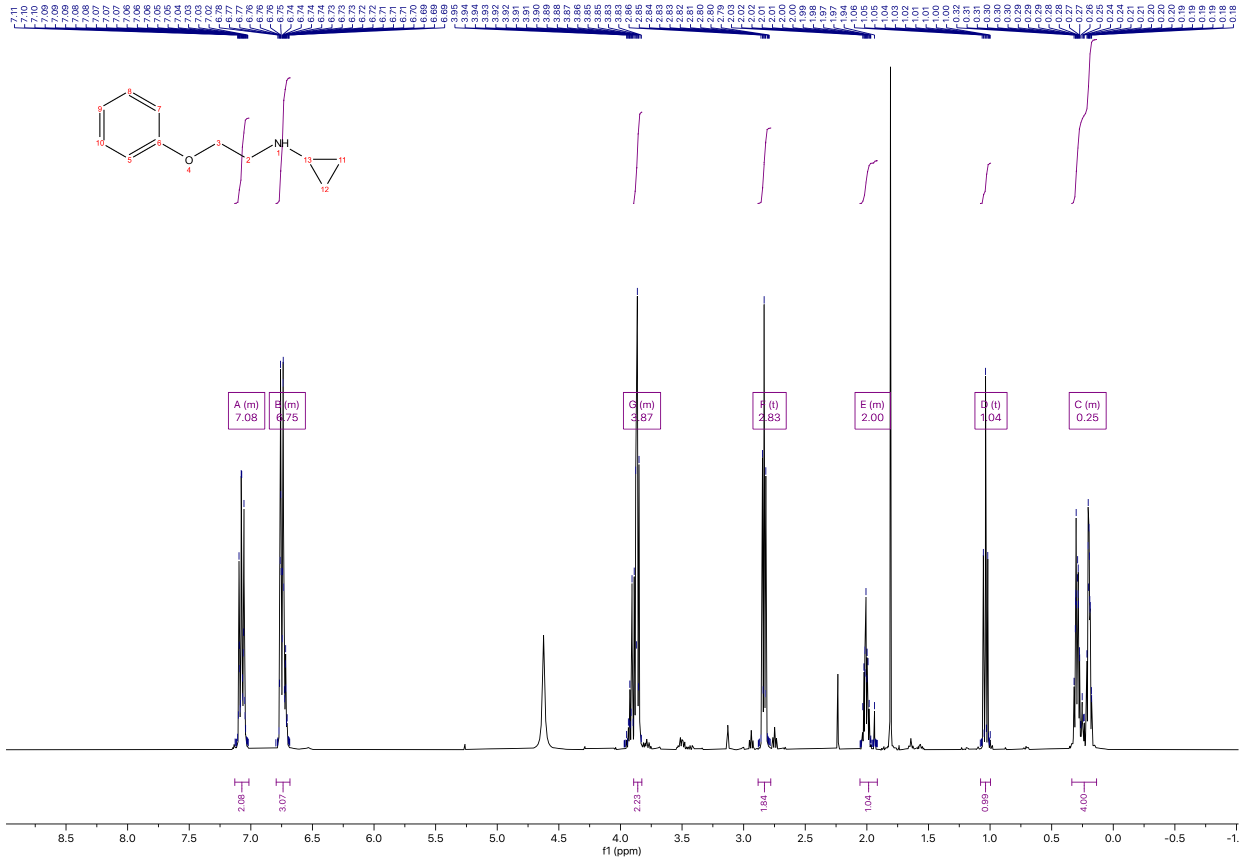

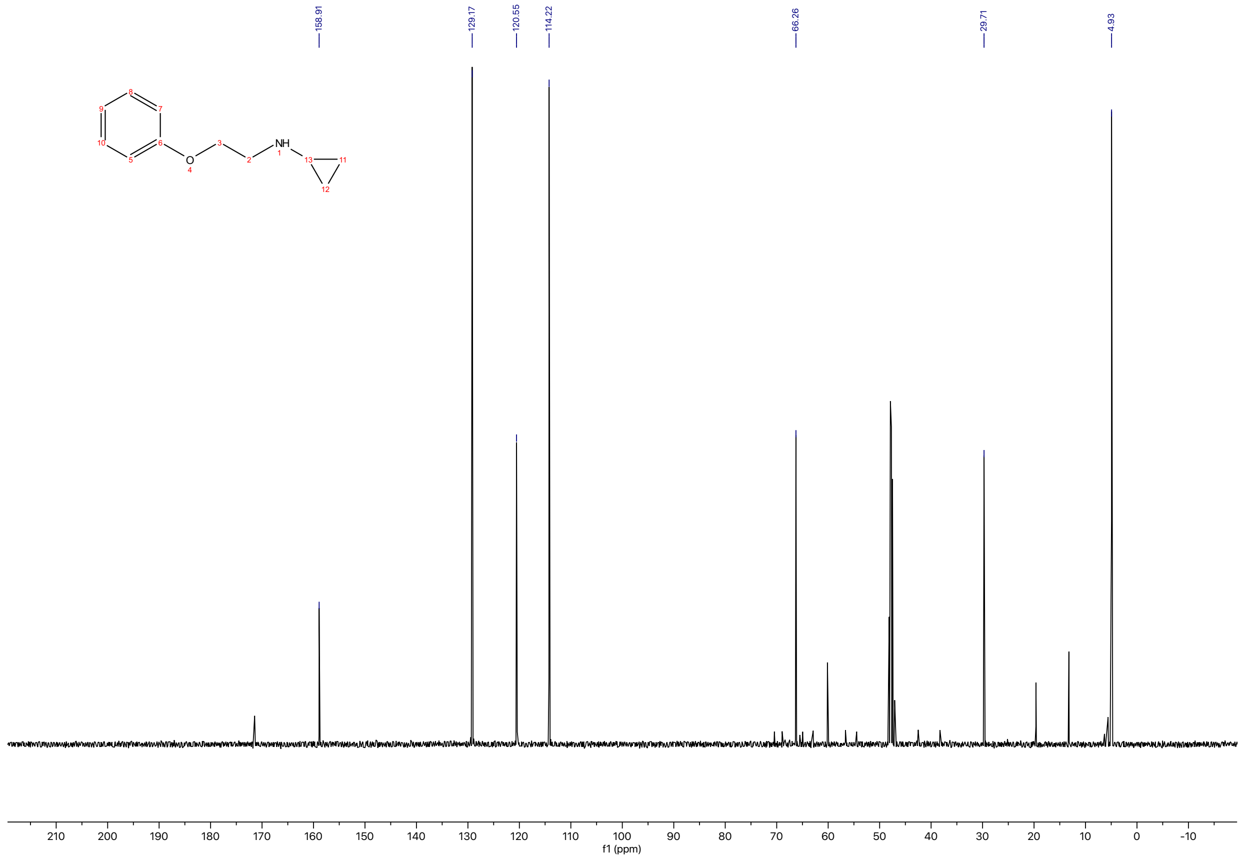

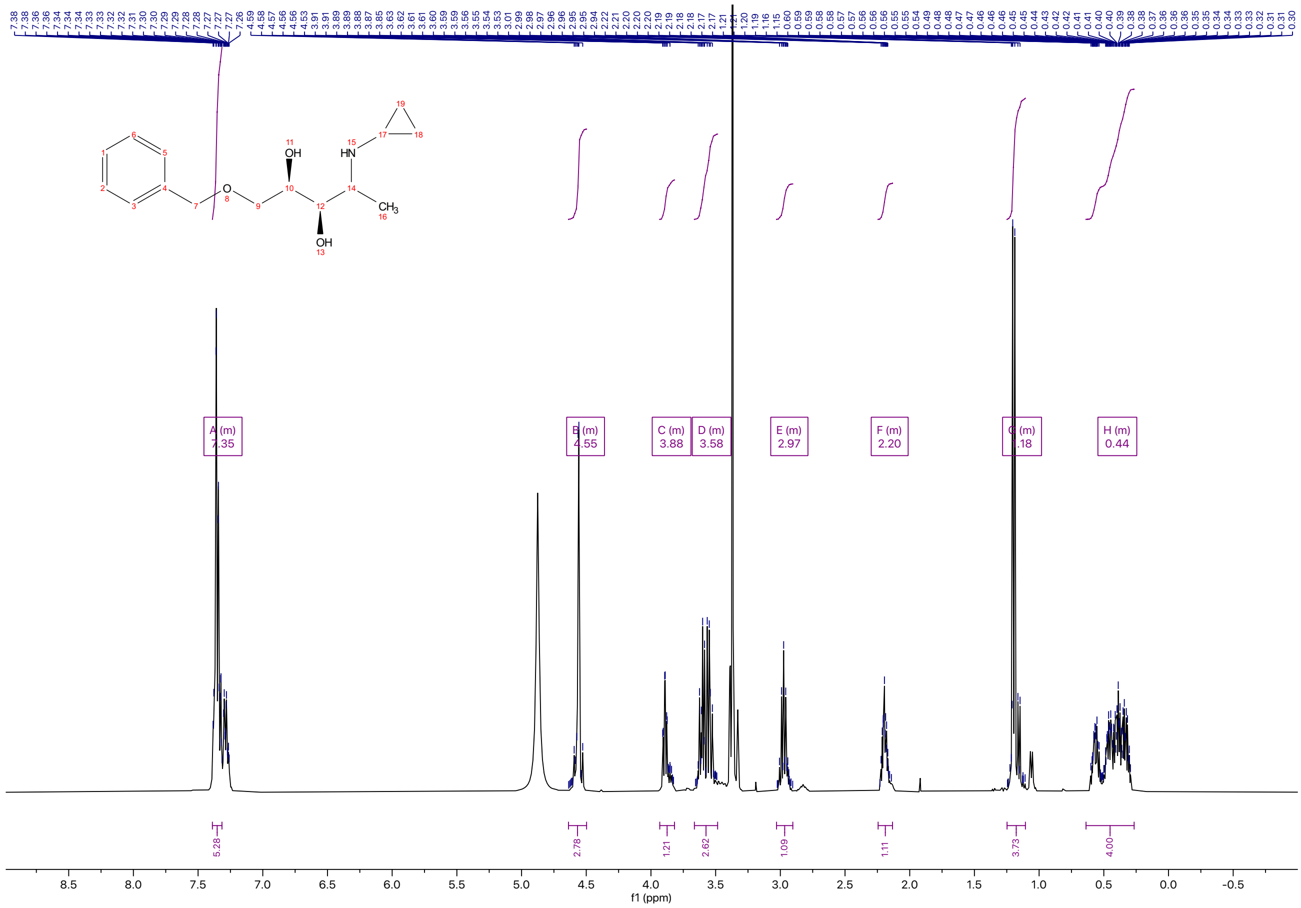

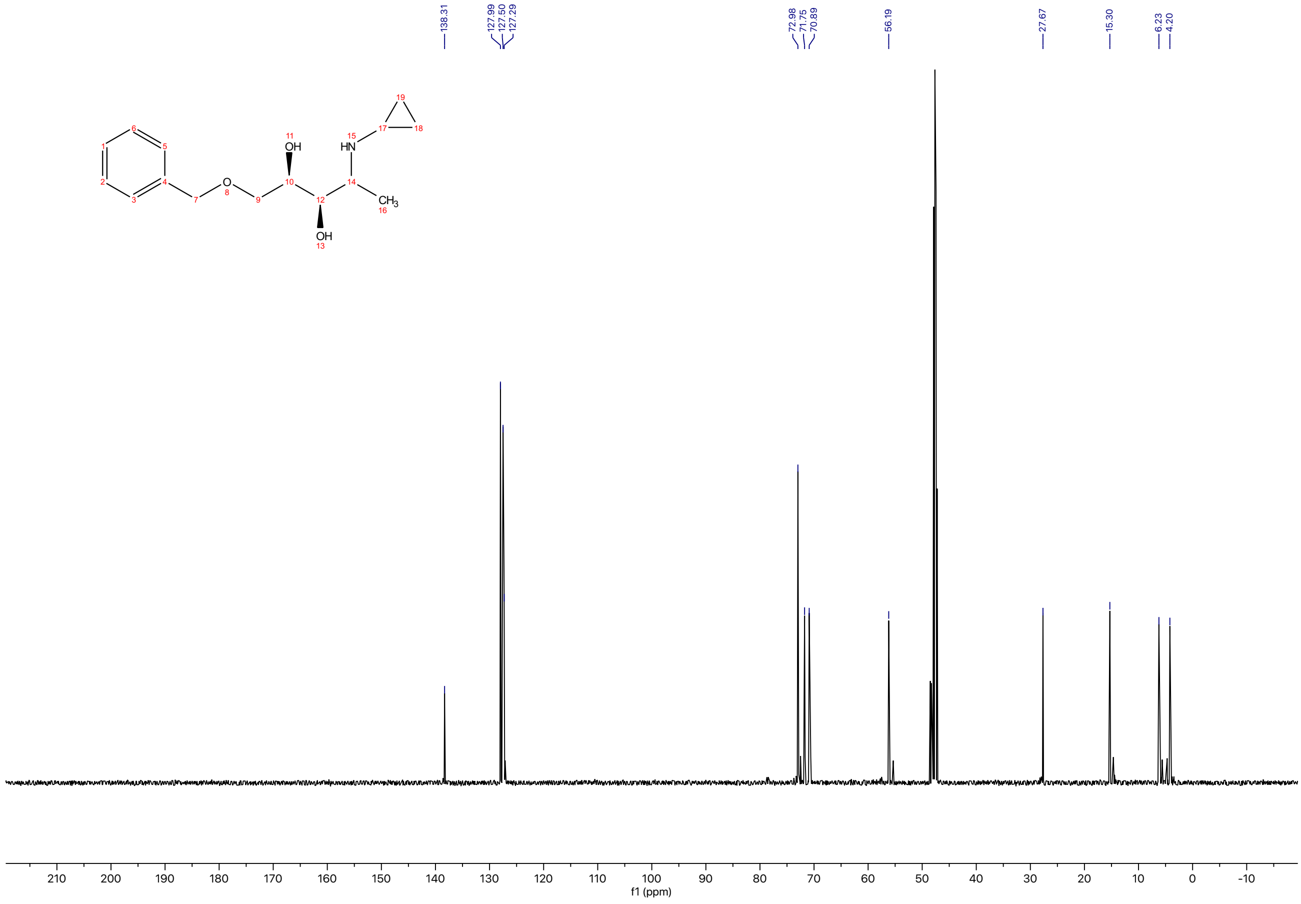

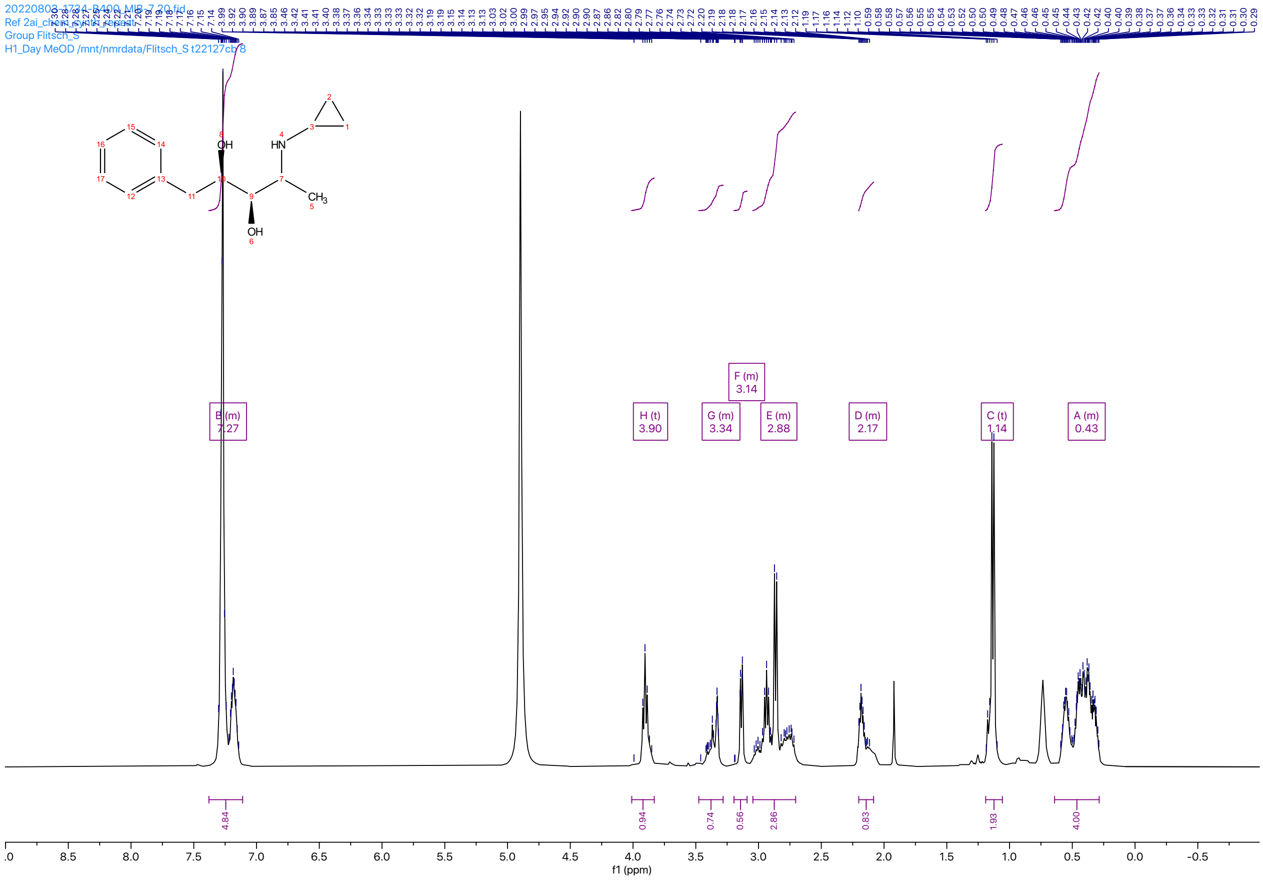

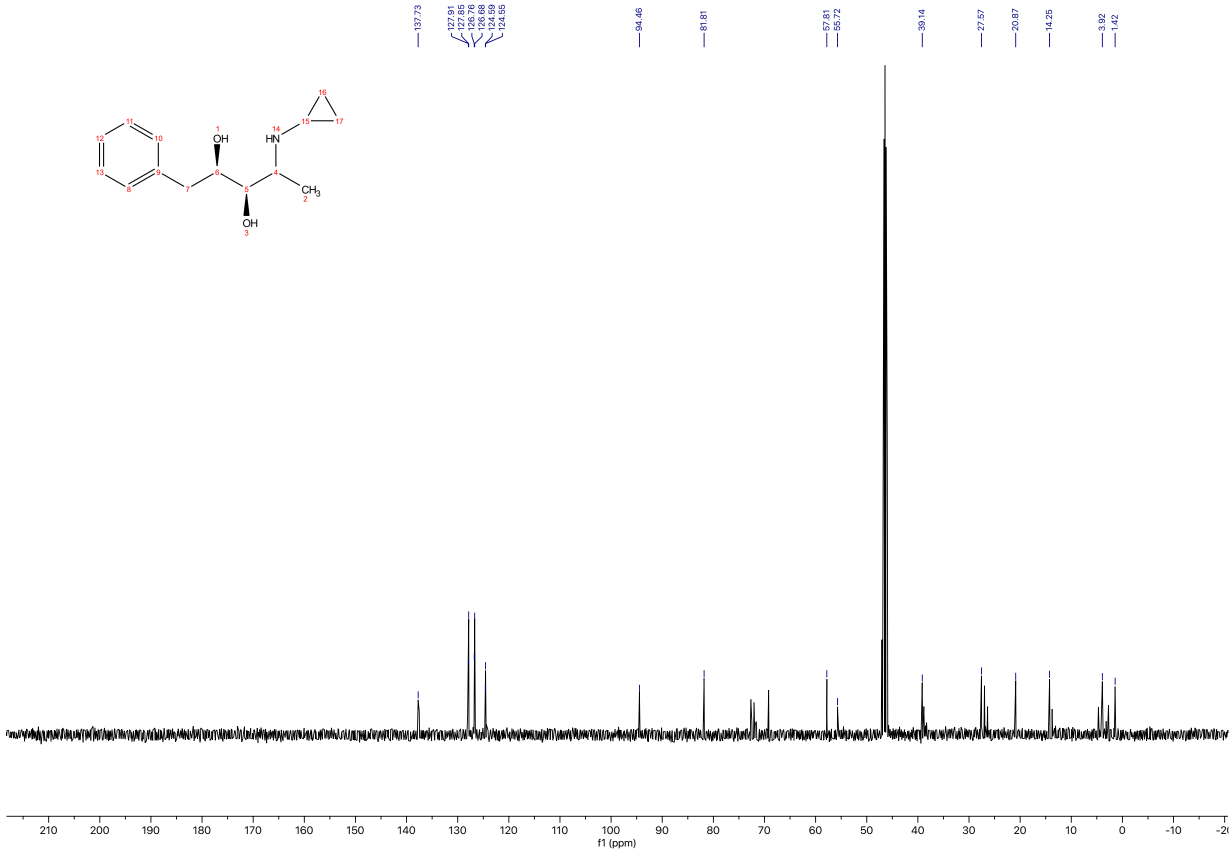

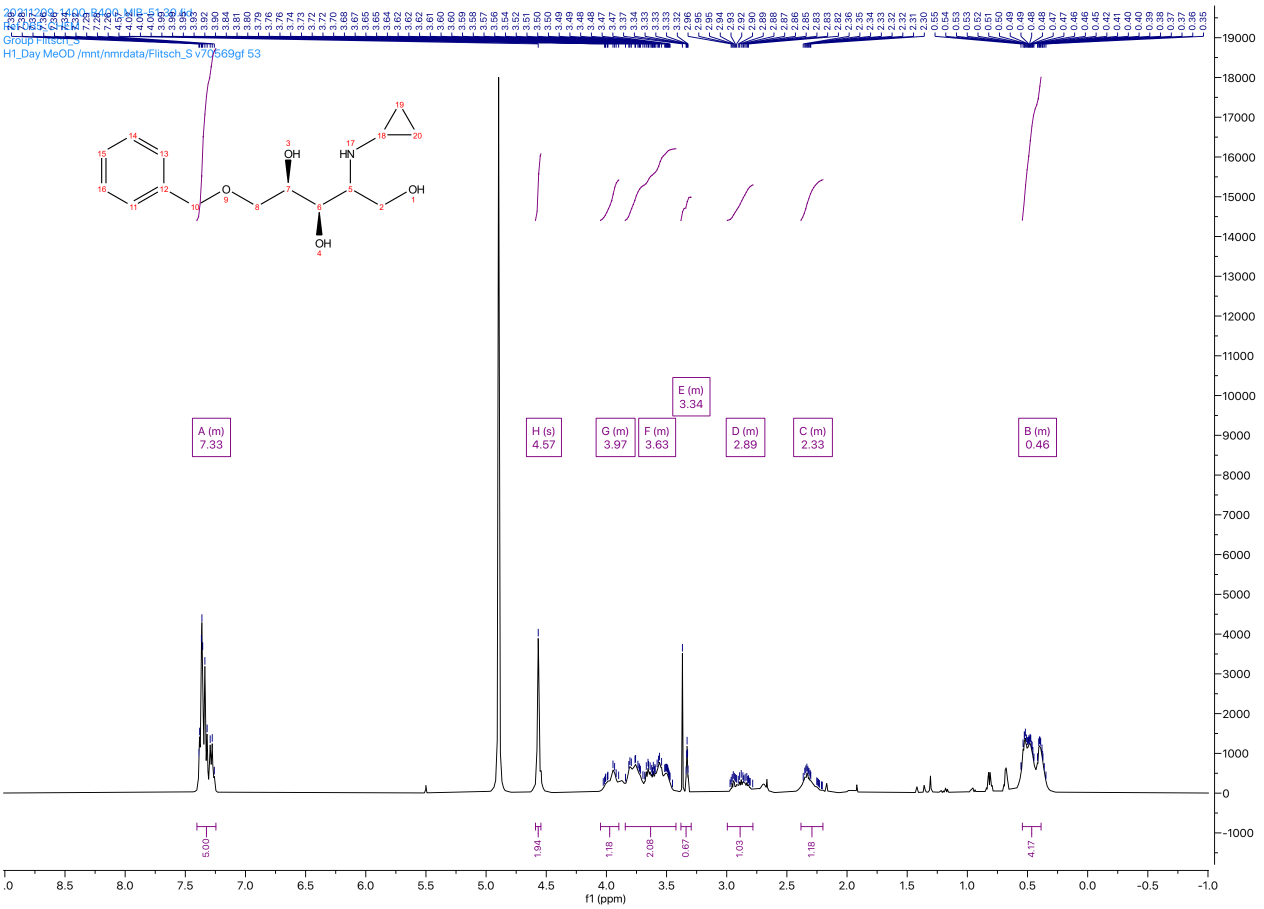

H1_Day MeOD /mnt/nmrdata/Flitsch_S v70569gf 53
A (m) 7.33
H (s) 4.57
G (m) 3.97
F (m) 3.63
E (m) 3.34
D (m) 2.89
C (m) 2.33
B (m) 0.46
5.00
1.94
1.18
2.08
0.67
1.03
1.18
4.17
f1 (ppm)

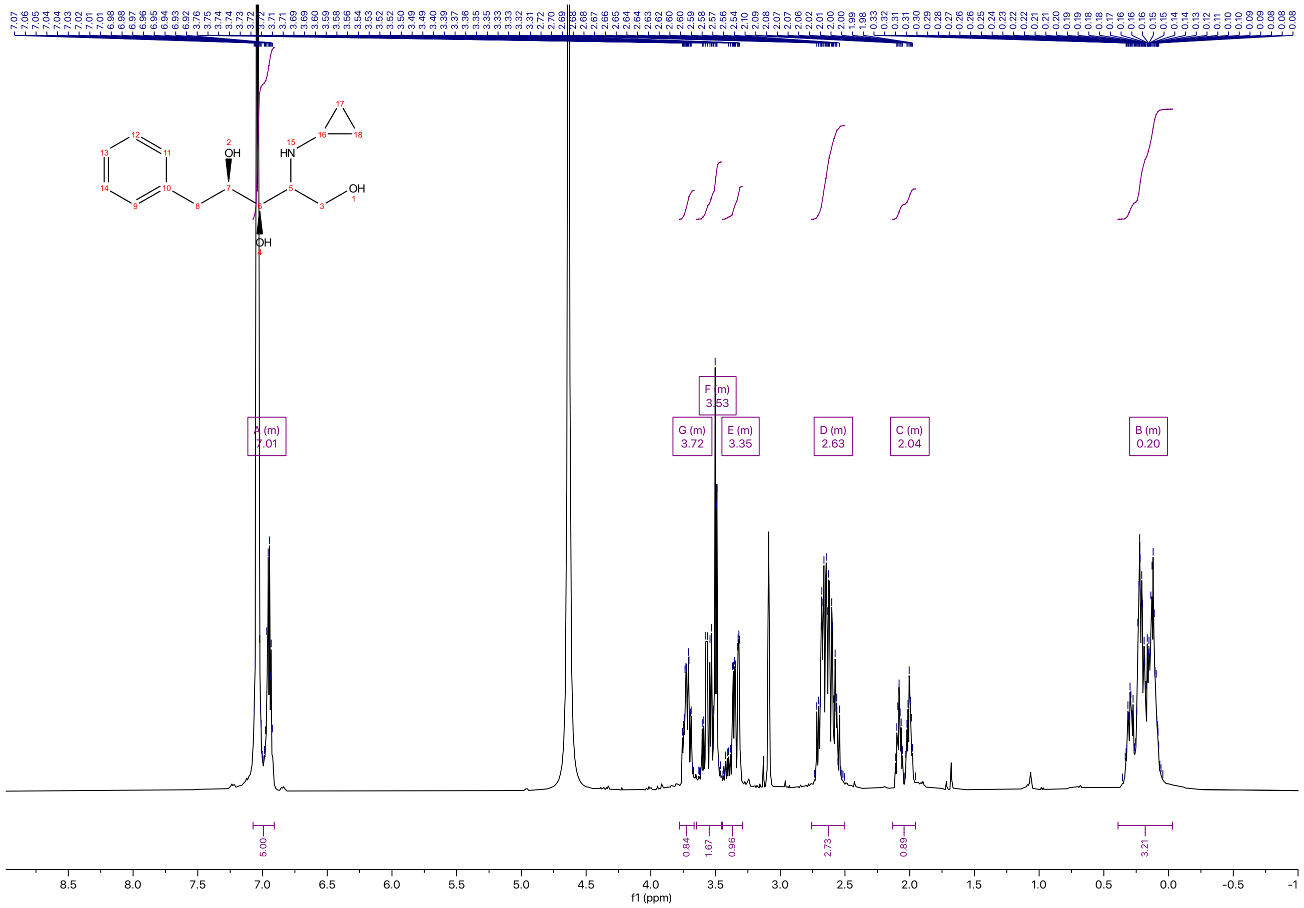

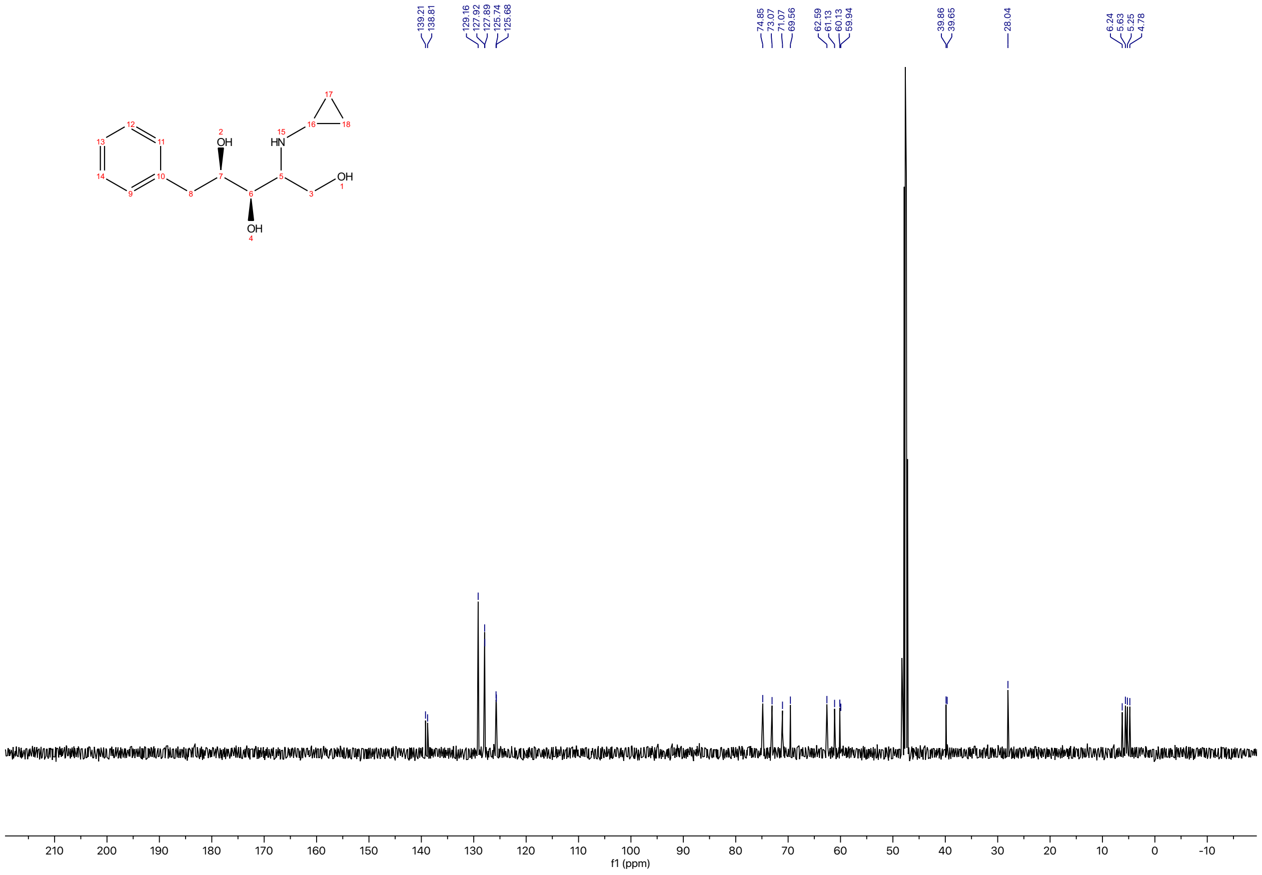

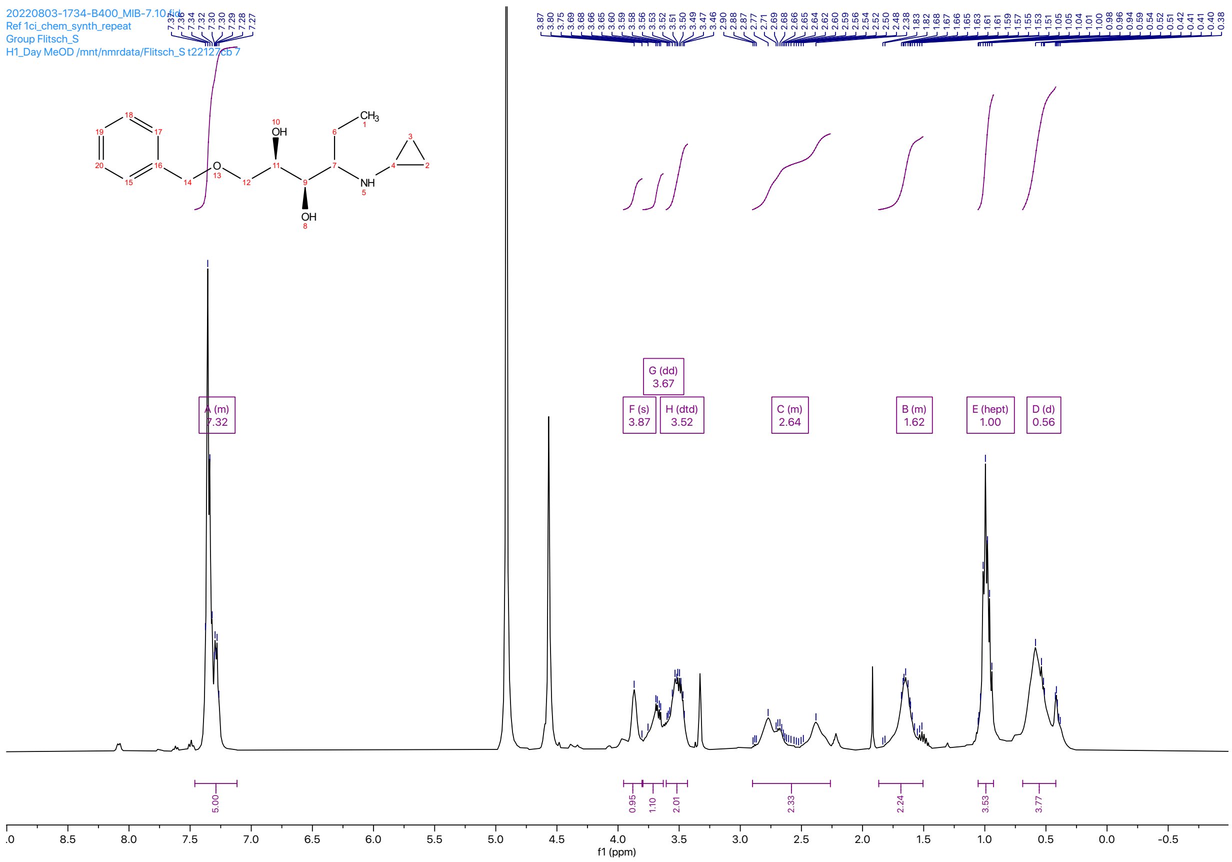

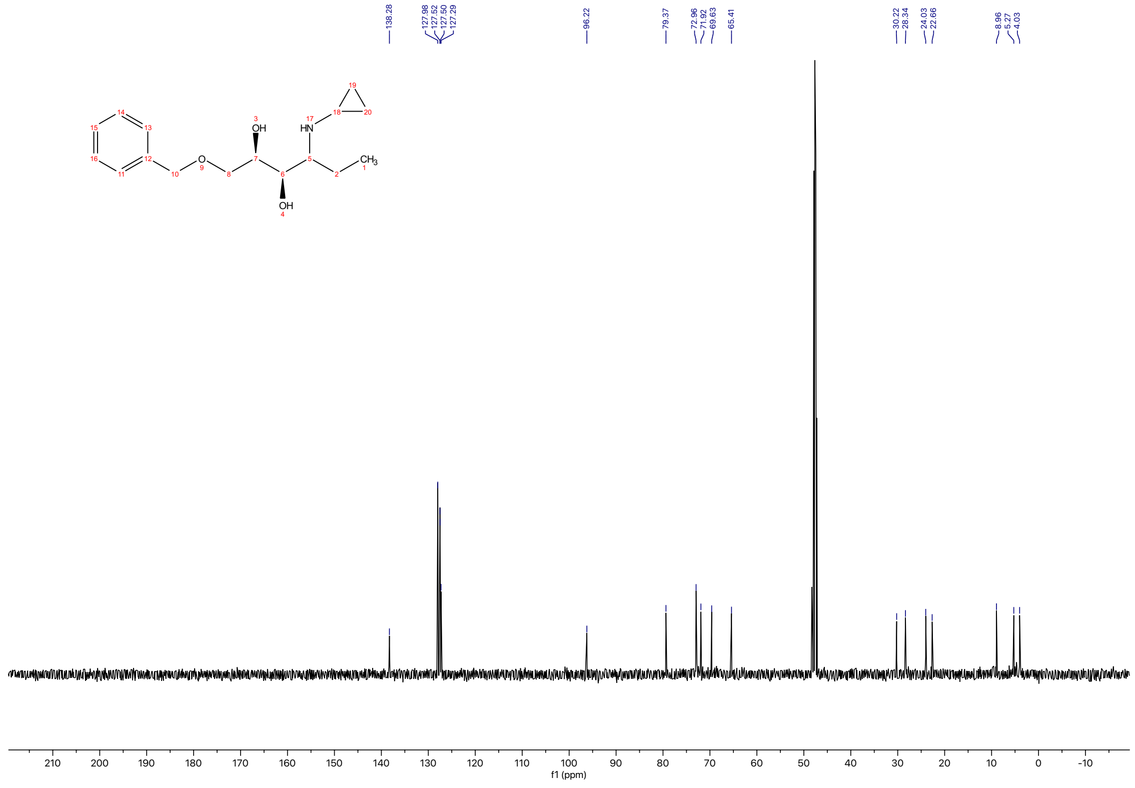

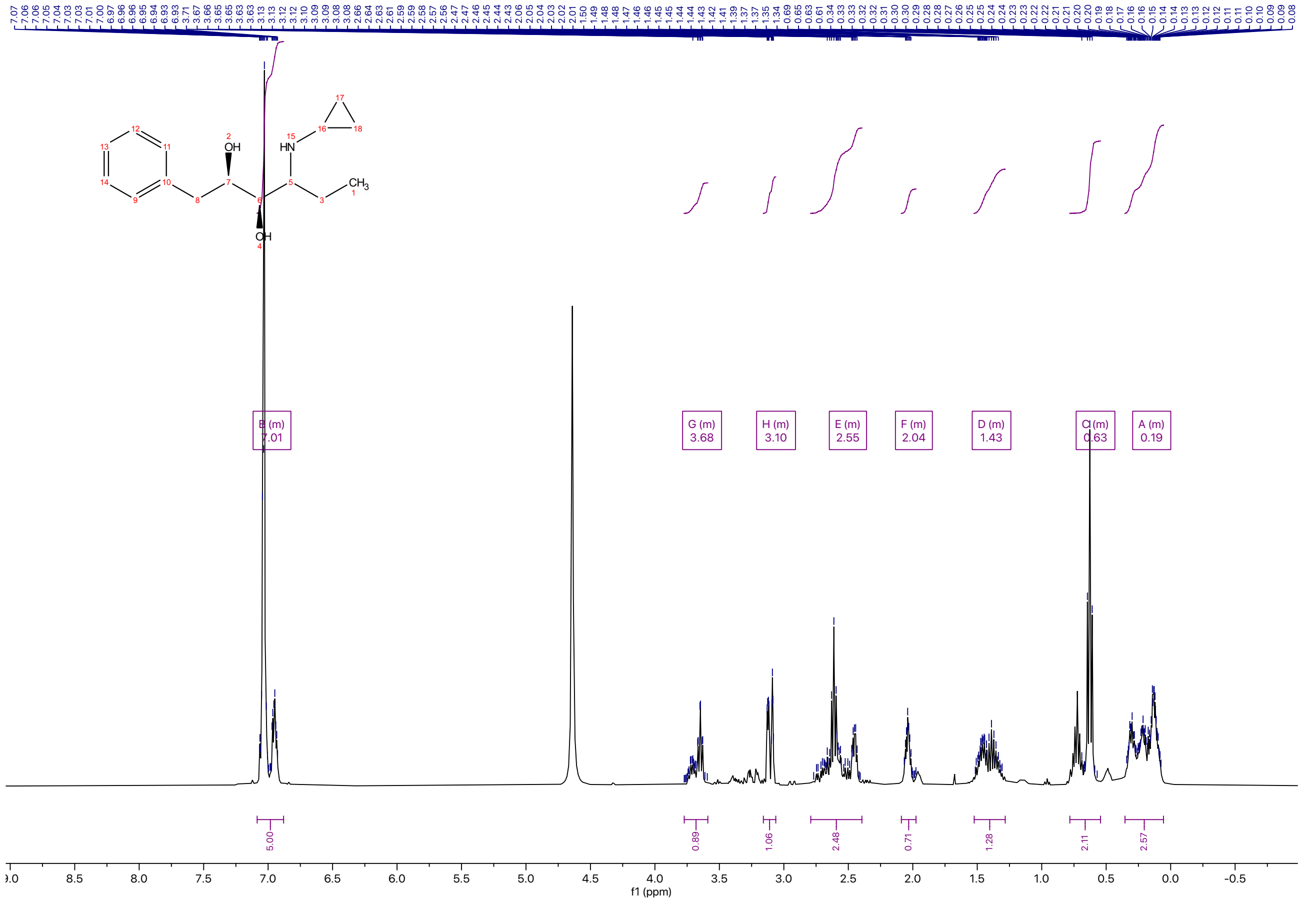

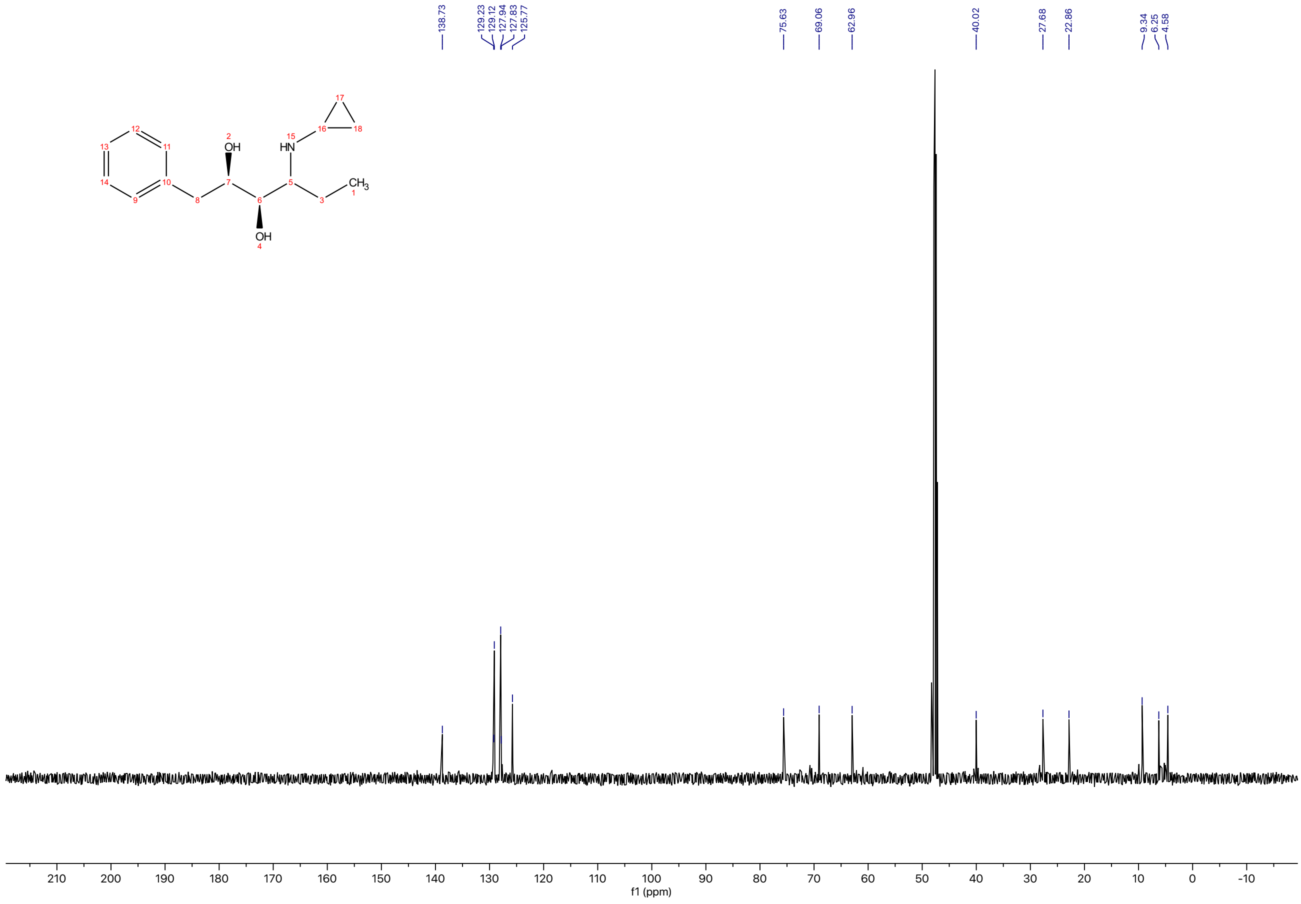

NMR Spectra of Biocatalytically synthesized compounds

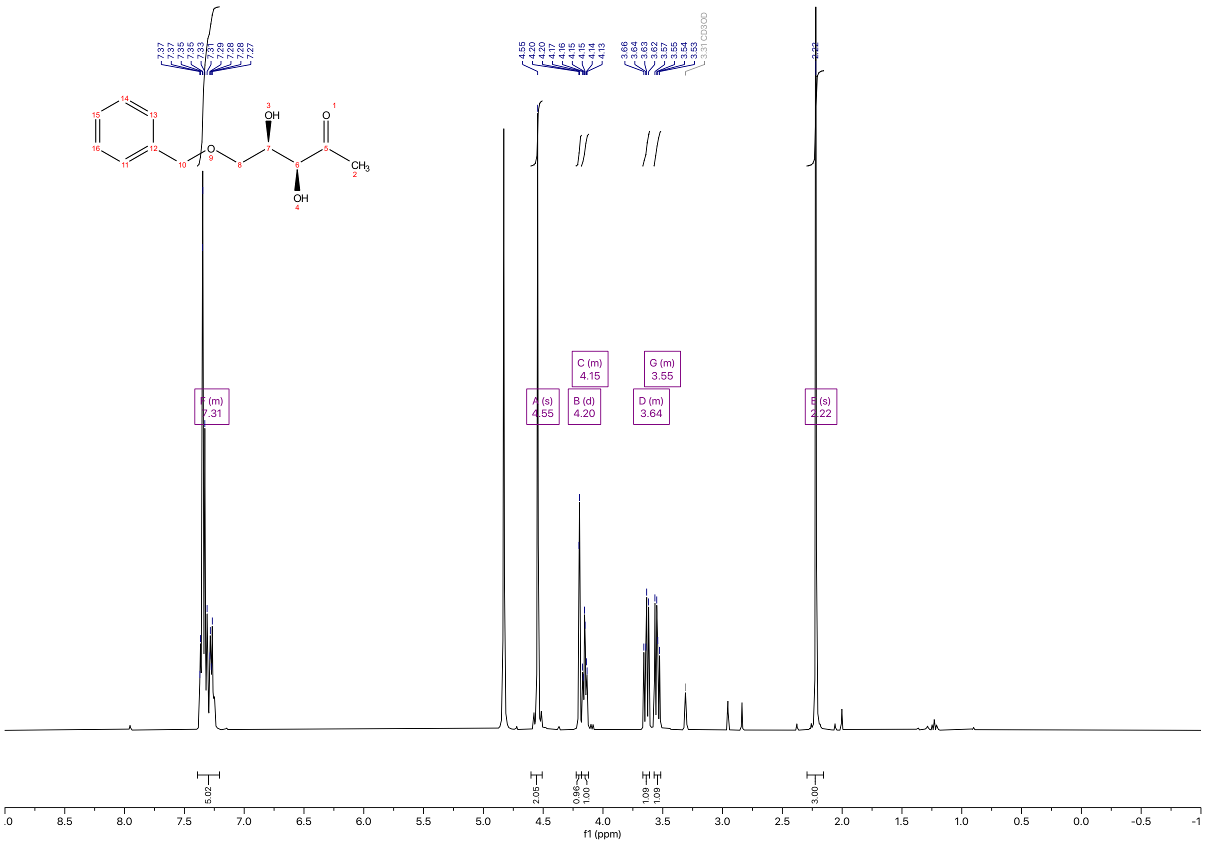

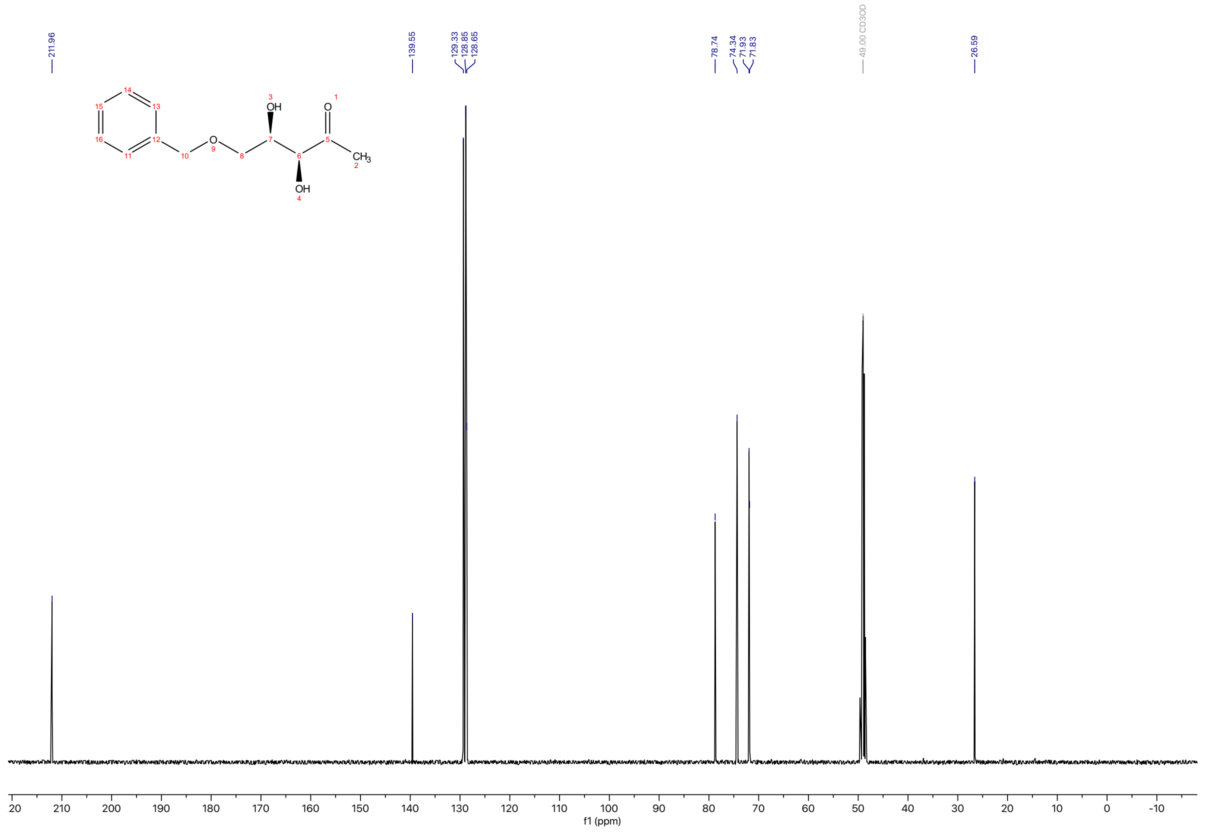

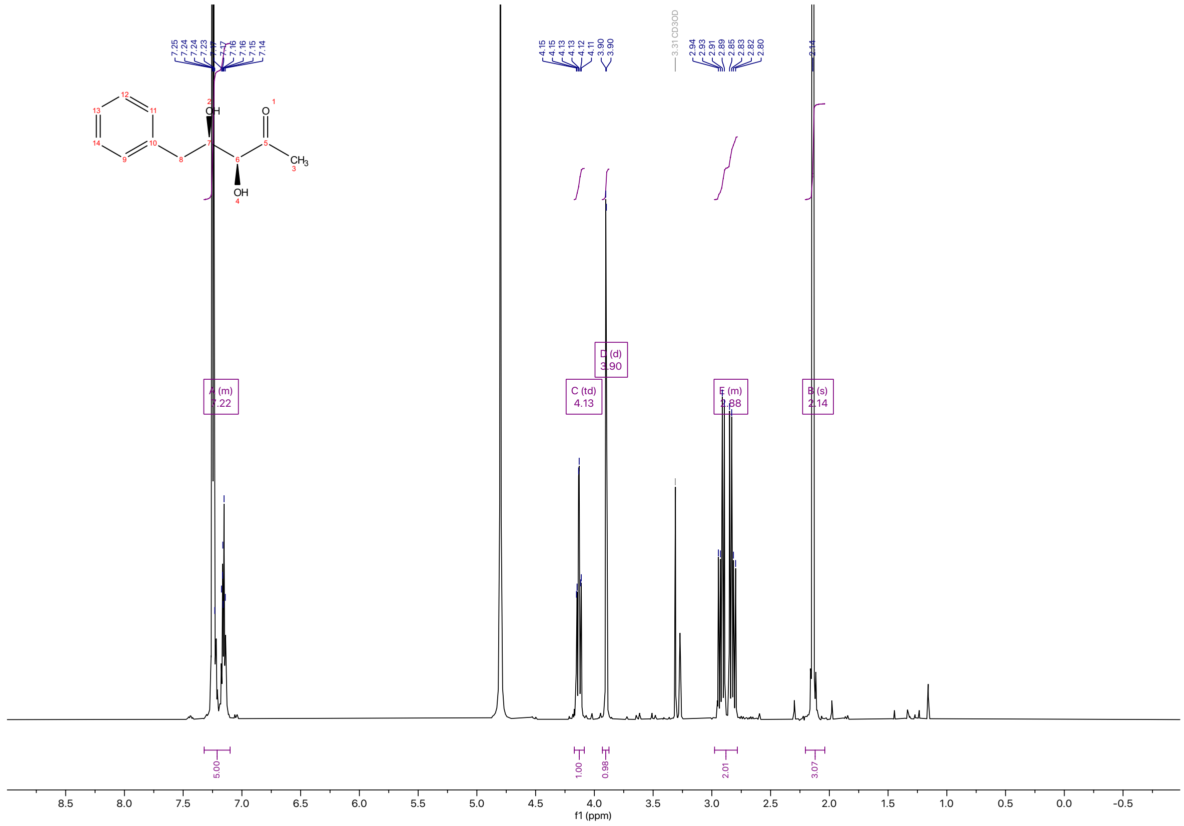

7.25
7.24
7.24
7.23
7.17
7.17
7.16
7.16
7.15
7.14
4.15
4.15
4.13
4.13
4.12
4.11
3.90
3.90
3.31 CD3OD
2.94
2.93
2.91
2.89
2.85
2.83
2.82
2.80
2.14
A (m)
7.22
C (td)
4.13
D (d)
3.90
E (m)
2.88
B (s)
2.14
5.00
1.00
0.98
2.01
3.07
f1 (ppm)

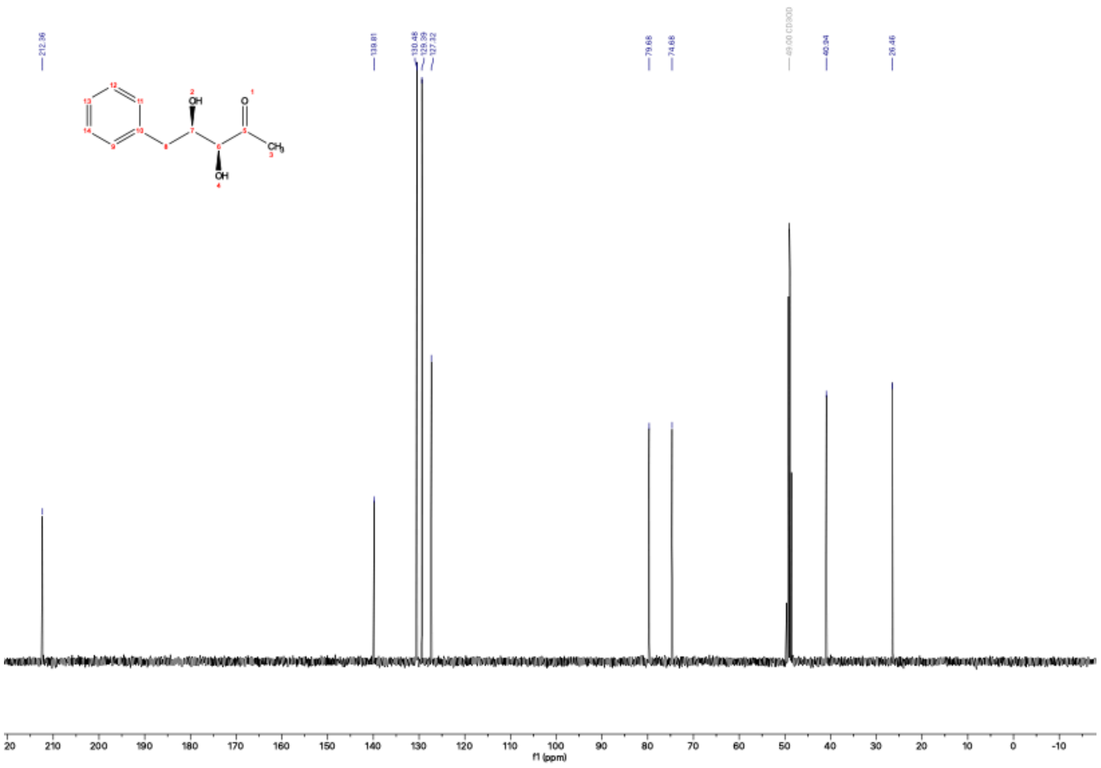

212.36
139.81
130.48
129.39
127.32
79.68
74.68
49.00 CD3OD
40.94
26.46
f1 (ppm)

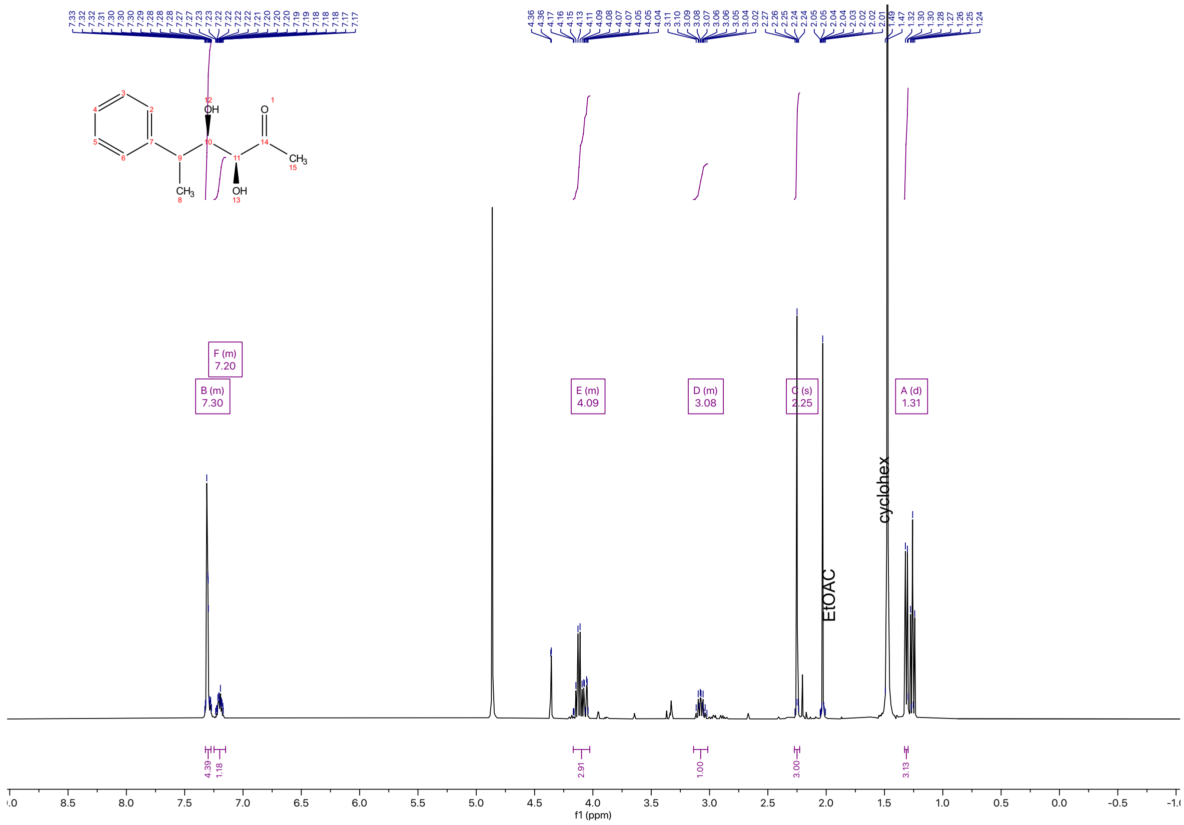
F (m)
7.20
B (m)
7.30
E (m)
4.09
D (m)
3.08
C (s)
2.25
A (d)
1.31
cyclohex
EtOAC
f1 (ppm)

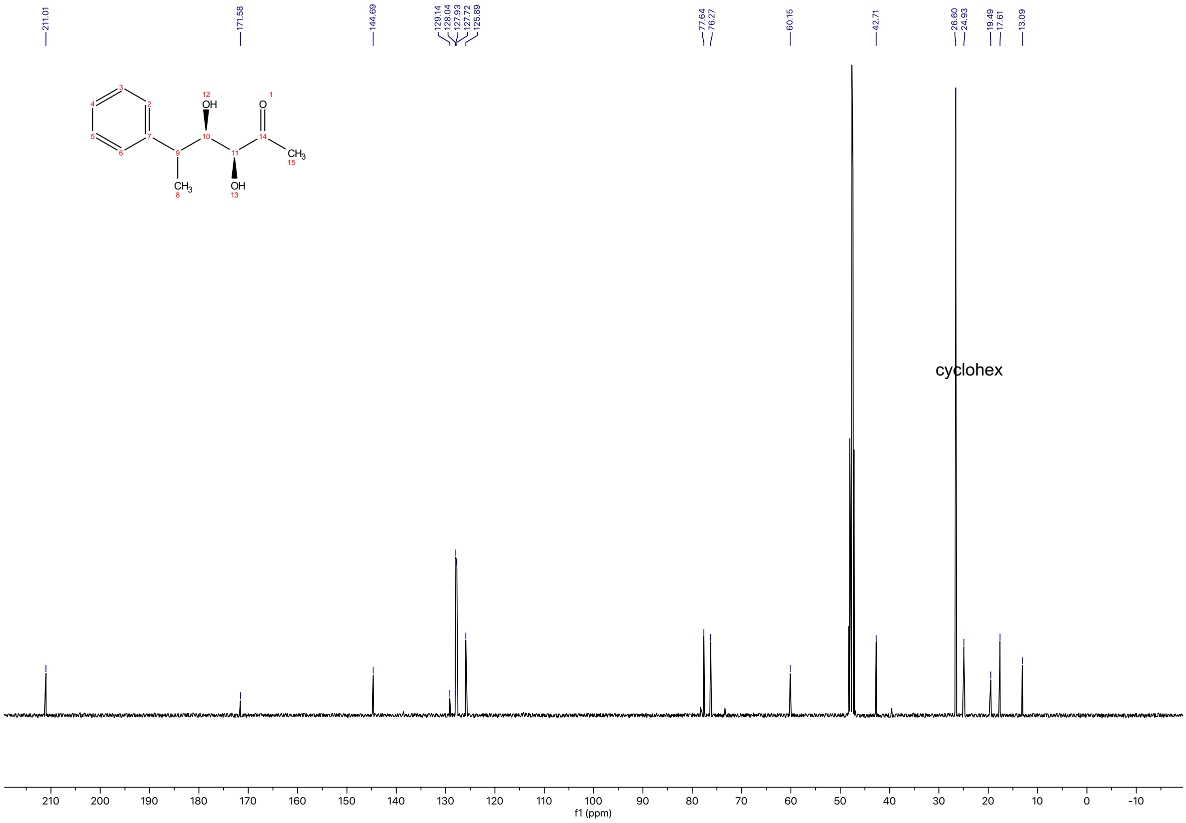
211.01
171.58
144.69
129.14
128.04
127.93
127.72
125.89
77.64
76.27
60.15
42.71
26.60
24.93
19.49
17.61
13.09
cyclohex
f1 (ppm)

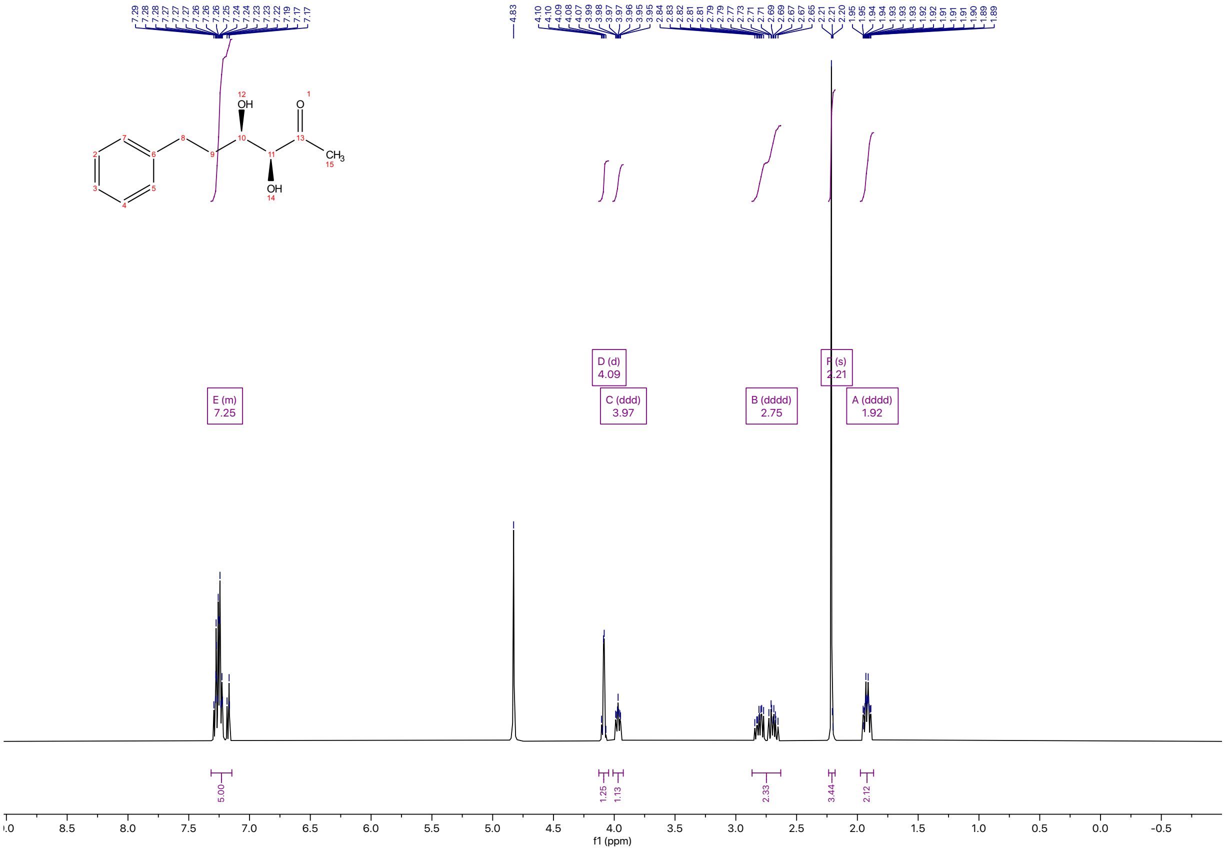

7.29 7.28 7.28 7.27 7.27 7.27 7.26 7.26 7.25 7.24 7.24 7.23 7.23 7.22 7.19 7.17 7.17
4.83
4.10 4.10 4.09 4.08 4.07 3.99 3.98 3.97 3.97 3.96 3.95 3.95 2.84 2.83 2.82 2.81 2.81 2.79 2.79 2.77 2.73 2.71 2.71 2.69 2.69 2.67 2.67 2.65 2.21 2.21 2.20 1.95 1.95 1.94 1.94 1.93 1.93 1.93 1.92 1.92 1.91 1.91 1.91 1.90 1.89 1.89
OH
O
CH3
OH
E (m)
7.25
D (d)
4.09
C (ddd)
3.97
B (dddd)
2.75
F (s)
2.21
A (dddd)
1.92
5.00
1.25
1.13
2.33
3.44
2.12
f1 (ppm)

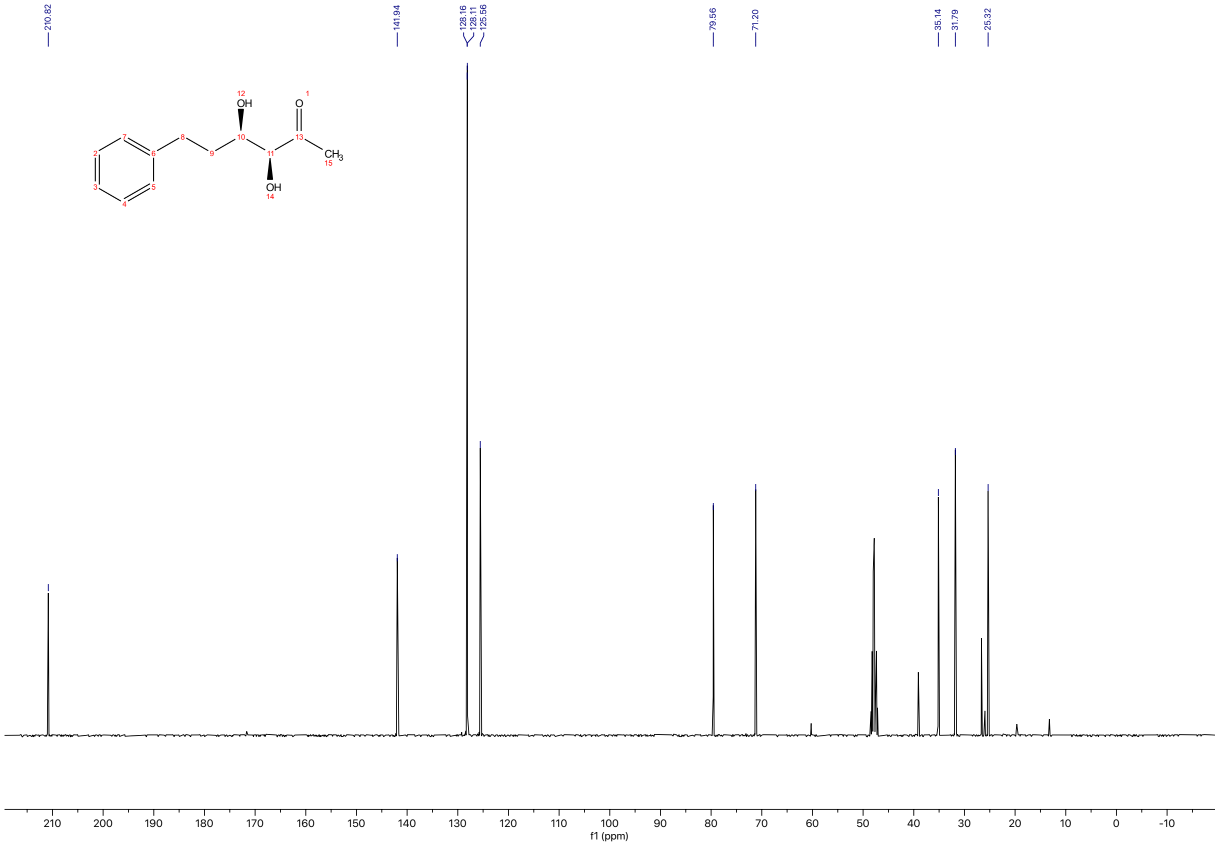

210.82
141.94
128.16
128.11
125.56
79.56
71.20
35.14
31.79
25.32
OH
O
CH3
OH
f1 (ppm)

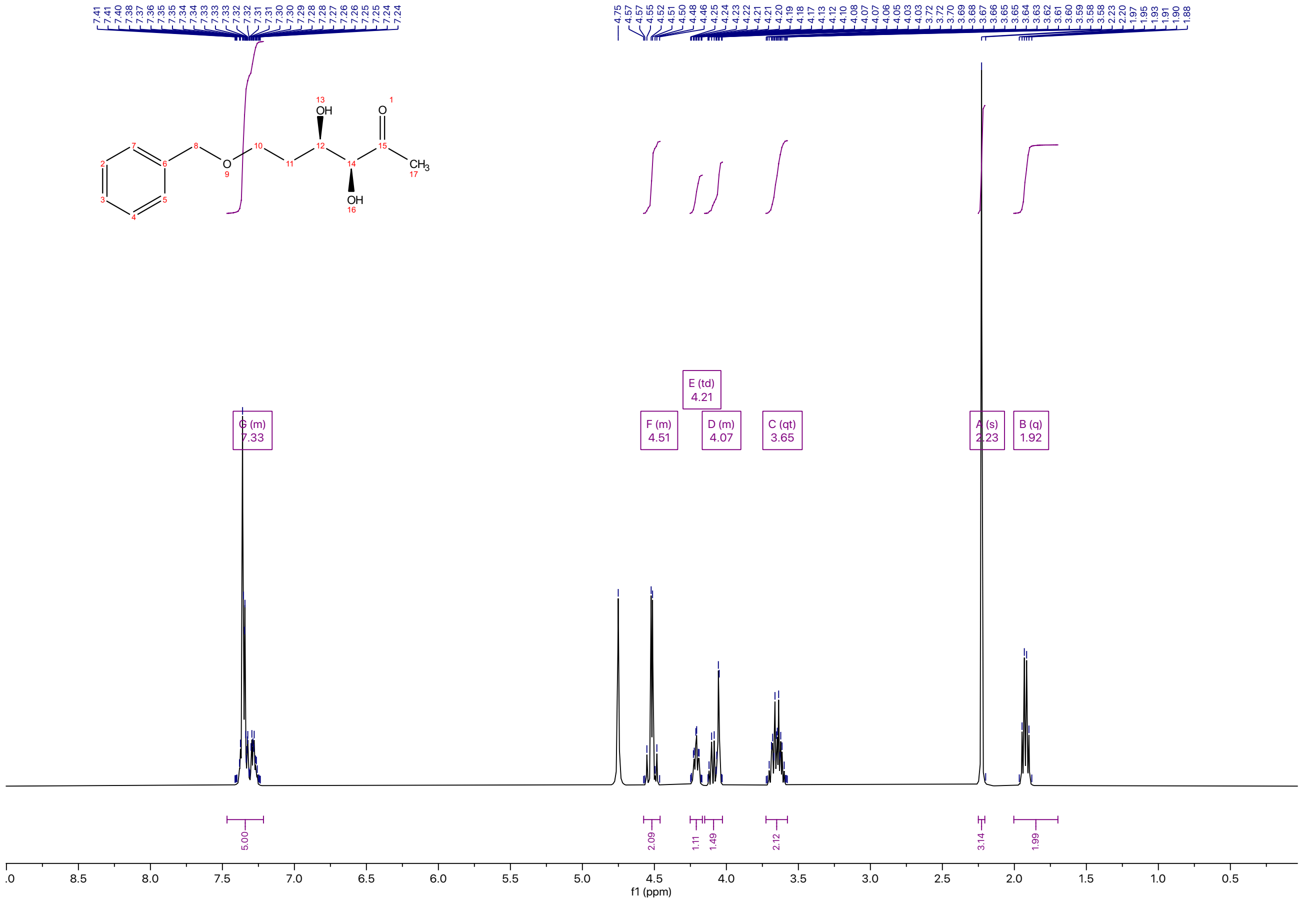
G (m)
7.33
F (m)
4.51
E (td)
4.21
D (m)
4.07
C (qt)
3.65
A (s)
2.23
B (q)
1.92
5.00
2.09
1.11
1.49
2.12
3.14
1.99
f1 (ppm)

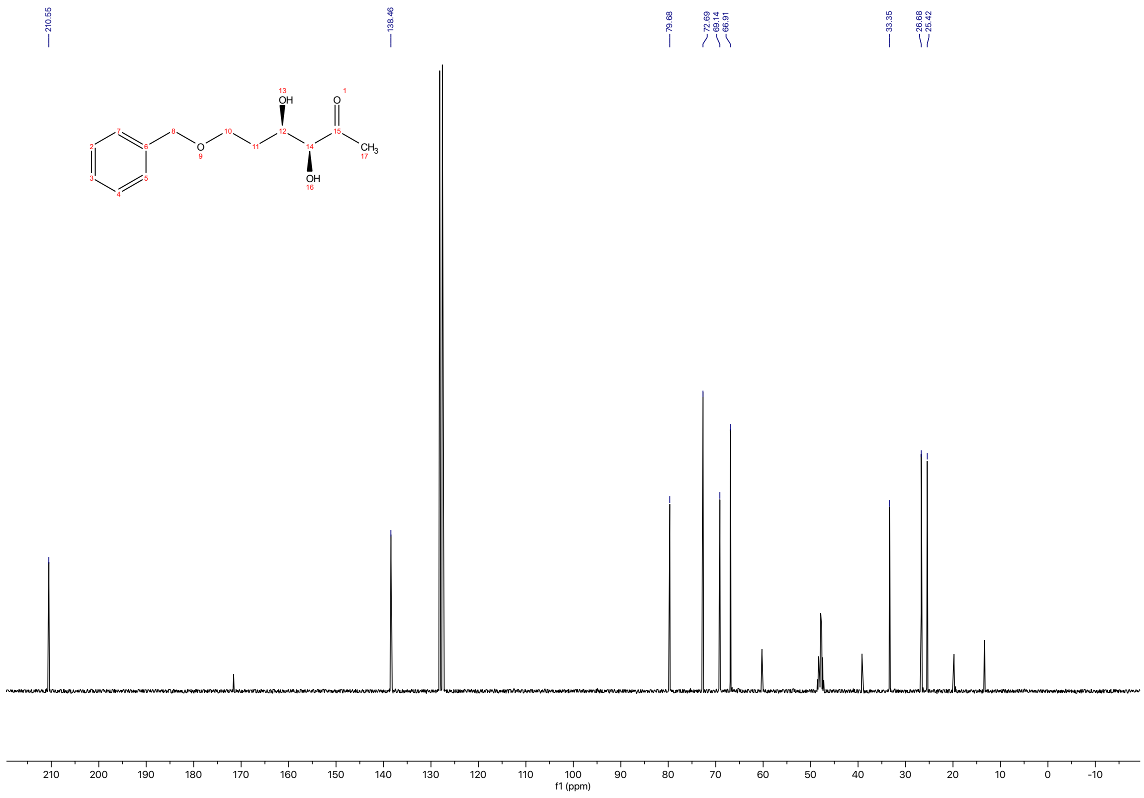
210.55
138.46
79.68
72.69
69.14
66.91
33.35
26.68
25.42
f1 (ppm)

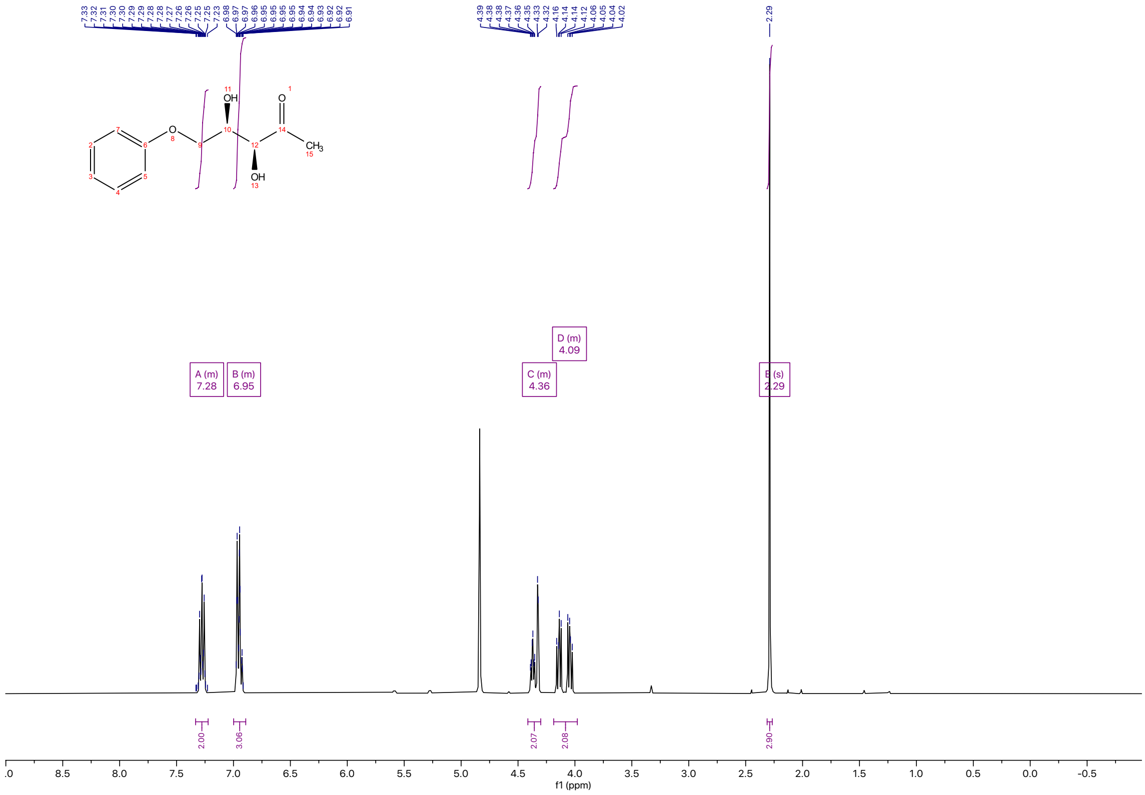

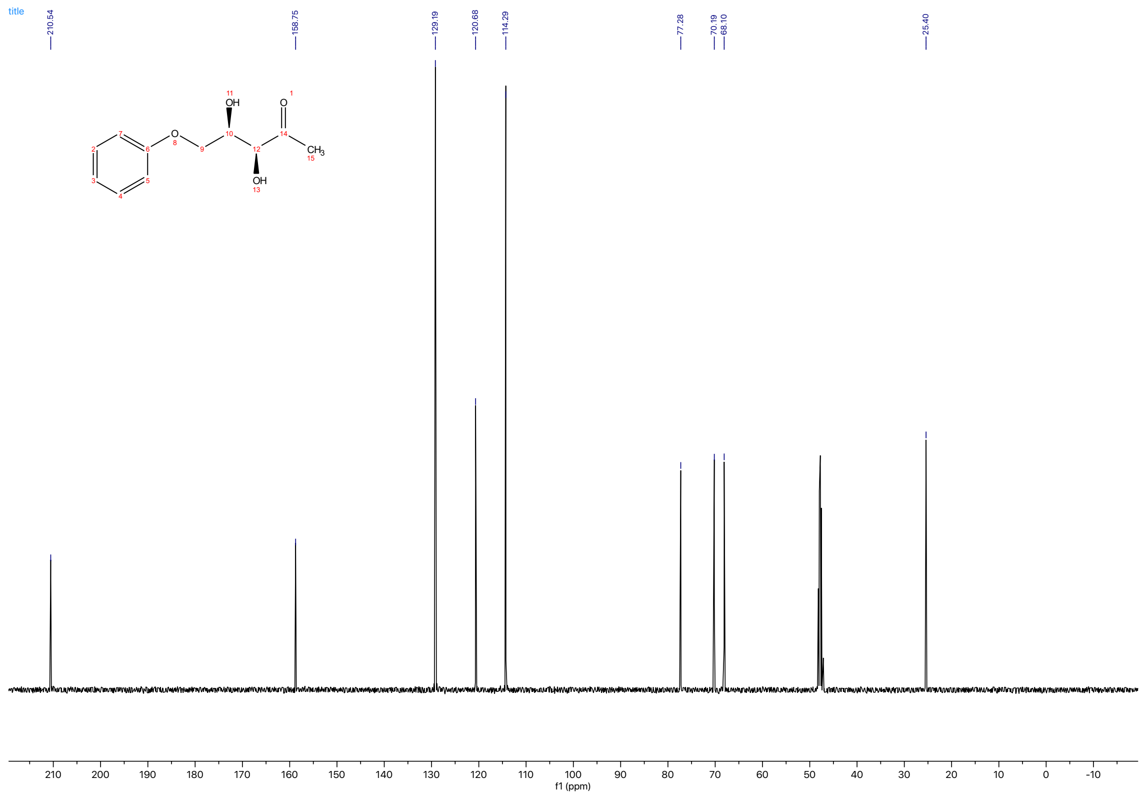

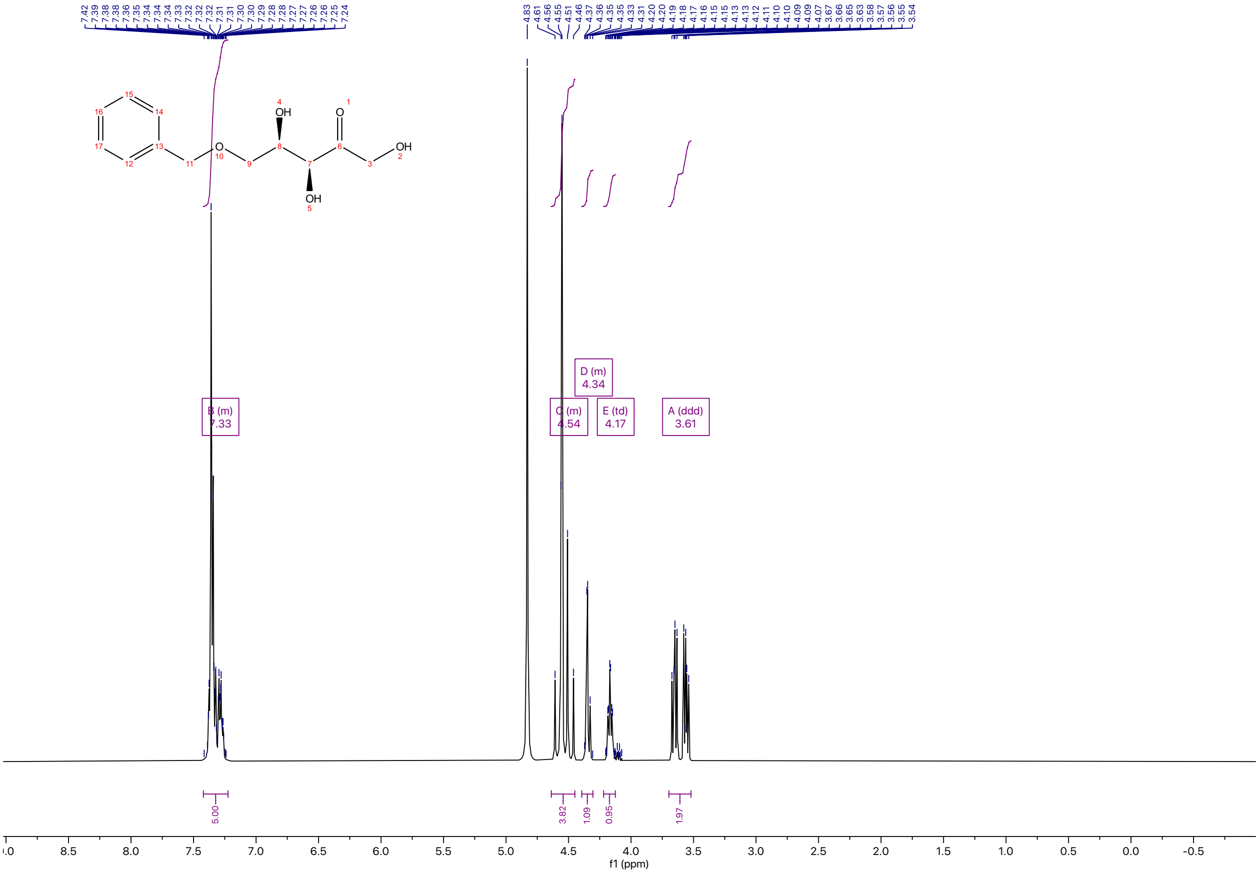
B (m)
7.33
C (m)
4.54
D (m)
4.34
E (td)
4.17
A (ddd)
3.61
5.00
3.82
1.09
0.95
1.97
f1 (ppm)

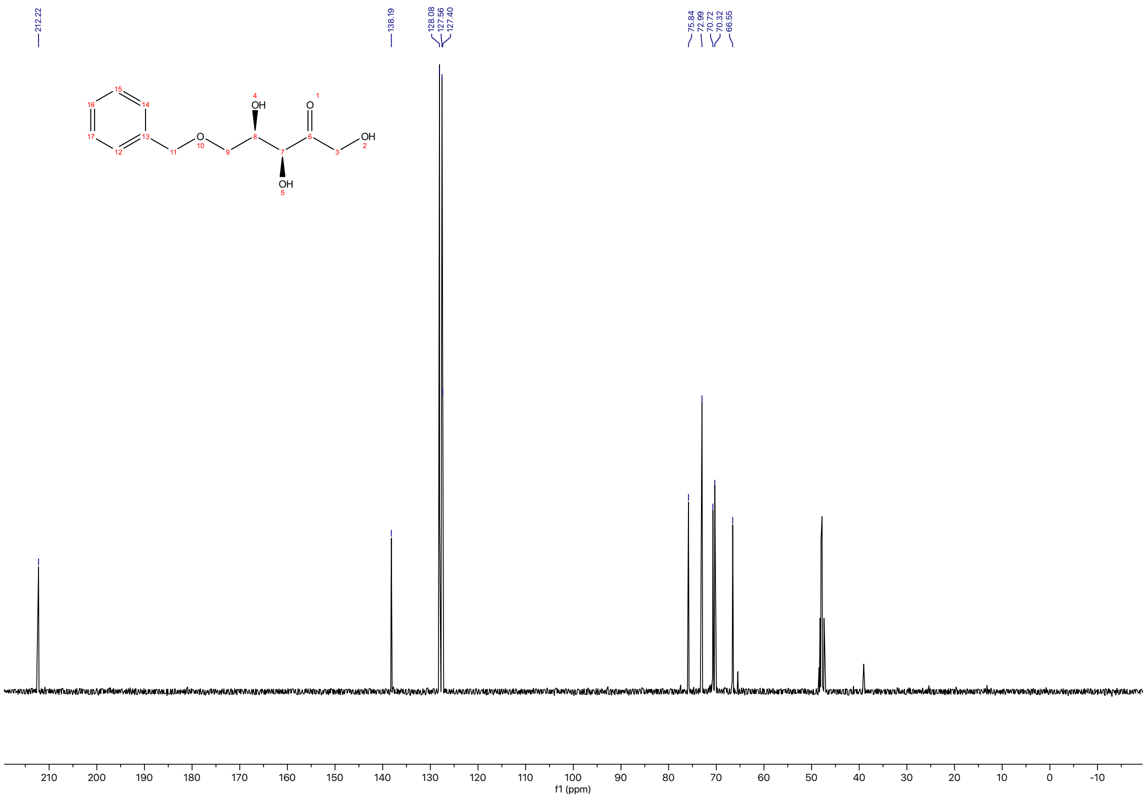
212.22
138.19
128.08
127.56
127.40
75.84
72.99
70.72
70.32
66.55
f1 (ppm)

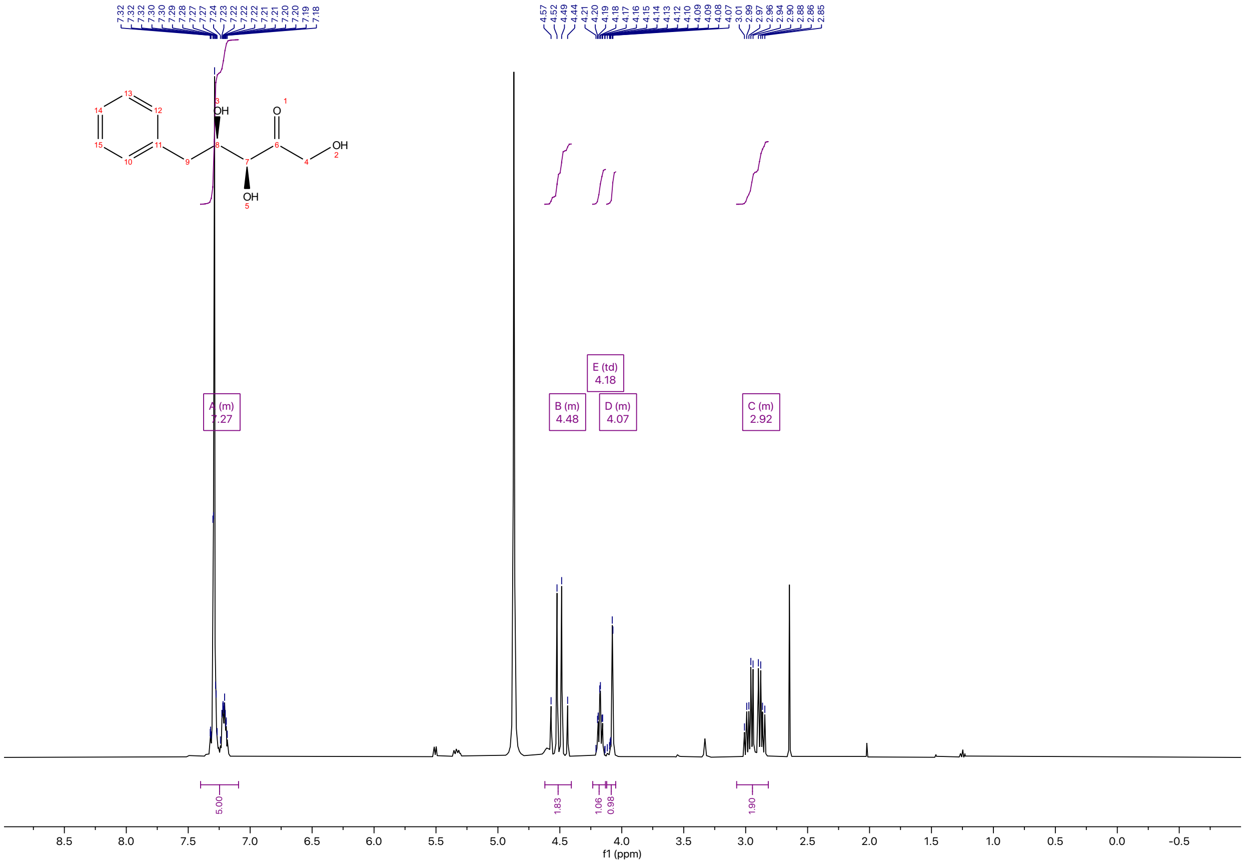
A (m)
7.27
B (m)
4.48
E (td)
4.18
D (m)
4.07
C (m)
2.92
5.00
1.83
1.06
0.98
1.90
f1 (ppm)

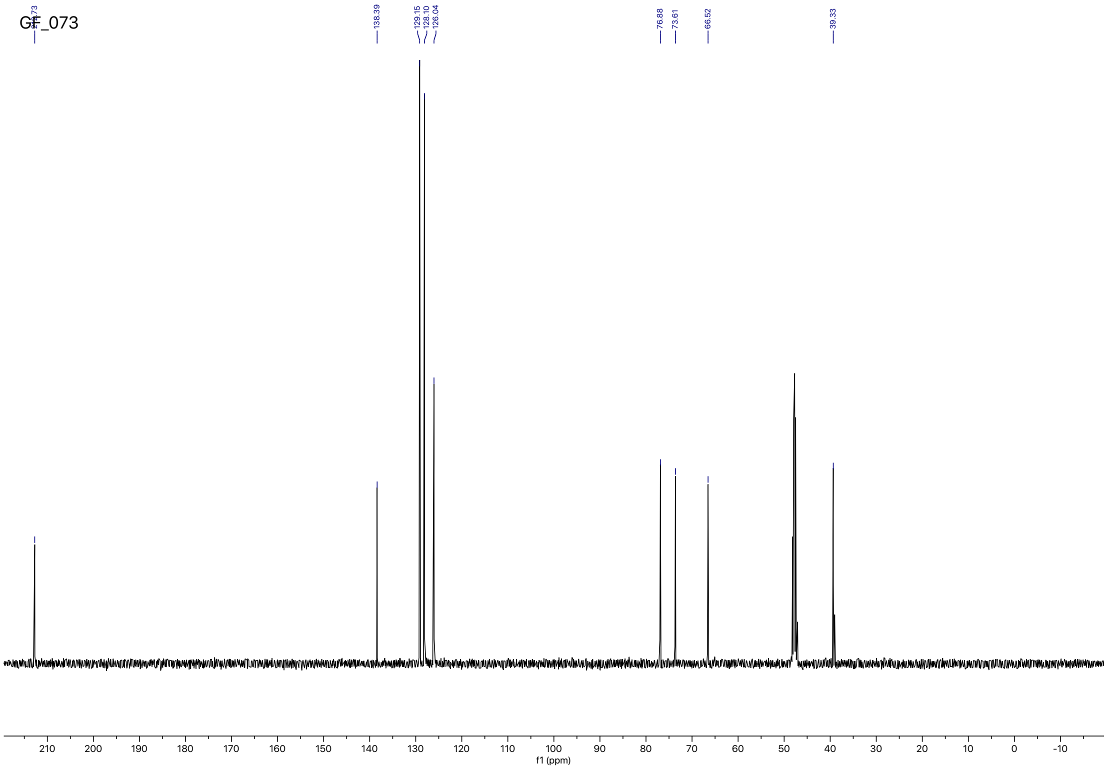
GF_073
138.39
129.15
128.10
126.04
76.88
73.61
66.52
39.33
f1 (ppm)

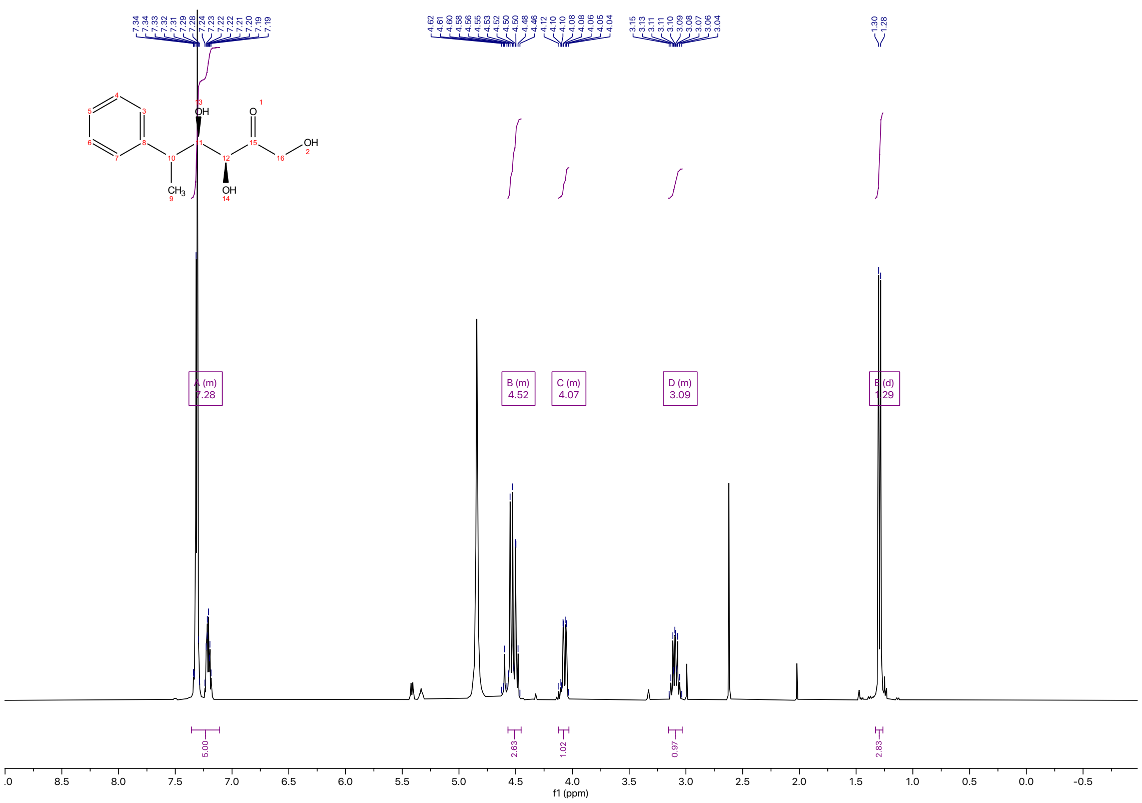

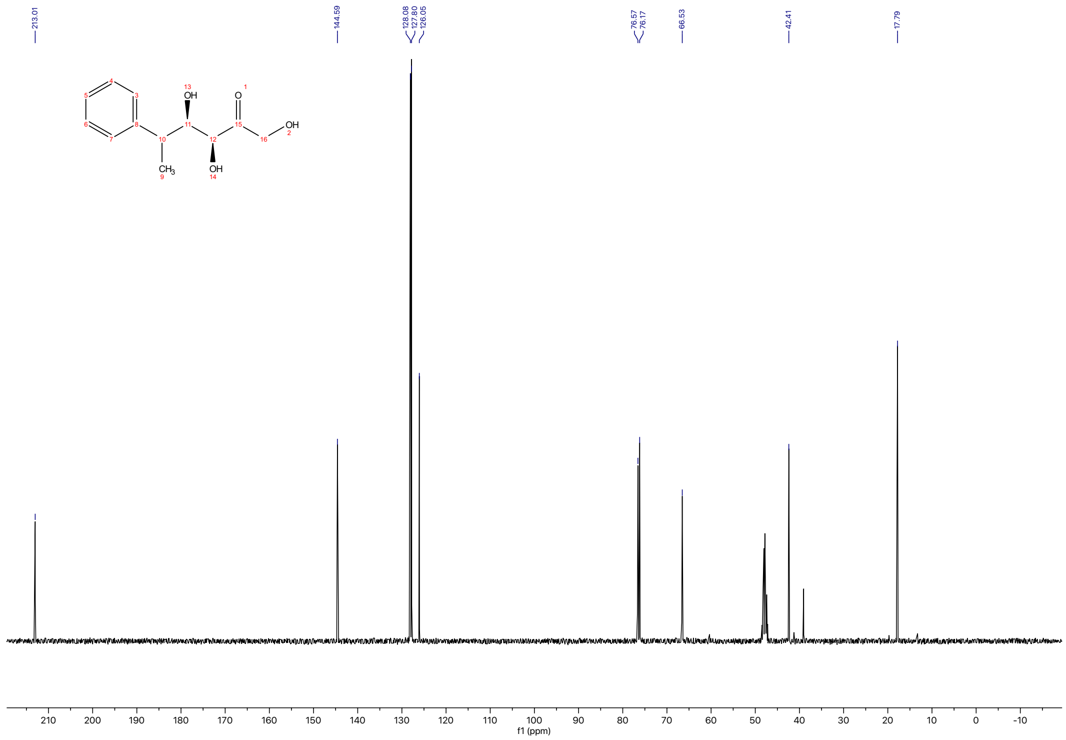

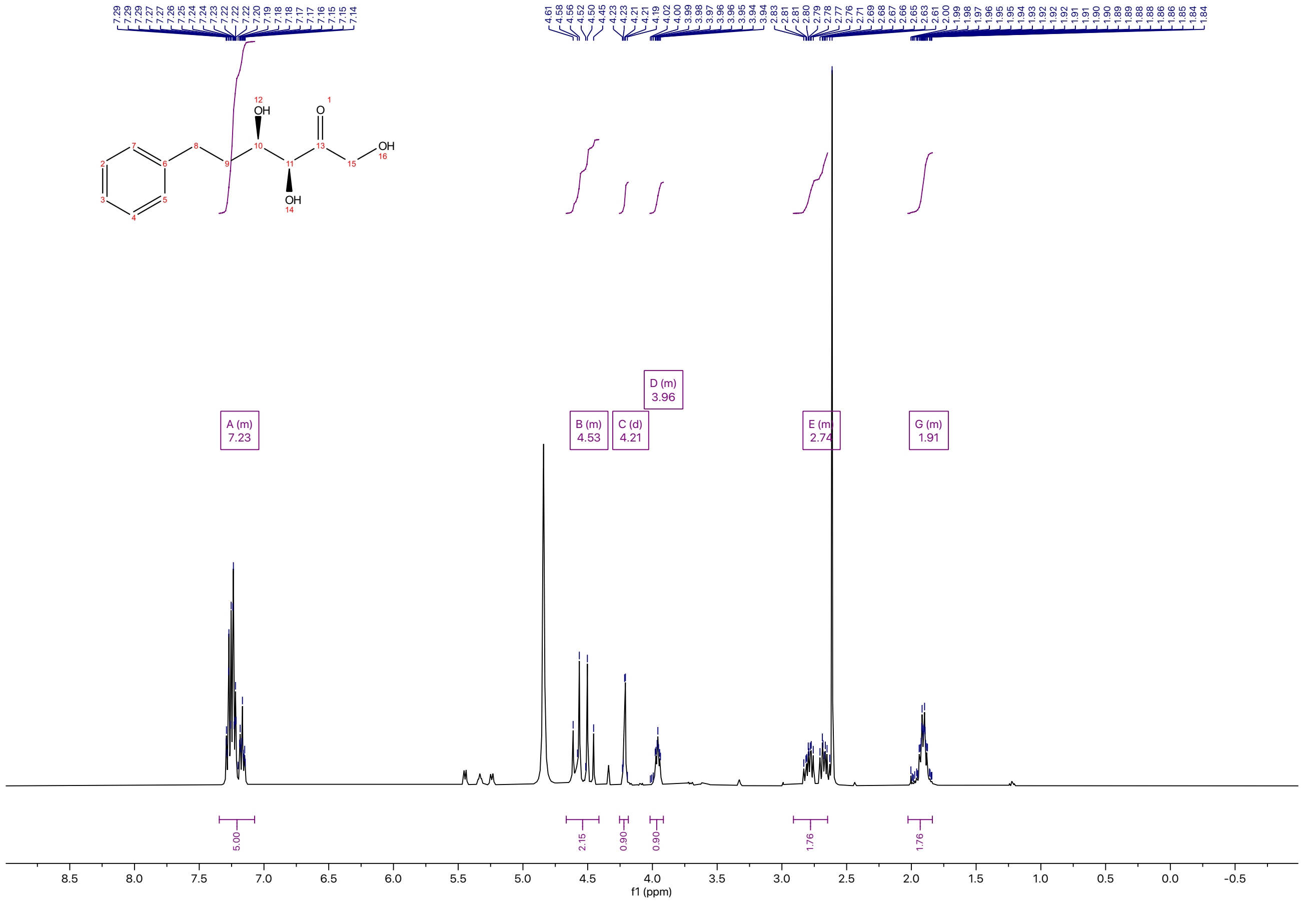

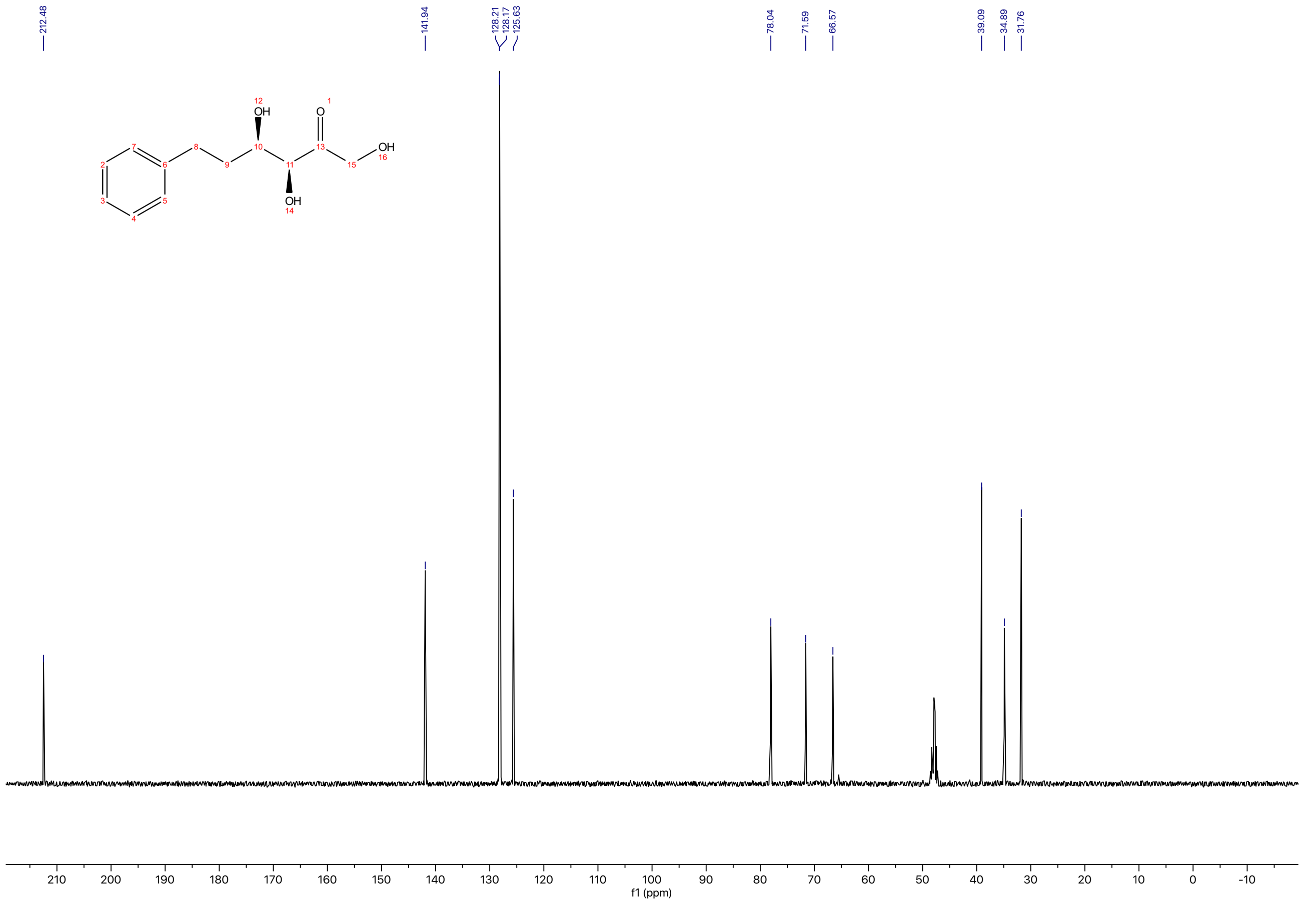

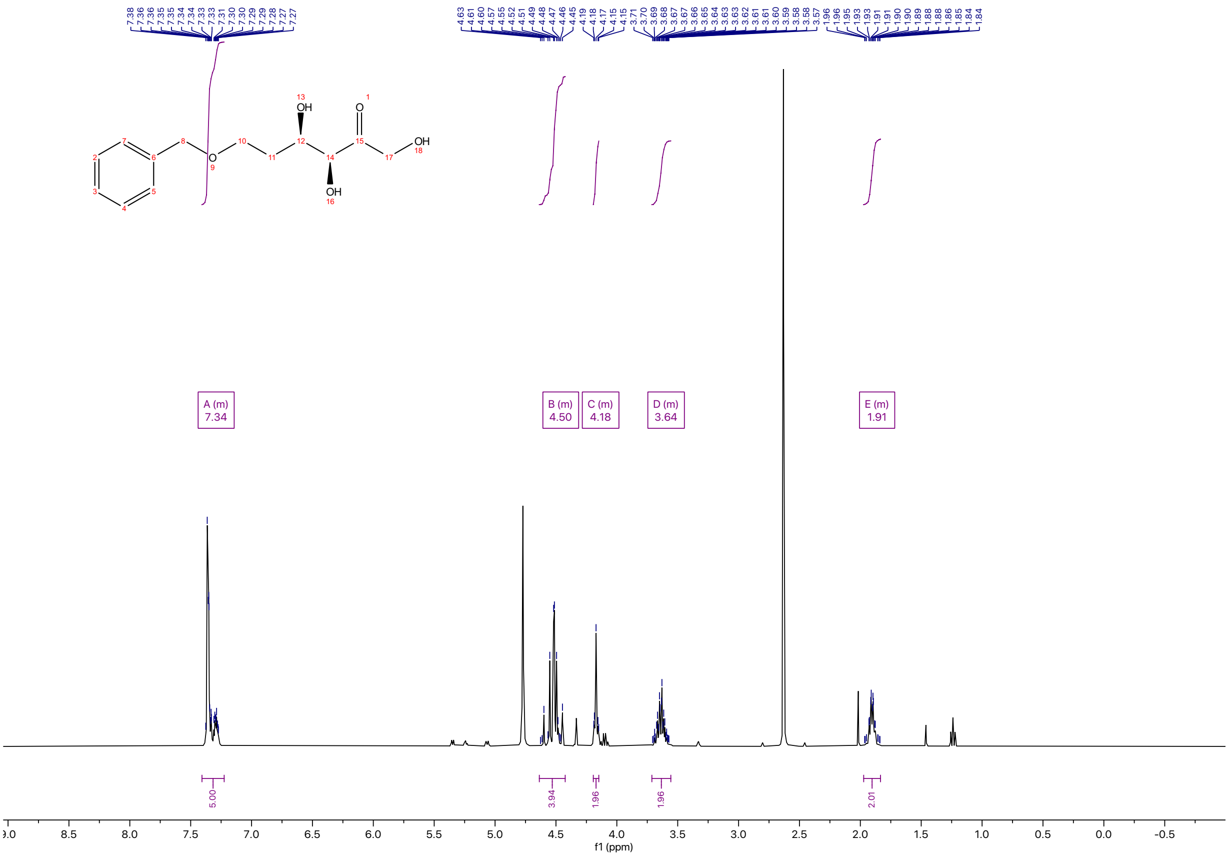
7.38 7.36 7.36 7.35 7.35 7.34 7.34 7.33 7.33 7.31 7.30 7.30 7.29 7.29 7.28 7.27 7.27
4.63 4.61 4.60 4.57 4.55 4.52 4.51 4.49 4.48 4.47 4.46 4.45 4.19 4.18 4.17 4.15 4.15 3.71 3.70 3.69 3.68 3.67 3.67 3.66 3.65 3.64 3.63 3.63 3.62 3.61 3.61 3.60 3.59 3.58 3.58 3.57 1.96 1.96 1.95 1.93 1.93 1.91 1.91 1.90 1.90 1.89 1.88 1.88 1.86 1.85 1.84 1.84
A (m) 7.34
B (m) 4.50
C (m) 4.18
D (m) 3.64
E (m) 1.91
5.00
3.94
1.96
1.96
2.01
f1 (ppm)

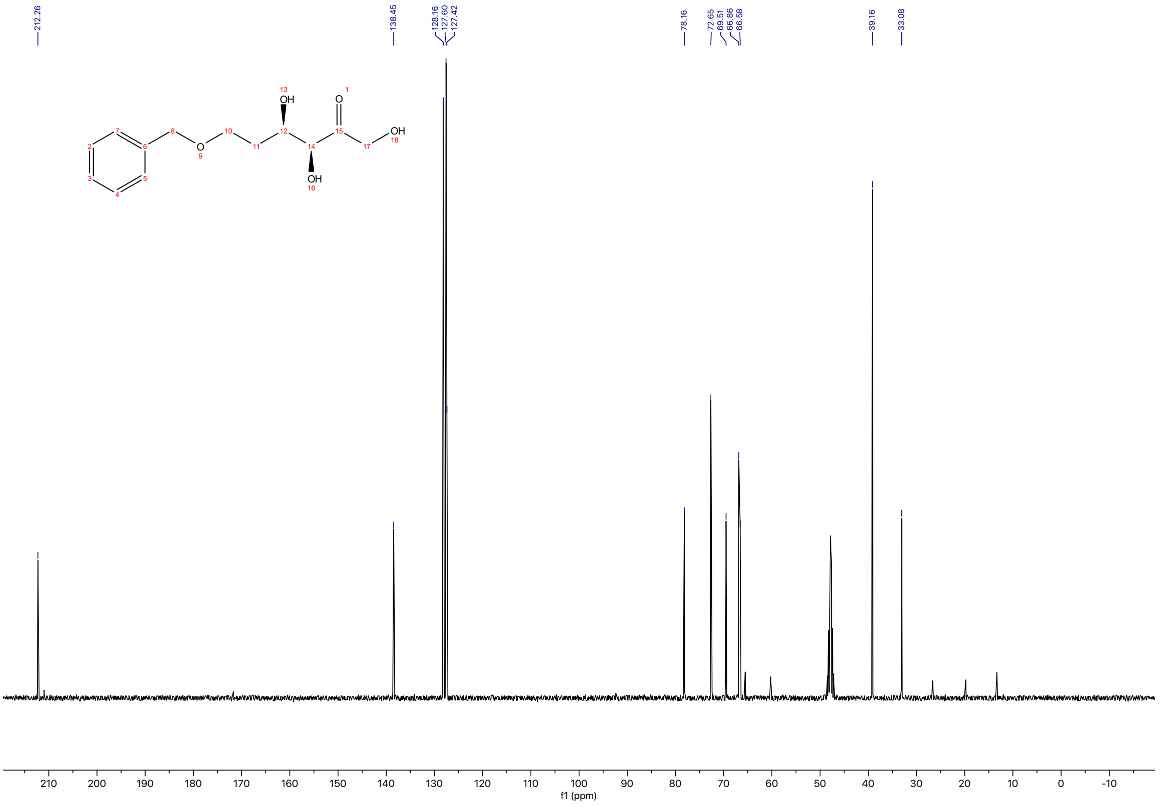
212.26
138.45
128.16 127.60 127.42
78.16
72.65 69.51 66.86 66.58
39.16
33.08
f1 (ppm)

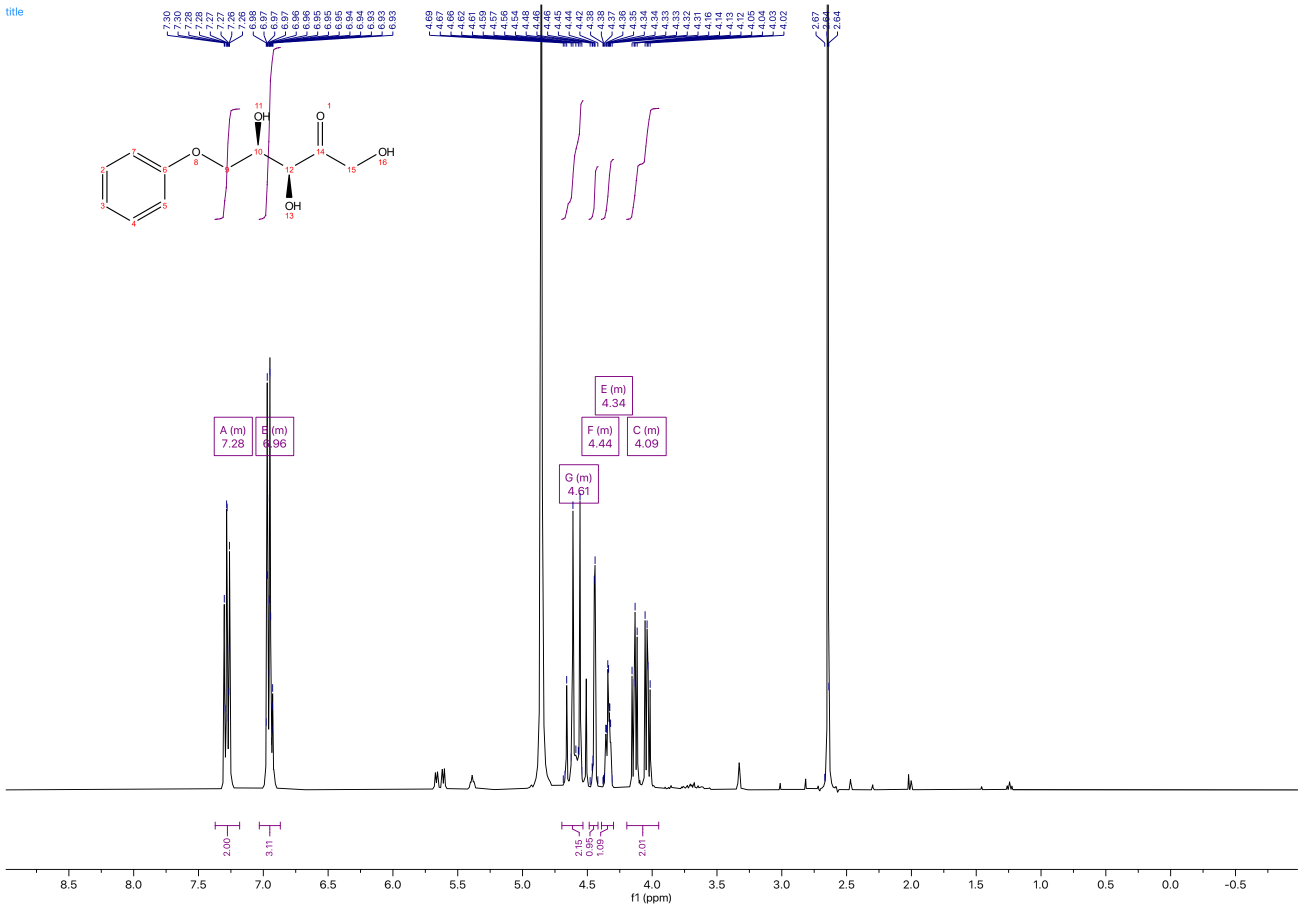
title
A (m)
7.28
B (m)
6.96
G (m)
4.61
F (m)
4.44
E (m)
4.34
C (m)
4.09
2.00
3.11
2.15
0.95
1.09
2.01
f1 (ppm)

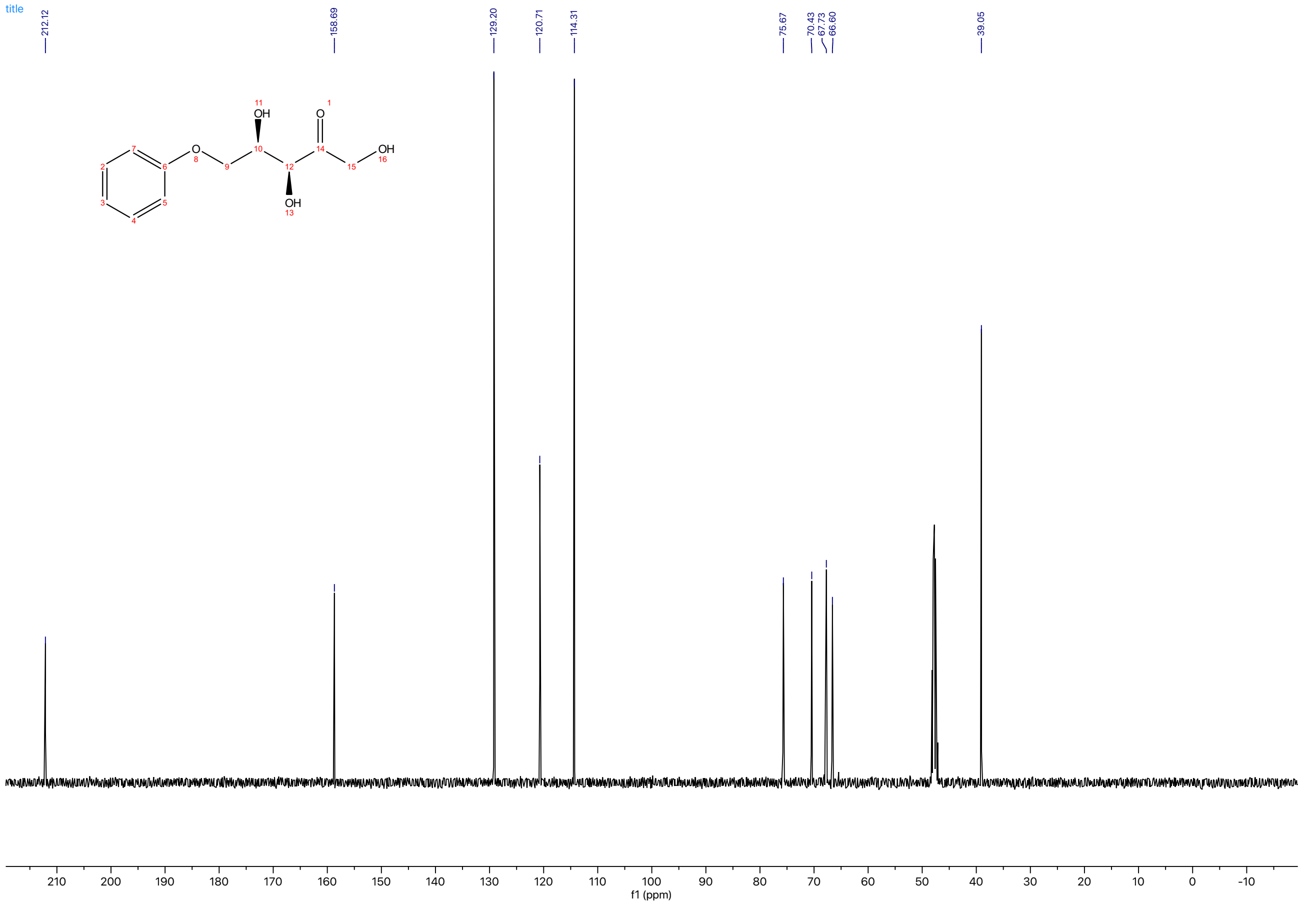
title
212.12
158.69
129.20
120.71
114.31
75.67
70.43
67.73
66.60
39.05
f1 (ppm)

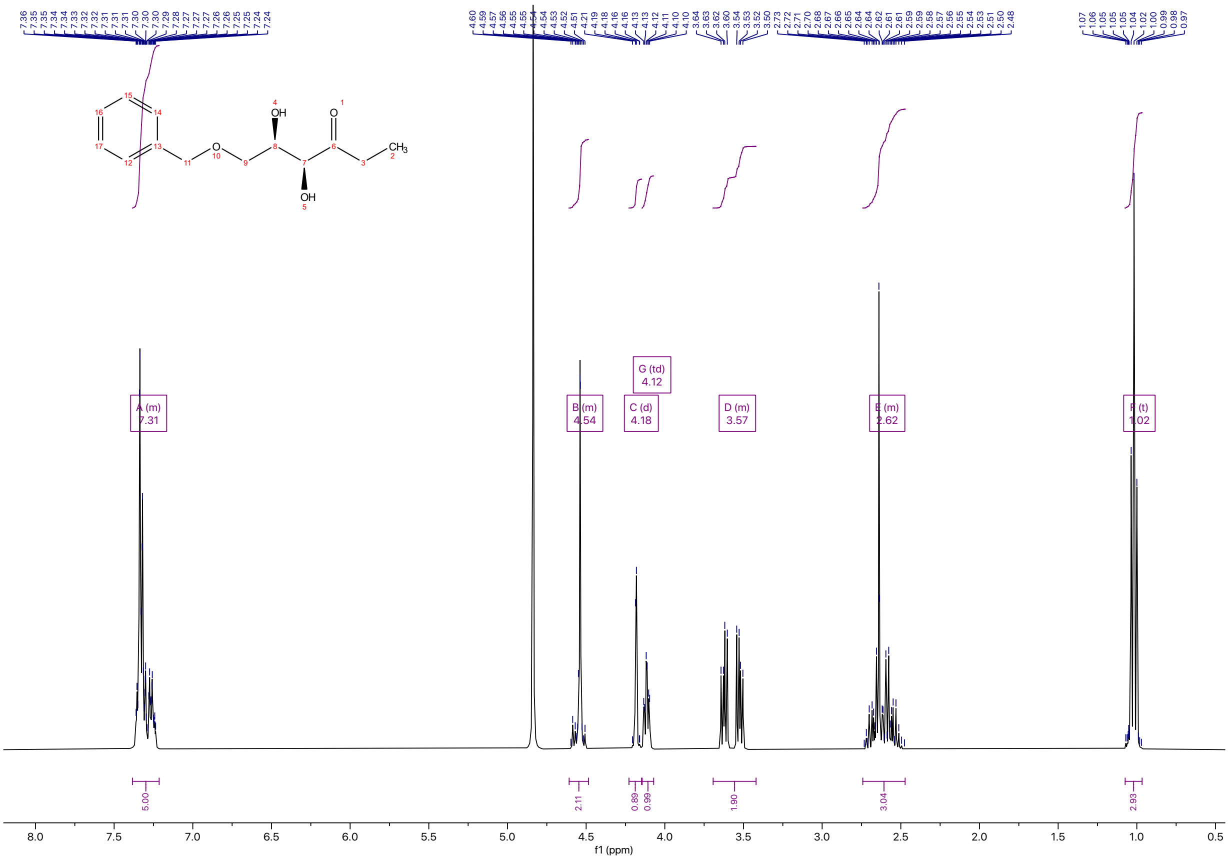
A (m)
7.31
B (m)
4.54
C (d)
4.18
G (td)
4.12
D (m)
3.57
E (m)
2.62
F (t)
1.02
5.00
2.11
0.89
0.99
1.90
3.04
2.93
f1 (ppm)

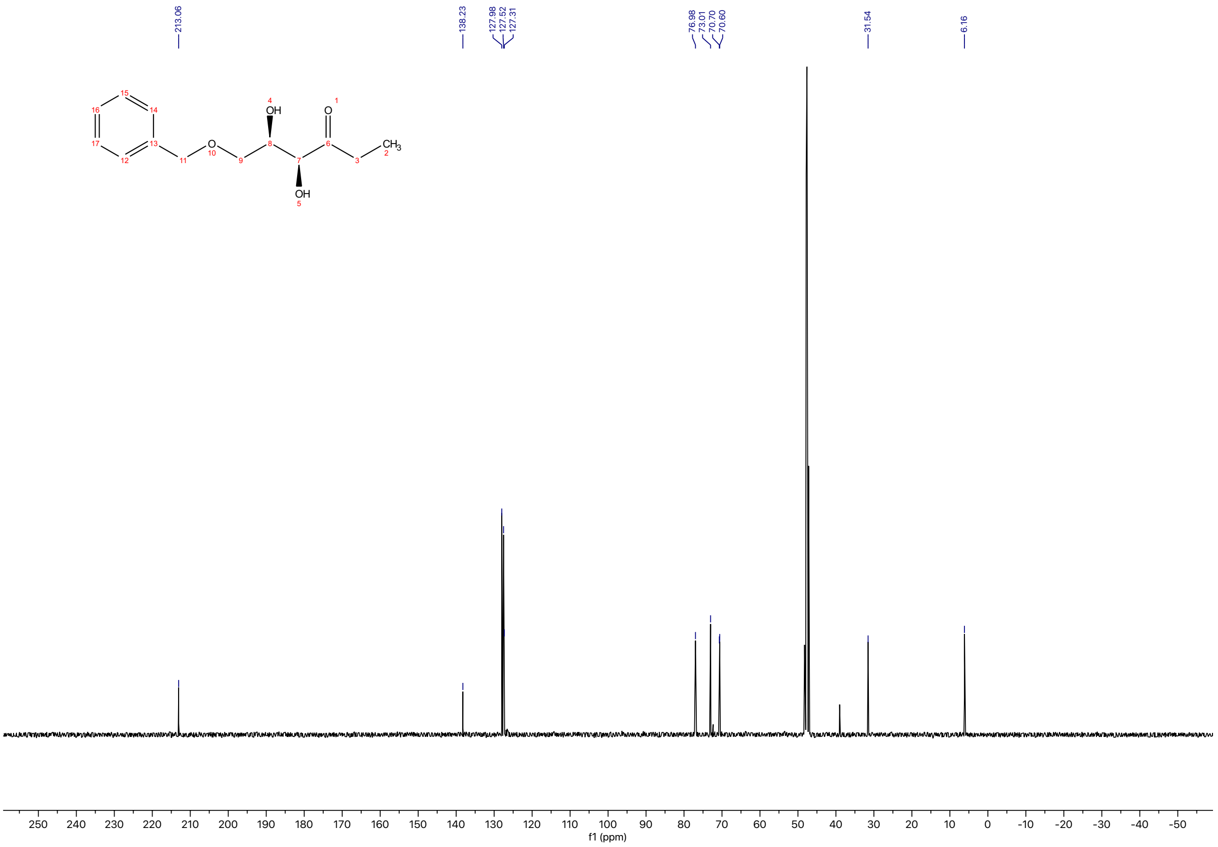
213.06
138.23
127.98
127.52
127.31
76.98
73.01
70.70
70.60
31.54
6.16
f1 (ppm)

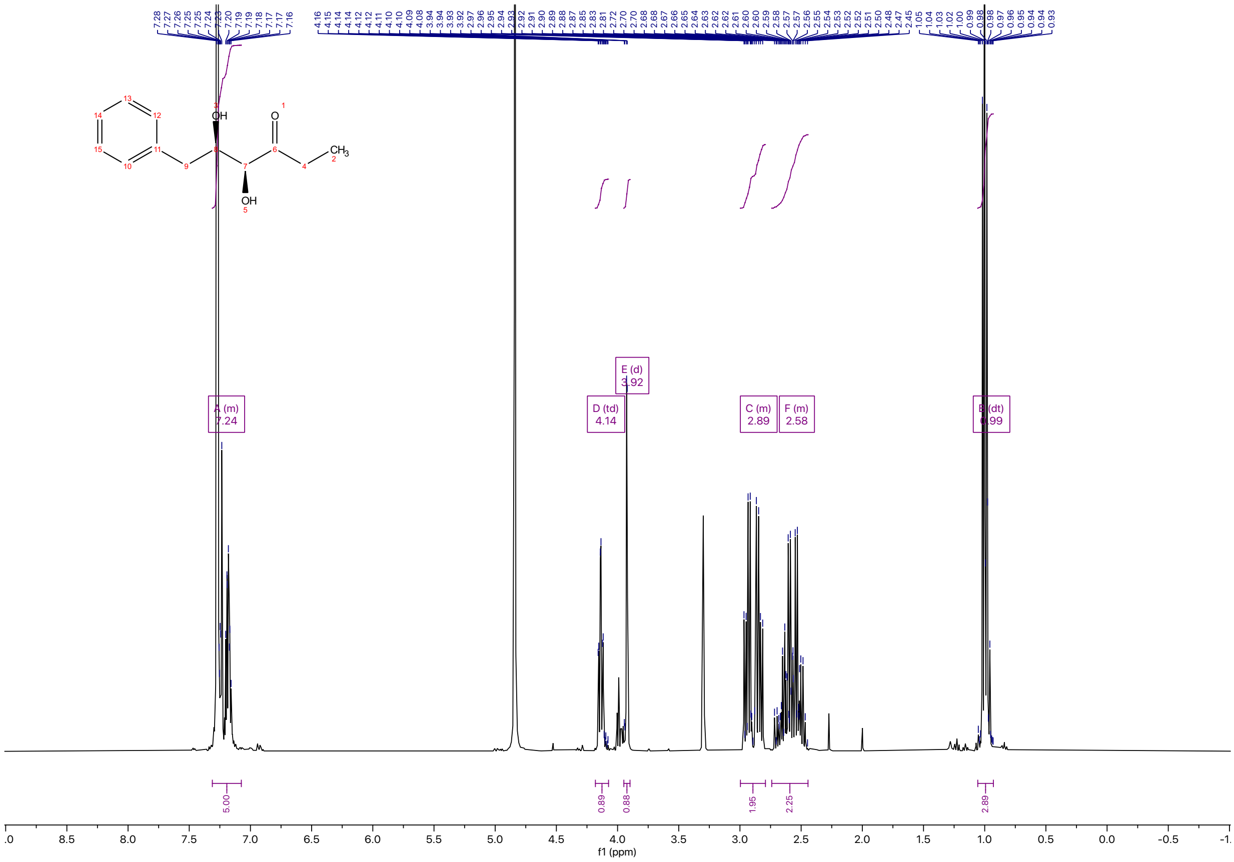
A (m)
7.24
D (td)
4.14
E (d)
3.92
C (m)
2.89
F (m)
2.58
B (dt)
0.99
f1 (ppm)

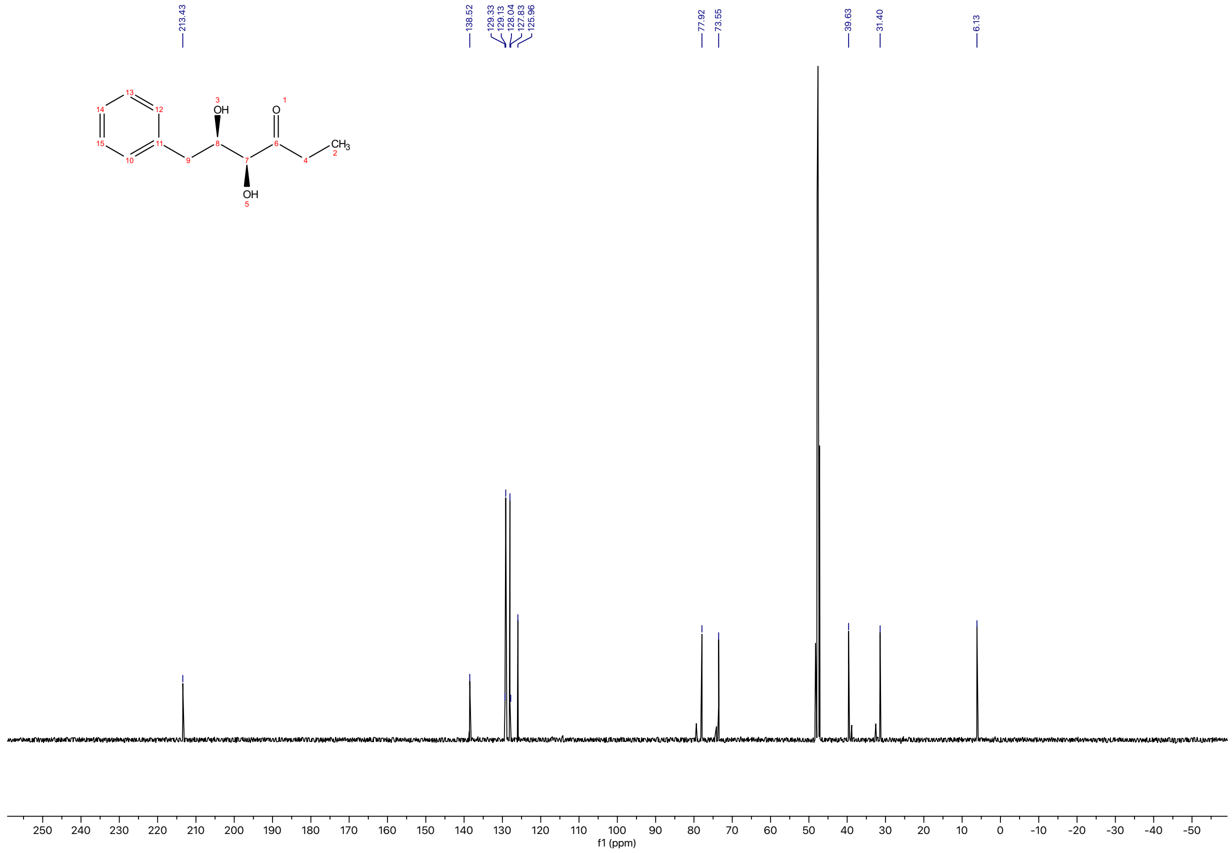
213.43
138.52
129.33
129.13
128.04
127.83
125.96
77.92
73.55
39.63
31.40
6.13
f1 (ppm)

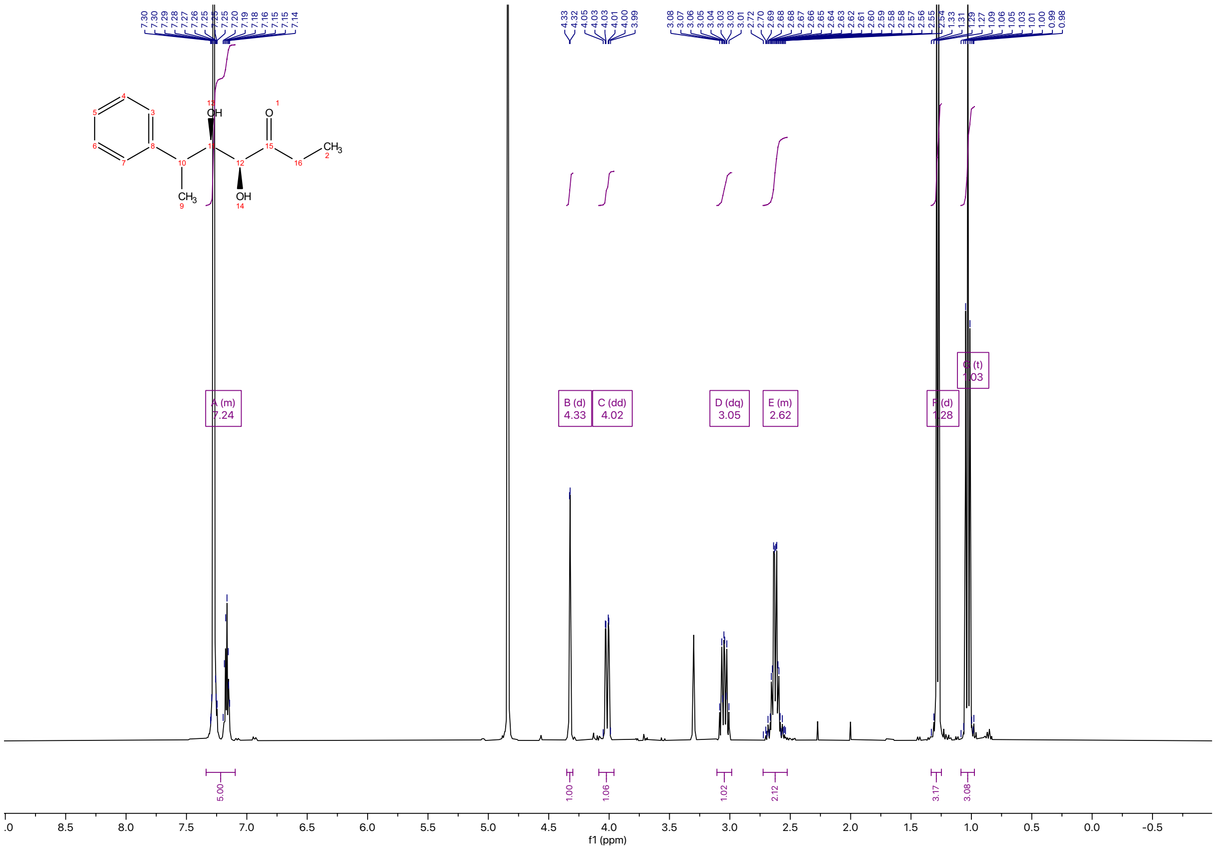

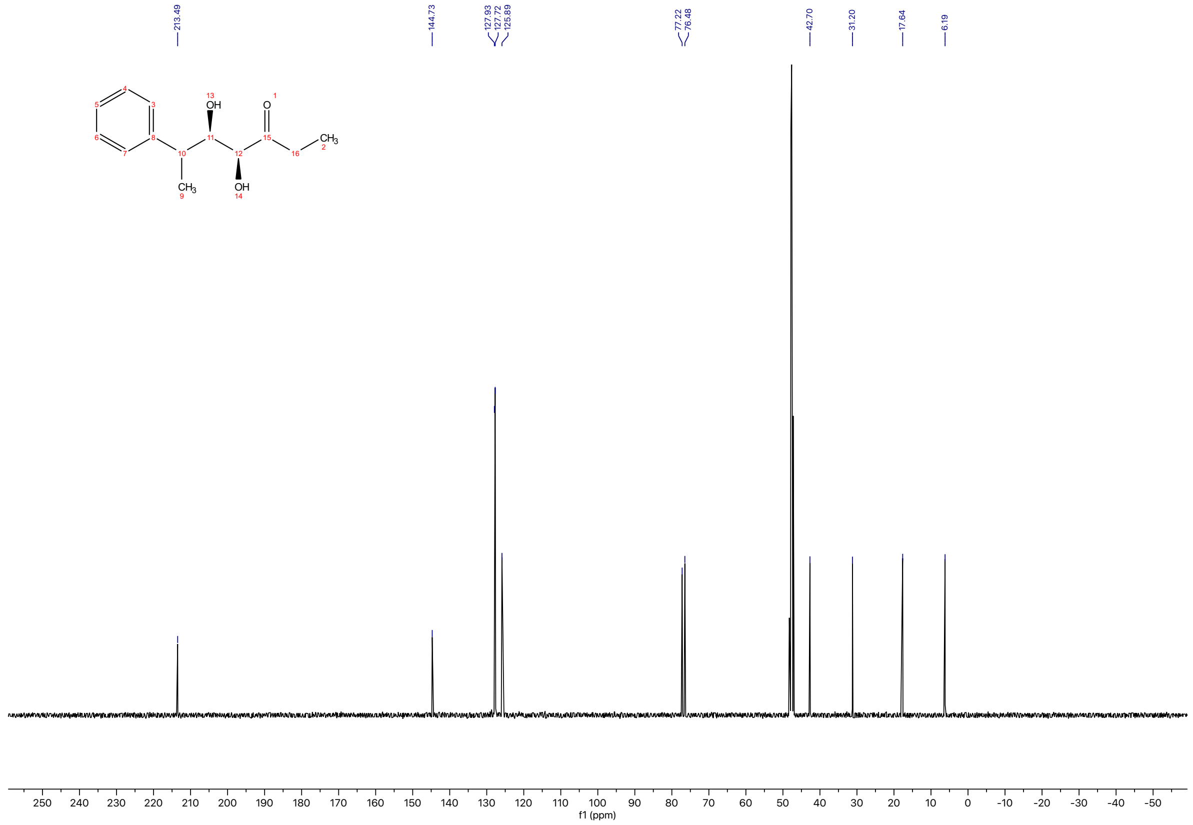

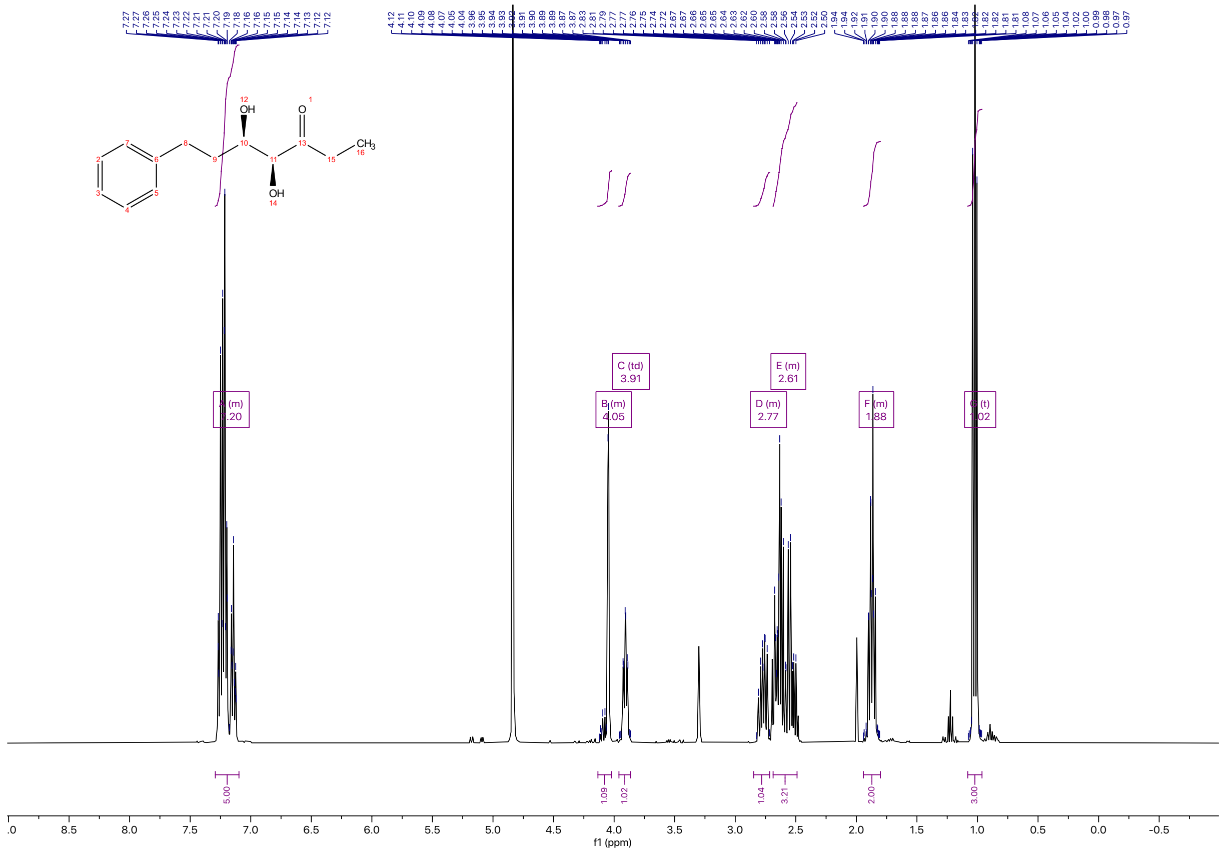

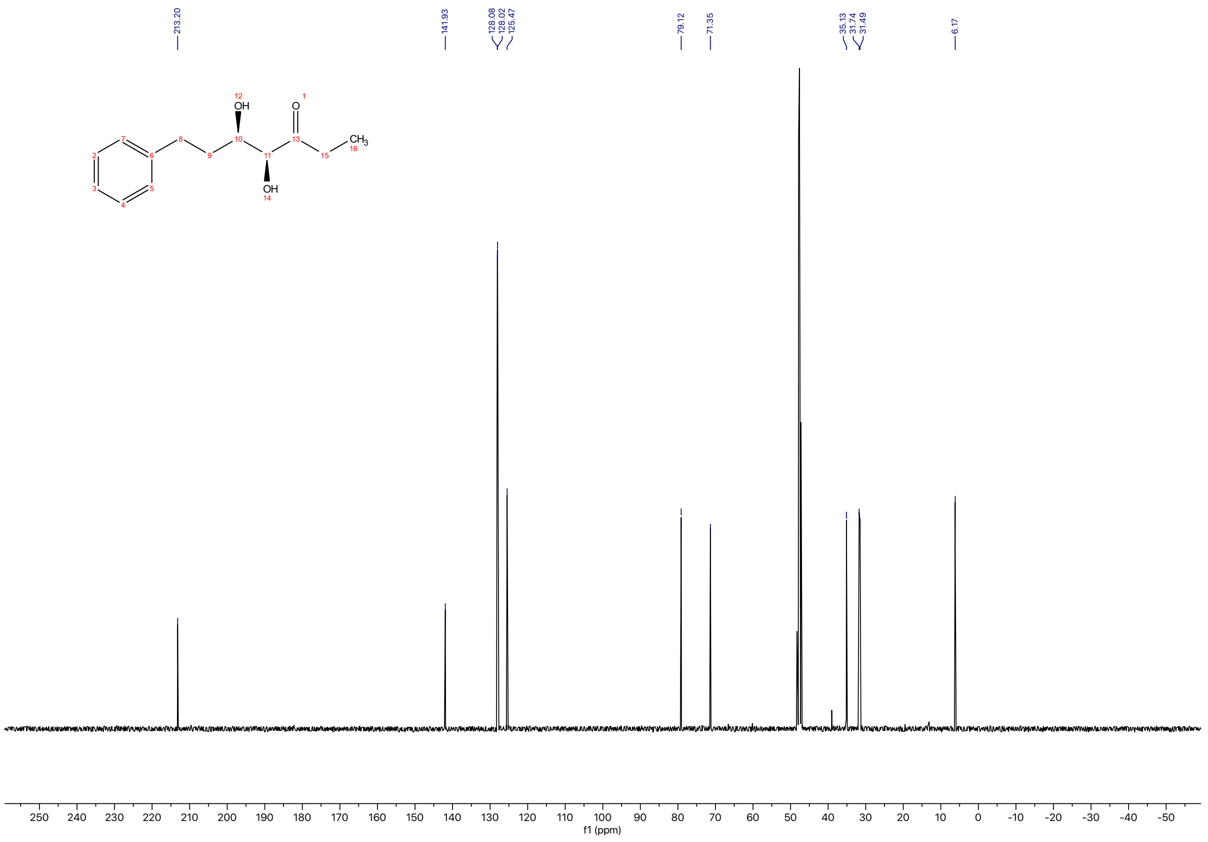

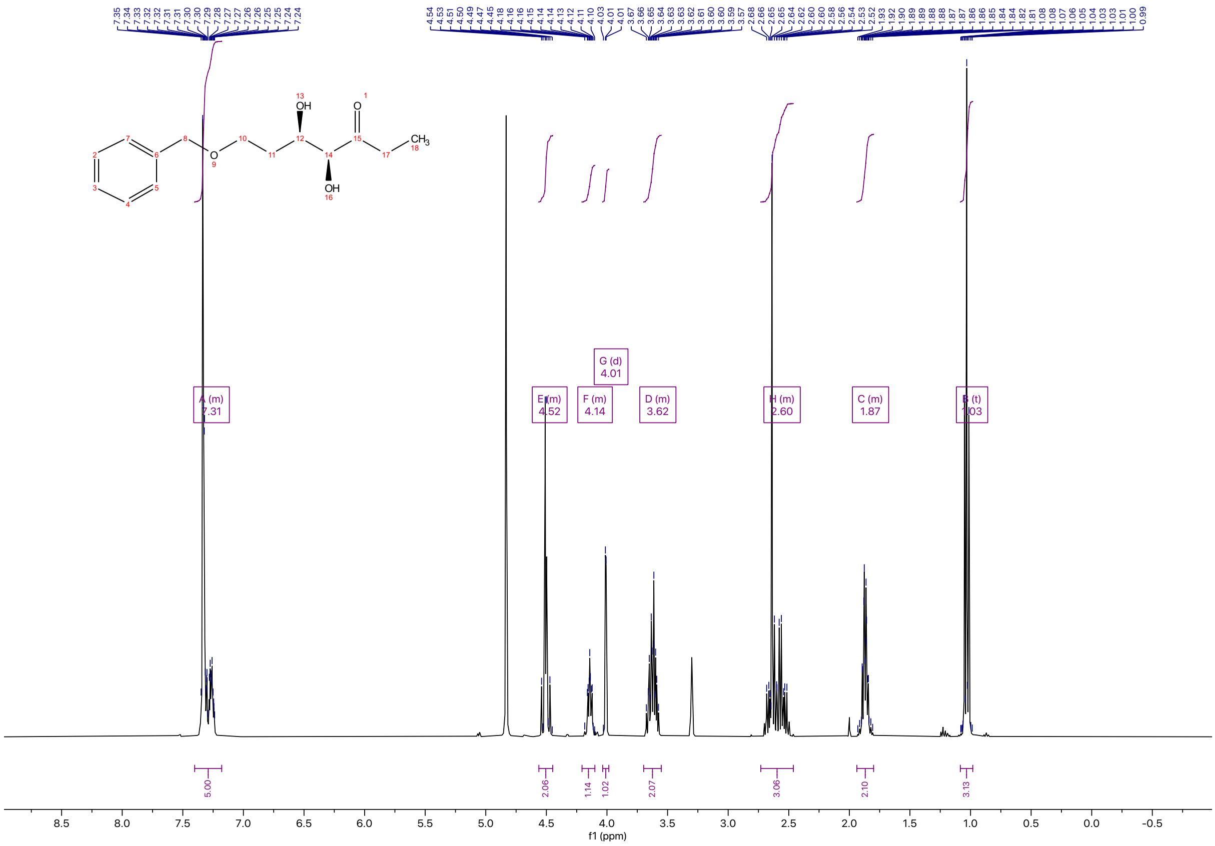

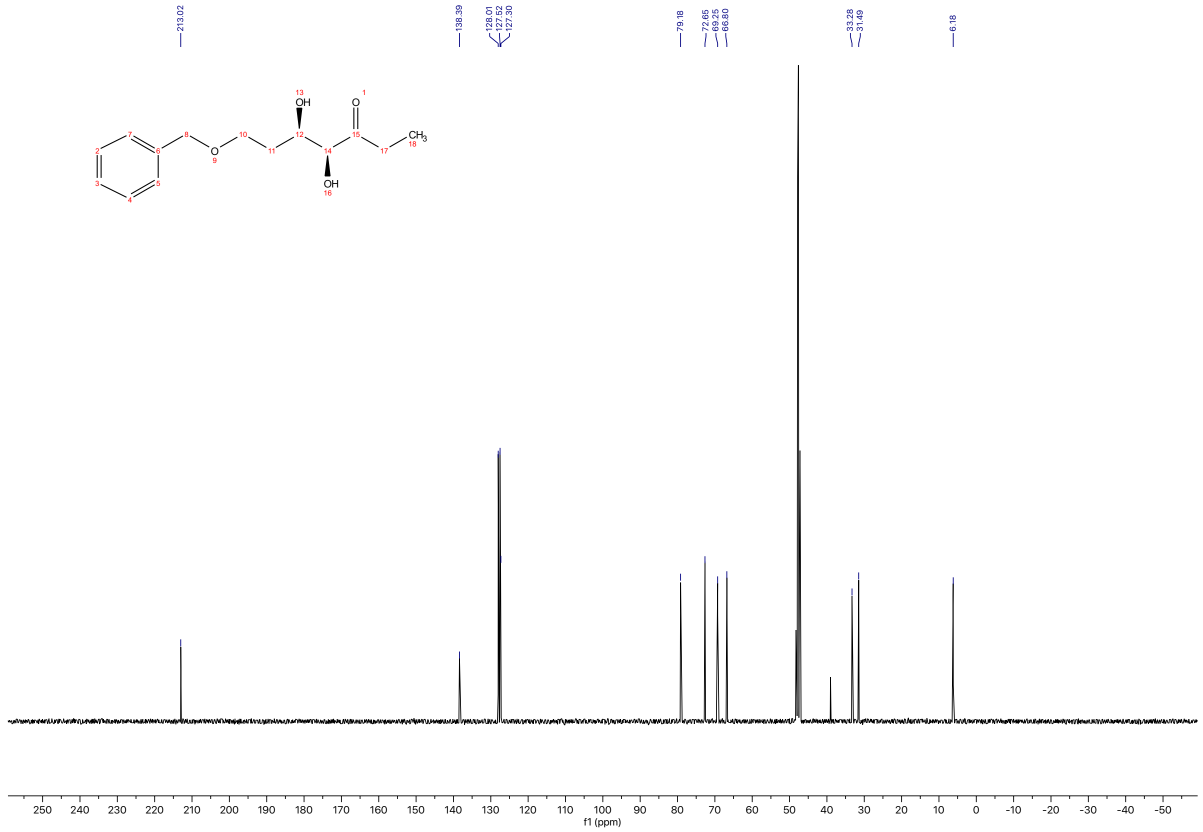

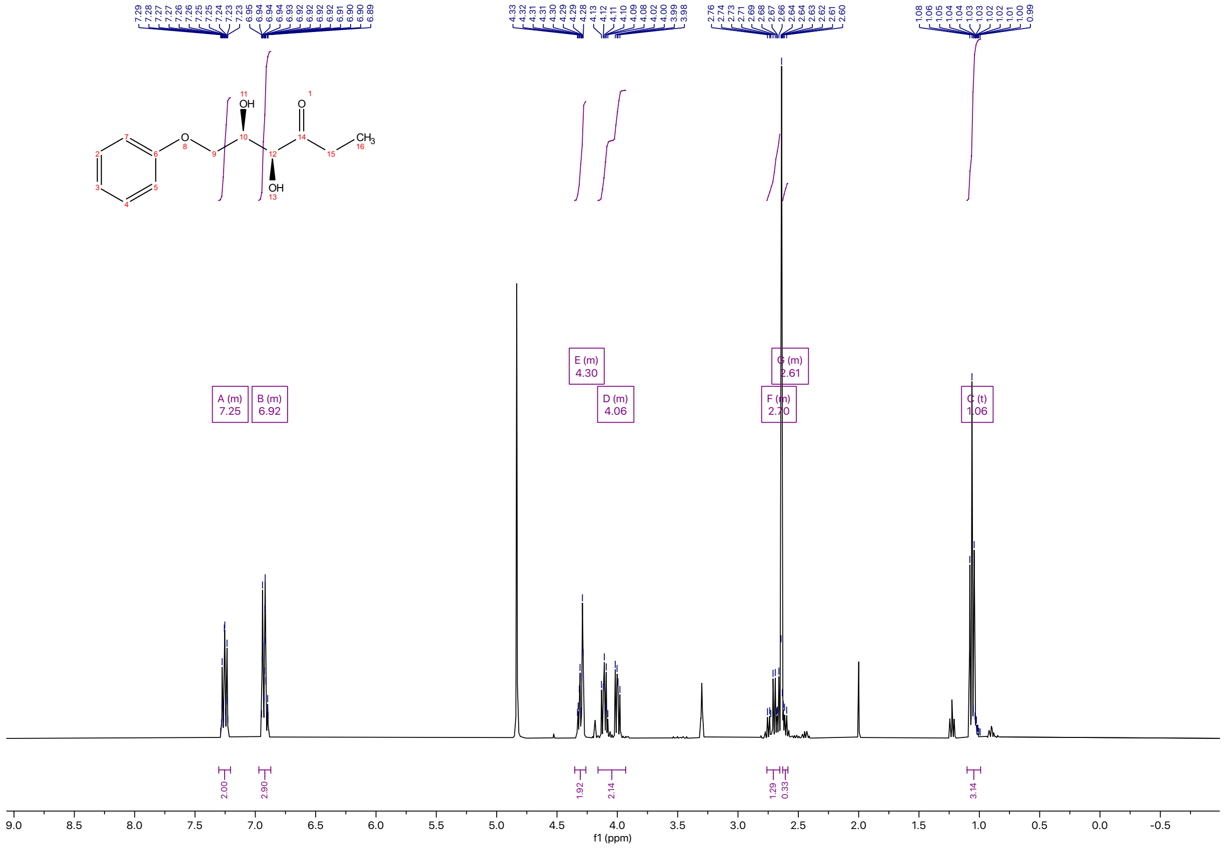

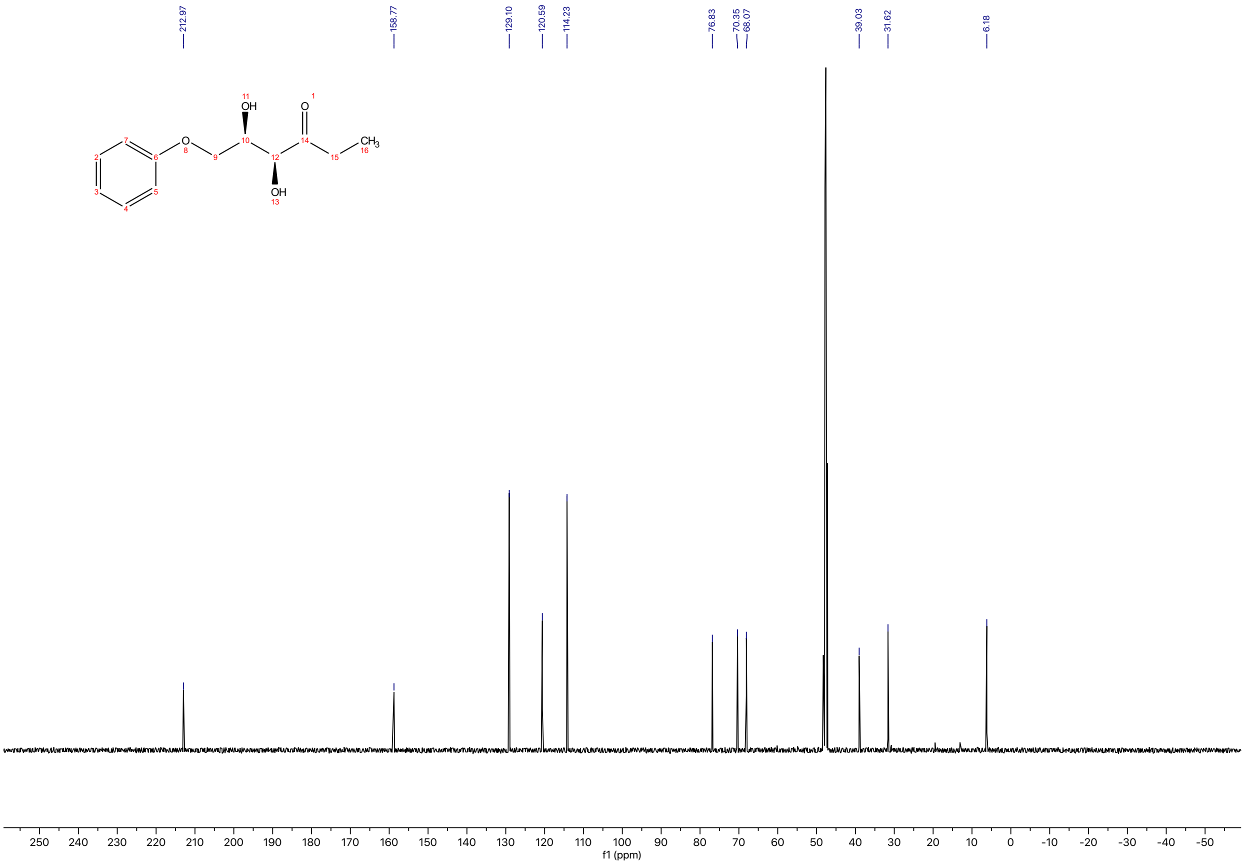

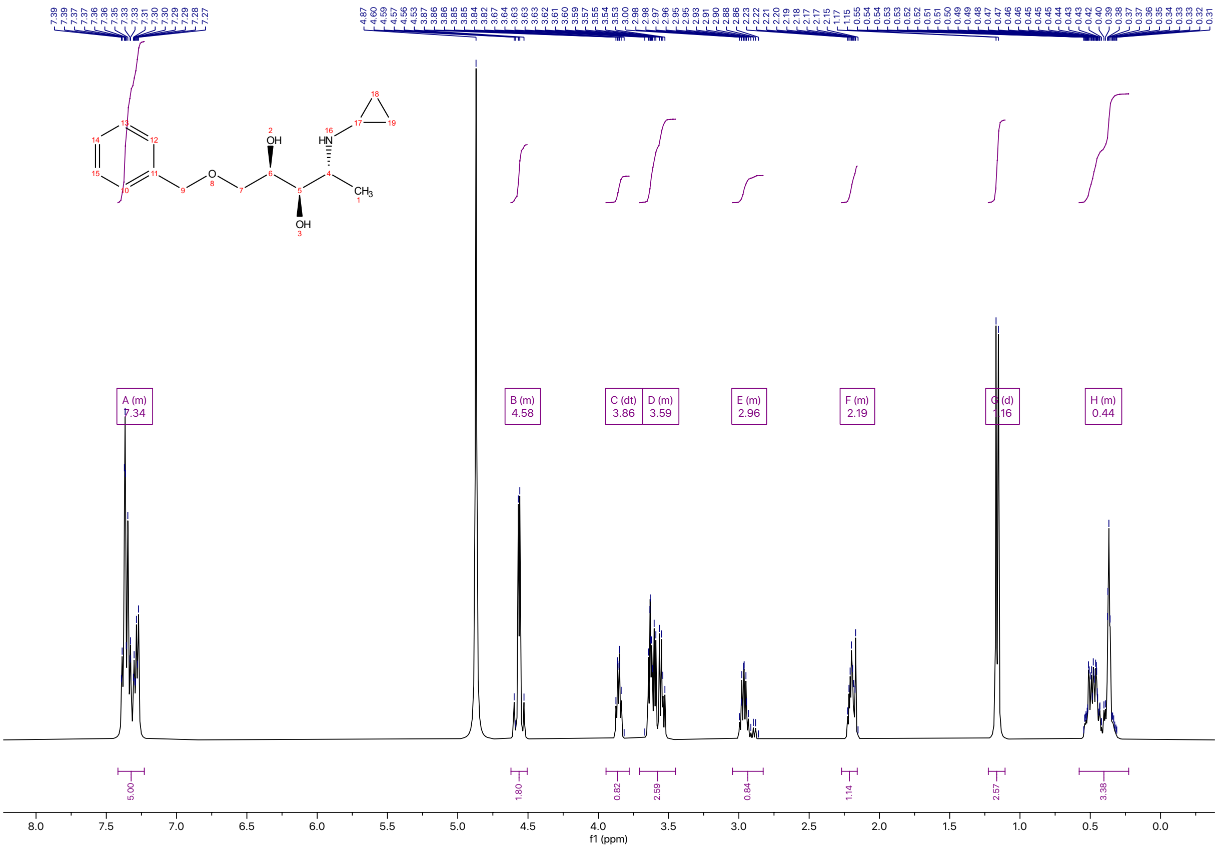

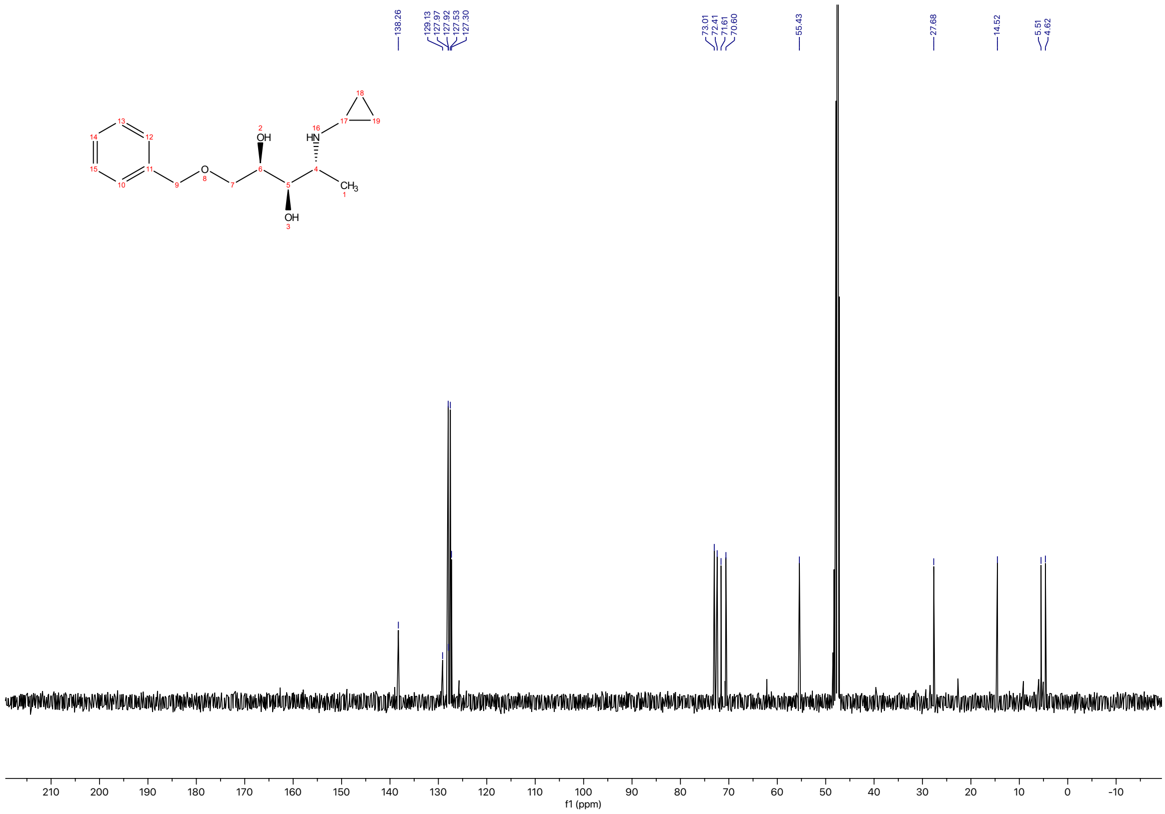

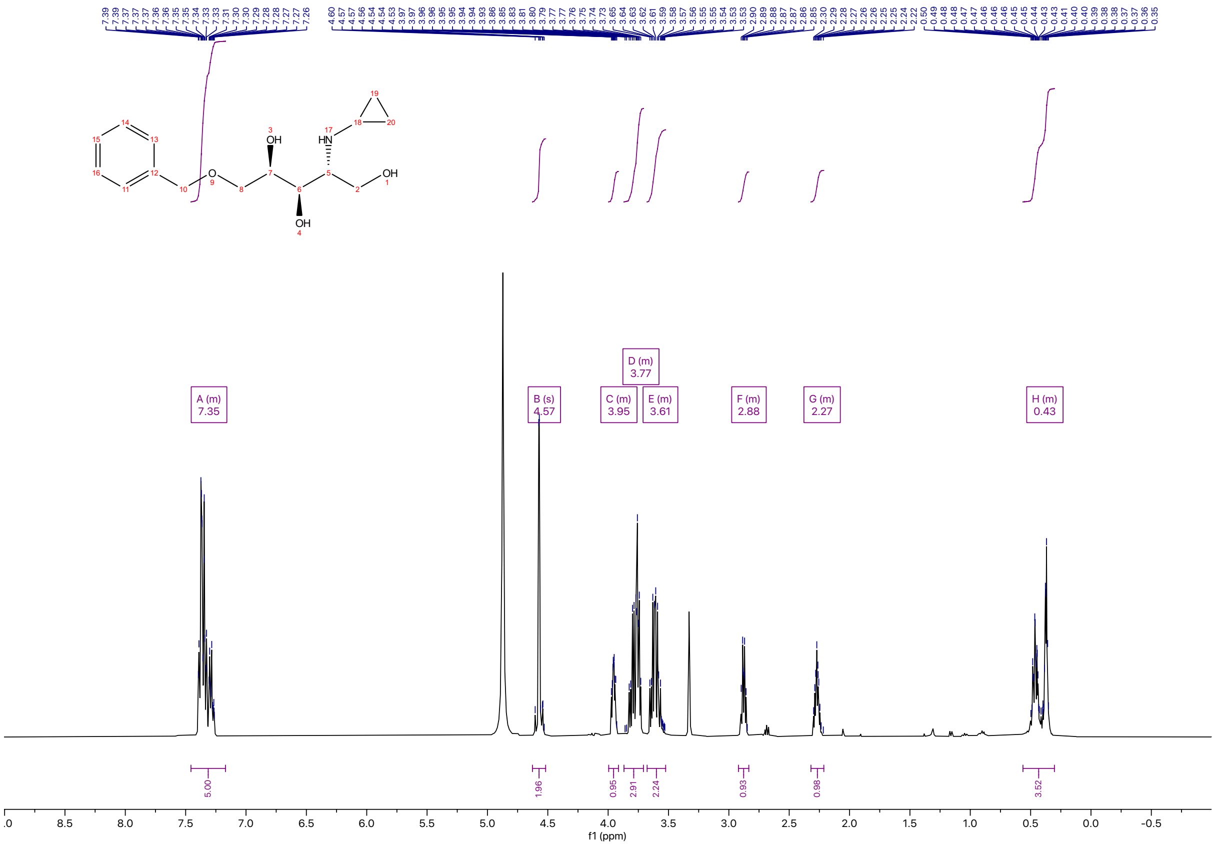

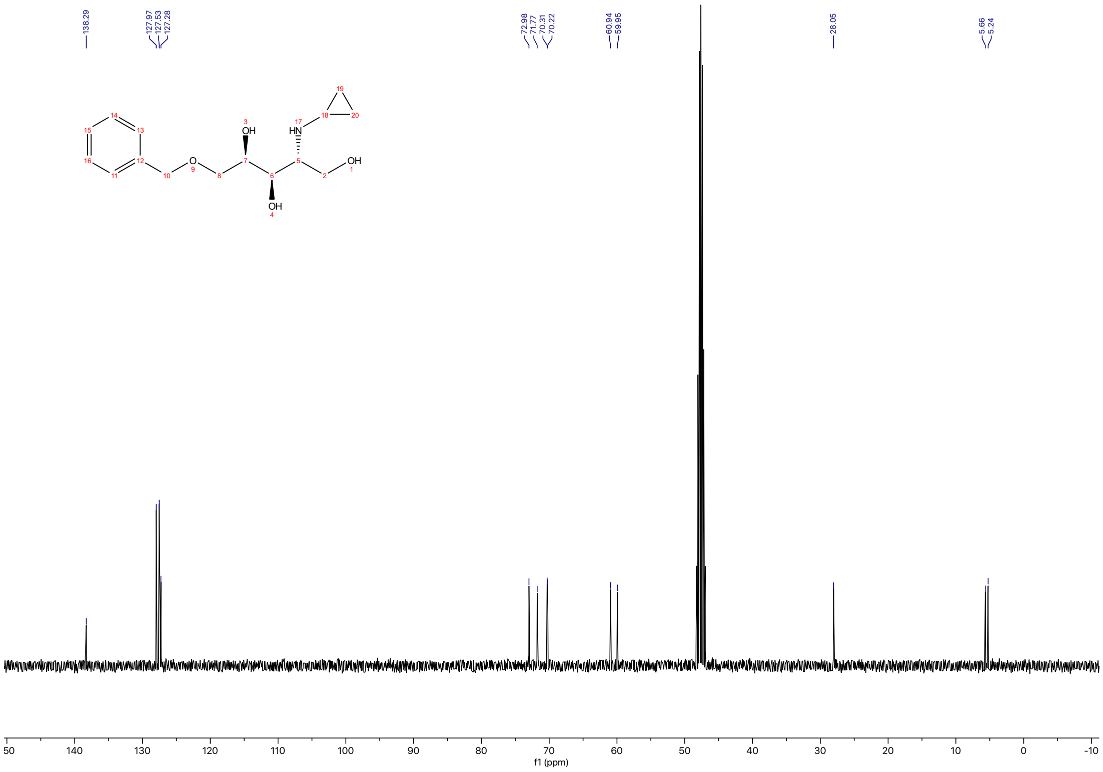

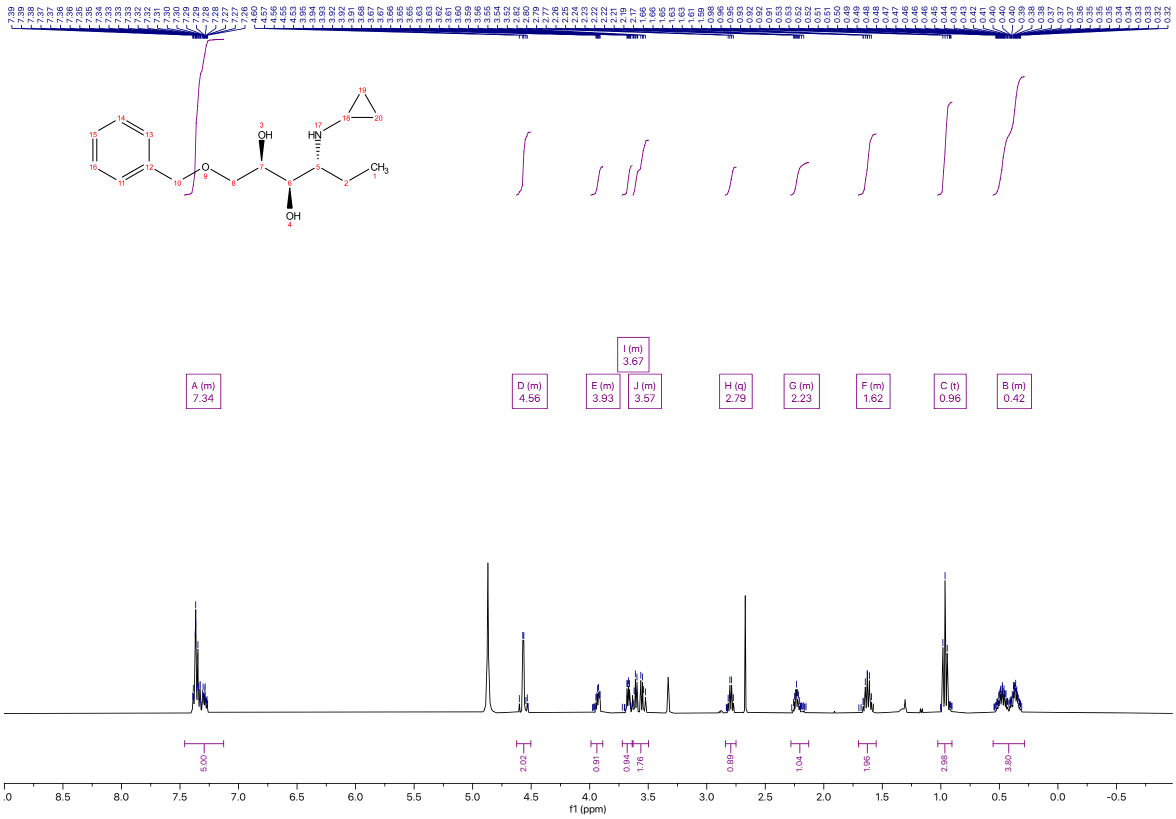

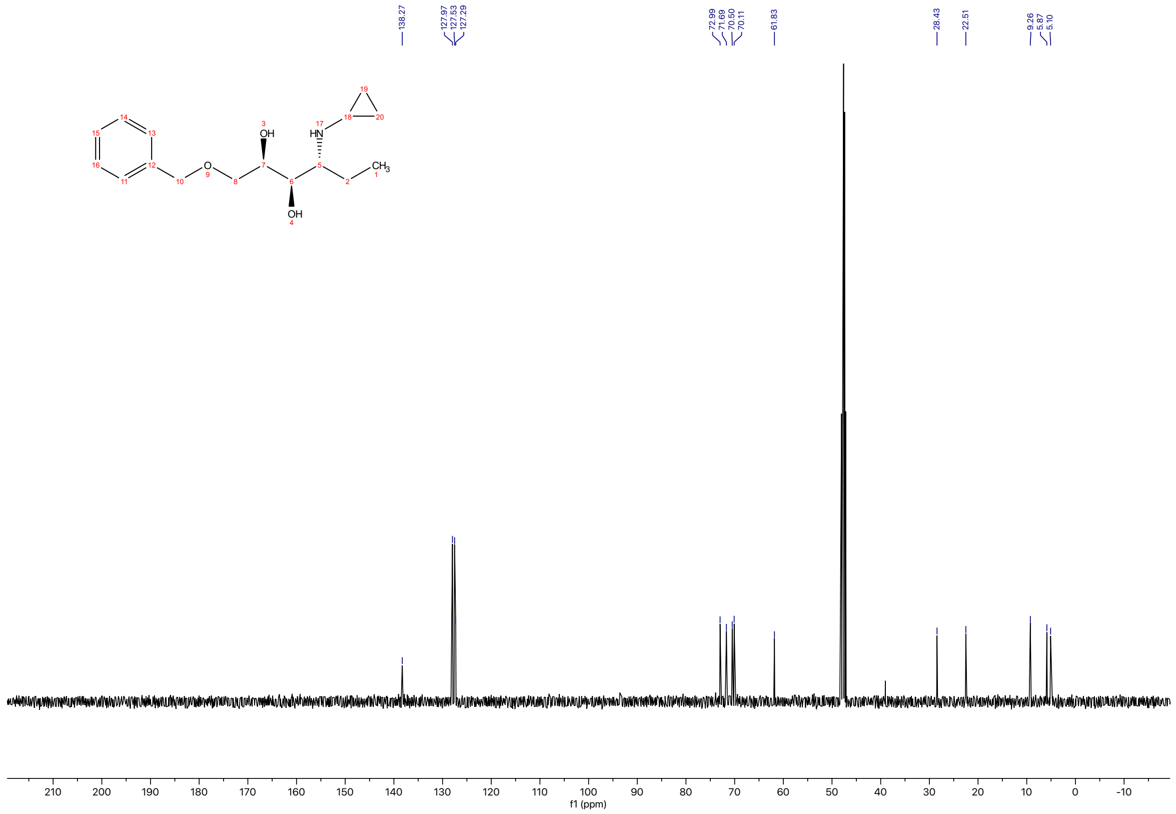

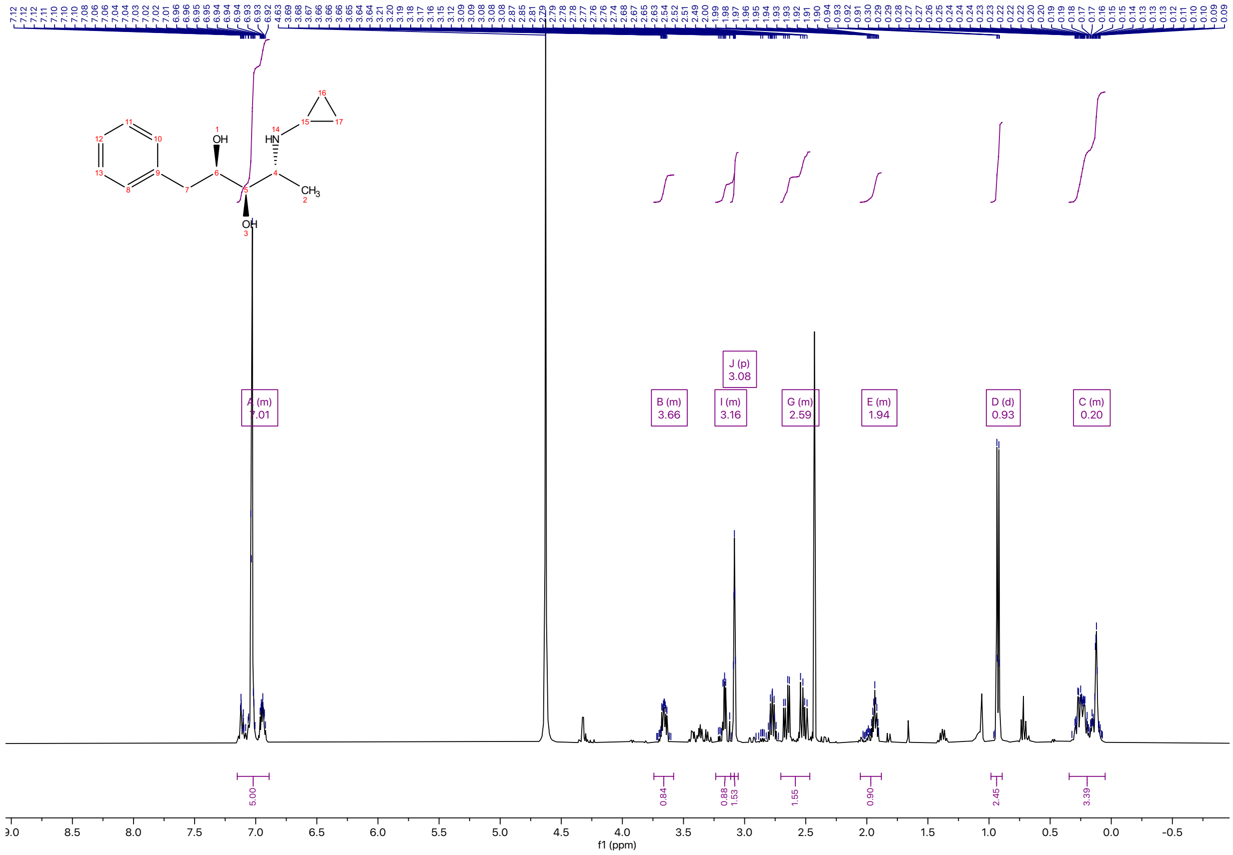

A (m)
7.01
B (m)
3.66
I (m)
3.16
J (p)
3.08
G (m)
2.59
E (m)
1.94
D (d)
0.93
C (m)
0.20
f1 (ppm)

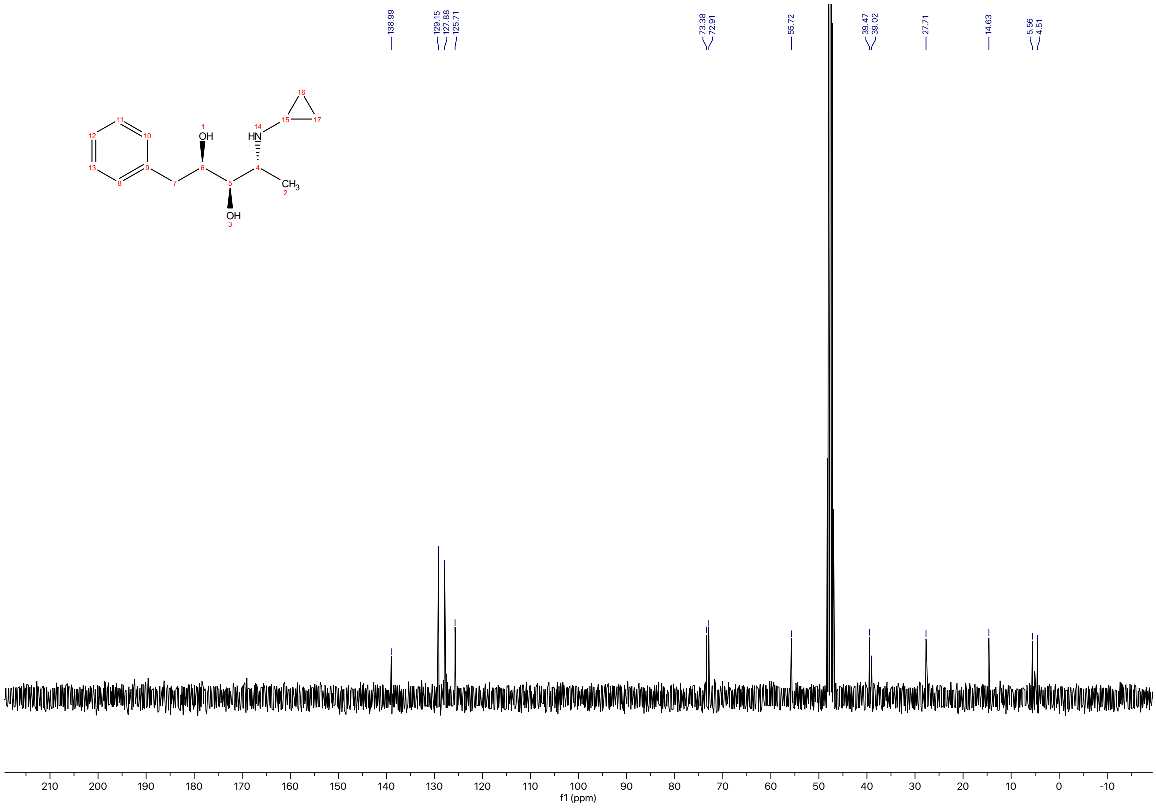

138.99
129.15
127.88
125.71
73.38
72.91
55.72
39.47
39.02
27.71
14.63
5.56
4.51
f1 (ppm)

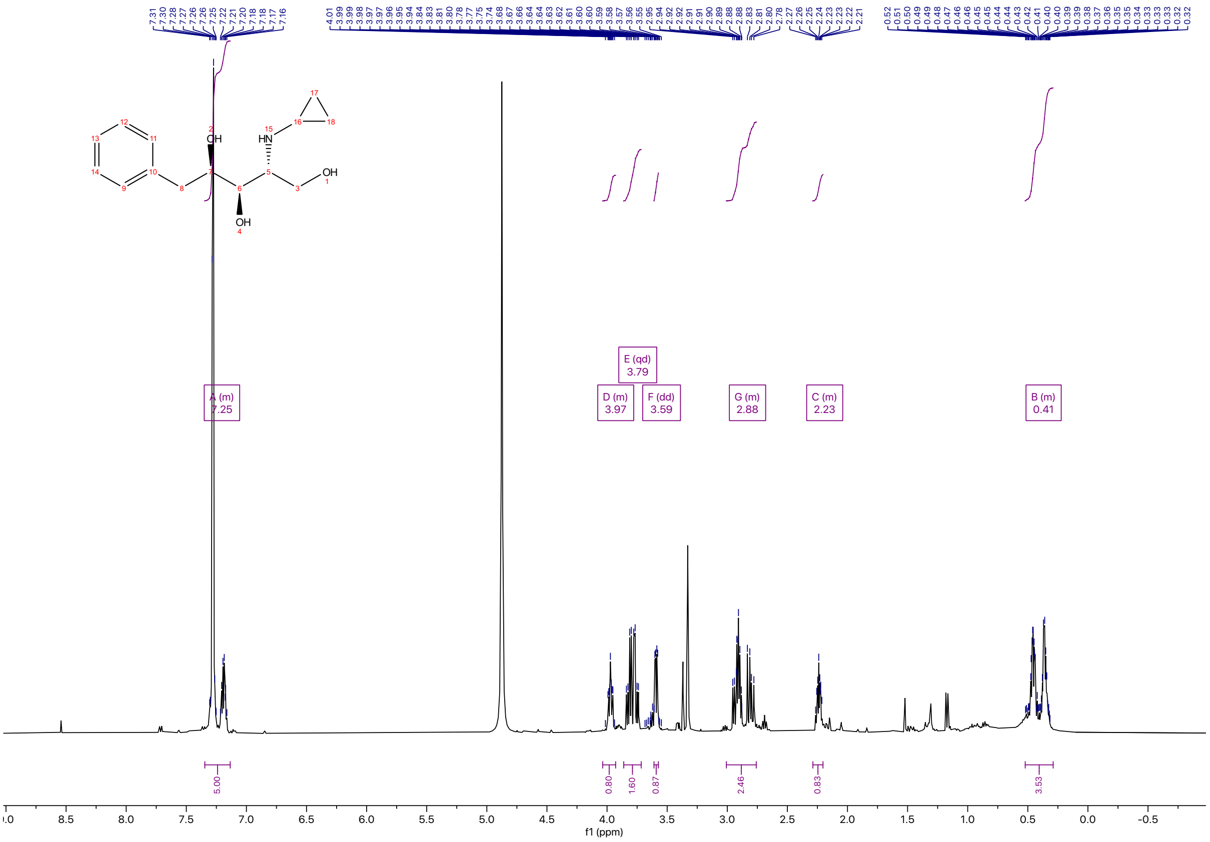
A (m) 7.25
D (m) 3.97
E (qd) 3.79
F (dd) 3.59
G (m) 2.88
C (m) 2.23
B (m) 0.41
5.00
0.80
1.60
0.87
2.46
0.83
3.53
f1 (ppm)

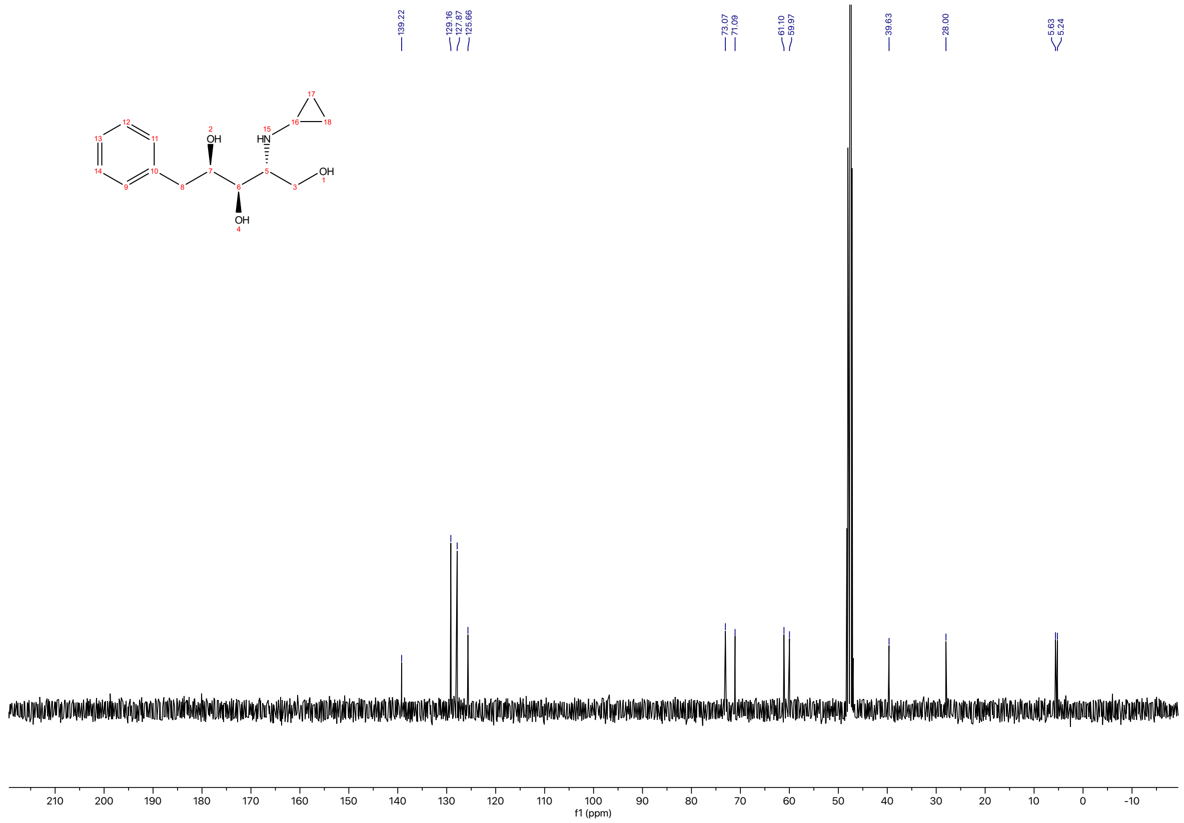
139.22
129.16
127.87
125.66
73.07
71.09
61.10
59.97
39.63
28.00
5.63
5.24
f1 (ppm)

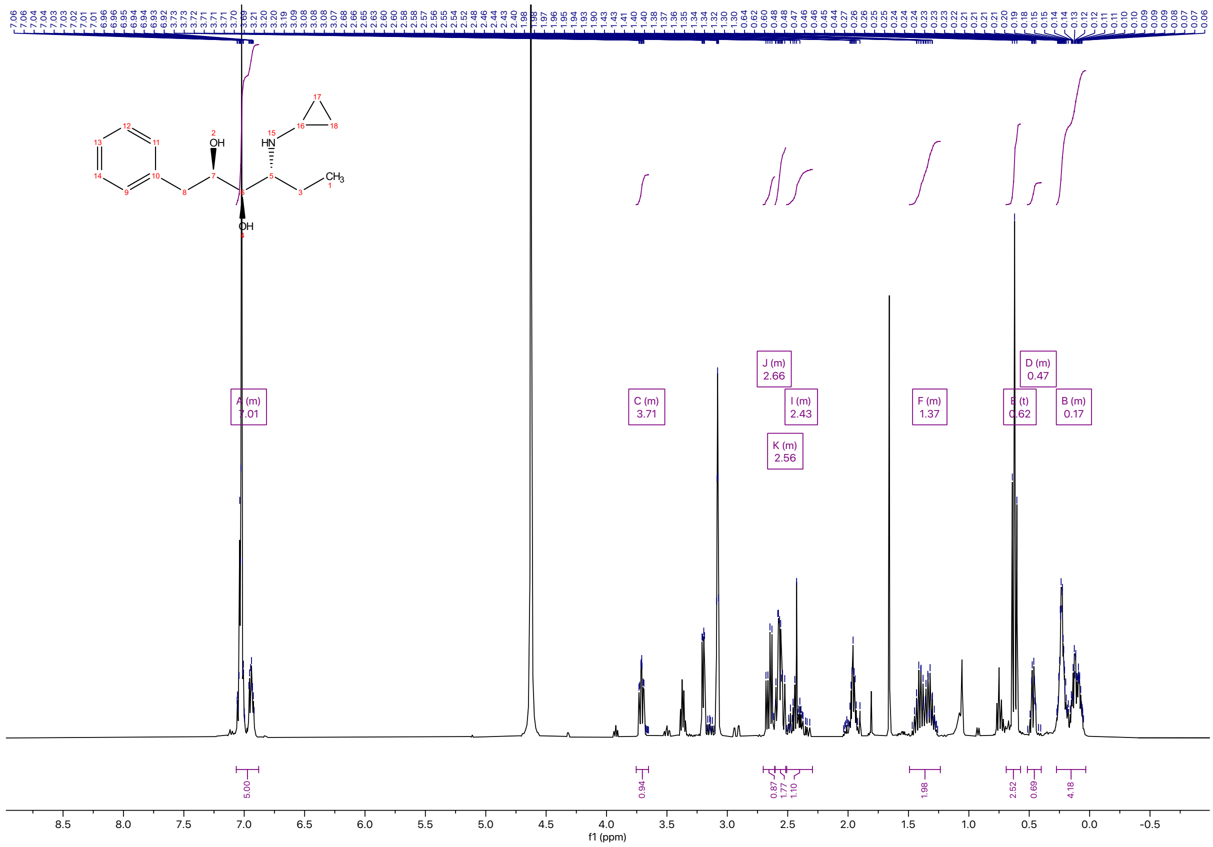
A (m)
7.01
C (m)
3.71
J (m)
2.66
I (m)
2.43
K (m)
2.56
F (m)
1.37
E (t)
0.62
D (m)
0.47
B (m)
0.17
f1 (ppm)

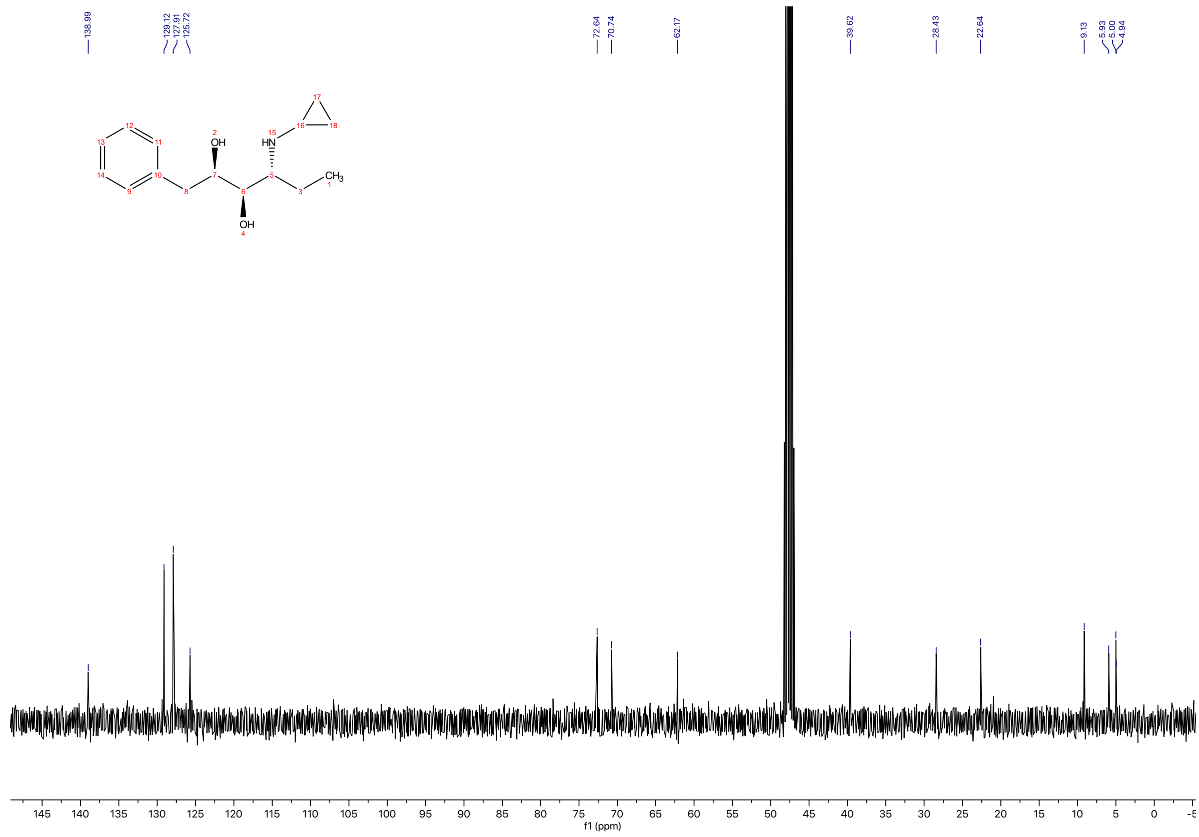
138.99
129.12
127.91
125.72
72.64
70.74
62.17
39.62
28.43
22.64
9.13
5.93
5.00
4.94
f1 (ppm)
